# Carbamoyl Radical-Mediated Synthesis and Semipinacol Rearrangement of β-Lactam Diols

**DOI:** 10.1002/chem.201304982

**Published:** 2014-04-07

**Authors:** Marie Betou, Louise Male, Jonathan W Steed, Richard S Grainger

**Affiliations:** [a] School of Chemistry, University of BirminghamEdgbaston, Birmingham B15 2TT (UK); [b] Department of Chemistry, Durham UniversitySouth Road, Durham DH1 3LE (UK)

**Keywords:** cyclization, fused-ring systems, nitrogen heterocycles, ring expansion, strained molecules

## Abstract

In an approach to the biologically important 6-azabicyclo[3.2.1]octane ring system, the scope of the tandem 4-*exo*-trig carbamoyl radical cyclization—dithiocarbamate group transfer reaction to ring-fused β-lactams is evaluated. β-Lactams fused to five-, six-, and seven-membered rings are prepared in good to excellent yield, and with moderate to complete control at the newly formed dithiocarbamate stereocentre. No cyclization is observed with an additional methyl substituent on the terminus of the double bond. Elimination of the dithiocarbamate group gives α,β- or β,γ-unsaturated lactams depending on both the methodology employed (base-mediated or thermal) and the nature of the carbocycle fused to the β-lactam. Fused β-lactam diols, obtained from catalytic OsO_4_-mediated dihydroxylation of α,β-unsaturated β-lactams, undergo semipinacol rearrangement via the corresponding cyclic sulfite or phosphorane to give keto-bridged bicyclic amides by exclusive *N*-acyl group migration. A monocyclic β-lactam diol undergoes Appel reaction at a primary alcohol in preference to semipinacol rearrangement. Preliminary investigations into the chemo- and stereoselective manipulation of the two carbonyl groups present in a representative 7,8-dioxo-6-azabicyclo[3.2.1]octane rearrangement product are also reported.

## Introduction

β-Lactams, both naturally occurring and synthetic, play a pre-eminent role as medicinally important compounds, particularly as antibiotics.[1, 2] The inherent ring strain and resultant reactivity of the four-membered ring also renders β-lactams useful intermediates for organic synthesis, particularly through ring-opening reactions at the amide bond.[2, 3] As a consequence of these dual roles in medicinal chemistry and synthesis, a wide range of methodologies have been developed for the preparation and subsequent transformation of β-lactams, both monocyclic and fused to other ring systems.[2, 4]

**Scheme 1:**
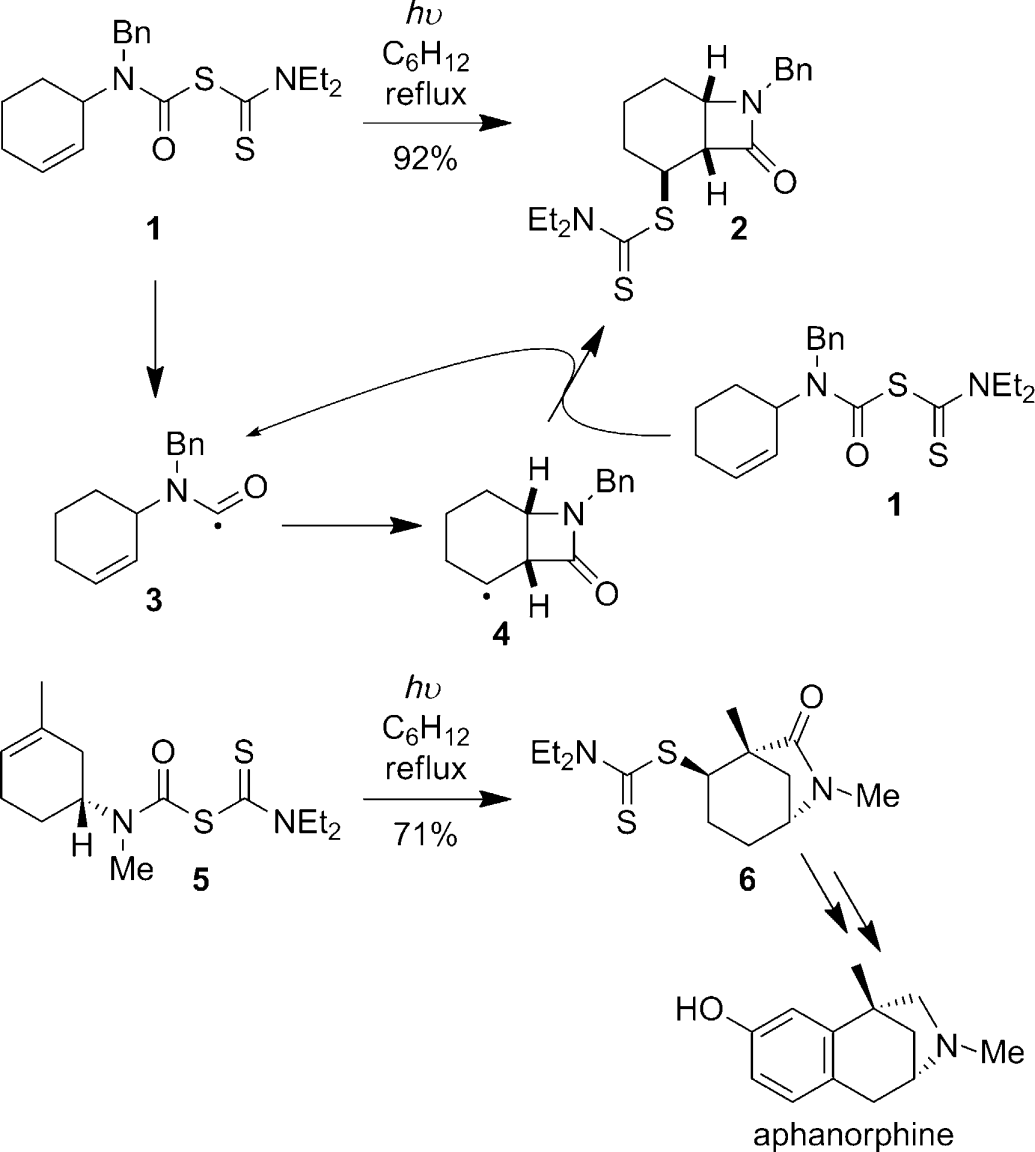
Tandem carbamoyl radical cyclization—dithiocarbamate group transfer mediated synthesis of *cis*-fused β-lactams and 6-azabicyclo[3.2.1]octane ring system.

We have previously reported a high yielding synthesis of fused β-lactam **2** through simple irradiation of readily prepared carbamoyl diethyldithiocarbamate **1** (Scheme 1).[5] 4-*Exo*-trig cyclization of carbamoyl radical **3** is followed by dithiocarbamate group transfer from **1** to the cyclohexyl radical **4** on the less hindered convex face of the bicyclic ring system.[6] In related research we have employed the regioselective cyclization of carbamoyl dithiocarbamate **5** to synthesize the bridged 6-azabicyclo[3.2.1]octane ring system **6** of aphanorphine, an alkaloid isolated from a blue–green algae.[7]

**Figure 1:**
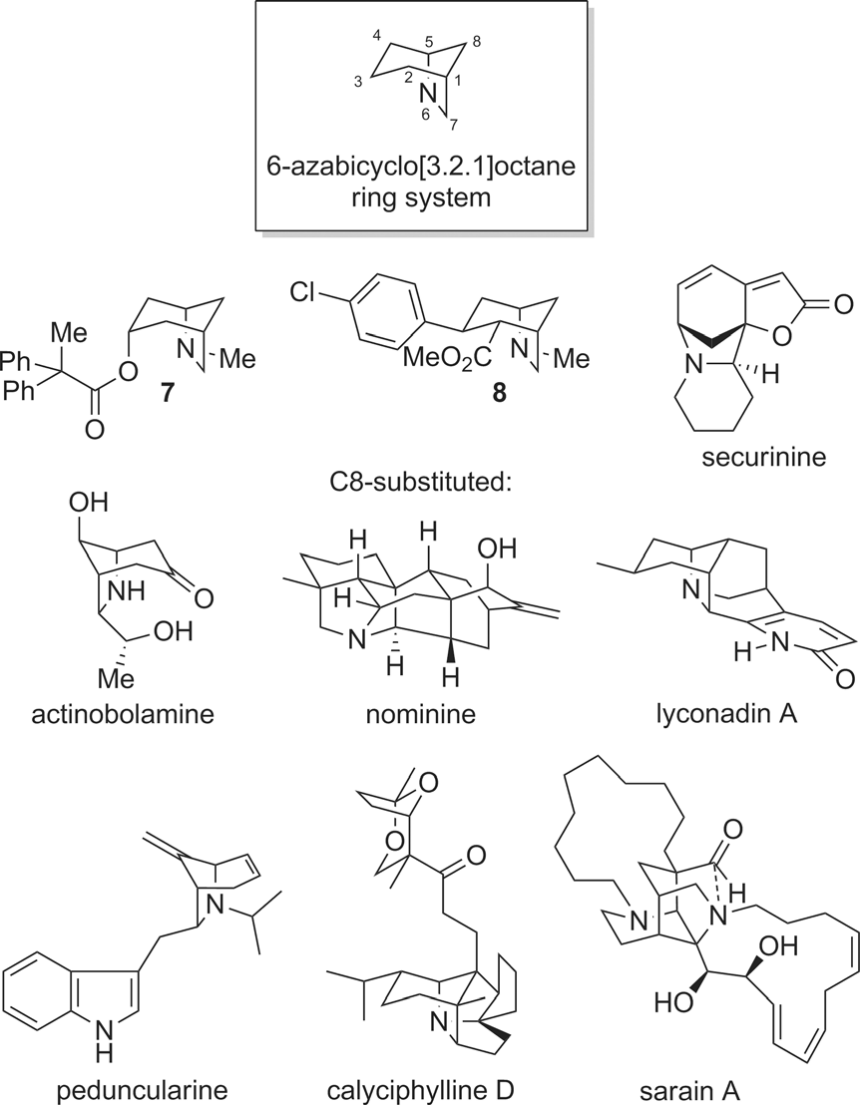
Representative natural and non-natural products containing the 6-azabicyclo[3.2.1]octane ring system.

The 6-azabicyclo[3.2.1]octane ring system is found within a range of synthetic[8]–[10] and naturally occurring,[7, 11]–[18] biologically active compounds (Figure 1). The former include **7** (“azaprophen”), a synthetic muscarinic anatagonist,[9] and **8**, a synthetic cocaine analogue and inhibitor of dopamine reuptake.[10] Representative natural products containing the 6-azabicyclo[3.2.1]octane ring system include aphanorphine,[7, 11] members of the *Securinega* alkaloids, for example securinine,[12] actinobolamine,[13] members of the hetisine alkaloids, for example nominine,[14] lyconadin A,[15] peduncularine,[16] calyciphilline D[17] and sarain A.[18] Hence this ring system has been the focus of intense synthetic interest, with a number of approaches reported in addition to those applied in target synthesis.[19]

**Scheme 2:**
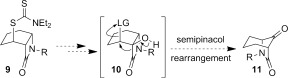
Proposed semipinacol rearrangement approach to keto-bridged 6-azabicyclo[3.2.1]octane ring system 11. LG=leaving group.

**Scheme 3:**
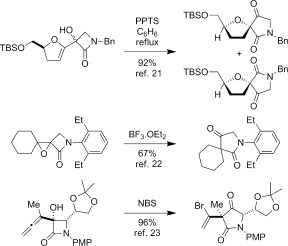
Semipinacol rearrangement of β-lactams with *N*-acyl group migration. PPTS=pyridinium *p*-toluenesulfonate.

**Figure 2:**
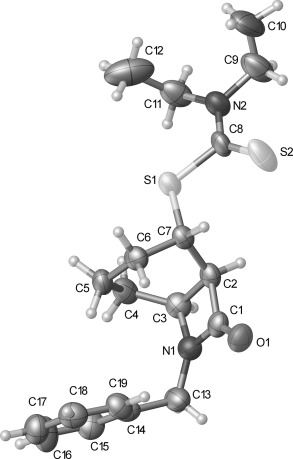
Crystal structure of β-lactam 2 with ellipsoids drawn at the 50 % probability level. The group N2, C8–C12 is disordered over two positions. Only the major component has been shown for clarity.

A challenge that must be addressed in the synthesis of many of the natural products shown in Figure 1 is the functionalization at C-8 of the 6-azabicyclo[3.2.1]octane ring system. Although potentially accessible through modification of our previous approach by incorporation of additional functionality in radical precursors, such as **5**, we instead sought to exploit the simpler, high-yielding synthesis of β-lactam **2** (Scheme 1). In principle, the bridged bicyclic amide **11**, bearing a ketone at C-8, could be prepared through a semipinacol rearrangement[20] of an oxygenated β-lactam **10**, in turn derived from dithiocarbamate **9**, the product of a group-transfer carbamoyl radical cyclization reaction (Scheme 2). Related semipinacol-like ring expansions of non-fused β-lactams to γ-lactams have been reported in the literature, and notably all occur with exclusive migration of the *N*-acyl group rather than the methylene or methine carbon atom (Scheme 3).[21]–[23] Our approach was also inspired by the X-ray crystal structure of β-lactam **2**, in which the cyclohexane ring adopts a boat-like arrangement to accommodate the *cis*-ring fusion, placing the diethyldithiocarbamate group axial with the C–S bond approximately antiperiplanar with the C–C(O) bond of the β-lactam (C1-C2-C7-S1 torsion angle −173.2 (2)°) (Figure 2).[24] In as much as **2** can be regarded as a model for the proposed rearrangement precursor **10**, migration of the *N*-acyl group of the β-lactam was expected to be preferred both electronically and stereoelectronically over migration of the *N*-alkyl group.

In this paper, we report the successful implementation of this strategy through conversion of fused β-lactams of general structure **9** in three or four steps to keto-bridged bicyclic lactams **11**. We also report studies on the scope and limitation of this methodology in our attempts to apply it to structurally related systems.[25]

## Results and Discussion

### Methodology development

**Scheme 4:**
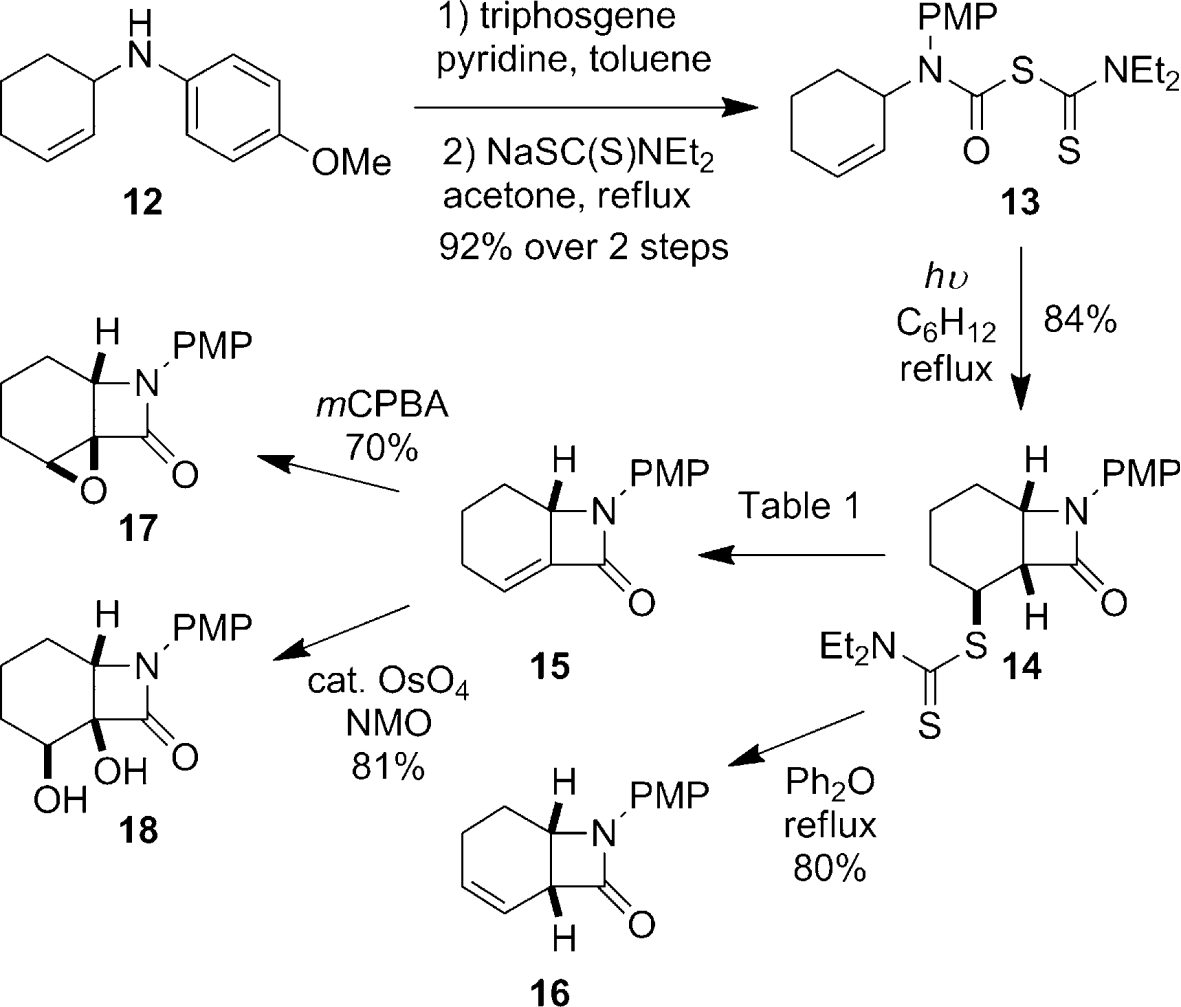
Preparation and dithiocarbamate group elimination of *N*-PMP β-lactam 14. *m*CPBA=*meta*-chloroperbenzoic acid; NMO=*N*-methylmorpholine *N*-oxide.

Methodology development was carried on *N*-*para*-methoxyphenyl (PMP) substituted lactams (Scheme 4). The PMP derivative **13** was prepared through a similar sequence to that for the *N*-benzyl system **1**.[5], 25a Treatment of *p*-methoxyaniline **12**, readily prepared through alkylation of *p*-anisidine with 3-bromocyclohexene, with triphosgene gave an isolable carbamoyl chloride intermediate of sufficient purity to be carried through to the next step without the need for further purification. Chloride displacement with commercially available sodium diethyldithiocarbamate salt was found to require more forcing conditions for an *N*-PMP substituent than for the analogous *N*-benzyl system **1** (refluxing acetone rather than room temperature), but the radical precursor **13** could nevertheless be prepared in similarly high yield. Irradiation of **13** gave the fused β-lactam **14** as a single diastereoisomer in 84 % yield. The stereochemistry at the new dithiocarbamate stereocentre was assigned based on the close spectral similarity of **14** with **2**, and the expected dithiocarbamate group transfer to the intermediate cyclohexyl radical (analogous to **4**, Scheme 1). More generally, *N*-PMP systems were found to display favourable spectroscopic and practical features, including increased crystallinity, compared with *N*-alkylated systems.

Our initial approach to convert β-lactam **14** into a suitable substrate for the proposed semipinacol rearrangement was based on incorporation of a hydroxyl group at the ring junction, and then employ the dithiocarbamate group as a latent leaving group.[26] Unfortunately attempts to deprotonate β-lactam **14** and quench the corresponding enolate with an electrophilic oxidant, a reaction that has precedent in non-fused systems,[27] met with failure.

Attention therefore turned to elimination of the dithiocarbamate group to form a ring-fused α,β-unsaturated β-lactam suitable for further oxidation. At the outset of this work we were unsure as to the feasibility of incorporating a double bond at the ring-junction of a [4.2.0] fused bicyclic β-lactam, although the analogous [5.2.0] bicyclic system had been previously prepared through a palladium-catalyzed carbonylation reaction.[28] Indeed, our previous studies on the thermal elimination of the dithiocarbamate group from **2** had shown exclusive elimination to the non-conjugaged alkene, and the same reaction conditions applied to the *N*-PMP β-lactam **14** gave alkene **16** regioselectively (Scheme 4).[29]

A screen of some common bases identified NaHMDS to be the most promising for further optimization, although avoidance of aqueous work-up proved necessary for the isolation of α,β-unsaturated β-lactam **15** (Scheme 4 and Table 1, entries 1–6). Increasing the equivalents of base or running the reaction at higher temperature resulted in inseparable mixtures of **15** and **16** (entries 7 and 8). Independent subjection of alkene **15** to 1.1 equivalents of NaHMDS in THF at room temperature for 6 h showed conversion to a 1:1 mixture of **15** and **16**, whereas under the same conditions no change occurred starting from **16**. This suggested that the formation of **16** in the base-mediated elimination reaction occurs through isomerization of **15** rather than a competing elimination pathway from **14**.

**Table 1:** Base-mediated elimination of dithiocarbamate 14.[a]

Entry	Reagent[b] (equiv)	*T* [°C]	*t* [h]	Result
1	LDA (1.1)	−78 to RT	18	**14**
2	quinoline[c]	120	18	**14**
3	NaH (1.1)	RT	26	**14**
4	*t*BuOK (1.1)	RT	6	22 % **14**, 37 % **15**
5	NaHMDS (1)	−78	4.5	22 % **14**, 46 % **15**
6	NaHMDS (1.5)	−78	4.5	47 % **15**
7	NaHMDS (3)	−78	1	42 % **15** + **16**
8	NaHMDS (1.5)	−78 to RT	7.5 then 18	46 % **15** + **16**
9	KHMDS (1.1)	−78	5.75	60 % **15**
10	LHMDS (1.1)	−78	4	61 % **15**
11	LHMDS (1.1)	0	2.75	16 % **15**
12	LHMDS (1.1)	−40	2.5	39 % **15**
13	LHMDS (1.05)+MeI (1.05)	−78	6.5	99 % **15**

[a] All reactions were carried out in THF unless otherwise stated.

[b] LDA=lithium diisopropylamide; K/Na/LHMDS=potassium/sodium/lithium hexamethyldisilazide.

[c] Quinoline was used as solvent.

Increased yields of **15** were obtained using KHMDS and LHMDS at low temperatures (Table 1, entries 9 and 10), with higher temperatures resulting in reduced yields (entries 11 and 12). Addition of MeI to activate the dithiocarbamate group towards elimination[26] caused a dramatic increase in yield, with alkene **15** the exclusive product formed in nearly quantitative yield (entry 13).

**Figure 3:**
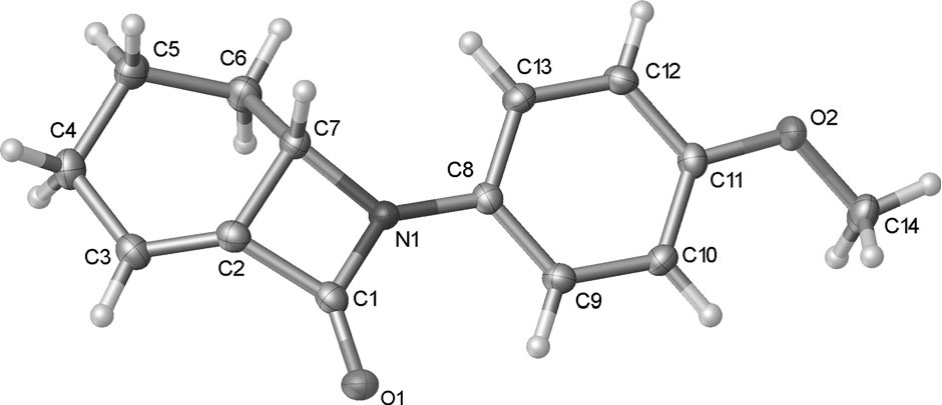
Crystal structure of alkene 15 with ellipsoids drawn at the 50 % probability level. Selected bond lengths and angles: C3-C2-C1 138.30(13), C3-C2-C7 125.47(12), C2-C3-C4 120.17(13), C7-C2-C3-C4 −5.1(2), C1-C2-C3-C4 −145.14(16)°.

The strain inherent in alkene **15** is evident in the X-ray crystal structure (Figure 3).[24] There is a notable deviation from planarity in the alkene, as evidenced in the C1-C2-C3-C4 torsion angle (−145.14(16)°). The C3-C2-C1 bond angle (138.30(13)°) is also larger than expected. This strain may account for the isomerisation of **15** to the non-conjugated alkene **16** under certain conditions.

Treatment of alkene **15** with *m*CPBA gave epoxide **17** in reasonable yield as long as a non-aqueous work-up was employed.[30] Osmium tetraoxide-catalyzed dihydroxylation gave diol **18** stereoselectively (Scheme 4).[31] Both **17** and **18** were assigned as *cis*-fused β-lactams. The corresponding *trans*-fused β-lactams would be considerably more strained and hence unlikely to form under these conditions.[32]

The semipinacol rearrangement of epoxide **17** was first attempted. Treatment of **17** with BF_3_ gave no reaction at low temperature (−78 °C, CH_2_Cl_2_), and decomposition upon warming to room temperature. The use of TiCl_4_ resulted in ring-opening of the epoxide to chloroalcohol **19**. However, treatment of **17** with PPTS in refluxing toluene gave tosylate **20** in 42 % yield after 2.25 h, and encouragingly the desired rearrangement product **21** when the reaction time was increased to 18 h, albeit in a moderate 33 % yield.

**Figure 4:**
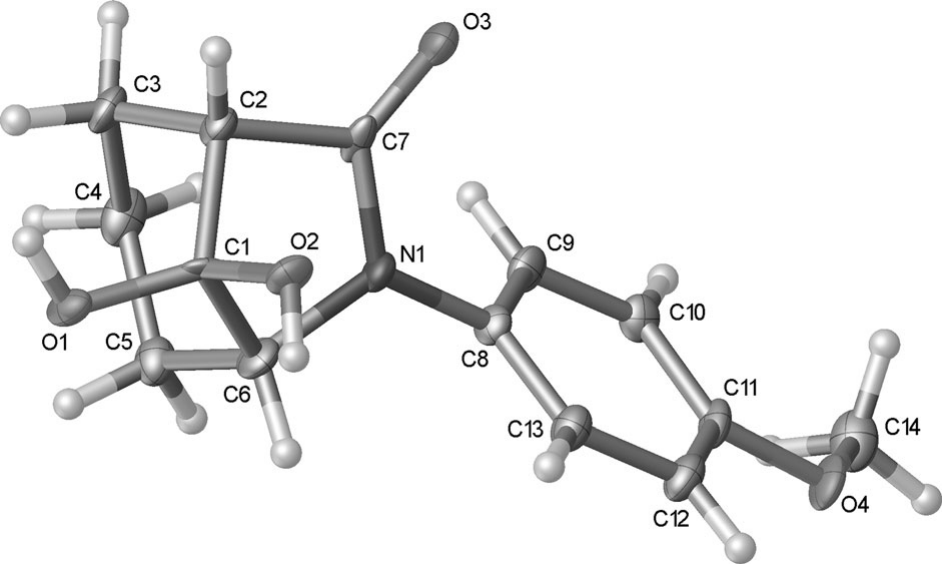
Crystal structure of hydrate 23 with ellipsoids drawn at the 50 % probability level.

Structural assignment of the semipinacol rearrangement product as ketone **21** rather than the alternative 1,2-dicarbonyl **22** arising from methine carbon migration could not be made unambiguously by NMR spectroscopic analysis. However X-ray crystallography confirmed the formation of the 6-azabicyclo[3.2.1]octane ring system. Surprisingly the corresponding hydrate **23** crystallized rather than ketone **21** from acetone solution (Figure 4). Intermolecular hydrogen bonding is evident in the solid state, which presumably stabilizes the hydrate structure.[24]

**Scheme 5:**
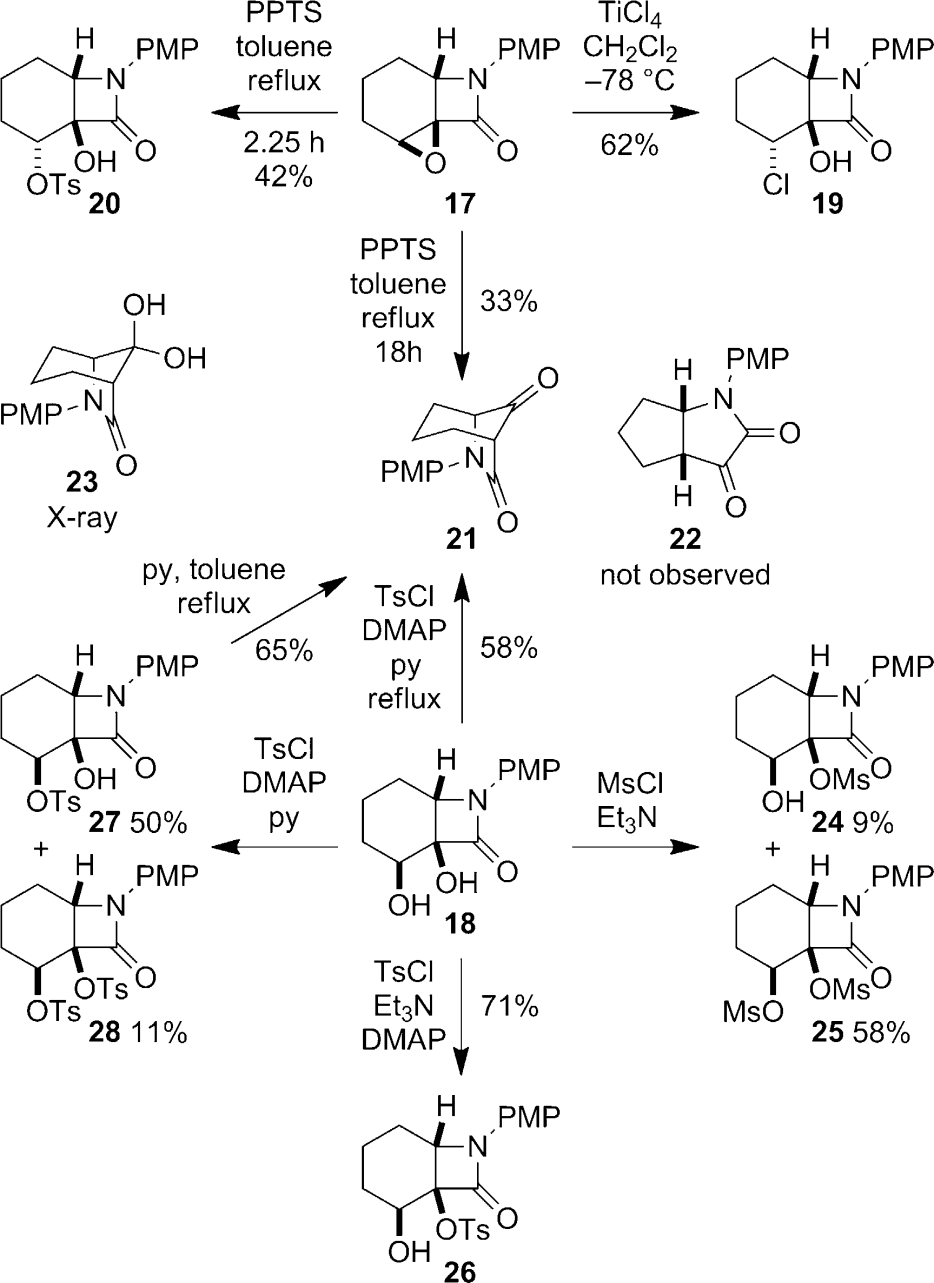
Attempted semipinacol rearrangement of epoxide 17 and diol 18. DMAP=4-dimethylaminopyridine; py=pyridine.

The more robust diol **18** offered a wider variety of potential conditions to affect the semipinacol rearrangement; however, selective activation of the secondary over the tertiary alcohol proved to be challenging. Attempted mesylation gave predominantly the dimesylate **25**, alongside **24**, the product of mesylation of the tertiary alcohol, with dimesylation favoured even in the presence of starting diol. Treatment of **18** with tosyl chloride, DMAP and triethylamine also showed the preference for reaction at the tertiary alcohol, with tosylate **26** isolated in 71 % yield. Switching to pyridine as base partially reversed this selectivity, with the desired secondary tosylate **27**, clearly structurally distinct from its epimer **20**, isolated in 50 % yield alongside ditosylate **28**. Tosylate **27** underwent the desired rearrangement to **21** in refluxing toluene in the presence of pyridine, and **21** could be prepared in 58 % yield in one step from **18** without isolation of the tosylate (Scheme 5). Attempted direct pinacol rearrangement of diol **18** using PPTS in toluene at 80 °C gave only starting material, and recourse to a stronger acid (TsOH) gave either no reaction, or degradation at higher temperatures.

The apparent higher reactivity of the tertiary over the secondary alcohol in **18** may be a consequence of the conformation adopted by the bicyclic ring system. Analogous to **2**, the cyclohexane ring of **18** is forced to adopt a boat-like conformation to accommodate the *cis*-ring fusion of the β-lactam. As a consequence the secondary alcohol is in a more hindered flagstaff position and the tertiary alcohol is pseudo-equatorial and relatively exposed. Hence reaction at the tertiary alcohol, or migration of groups from secondary to tertiary, is feasible.

**Scheme 6:**
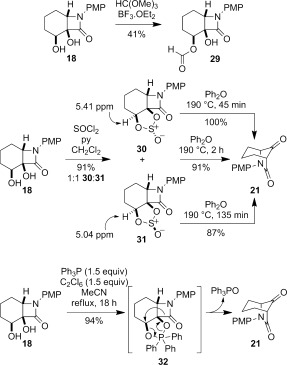
Attempted semipinacol rearrangement through cyclic activation.

The moderate yields of **21** from epoxide **17** and diol **18**, coupled with the difficulties in selectively activating the secondary alcohol of **18**, led us to investigate cyclic systems. Attempted rearrangement via a cyclic orthoester through treatment of diol **18** with trimethylorthoformate and a Lewis acid gave the formate ester **29** in 41 % yield (Scheme 6).[33] Suspecting that the difficulty might lie in forming a five-membered ring intermediate with a carbon linking the two alcohols, attention turned to the use of a heteroatom linker offering a potential driving force for rearrangement.[34] Although less precedented in the literature, we were drawn to reports on the rearrangements of cyclopropyl diols to cyclobutanones by in situ formation of cyclic sulfites or sulfates, occurring at or below room temperature.[35, 36]

**Figure 5:**
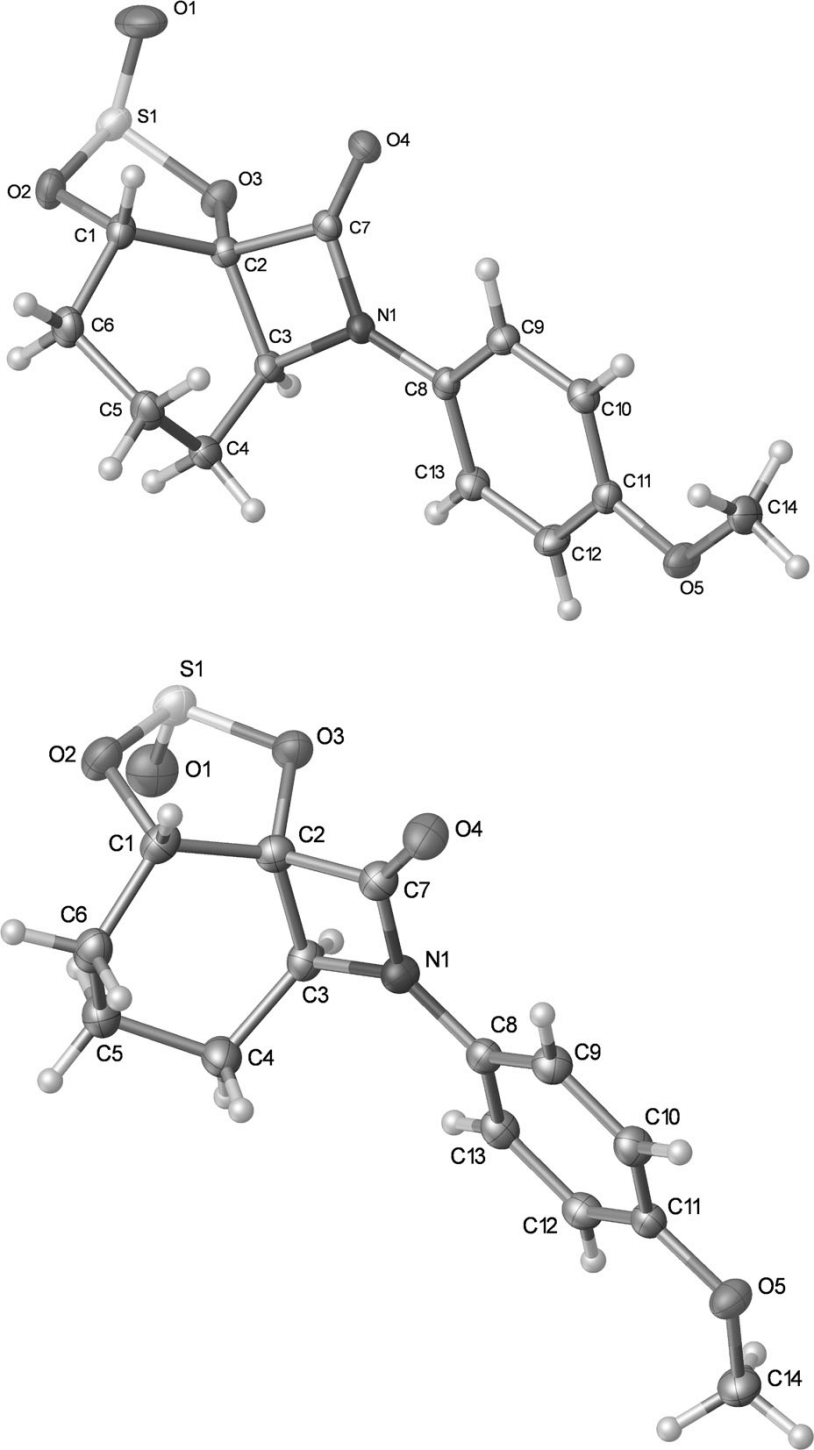
Crystal structures of 30 (top) and 31 (bottom) with ellipsoids drawn at the 50 % probability level. Selected bond lengths and torsion angles: 30: C2–C7: 1.542(2), C2–C3: 1.558(2), C1–O2: 1.4559(18) Å, O2-C1-C2-C7: 140.95(13), O2-C1-C2-C3: −117.46(14)°; 31: C2–C7: 1.5394(17), C2–C3: 1.5617(17), C1–O2 1.4670(15) Å; O2-C1-C2-C7 147.23(11), O2-C1-C2-C3 −108.61(12)°.

Treatment of diol **18** with thionyl chloride and pyridine gave a 1:1 mixture of cyclic sulfites **30** and **31** in 91 % yield (Scheme 6). The sulfites were separable by column chromatography, and the stereochemistry at the sulfinyl group initially assigned on the basis of the ^1^H NMR chemical shift of the proton in the cyclic sulfite ring, which appears at *δ*=5.41 ppm for **30** and 5.04 ppm for **31**. The anisotropy of the sulfinyl (S=O) group results in a downfield shift for the appropriately aligned proton adjacent to oxygen in **30**.[37] Subsequent X-ray crystallography of both **30** and **31** confirmed the stereochemical assignment (Figure 5).[24]

Heating a solution of cyclic sulfites **30** and **31** in diphenyl ether at 190 °C effected the desired semipinacol rearrangement to the target bridged bicyclic ketone **21**, which was isolated in excellent yield by direct column chromatography of the reaction mixture (Scheme 6). Other solvents at comparable or lower temperatures were less effective, and attempts to catalyze the process with a Lewis acid resulted in lower yields (see the Supporting Information). Qualitatively, **31** required a longer reaction (135 min) time than **30** (45 min) for the semipinacol rearrangement to go to completion, with the yield of ketone **21** also lower (87 vs. 100 %). Although the crystal structures show that the migrating C2–C7 bond in **31** is shorter than in **30** (1.5394(17) vs. 1.542(2) Å), conversely the leaving group and the migrating group is better aligned in **31** than in **30** (O2-C1-C2-C7 torsion angle 147.23(11) vs. 140.95(13)°). Reduction in the overall dipole moment might also rationalize the faster and higher yielding rearrangement of **30** compared to **31**: the S=O and C=O dipoles in **30** are more closely aligned than in **31**. However, in the absence of additional examples and knowledge of the concertedness of the rearrangement with release of SO_2_ at relatively high temperatures, these rationales should be regarded as speculative at best.

Although the rearrangement of cyclic sulfites **30** and **31** overcame the need to selectively activate the secondary alcohol of **18**, and provided **21** in much higher overall yield than previously achieved, we were keen to reduce both the temperature and the additional step required. To this end we investigated the use of cyclic phosphoranes. These can be prepared through reaction of diols with Ph_3_PCl_2_, conveniently generated in situ through reaction of Ph_3_P with a suitable chlorine source.[38] We[39] and others[40] have used in-situ-generated cyclic phosphoranes to achieve related rearrangements with 1,2-hydride migration (Meinwald-like rearrangement). In practice, treatment of diol **18** with 1.5 equivalents of Ph_3_P and C_2_Cl_6_ in refluxing acetonitrile gave the target ketone **21** in an excellent 94 % yield through the presumed rearrangement of an intermediate cyclic phosphorane **32** (Scheme 6). In contrast to the use of similar methodology for the rearrangement of diols to ketones with 1,2-hydride migration,[39, 40] the addition of a base such as *i*Pr_2_NEt to neutralize the HCl formed in the cyclic phosphorane formation is not necessary, and in fact proved detrimental to the yield of **21**. The formation of triphenylphosphine oxide as a byproduct did not prove problematic for the purification of **21** by column chromatography. This represents the first use of cyclic phosphoranes to affect semipinacol-like rearrangement with carbon–carbon bond migration.

### Methodology scope and limitations

With a successful route from fused β-lactam **14** to bridged bicyclic ketone **21** by the semipinacol rearrangement of cyclic derivatives of diol **18** in hand, attention turned to determining the scope and limitations of the methodology.

**Carbamoyl radical mediated synthesis of β-lactams**: The 4-*exo*-trig carbamoyl radical cyclization has been previously investigated to a limited extent for the synthesis of β-lactams.[41]–[45] However, at the outset of this work we were aware of only one additional example in the literature, related to the cyclization of **3** to **4**, for the synthesis of a ring-fused system.[42]

A range of carbamoyl radical precursors **33**–**39** were prepared in two steps (one purification) from the corresponding amine by using our previously described methodology: formation of a carbamoyl chloride using triphosgene and pyridine in toluene, followed by chloride displacement with sodium diethyldithiocarbamate in acetone (Table 2). Yields were generally >80 % over two steps.

**Table 2:** 4-*Exo*-trig carbamoyl radical cyclization—dithiocarbamate group transfer mediated synthesis of β-lactams.[a]

Entry	Radical precursor	β-Lactam	Yield [%][b]
1	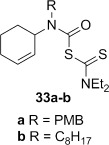	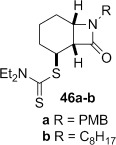	**a**: 80
2			**b**: 91
3	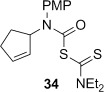	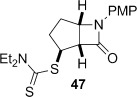	92
4[c]	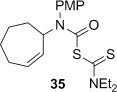	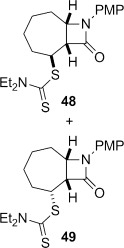	39
			29
5	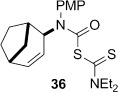	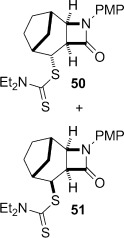	65
			16
6	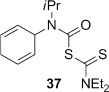	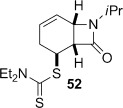	55
7	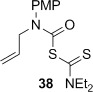	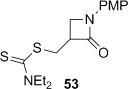	86
8	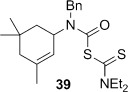	no reaction	0
9[c]		degradation	0

[a] Conditions: *hυ*, 500 W Halogen lamp, Pyrex, cyclohexane (0.1 m), reflux, 2–5 h.

[b] Isolated yield after column chromatography.

[c] Chlorobenzene as the reaction solvent.

The bridged bicyclic amine **42** was prepared from the known dibromide **40** through a sequence of allylic substitution[46] followed by reductive lithiation to remove the vinyl bromide. Attempts to employ vinyl bromide **41 a** in a palladium-mediated cyclocarbonylation to α,β-unsaturated β-lactam **43**, a reaction reported for the corresponding non-bridged system,[28] were unsuccessful.

**Scheme 7:**
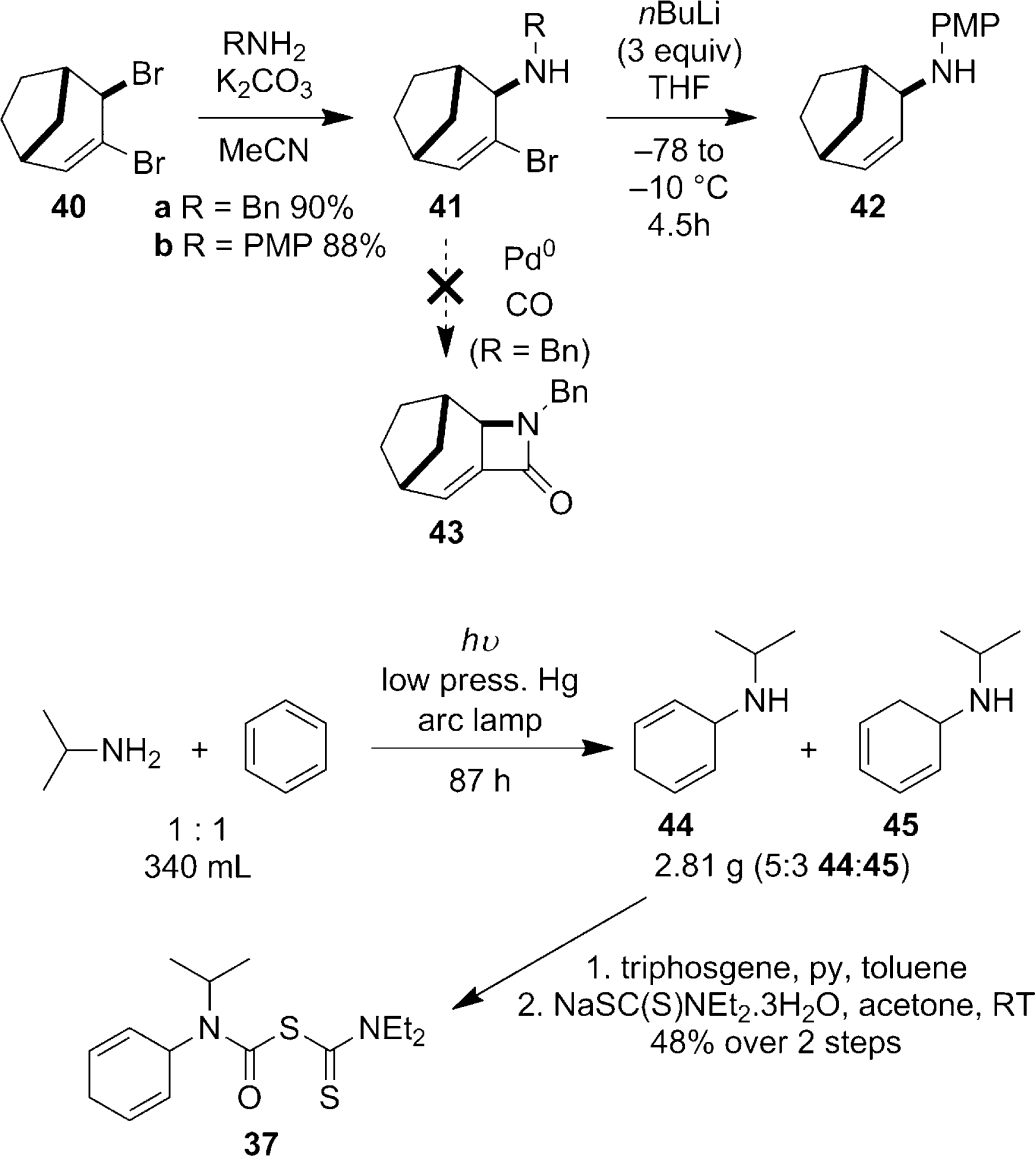
Preparation of allylic amines through reductive debromination and amine–benzene photoaddition.

The novel 1,4-cyclohexadiene **44** was prepared as an inseparable 5:3 mixture with conjugated diene **45** through photoaddition of isopropylamine to benzene.[47] Treatment of the mixture of aminodienes with triphosgene gave a mixture of carbamoyl chlorides, from which the skipped diene **37** could be isolated in 48 % yield over two steps after reaction with sodium diethyldithiocarbamate (Scheme 7).

4-*Exo*-trig carbamoyl radical cyclization of carbamoyl dithiocarbamates **33**–**38** proceeded in reasonable to excellent yield under our standard conditions (irradiation with a 500 W halogen lamp; Table 2, entries 1–7). Although the *cis*-ring junction in the β-lactam products is ensured due to the constraint of the tether in the cyclization, the stereochemistry at the dithiocarbamate stereocentre depends on the facial selectivity in the group transfer to the intermediate carbon-centred radical. The cyclization of **33 a**–**b**, **34**, and **37** gave rise to single β-lactam products (Table 2, entries 1–3 and 6). As for **14**, the stereochemistry of β-lactams **46 a** and **46 b** was assigned based on the close spectral similarity to the previously reported β-lactam **2**.[24] The stereochemistry in **47** and **52** was assigned on the basis of the expected dithiocarbamate group transfer to the less-hindered face of the bicyclic radical intermediate, but has not been unambiguously determined.

**Figure 6:**
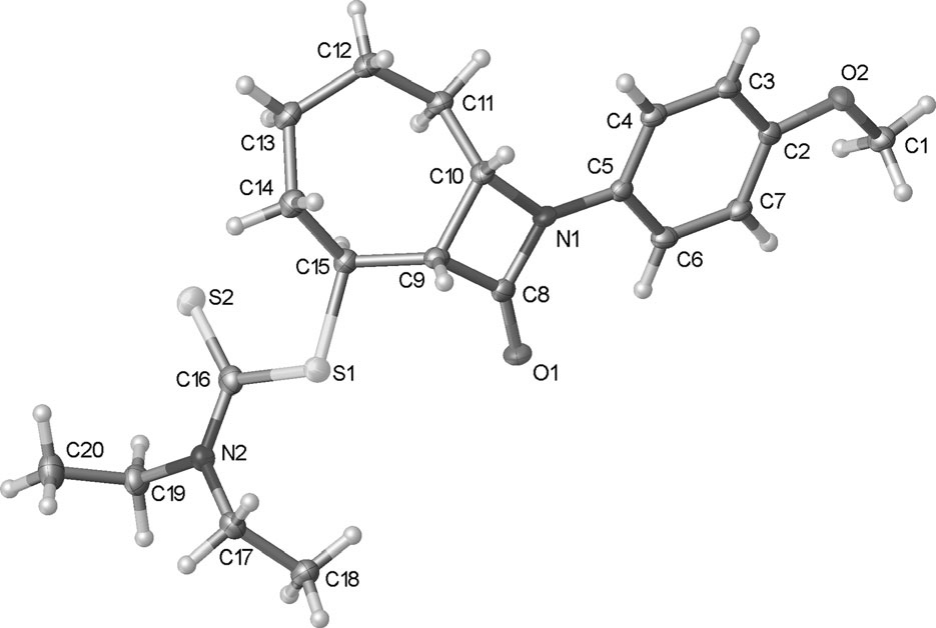
Crystal structure of 48 with ellipsoids drawn at the 50 % probability level.

The cyclization of the seven-membered carbocylic systems **35** and **36** gave rise to mixtures of diastereomers (Table 2, entries 4 and 5). The structure of the major diastereomer **48** from the cyclization of **35** was confirmed by X-ray crystallography (Figure 6).[24] The relative stereochemistry in the bridged systems **50** and **51** was confirmed by X-ray analysis of a subsequent derivative of the minor isomer **51** (vide infra). Hence the major isomer in the cyclization of both **35** and **36** results from group transfer *syn* to the hydrogen atoms at the β-lactam ring junction (as it does in the cyclization of **1**, **13**, **33**, **34**, and **37**), although the diastereoselectivity is higher for **36**, presumably due to the additional rigidity in the tricyclic ring system rendering the face *syn* to the one-carbon bridge less accessible.

**Figure 7:**
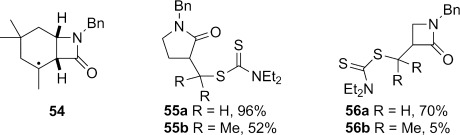
Effect of substitution on carbamoyl radical cyclization—dithiocarbamate group transfer.

Carbamoyl dithiocarbamate **39** did not provide any of the expected cyclization product under our standard conditions, with extensive degradation occurring when the higher boiling chlorobenzene was instead used in place of cyclohexane as reaction solvent (Table 2, entries 8 and 9). Cyclization of **39**, if it occurred, would generate tertiary alkyl radical **54** (Figure 7). We have previously observed lower yields in the 5-*exo*-trig carbamoyl radical cyclization onto a trisubstituted alkene to produce **55 b** compared with a terminal alkene to produce **55 a**.5b This situation is exacerbated in the case of the 4-*exo*-trig cyclization, with lactam **56 b** only isolated in 5 % yield from a complex reaction mixture, compared to **56 a** lacking the methyl groups.5a, [48] A combination of a slower cyclization[45] of the nucleophilic carbamoyl radical onto a more electron-rich double bond, and a slower group-transfer reaction,[49] may accounts for these trends, and the lack of product formation from **39**.

**Dithiocarbamate elimination**: Elimination of the dithiocarbamate group from β-lactams **46 a** and **2** bearing *N*-benzylic groups to form α,β-unsaturated β-lactams **57 a** and **57 c** occurred in excellent yield (Table 3, entries 1 and 2). The *N*-octyl lactam **46 b** required a larger excess of LHMDS and MeI to drive the reaction to completion, with α,β-unsaturated lactam **57 b** obtained in lower yield (entry 2). Thermal elimination of **46 a** gave alkene **58 a** in a similar yield to **16** and that previously reported for **58 c**.[29]

**Table 3:** Base-mediated and thermal elimination of the dithiocarbamate functional group.

Entry	β-Lactam	Base-mediated elimination[a]	Yield [%][b]	Thermal elimination[c]	Yield [%][b]
1	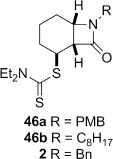	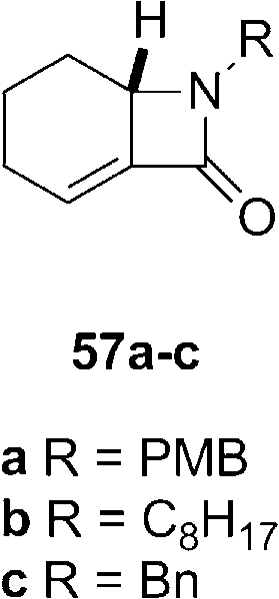	**a**: 88	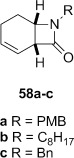	**a**: 80
2			**b**: 67		**b**: not determined
3			**c**: 93		**c**: 79[29]
4	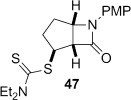	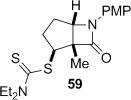	9[d]	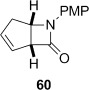	77
5	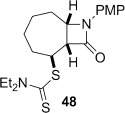	no reaction	–	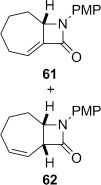	85
					10
6	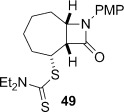	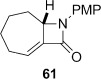	93	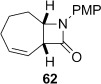	89
7	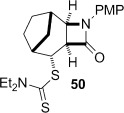	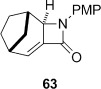	78	degradation	
8	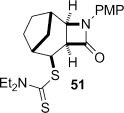	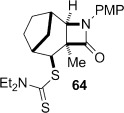	82	no reaction, recovered starting material	
9	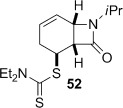	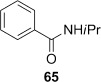	2+98 rsm[e]	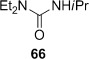	34
10			65+22 rsm[f]		
11			89+11 rsm[g]		
12	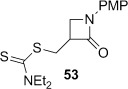	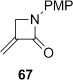	95	not determined	

[a] Conditions: MeI (1.1–5 equiv), LHMDS (1.1–5 equiv), THF, −78 °C, 5–8 h.

[b] Isolated yield after column chromatography.

[c] Conditions: Ph_2_O, reflux, 1–7 h.

[d] Conditions: MeI (5 equiv), LHMDS (5 equiv), THF, −78 °C to RT, 18 h.

[e] Conditions: MeI (10 equiv), LHMDS (10 equiv), −78 °C to RT, 18 h.

[f] Conditions: MeI (1.1 equiv), LDA (1.1 equiv), THF, −78 °C to RT, 18 h.

[g] Conditions: LHMDS (1.5 equiv), Davis oxaziridine (1.5 equiv), 0 °C to RT, 18 h.

Attempted base-mediated elimination of the cyclopentyldithiocarbamate **47** was unsuccessful, with only starting material returned. The use of five equivalents each of LHMDS and MeI gave mainly starting material alongside small quantities of the C-methylated β-lactam **59**, which suggests that deprotonation occurs but presumably the α,β-unsaturated β-lactam is too strained to form. Thermal elimination of **47** gave the expected alkene **60** in good yield (Table 3, entry 4).

The epimeric dithiocarbamates **48** and **49** showed divergent behaviour towards thermal and basic elimination conditions (Table 3, entries 5 and 6). Whereas **48** proved surprisingly resistant to base-mediated elimination, thermal elimination gave predominantly the conjugated alkene **61**, presumably the larger ring size better accommodating the double bond at the ring junction. In contrast, thermal elimination of **49** gave non-conjugated alkene **62**, consistent with the concerted Chugaev-like mechanism of the process.[29] Previous studies in our group have shown that, despite the high temperatures required for thermal elimination of the dithiocarbamate group, equilibration between alkene regioisomers does not occur under the reaction conditions. Product outcome is determined by the availability of a suitable β-H *syn* to the dithiocarbamate group, hence only **62** forms from thermolysis of **49**. Base-mediated elimination was successful in the case of **49**, providing the target alkene **61** in an excellent 93 % yield. Hence both **48** and **49** could be converged to the desired alkene **61** under appropriate conditions.

**Figure 8:**
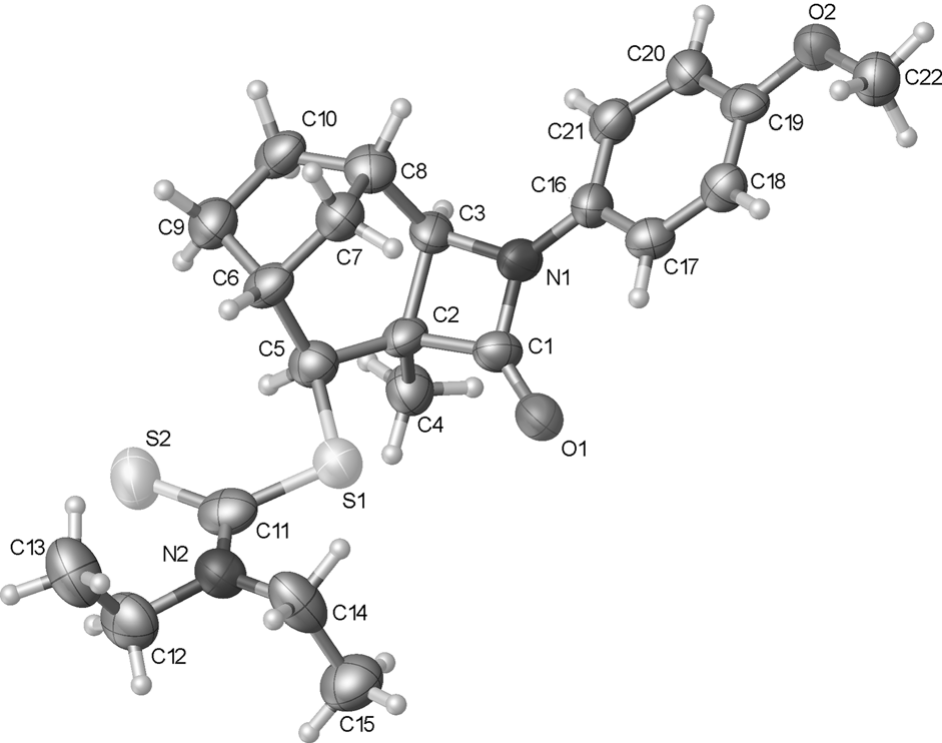
Crystal structure of 64 with ellipsoids drawn at the 50 % probability level.

Tricyclic dithiocarbamate **50** was successfully converted to alkene **63** under basic conditions, but degraded upon attempted thermolysis (Table 3, entry 7). The minor epimer **51** could not be utilized—it underwent clean C-methylation to provide **64** in high yield under basic conditions, and unsurprisingly could not be eliminated under thermal conditions given that this would generate an *anti*-Bredt alkene (entry 8). The structure of **64** was proven by X-ray crystallography (Figure 8),[24] which, given that the deprotonation and resulting methylation of the β-lactam does not affect the dithiocarbamate stereocentre, also confirmed that the major diastereoisomer formed in the cyclization of **36** was **50** (Table 2, entry 5).

**Scheme 8:**
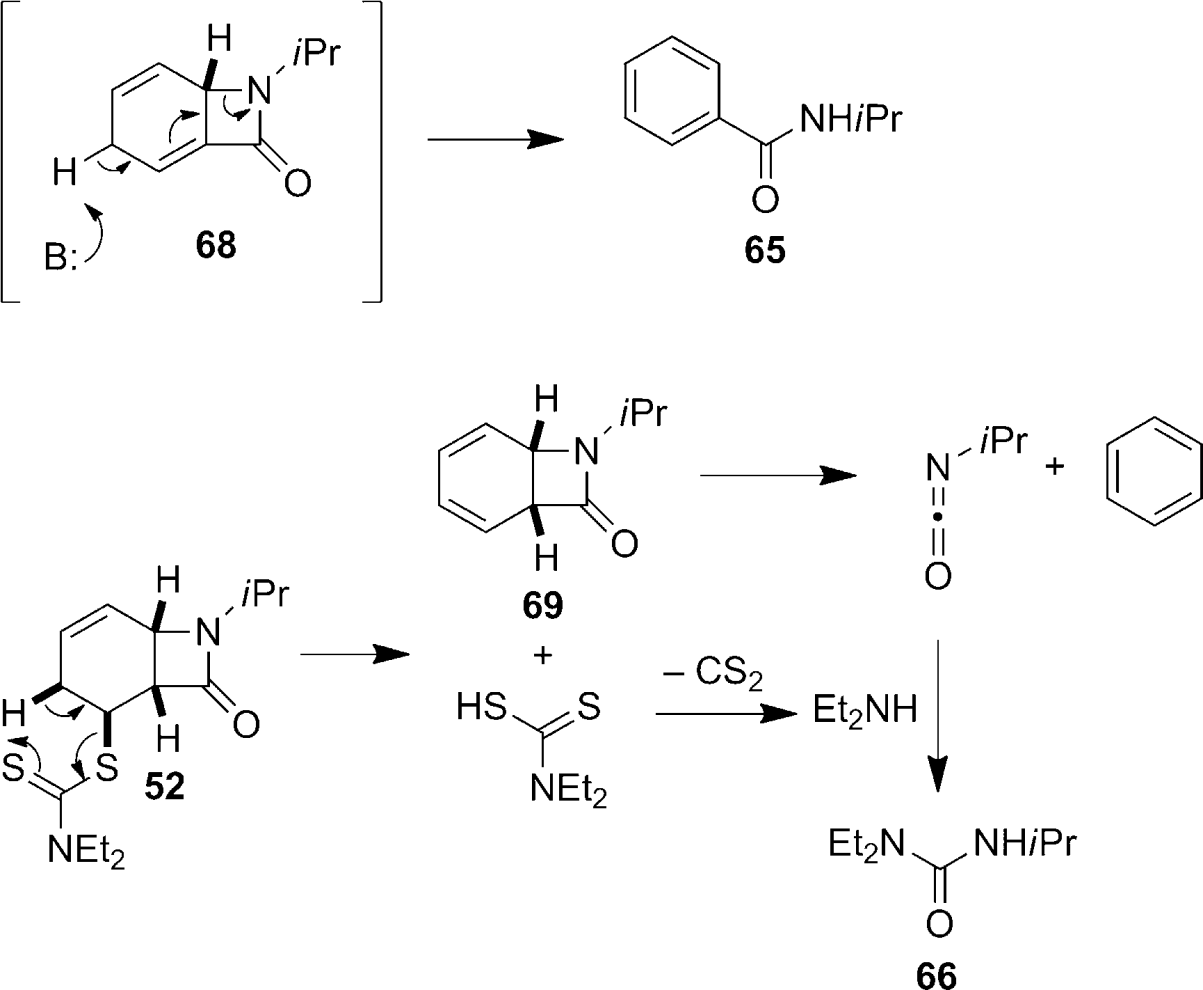
Base-mediated and thermal elimination of dithiocarbamate 52.

Attempted base-mediated elimination of **52** gave mainly starting material and small amounts of *N*-isopropylbenzamide (**65**) (Table 3, entry 9). The yield of **65** increased slightly with a change of base to LDA (entry 10), and dramatically when MeI was replaced with the Davis oxaziridine in an attempt to trap out the putative deprotonated β-lactam with an oxygen source (entry 11). Although the role of the oxaziridine in this process is not known,[50] the formation of **65** in all cases is consistent with a presumably facile base-mediated fragmentation of the target diene **68**, generated in situ (Scheme 8).

Thermolysis of **52** also generated a surprising result. In this case the urea **66** was isolated from the reaction mixture in 34 % yield (Table 3, entry 9). The formation of **66** can be rationalized according to the sequence shown in Scheme 8. At the high temperatures involved, conjugated diene **69**, generated in situ, undergoes pyrolytic ring fission to benzene and isopropylisocyanate.[51] The dithiocarbamic acid byproduct of the dithiocarbamate group elimination fragments to diethylamine and carbon disulfide,[29] and whereas normally these are lost at high temperature, the amine reacts with the isocyanate to form urea **66**.

Base-mediated elimination of the dithiocarbamate group in the simple monocyclic β-lactam **53** gave alkene **67** in excellent yield (Table 3, entry 12). We have previously found the analogous monocyclic *N*-Bn β-lactam degrades under thermal elimination conditions,[29] and hence overall this route provides efficient access to *exo*-methylene β-lactams in combination with our high-yielding radical cyclization methodology. Alkene **67** has previously been synthesized in a palladium-catalyzed carbonylation of a 2-bromoallylamine, albeit in low yield.[28]

**Scheme 9:**
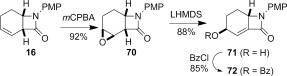
Functionalization of alkene 16.

*m*CPBA-mediated epoxidation of alkene **16** provided a means to introduce additional functionality on the cyclohexanone ring of bicyclic lactam **21** (Scheme 9). The stereochemistry of epoxide **70** was assigned based on the expected preferential attack on the convex face of the bicyclic ring system. Regioselective base-mediated ring-opening of epoxide **70** gave α,β-unsaturated β-lactam **71**. The liberated alcohol was protected as the benzoate ester **72**.

**Dihydroxylation and semipinacol rearrangement**: The dihydroxylation of α,β-unsaturated β-lactams **57 a**–**c**, **72**, **61** and **67** gave diols **73 a**–**c**, **74**, **75** and **78**, respectively, in reasonable to good yield (Table 4, yields of diols in parentheses). Treatment of alkene **63** under the same conditions gave the expected diol **76** as the major product as an inseparable mixture along with the α-hydroxyketone **77**. Attempts to minimize the formation of the unwanted byproduct **77** were unsuccessful.[52] In all cases dihydroxylation is completely diastereoselective, re-establishing the *cis*-ring fusion of the β-lactam.

**Table 4:** Dihydroxylation and semipinacol rearrangement of β-lactams.

Entry	Diol (% yield from alkene dihydroxylation)[a]	Method[b]	Product(s)	Yield [%]
1	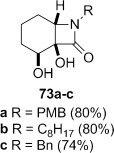	A	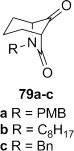	**79 a**: 77
2				**79 b**: 77
3				**79 c**: 83
4		B		**79 a**: 80
5				**79 b**: 80
6				**79 c**: 97
7	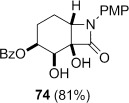	A	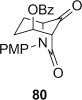	75
8		B		77
9	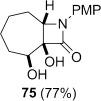	A	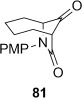	82
10		B		98
11	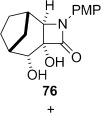	A[c]	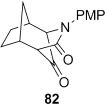	64[d]
12	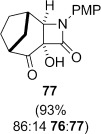	B	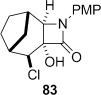	28[e]
13	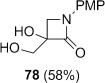	A	no reaction[f]	–
14		B	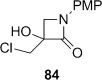	85

[a] Dihydroxylation conditions: cat. OsO_4_, NMO (2.4 equiv), 5:5:2 acetone/H_2_O/*t*BuOH, 40 °C, 18 h.

[b] Method A: i) SOCl_2_, pyridine, 0 °C to RT; ii) Ph_2_O, 190 °C, 2–5 h. Method B: PPh_3_, C_2_Cl_6_, CH_3_CN, reflux, 18 h.

[c] Reaction conditions: ii) Ph_2_O, reflux, 2 h.

[d] Yield over three steps from alkene **63**.

[e] Yield over two steps from alkene **63**.

[f] No reaction at 190 °C in Ph_2_O. Decomposition in refluxing Ph_2_O.

Semipinacol rearrangement of diols **73 a**–**c**, **74**, **75** and **78** was attempted via both the corresponding cyclic sulfites in two steps (Table 4, method A), and in one step via the cyclic phosphorane (method B). As for **30** and **31**, cyclic sulfites were obtained as approximately 1:1 mixtures of diastereomers. For comparison purposes yields in Table 4 for method A are over two steps, formation of the cyclic sulfite and subsequent thermolysis of the mixture. In general yields are comparable or slightly better using method B, but in some cases this method fails, despite the milder conditions.

β-Lactams fused to six-membered rings rearranged under both conditions (Table 4, entries 1–8). Notably the epoxide stereochemistry, established in **70**, is translated into the axially-orientated benzoate in **80**. The lack of a large axial–axial coupling for the proton adjacent to oxygen in **80** is consistent with the axial orientation, and confirms the expected stereoselectivity of the epoxidation step (Scheme 9).

Rearrangement of the seven-membered ring-fused β-lactam diol **75** gave the keto-bridged bicyclic lactam **81**, again with complete selectivity for *N*-acyl group migration, despite the larger ring size (Table 4, entries 9 and 10). The 7-azabicyclo[4.2.1]nonane ring system **81** is found in members of the *Gelsemium* alkaloids, which have been the subject of some synthetic interest.[53]

Treatment of the mixture of diol **76** and ketoalcohol **77** with thionyl chloride and pyridine allowed for the separation of **77** from the cyclic sulfites derived from **76**. Rearrangement of the cyclic sulfites did not occur at 190 °C, but in refluxing diphenyl ether (b.p. 259 °C) conversion to the doubly bridged ring system **82** occurred (Table 4, entry 11). In contrast, direct subjection of the mixture of **76** and **77** to Ph_3_P and C_2_Cl_6_ in refluxing MeCN (method B) did not give any of the semipinacol rearrangement product **82**. Instead, chloroalcohol **83** was isolated in low yield. The conversion of alcohols to chlorides by using Ph_3_P and electrophilic chlorine sources (Appel conditions) is known in the literature.[54] The stereochemistry in **83** was assigned on the basis of the expected S_N_2 displacement by chloride, and also suggested by the lack of coupling between the proton adjacent to chlorine with the adjacent bridgehead proton. Molecular models showed that the dihedral angle between these protons is close to 90° when the chlorine is *syn* to the one carbon bridge, as in **83**. The lack of rearrangement of **76** under these conditions may suggest that the cyclic phosphorane does not form, although we do not have any evidence for this. The higher temperature required to rearrange the corresponding sulfite suggests that the barrier to rearrangement is higher for the diol **76** compared to diol **75**, which lacks the constraint imposed by the additional one-carbon bridge, allowing other reaction pathways to compete. Raising the temperature of the Ph_3_P/C_2_Cl_6_ reaction by running the reaction in a microwave up to 150 °C over 3 h still only provided **83** in low yield, with no evidence of formation of **82**.

Semipinacol rearrangement of the monocyclic β-lactam diol **78** could not be achieved under either set of conditions. Although the cyclic sulfite could be prepared in 68 % yield, no rearrangement occurred upon heating at 190 °C, and the reaction mixture underwent decomposition in refluxing Ph_2_O. High-yielding conversion to the chloroalcohol **84** occurred upon treatment of **78** with Ph_3_P and C_2_Cl_6_ in refluxing acetonitrile. Clearly the competing S_N_2 substitution pathway is particularly favourable at the primary alcohol of **78**. More generally, the preference for ring-fused systems to undergo semipinacol rearrangement rather than Appel reactions can, therefore, be ascribed to a combination of factors: the slower S_N_2 reaction at a secondary rather than a primary alcohol, the enforcement of a favourable orbital alignment for bond migration, and increased ring strain offering a greater driving force for rearrangement.

**Scheme 10:**
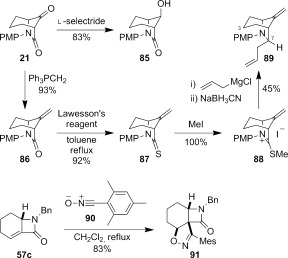
Reactions of lactams 21 and 57 c.

**Functional-group transformations**: The 7,8-dioxo-6-azabicyclo[3.2.1]octane ring system is a potentially versatile intermediate for organic synthesis. Preliminary studies have shown that the two carbonyl groups in **21** can be chemo- and stereoselectively functionalized (Scheme 10). Treatment with l-selectride gave the axial alcohol **85** stereoselectively. Wittig methylenation gave terminal alkene **86** in excellent yield. Carbon–carbon bond formation at the amide carbonyl proceeded through formation of thioamide **87**, activation as the methyl sulfonium salt **88**, and subsequent treatment with allyl Grignard followed by sodium cyanoborohydride.16a The resulting diene **89** was isolated as a single diastereomer, presumed to be the result of reduction from the less-hindered *exo*-face of the intermediate imminium. Tentative assignment of the C-7 stereocentre was also based on the absence of an nOe signal between the axial hydrogen at C-3 and the newly installed hydrogen at C-7.

In the course of investigating potential activation pathways for the transformation of α,β-unsaturated β-lactam **57 c** into bridged bicyclic amide **79 c**, we also investigated a 1,3-dipolar cycloaddition reaction with the isolable nitrile oxide **90**.[55] The double bond in **57 c** was shown to be a competent dipolarophile, providing 2-isoxazoline **91** as a single stereoisomer in good yield (83 %). Notably, the regioselectivity is opposite to that reported for the 1,3-dipolar cycloaddition of a nitrile oxide with a monocyclic *exo*-methylene β-lactam.[56]

## Conclusions

The 4-*exo*-trig carbamoyl radical cyclization—dithiocarbamate group transfer reaction has been shown to be an efficient and practical methodology for the synthesis of ring-fused β-lactams. Good yields of β-lactams are achieved with the exception of a system carrying double substitution at the alkene terminus. Novel conditions for the base-mediated elimination of the dithiocarbamate group have been developed, which provide access to alkene regioisomers unavailable through thermolysis. Dihydroxylation of α,β-unsaturated β-lactams provides substrates which undergo semipinanol rearrangement with exclusive *N*-acyl group migration under all conditions. This selectivity is consistent with prior examples of semipinacol rearrangement of non-fused β-lactams in the literature and the expected better alignment of the migrating bond with the breaking C–O bond, even when constrained within a heterocyclic ring system. In situ generated cyclic phosphoranes have been shown to undergo semipinacol rearrangement with C–C bond migration for the first time, and provide milder and shorter routes to target compounds over the use of non-cyclic systems and of cyclic sulfites, unless chloroalcohol formation competes. The resulting keto-bridged bicyclic lactams are versatile intermediates in target synthesis.
