# Supplementary material for: Carbamoyl Radical-Mediated Synthesis and Semipinacol Rearrangement of β-Lactam Diols
Source: Chemistry. 2014 Apr 7;20(21):6505–17. doi: 10.1002/chem.201304982 (PMC4320754; doi:10.1002/chem.201304982)

CHEMISTRY
A European Journal

Supporting Information

© Copyright Wiley-VCH Verlag GmbH & Co. KGaA, 69451 Weinheim, 2014

Carbamoyl Radical-Mediated Synthesis and Semipinacol Rearrangement of β-Lactam Diols

Marie Betou,[a] Louise Male,[a] Jonathan W. Steed,[b] and Richard S. Grainger*[a]

chem_201304982_sm_miscellaneous_information.pdf

Carbamoyl radical-mediated synthesis and semipinacol rearrangement of β–lactam diols

Marie Betou,[a] Louise Male,[a] Jonathan W. Steed[b] and Richard S. Grainger*[a]

[a] Dr R. S. Grainger, Dr M. Betou, Dr L. Male
School of Chemistry
University of Birmingham
Edgbaston, Birmingham B15 2TT, UK
Fax: (+44) 121 4144403
E-mail: r.s.grainger@bham.ac.uk

[b] Prof. Jonathan W. Steed
Department of Chemistry
Durham University
South Road, Durham DH1 3LE, UK

Supporting Information

Supporting information for this article is available on the WWW under http://www.chemeurj.org/ or from the author.

Contents

S2 General experimental

S3-S33 Experimental procedures and optimization studies

S34 References

S35-S158 Copies of ^{1}H and ^{13}C NMR spectra

General experimental

^{1}H and ^{13}C NMR spectra were recorded on a Bruker AVIII 300, Bruker AVIII 400 spectrometer. Spectra were recorded in deuterochloroform referenced to residual $CHCl_3$ (^{1}H, 7.26 ppm; ^{13}C, 77.2 ppm). Chemical shifts (δ) are reported in ppm and coupling constants (J) are reported in Hz. The following abbreviations are used to describe multiplicity; s-singlet, d-doublet, t-triplet, q-quartet, hept-heptet, m-multiplet, br-broad. Mass spectra were recorded on a LCT spectrometer or on a LTQ Orbitrap spectrometer (NMSSC Swansea) utilising electrospray ionisation (recorded in the positive mode) with a methanol mobile phase, or electron impact ionisation, or chemical ionisation and are reported as (*m/z* (%)). HRMS were recorded on a LCT spectrometer or on a LTQ Orbitrap spectrometer (NMSSC Swansea) using lock mass incorporated into the mobile phase. IR spectra were recorded neat on Perkin Elmer 100-series FT-IR spectrometer. Melting points were determined using open glass capillaries on a Gallenkamp melting point apparatus and are uncorrected. Analytical t.l.c. was carried out on Merck 60 F245 aluminium-backed silica gel plates. Short-wave UV radiation (245 nm), $KMnO_4$ and vanillin were used to visualize components. Compounds were purified by flash column chromatography using Merck silica gel 60 (0.040-0.063 nm). The following cooling baths were used; 0 °C (ice/water) and -78 °C (dry ice/acetone). All reactions in non-aqueous solvents were carried out under argon in oven-dried glassware. Solvents were degassed by bubbling argon through a needle immersed in the solvent for the stated length of time.

Solvents and reagents were purified as follows:
Pyridine and triethylamine were distilled from potassium hydroxide. NBS was recrystallized from H_2O, PPh_3 was recrystallized from conc. HCl and H_2O. Piperitone, $Ti(OEt)_4$ and $Ti(O\textit{i}Pr)_4$ were purified by bulb-to-bulb distillation under reduced pressure. THF, Et_2O, toluene, CH_3OH, CH_2Cl_2 and CH_3CN were dried by passing through activated alumina columns.
*n*BuLi was purchased as a 1.6 M solution in hexanes and the solution was titrated with menthol in the presence of 1-(biphenyl-4-yl)-3-phenyl-2-azapropene (“BLUE”).
*m*CPBA was purified by washing with a pH 7 phosphate buffer: A buffer solution was prepared from 0.1 M NaOH (154 mL) and 0.2 M KH_2PO_4 (94 mL) and made up to 376 mL with distilled water. *m*CPBA (77% w/w, 10 g) was dissolved in diethyl ether (100 mL) and washed four times with the buffer solution. The organic extract was dried over $MgSO_4$ and carefully evaporated under reduced pressure to yield pure *m*CPBA (7.3 g).

All other reagents and solvents were purchased from Aldrich, Alfa Aesar, Fisher Scientific, Merck or TCI Europe and were used as received. LHMDS as a solution in THF was purchased only from Aldrich.

The preparation of compounds **1**,[1] **2**,[1] **12-15**,[2] **18**,[2] **21**,[2] **30-31**,[2] **33a-b**,[2] **35**,[2] **46a-b**,[2] **48-49**,[2] **57a-c**,[2] **58c**,[3] **61**,[2] **73a-c**,[2] **75**,[2] **79a-c**,[2] **81**[2] has been previously reported. Dibromide **40**[4a] and mesityl nitrile oxide **90**[4a] were prepared according to the literature procedures.

(±)-(1*R*,6*S*)-7-(4-Methoxyphenyl)-7-azabicyclo[4.2.0]oct-2-en-8-one **16**

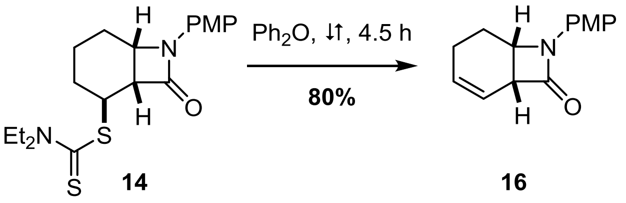

A known compound[2] prepared according to a modified literature procedure.[3] A solution of lactam **14** (1.22 g, 3.23 mmol) in diphenyl ether (25 mL) was heated to reflux. After 4.5 h, the reaction mixture was directly purified by column chromatography (Hexane to Hexane:EtOAc 7:3) to give lactam **16** (0.593 g, 80%) as a brown solid. mp 72-75 °C; R_f 0.47 (Pet ether:EtOAc 3:1); ν_{max} (neat)/cm^{-1} : 2943, 1722, 1509, 1243, 1119, 1030, 842, 804; δ_H (400 MHz, CDCl$_3$) 1.48-1.57 (1H, m, CH$_2$), 1.93-2.10 (2H, m, CH$_2$), 2.28-2.32 (1H, m, CH$_2$), 3.78-3.83 (4H, m, CHC*H*C(O) and OCH$_3$), 4.42-4.44 (1H, m, CH$_2$C*H*N), 5.87-5.91 (1H, m, CH$_2$C*H*=CH), 6.04-6.09 (1H, m, CHC*H*=CH), 6.87 (2H, d, *J* 9.0 Hz, Ar H), 7.33 (2H, d, *J* 9.0 Hz, Ar H); δ_C (100 MHz, CDCl$_3$) 19.5 (CH$_2$), 22.8 (CH$_2$), 48.2 (CH, =CH*C*HC(O)), 51.3 (CH, CH$_2$*C*HN), 55.6 (CH$_3$, OCH$_3$), 114.6 (2 x CH, Ar), 118.8 (2 x CH, Ar), 121.3 (CH, CH$_2$*C*H=CH), 130.8 (C, ArCN), 131.5 (CH, CH=*C*HCH), 156.2 (C, ArCO), 164.4 (C, C(O)N); m/z (ES) 252.0989 ([M+Na]$^+$ $C_{14}H_{15}NNaO_2$ requires 252.1000), 284.2 (5%), 252 (100).

(±)-(1*R*,3*S*,7*S*)-8-(4-Methoxyphenyl)-2-oxa-8-azatricyclo[5.2.0.0^{1,3}]nonan-9-one **17**

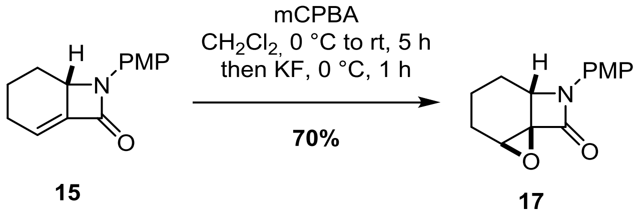

A novel compound prepared according to a modified literature procedure. [5] A solution of lactam **15** (0.300 g, 1.31 mmol) in CH$_2$Cl$_2$ (9 mL) was cooled to 0 °C, treated with recrystallised *m*CPBA (0.677 g, 3.93 mmol) and allowed to warm to rt over 1 h. After 4 h at rt, the reaction mixture was cooled to 0 °C, diluted with CH$_2$Cl$_2$ (9 mL) and treated with KF (0.686 g, 11.8 mmol). After stirring at 0 °C for 1 h, the reaction mixture was filtered through a pad of celite (eluent CH$_2$Cl$_2$), the filtrate was evaporated under reduced pressure and purified by column chromatography (Pet ether:EtOAc 9:1 to Pet ether:EtOAc 4:1) to give epoxy lactam **17** (0.224 g, 70%) as a white solid. mp 98-100 °C; R_f 0.43 (Pet ether:EtOAc 3:1); ν_{max} (neat)/cm^{-1} : 2923, 1740, 1514, 1242, 825; δ_H (300 MHz, CDCl$_3$) 1.21-1.30 (2H, m, CH$_2$), 1.64-1.72 (1H, m, CH$_2$), 1.76-1.88 (1H, m, CH$_2$), 2.19-2.26 (1H, m, CH$_2$), 2.32-2.39 (1H, m, CH$_2$), 3.68 (1H, d, *J* 2.4 Hz, CH$_2$C*H*O), 3.79 (3H, s, OCH$_3$), 4.11-4.16 (1H, m, CH$_2$C*H*N), 6.88 (2H, d, *J* 9.2 Hz, Ar H), 7.37 (2H, d, *J* 9.2 Hz, Ar H); δ_C (100 MHz, CDCl$_3$) 16.8 (CH$_2$), 25.1 (CH$_2$), 27.2 (CH$_2$), 54.0 (CH, CH$_2$CHO), 55.6 (CH$_3$, OCH$_3$), 56.4 (CH, CH$_2$*C*HN), 66.9 (C, CH*C*OC(O)), 114.7 (2 x CH, Ar), 118.3 (2 x CH, Ar), 131.6 (C, ArCN), 156.4 (C, ArCO), 164.2 (C, C(O)N); m/z (EI) 245.1048 (M$^+$ $C_{14}H_{15}NO_3$ requires 245.1052), 245 (100%), 217 (6), 189 (49), 174 (10), 149 (27), 134 (28).

(±)-(1*S*,2*R*,6*S*)-2-Chloro-1-hydroxy-7-(4-methoxyphenyl)-7-azabicyclo[4.2.0]octan-8-one **19**

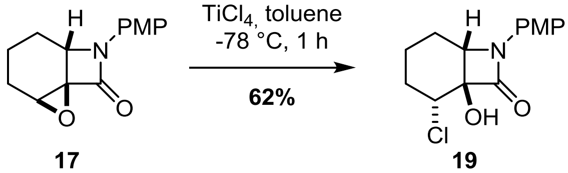

17 **19**

A novel compound prepared according to a modified literature procedure.[6] A solution of epoxy lactam **17** (30 mg, 0.12 mmol) in toluene (9 mL) was cooled to -78 °C and treated with $TiCl_4$ (14 µL, 0.13 mmol). After 1 h, the solution was carefully quenched with H_2O (4 mL) and extracted with EtOAc (2 x 5 mL). The combined organic extracts were washed with brine (10 mL), dried over $MgSO_4$, filtered and evaporated under reduced pressure to give chloro hydroxy lactam **19** (21 mg, 62%) as a yellow solid. mp 108-111 °C; R_f 0.24 (Pet ether:EtOAc 7:3); ν_{max} (neat)/cm^{-1} : 3350, 2946, 1709, 1511, 1245, 832; δ_H (400 MHz, $CDCl_3$) 1.28 (1H, br s, OH), 1.55-1.66 (1H, m, CH_2), 1.68-1.79 (1H, m, CH_2), 1.83-2.02 (2H, m, CH_2), 2.12-2.19 (1H, m, CH_2), 2.23-2.32 (1H, m, CH_2), 3.78 (3H, s, OCH_3), 4.28 (1H, dd, *J* 5.2 and 9.6 Hz, CH_2C*H*Cl), 4.30-4.32 (1H, m, CH_2C*H*N), 6.87 (2H, d, *J* 9.0 Hz, Ar H), 7.31 (2H, d, *J* 9.0 Hz, Ar H); δ_C (100 MHz, $CDCl_3$) 16.4 (CH_2), 20.3 (CH_2), 28.0 (CH_2), 55.6 (CH_3, OCH_3), 60.3 (CH, CH_2*C*HN), 62.0 (CH, CH_2*C*HCl), 84.5 (C, CH*C*OH), 114.7 (2 x CH, Ar), 119.0 (2 x CH, Ar), 130.1 (C, ArCN), 156.8 (C, ArCO), 163.8 (C, C(O)N); m/z (ES) 304.0702 ($[M+Na]^+$ $C_{14}H_{16}{}^{35}ClNNaO_3$ requires 304.0716), 306 (24%), 304 (100).

(±)-(1*R*,2*R*,6*S*)-1-Hydroxy-7-(4-methoxyphenyl)-8-oxo-7-azabicyclo[4.2.0]octan-2-yl 4-methyl benzene sulfonate **20**

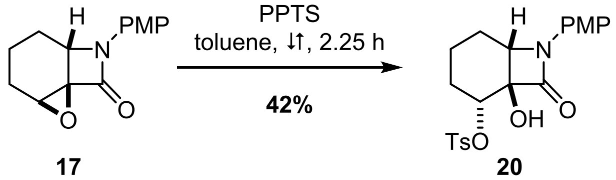

17 **20**

A novel compound prepared according to a modified literature procedure.[6] A solution of epoxy lactam **17** (0.163 g, 0.665 mmol) in toluene (4 mL) was treated with PPTS (0.184 g, 0.733 mmol) and refluxed for 2.25 h. The reaction mixture was quenched with H_2O (4 mL) and extracted with EtOAc (2 x 4 mL). The combined organic extracts were washed with brine (5 mL), dried over $MgSO_4$, filtered, evaporated under reduced pressure and purified by column chromatography (Pet ether:EtOAc 4:1 to Pet ether:EtOAc 7:3) to give *trans* hydroxy tosyl lactam **20** (0.116 g, 42%) as a white solid. mp 121-123 °C; R_f 0.34 (Pet ether:EtOAc 7:3); ν_{max} (neat)/cm^{-1} : 3433, 2949, 1730, 1512, 1172, 816; δ_H (400 MHz, $CDCl_3$) 1.59-1.72 (2H, m, CH_2), 1.77-1.92 (2H, m, CH_2), 2.02-2.11 (2H, m, CH_2), 2.44 (3H, s, $PhCH_3$), 3.78 (3H, s, OCH_3), 4.22 (1H, br s, OH), 4.24 (1H, t, *J* 3.0 Hz, CH_2C*H*N), 4.98 (1H, dd, *J* 5.8 and 11.8 Hz, CH_2C*H*OTs), 6.86 (2H, d, *J* 9.0 Hz, Ar H), 7.22 (2H, d, *J* 9.0 Hz, Ar H), 7.34 (2H, d, *J* 8.2 Hz, Ar H), 7.88 (2H, d, *J* 8.2 Hz, Ar H); δ_C (100 MHz, $CDCl_3$) 16.0 (CH_2), 20.7 (CH_2), 21.8 (CH_3, $PhCH_3$), 24.7 (CH_2), 55.6 (CH_3, OCH_3), 61.8 (CH, CH_2*C*HN), 80.9 (CH, CH_2*C*HOTs), 83.5 (C, CH*C*OH), 114.7 (2 x CH, Ar), 119.0 (2 x CH, Ar), 128.1 (2 x CH, Ar), 130.0 (2 x CH, Ar), 133.4 (C, ArCN), 145.3 (2 x C, Ar), 156.8 (C, ArCO), 163.1 (C, C(O)N); m/z (ES) 440.1147 ($[M+Na]^+$ $C_{21}H_{23}NNaO_6S$ requires 440.1144), 456 (18%), 440 (100), 423 (19), 268 (21).

(±)-(1*R*,2*S*,6*S*)-7-(4-methoxyphenyl)-8-oxo-7-azabicyclo[4.2.0]octan-1,2-diyl dimethanesulfonate **24** and (±)-(1*R*,2*S*,6*S*)-2-Hydroxy-7-(4-methoxyphenyl)-8-oxo-7-azabicyclo[4.2.0]octan-1-yl methane sulfonate **25**

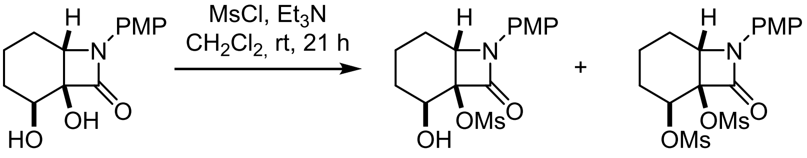

18 **24** 9% **25** 58%

Novel compounds prepared according to a modified literature procedure. [7] A solution of dihydroxy lactam **18** (0.100 g, 0.380 mmol) in CH_2Cl_2 (1 mL) was treated with Et_3N (0.11 mL, 0.80 mmol) and MsCl (31 µL, 0.40 mmol). After 18 h at rt, further Et_3N (0.11 mL, 0.80 mmol) and MsCl (31 µL, 0.40 mmol) were added to drive the reaction to completion. After 3 h, the reaction mixture was quenched with HCl (2 mL of a 1 M aq. solution) and extracted with CH_2Cl_2 (2 x 3 mL). The combined organic extracts were washed with brine (4 mL), dried over $MgSO_4$, filtered, evaporated under reduced pressure and purified by column chromatography (Pet ether:EtOAc 7:3 to Pet ether:EtOAc 3:7) to give *cis* hydroxyl mesyl lactam **24** (11 mg, 9%) as a colourless oil and dimesyl lactam **25** (93 mg, 58%) as a white solid.

24: R_f 0.22 (Pet ether:EtOAc 1:1); ν_{max} (neat)/cm^{-1} : 3485, 2937, 1741, 1511, 1357, 1245, 830; δ_H (400 MHz, $CDCl_3$) 1.34-1.45 (1H, m, CH_2), 1.68-1.76 (1H, m, CH_2), 1.79-1.88 (1H, m, CH_2), 1.98-2.13 (2H, m, CH_2), 2.28-2.37 (1H, m, CH_2), 2.65 (1H, br s, OH), 3.33 (3H, s, SO_2CH_3), 3.80 (3H, s, OCH_3), 4.39 (1H, br s, CH_2C*H*N), 4.66-4.67 (1H, m, CH_2C*H*OH), 6.90 (2H, d, *J* 9.0 Hz, Ar H), 7.34 (2H, d, *J* 9.0 Hz, Ar H); δ_C (100 MHz, $CDCl_3$) 12.4 (CH_2), 20.1 (CH_2), 20.9 (CH_2), 40.7 (CH_3, SO_2CH_3), 55.7 (CH_3, OCH_3), 60.8 (CH, CH_2CHN), 67.1 (CH, CH_2CHOH), 91.3 (C, CH*C*OMs), 114.8 (2 x CH, Ar), 119.5 (2 x CH, Ar), 129.6 (C, ArCN), 157.3 (C, ArCO), 160.4 (C, C(O)N); m/z (ES) 364.0826 ($[M+Na]^+$ $C_{15}H_{19}NNaO_6S$ requires 364.0831), 364 (100%), 268 (17).

25: mp 86-88 °C; R_f 0.50 (Pet ether:EtOAc 1:1); ν_{max} (neat)/cm^{-1} : 2963, 1736, 1513, 1358, 1252, 1171, 824; δ_H (400 MHz, $CDCl_3$) 1.41-1.52 (1H, m, CH_2), 1.73-1.84 (1H, m, CH_2), 1.94-2.03 (1H, m, CH_2), 2.17-2.33 (3H, m, CH_2), 3.15 (3H, s, SO_2CH_3), 3.33 (3H, s, SO_2CH_3), 3.80 (3H, s, OCH_3), 4.72-4.73 (1H, m, CH_2C*H*N), 5.28-5.30 (1H, m, CH_2C*H*OMs), 6.91 (2H, d, *J* 9.0 Hz, Ar H), 7.33 (2H, d, *J* 9.0 Hz, Ar H); δ_C (100 MHz, $CDCl_3$) 12.0 (CH_2), 20.2 (CH_2), 24.0 (CH_2), 39.1 (CH_3, SO_2CH_3), 40.9 (CH_3, SO_2CH_3), 55.7 (CH_3, OCH_3), 60.7 (CH, CH_2*C*HN), 77.0 (CH, CH_2*C*HOMs), 88.7 (C, CH*C*OMs), 114.9 (2 x CH, Ar), 119.6 (2 x CH, Ar), 129.1 (C, ArCN), 157.7 (C, ArCO), 158.4 (C, C(O)N); m/z (ES) 442.0601 ($[M+Na]^+$ $C_{16}H_{21}NNaO_8S_2$ requires 442.0606), 442 (100%), 229 (23), 197 (38).

(±)-(1*R*,2*S*,6*S*)-2-Hydroxy-7-(4-methoxyphenyl)-8-oxo-7-azabicyclo[4.2.0]octan-1-yl methylbenzene sulfonate **26**

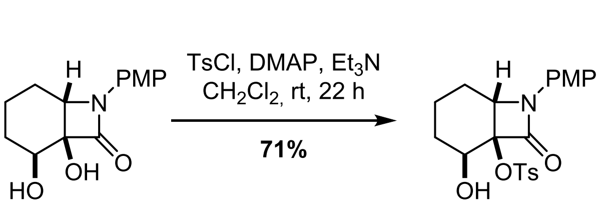

18 **26**

A novel compound prepared according to a modified literature procedure. [7] A solution of dihydroxy lactam **18** (0.100 g, 0.380 mmol) in CH_2Cl_2 (2 mL) was treated with Et_3N (58 µL, 0.42 mmol), DMAP (10 mg, 0.082 mmol) and TsCl (78 mg, 0.42 mmol). After 18 h at rt, further Et_3N (58 µL, 0.42 mmol) and TsCl (78 mg, 0.42 mmol) were added to drive the reaction to completion. After 4 h, the reaction

mixture was quenched with H_2O (2 mL) and extracted with Et_2O (2 x 3 mL). The combined organic extracts were washed with brine, dried over $MgSO_4$, filtered, evaporated under reduced pressure and purified by column chromatography (Pet ether:EtOAc 4:1 to Pet ether:EtOAc 3:2) to give *cis* hydroxyl tosyl lactam **26** (0.113 g, 71%) as a white solid. mp 84-86 °C; R_f 0.29 (Hexane:EtOAc 3:2); ν_{max} (neat)/cm^{-1} : 3524, 2968, 1746, 1512, 1172, 1048, 856; δ_H (300 MHz, $CDCl_3$) 1.32-1.42 (1H, m, CH_2), 1.62-2.15 (4H, m, CH_2), 2.28-2.38 (1H, m, CH_2), 2.43 (3H, s, $PhCH_3$), 2.88 (1H, br s, OH), 3.80 (3H, s, OCH_3), 4.30 (1H, t, *J* 3.3 Hz, CH_2C*H*N), 4.78 (1H, t, *J* 2.9 Hz, CH_2C*H*OH), 6.89 (2H, d, *J* 9.0 Hz, Ar H), 7.31-7.35 (4H, m, Ar H), 7.89 (2H, d, *J* 8.4 Hz, Ar H); δ_C (100 MHz, $CDCl_3$) 12.2 (CH_2), 20.1 (CH_2), 21.7 (CH_3, $PhCH_3$), 23.8 (CH_2), 55.5 (CH_3, OCH_3), 60.0 (CH, CH_2CHN), 66.9 (CH, CH_2*C*HOTs), 91.6 (C, CH*C*OH), 114.7 (2 x CH, Ar), 119.3 (2 x CH, Ar), 128.1 (2 x CH, Ar), 129.8 (2 x CH, Ar), 134.1 (C, ArCN), 145.4 (2 x C, Ar), 157.0 (C, ArCO), 160.2 (C, C(O)N); m/z (ES) 440.1141 ($[M+Na]^+$ $C_{21}H_{23}NNaO_6S$ requires 440.1144), 440 (100%).

(±)-(1*R*,2*S*,6*S*)-1-Hydroxy-7-(4-methoxyphenyl)-8-oxo-7-azabicyclo[4.2.0]octan-2-yl methylbenzene sulfonate **27** and (±)-(1*R*,2*S*,6*S*)-7-(4-methoxyphenyl)-8-oxo-7-azabicyclo [4.2.0]octan-1,2-diyl bis(4-methylbenzenesulfonate) **28**

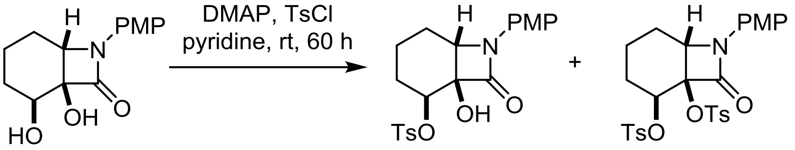

18 **27** 50% **28** 11%

Novel compounds prepared according to a modified literature procedure. [8] A solution of dihydroxy lactam **18** (0.300 g, 1.14 mmol) in pyridine (6 mL) was treated with DMAP (28 mg, 0.23 mmol) and TsCl (0.652 g, 3.42 mmol). After 60 h at rt, the reaction mixture was quenched with H_2O (3 mL) and extracted with EtOAc (2 x 5 mL). The combined organic extracts were washed sequentially with HCl (5 mL of a 1 M aq. solution) and brine (5 mL), dried over $MgSO_4$, filtered, evaporated under reduced pressure and purified by column chromatography (Pet ether:EtOAc 9:1 to Pet ether:EtOAc 1:1) to give *cis* hydroxyl tosyl lactam **27** (0.238 g, 50%) as a colourless oil and *cis* ditosyl lactam **28** (75 mg, 11%) as a white solid.

27: R_f 0.22 (Pet ether: EtOAc 1:1); ν_{max} (neat)/cm^{-1} : 3403, 2942, 1740, 1511, 1246, 871; δ_H (300 MHz, $CDCl_3$) 1.30-1.44 (1H, m, CH_2), 1.54-1.67 (1H, m, CH_2), 1.71-1.83 (1H, m, CH_2), 1.89-2.00 (1H, m, CH_2), 2.09-2.15 (2H, m, CH_2), 2.45 (3H, s, $PhCH_3$), 3.47 (1H, br s, OH), 3.78 (3H, s, OCH_3), 4.21-4.23 (1H, m, CH_2C*H*N), 4.94 (1H, t, *J* 3.3 Hz, CH_2C*H*OTs), 6.87 (2H, d, *J* 9.0 Hz, Ar H), 7.29 (2H, d, *J* 9.0 Hz, Ar H), 7.36 (2H, d, *J* 8.3 Hz, Ar H), 7.85 (2H, d, *J* 8.3 Hz, Ar H); δ_C (100 MHz, $CDCl_3$) 12.1 (CH_2), 20.4 (CH_2), 21.8 (CH_3, $PhCH_3$), 23.5 (CH_2), 55.6 (CH_3, OCH_3), 61.6 (CH, CH_2CHN), 79.1 (CH, CH_2*C*HOTs), 82.3 (C, CH*C*OH), 114.7 (2 x CH, Ar), 119.0 (2 x CH, Ar), 128.1 (2 x CH, Ar), 130.1 (2 x CH, Ar), 133.3 (C, ArCN), 145.4 (2 x C, Ar), 156.9 (C, ArCO), 163.4 (C, C(O)N); m/z (ES) 440.1138 ($[M+Na]^+$ $C_{21}H_{23}NNaO_6S$ requires 440.1144), 440 (100%), 300.2 (3).

28: mp 126-129 °C; R_f 0.76 (Pet ether: EtOAc 1:1); ν_{max} (neat)/cm^{-1} : 2967, 1757, 1512, 1358, 1259, 1174, 855; δ_H (400 MHz, $CDCl_3$) 1.35-1.46 (1H, m, CH_2), 1.69-1.88 (2H, m, CH_2), 2.17-2.34 (3H, m, CH_2), 2.42 (3H, s, $PhCH_3$), 2.45 (3H, s, $PhCH_3$), 3.81 (3H, s, OCH_3), 4.84-4.87 (2H, m, CH_2C*H*N and CH_2C*H*OTs), 6.90 (2H, d, *J* 8.8 Hz, Ar H), 7.25-7.32 (6H, m, Ar H), 7.63 (2H, d, *J* 8.4 Hz, Ar H), 7.79 (2H, d, *J* 8.4 Hz, Ar H); δ_C (100 MHz, $CDCl_3$) 11.9 (CH_2), 20.1 (CH_2), 21.8 (2 x CH_3, $PhCH_3$), 23.2 (CH_2), 55.6 (CH_3, OCH_3), 60.3 (CH, CH_2CHN), 76.8 (CH, CH_2*C*HOTs), 88.7 (C, CH*C*OTs), 114.8 (2 x CH, Ar), 119.5 (2 x CH, Ar), 128.0 (2 x CH, Ar), 128.3 (2 x CH, Ar), 129.4 (C, Ar), 129.6 (2 x CH, Ar), 129.8 (2 x CH, Ar), 133.2 (C, ArCN), 134.3

(C, Ar), 144.9 (C, Ar), 145.2 (C, Ar), 157.4 (C, ArCO), 158.5 (C, C(O)N); m/z (ES) 594.1219 ($[M+Na]^+$ $C_{28}H_{29}NNaO_8S_2$ requires 594.1232), 594 (100%).

(±)-(1*R*,2*S*,6*S*)-1-Hydroxy-7-(4-methoxyphenyl)-8-oxo-7-azabicyclo[4.2.0]octan-2-yl formate **29**

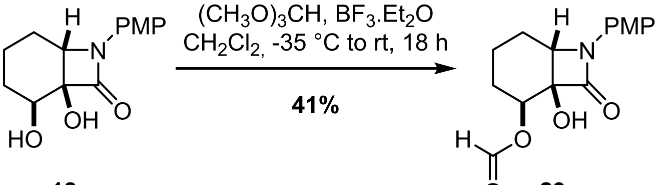

18 **29**

A novel compound prepared according to a modified literature procedure.[9] A solution of dihydroxy lactam **18** (0.100 g, 0.380 mmol) in CH_2Cl_2 (3 mL) at -35 °C was treated with trimethyl orthoformate (46 µL, 0.42 mmol) and $BF_3.Et_2O$ (53 µL, 0.42 mmol). The reaction mixture was progressively warmed to rt over 18 h. $NaHCO_3$ (3 mL of a saturated aq. solution) was then added to quench the reaction mixture and it was extracted with CH_2Cl_2 (2 x 5 mL). The combined organic extracts were washed with brine (5 mL), dried over $MgSO_4$, filtered, evaporated under reduced pressure and purified by column chromatography (Pet ether:EtOAc 4:1 to Pet ether:EtOAc 3:2) to give formate **29** (45 mg, 41%) as a yellow solid. mp 94-97 °C; R_f 0.19 (Pet ether:EtOAc 3:2); ν_{max} (neat)/cm^{-1} : 3336, 2956, 1708, 1512, 1439, 1160, 832; δ_H (400 MHz, $CDCl_3$) 1.39-1.50 (1H, m, CH_2), 1.58-1.68 (1H, m, CH_2), 1.86-1.99 (2H, m, CH_2), 2.12-2.17 (2H, m, CH_2), 3.78 (3H, s, OCH_3), 4.19 (1H, t, *J* 3.2 Hz, CH_2C*H*N), 4.37 (1H, br s, OH), 5.47 (1H, t, *J* 3.6 Hz, CH_2C*H*O), 6.86 (2H, d, *J* 9.0 Hz, Ar H), 7.30 (2H, d, *J* 9.0 Hz, Ar H), 8.18 (1H, s, HC(O)O); δ_C (100 MHz, $CDCl_3$) 12.6 (CH_2), 20.8 (CH_2), 22.8 (CH_2), 55.6 (CH_3, OCH_3), 61.3 (CH, CH_2*C*HN), 70.4 (CH, CH_2*C*HO), 82.4 (C, CH*C*OH), 114.7 (2 x CH, Ar), 119.1 (2 x CH, Ar), 130.2 (C, ArCN), 156.9 (C, ArCO), 161.1 (CH, HC(O)O), 164.8 (C, C(O)N); m/z (ES) 314.1000 ($[M+Na]^+$ $C_{15}H_{17}NNaO_5$ requires 314.1004), 314 (100%).

Optimization studies for the semipinacol rearrangement of cyclic sulfites 30 and 31:

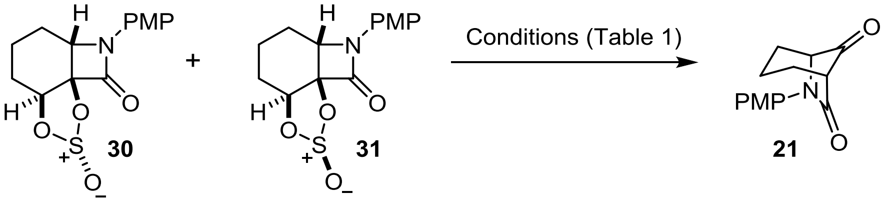

30 **31** **21**

Entry	Reagents	work-up	T / time	Result
**1**	CH_3CN	concentration	reflux / 5 h	**30:31**
**2**	toluene	concentration	reflux / 5 h	**30:31**
**3**	triethylbenzene	/	190 °C / 3.5 h	**21** 77%
**4**	Ph_2O	/	190 °C / 2 h	**21** 91%
**5**	DMF	H_2O, EtOAc	reflux / 4 h	**21** 31%
**6**	DMSO	H_2O, EtOAc	150 °C / 18 h	degradation
**7**	$BF_3.Et_2O$ (2.1 eq), toluene	concentration	reflux/ 18 h	degradation
**8**	$BF_3.Et_2O$ (2.1 eq), toluene	H_2O, EtOAc	reflux / 6 h	**21** 49%

N-(Cyclopent-2-en-1-yl)-4-methoxyaniline **93**

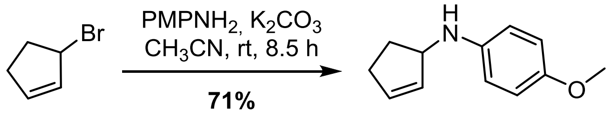

93

A novel compound prepared according to a modified literature procedure.[10] A solution of 4-methoxyaniline (1.50 g, 12.2 mmol) in CH_3CN (12 mL) was treated with 3-bromocyclopentene (0.662 g, 4.50 mmol) and K_2CO_3 (0.684 g, 4.95 mmol). After 8.5 h at rt, the reaction mixture was quenched with NH_4Cl (10 mL of a saturated aq. solution) and extracted with Et_2O (2 x 15 mL). The combined organic extracts were washed with brine (20 mL), dried over $MgSO_4$, filtered, evaporated under reduced pressure and purified by column chromatography (Hexane to Hexane:EtOAc 96:4) to give amine **93** (0.601 g, 71%) as an orange oil. R_f 0.52 (Pet ether:EtOAc 9:1); ν_{max} (neat)/cm^{-1}: 3375, 2933, 1507, 1229, 1034, 816, 722; δ_H (400 MHz, $CDCl_3$) 1.61-1.70 (1H, m, CH_2), 2.28-2.40 (2H, m, CH_2), 2.43-2.53 (1H, m, CH_2), 3.26 (1H, br s, NH), 3.76 (3H, s, OCH_3), 4.46-4.54 (1H, m, CH_2C*H*N), 5.85 (1H, dq, *J* 2.2 and 5.8 Hz, CH_2C*H*=CH), 5.94-5.97 (1H, m, CH=C*H*CHN), 6.62 (2H, d, *J* 9.0 Hz, Ar H), 6.79 (2H, d, *J* 9.0 Hz, Ar H); δ_C (100 MHz, $CDCl_3$) 31.3 (2 x CH_2), 55.9 (CH_3, OCH_3), 60.6 (CH, CH_2*C*HN), 115.0 (2 x CH, Ar), 115.1 (2 x CH, Ar), 132.3 (CH, CH_2CH=CH), 134.0 (CH, CH=CHCHN), 142.0 (C, ArCN), 152.3 (C, ArCO); m/z (ES) 190.1225 ($[M+H]^+$ $C_{12}H_{16}NO$ requires 190.1226), 190 (100%).

- Cyclopent-2-en-1-yl(4-methoxyphenyl)carbamic chloride **94**

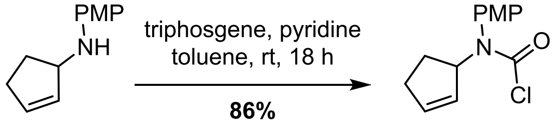

93 **94**

A novel compound prepared according to a modified literature procedure.[10] A solution of triphosgene (1.20 g, 4.04 mmol) in toluene (74 mL) was treated with pyridine (1.06 mL, 13.1 mmol) and subsequently with a solution of amine **93** (2.06 g, 10.9 mmol) in toluene (10 mL). The reaction mixture was stirred for 18 h at rt, quenched with NH_4Cl (70 mL of a saturated aq. solution) and extracted with Et_2O (2 x 70 mL). The combined organic extracts were washed sequentially with HCl (100 mL of a 0.25 M aq. solution), H_2O (100 mL) and brine (100 mL), dried over $MgSO_4$, filtered and evaporated under reduced pressure to give carbamoyl chloride **94** (2.36 g, 86%) as a yellow solid. mp 66-69 °C; R_f 0.76 (Pet ether:EtOAc 4:1); ν_{max} (neat)/cm^{-1}: 2933, 1727, 1509, 1458, 1394, 1212, 1030, 930, 739; δ_H (400 MHz, $CDCl_3$) (as a mixture of rotamers) 1.80-1.96 (2H, m, CH_2), 2.07-2.19 (1H, m, CH_2), 2.21-2.30 (1H, m, CH_2), 3.81 (3H, s, OCH_3), 5.46-5.59 (1H, m, CH_2C*H*N), 5.65-5.68 (1H, m, CH_2C*H*=CH), 5.81-5.86 (1H, m, CH=C*H*CHN), 6.85 (2H, d, *J* 8.0 Hz, Ar H), 6.92-7.13 (2H, m, Ar H); δ_C (100 MHz, $CDCl_3$) 28.3 (CH_2), 31.4 (CH_2), 55.5 (CH_3, OCH_3), 66.6 (CH, CH_2*C*HN), 114.1 (2 x CH, Ar), 129.2 (CH, CH_2CH=CH), 131.1 (2 x CH, Ar), 131.8 (C, ArCN), 136.7 (CH, CH=*C*HCHN), 149.8 (C, ArCO), 159.6 (C, NC(O)Cl); m/z (ES) 274.0608 ($[M+Na]^+$ $C_{13}H_{14}{}^{35}ClNNaO_2$ requires 274.0605), 276 (33%), 274 (100), 252 (22), 186 (17), 172 (38).

Diethylthiocarbamic acid-[(4-methoxyphenyl)(cyclopent-2-enyl)carbamic acid]-thioanhydride **34**

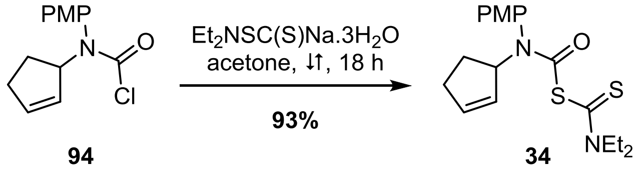

94 **34**

A novel compound prepared according to a modified literature procedure.[10] A solution of carbamoyl chloride **94** (0.658 g, 2.61 mmol) in acetone (20 mL) was treated with sodium diethyldithiocarbamate trihydrate (2.36 g, 10.5 mmol). The solution was heated at reflux for 18 h, quenched with H_2O (20 mL) and extracted with Et_2O (3 x 20 mL). The combined organic extracts were washed with brine (70 mL), dried over $MgSO_4$, filtered, evaporated under reduced pressure and purified by column chromatography (Pet ether to Pet ether:EtOAc 7:3) to give dithiocarbamate **34** (0.885 g, 93%) as a yellow oil. R_f 0.32 (Pet ether:EtOAc 4:1); ν_{max} (neat)/cm^{-1}: 2933, 1742, 1667, 1508, 1417, 1242, 1028, 916, 741; δ_H (400 MHz, $CDCl_3$) (as a mixture of rotamers) 1.21 (3H, t, *J* 7.0 Hz, CH_2CH_3), 1.31 (3H, t, *J* 7.0 Hz, CH_2CH_3), 1.74-1.81 (1H, m, CH_2), 1.85-1.93 (1H, m, CH_2), 2.08-2.15 (1H, m, CH_2), 2.17-2.27 (1H, m, CH_2), 3.77 (2H, q, *J* 7.0 Hz, CH_2CH_3), 3.80 (3H, s, OCH_3), 3.99 (2H, q, *J* 7.0 Hz, CH_2CH_3), 5.62-5.72 (2H, m, CH_2CHN and CH_2CH=CH), 5.79-5.82 (1H, m, CH=C*H*CHN), 6.83 (2H, d, *J* 8.4 Hz, Ar H), 7.04-7.13 (2H, m, Ar H); δ_C (100 MHz, $CDCl_3$) 11.1 (CH_3, CH_2CH_3), 13.6 (CH_3, CH_2CH_3), 28.3 (CH_2), 31.4 (CH_2), 48.7 (CH_2, CH_2CH_3), 50.4 (CH_2, CH_2CH_3), 55.5 (CH_3, OCH_3), 64.0 (CH, CH_2CHN), 114.2 (2 x CH, Ar), 129.3 (C, ArCN), 129.8 (CH, CH_2CH=CH), 132.4 (2 x CH, Ar), 136.1 (CH, CH=*C*HCHN), 160.2 (C, ArCO), 164.1 (C, C(O)N), 185.7 (C, SC(S)); m/z (ES) 365.1356 ([M+H]$^+$ $C_{18}H_{25}N_2O_2S_2$ requires 365.1352), 397 (34%), 365 (100), 289 (53), 150 (10).

(±)-(1*R*,2*S*,5*S*)-6-(4-Methoxyphenyl)-7-oxo-6-azabicyclo[3.2.0]heptan-2-yl diethylcarbamodithioate **47**

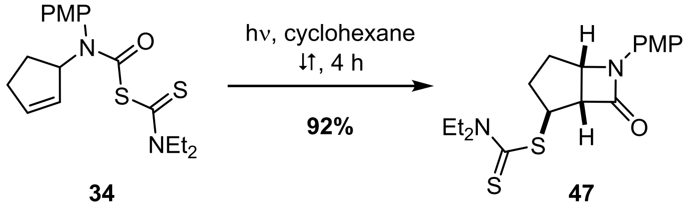

34 **47**

A novel compound prepared according to a modified literature procedure.[1] A solution of dithiocarbamate **34** (2.87 g, 7.86 mmol) in cyclohexane (79 mL) was degassed for 10 min and irradiated with a 500 W lamp which generated enough heat to bring the solution to reflux. After 4 h, the solvent was removed under reduced pressure and the crude product obtained was purified by column chromatography (Pet ether to Pet ether:EtOAc 1:1) to give lactam **47** (2.62 g, 92%) as a beige solid. mp 117-119 °C; R_f 0.45 (Pet ether:EtOAc 7:3); ν_{max} (neat)/cm^{-1}: 2929, 1721, 1509, 1398, 1206, 1031, 914, 830, 800; δ_H (300 MHz, $CDCl_3$) 1.27 (6H, dt, *J* 2.6 and 7.1 Hz, CH_2CH_3), 1.71-1.84 (1H, m, CH_2), 2.12-2.35 (3H, m, CH_2), 3.70 (2H, q, *J* 7.1 Hz, CH_2CH_3), 3.78 (3H, s, OCH_3), 3.80-3.85 (1H, m, CHC*H*C(O)), 4.01 (2H, q, *J* 7.1 Hz, CH_2CH_3), 4.48 (1H, t, *J* 4.2 Hz, CH_2CHS), 4.61 (1H, d, *J* 5.4 Hz, CH_2CHN), 6.88 (2H, d, *J* 9.0 Hz, Ar H), 7.35 (2H, d, *J* 9.0 Hz, Ar H); δ_C (100 MHz, $CDCl_3$) 11.7 (CH_3, CH_2CH_3), 12.7 (CH_3, CH_2CH_3), 25.8 (CH_2), 30.2 (CH_2), 46.8 (CH_2, CH_2CH_3), 49.1 (CH_2, CH_2CH_3), 49.2 (CH, CH*C*HC(O)), 55.6 (CH_3, OCH_3), 57.1 (CH, CH_2CHS), 59.9 (CH, CH_2CHN), 114.6 (2 x CH, Ar), 118.1 (2 x CH, Ar), 130.9 (C, ArCN), 156.2 (C, ArCO), 163.2 (C, C(O)N), 193.5 (C, SC(S)); m/z (ES) 365.1356 ([M+H]$^+$ $C_{18}H_{25}N_2O_2S_2$ requires 365.1352), 365 (100%).

(±)-(2*R*,5*S*)-*N*-Benzyl-3-bromobicyclo[3.2.1]oct-3-en-2-amine **41a**

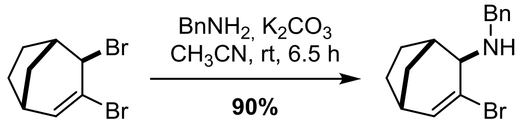

40 **41a**

A novel compound prepared according to a modified literature procedure.[10] A solution of benzylamine (7.81 mL, 71.5 mmol) in CH_3CN (72 mL) was treated with dibromide **40**[4a] (7.04 g, 26.5 mmol) and K_2CO_3 (4.03 g, 29.1 mmol). After 6.5 h at rt, the reaction was quenched with H_2O (70 mL) and extracted with Et_2O (2 x 70 mL). The combined organic extracts were washed with brine (90 mL), dried over $MgSO_4$, filtered, concentrated under reduced pressure and purified by column chromatography (Hexane:Et_2O 98:2 to Hexane:Et_2O 9:1) to give amine **41a** (6.98 g, 90%) as a pale yellow oil. R_f 0.32 (Pet ether:Et_2O 95:5); ν_{max} (neat)/cm^{-1} : 3332, 2940, 1630, 1452, 1331, 1107, 1027, 882; δ_H (400 MHz, $CDCl_3$) 1.28-1.40 (2H, m, CH_2), 1.60-1.69 (1H, m, CH_2), 1.73-1.79 (2H, m, CH_2 and NH), 1.86-1.95 (2H, m, CH_2), 2.51-2.59 (2H, m, CH_2C*H*CHN and CH_2C*H*CH=), 2.96 (1H, d, *J* 2.4 Hz, CHC*H*N), 3.88, 3.98 (2H, ABq, *J* 13.2 Hz, NCH_2Ph), 6.31 (1H, d, *J* 6.8 Hz, C*H*=CBr), 7.26-7.44 (5H, m, Ar H); δ_C (100 MHz, $CDCl_3$) 26.5 (CH_2), 30.7 (CH_2), 32.6 (CH_2), 38.2 (CH), 38.3 (CH), 52.3 (CH_2, NCH_2Ph), 68.5 (CH, CH*C*HN), 124.3 (C, =CBr), 127.0 (CH, Ar), 128.3 (2 x CH, Ar), 128.5 (2 x CH, Ar), 138.1 (CH, CH*C*H=CBr), 140.6 (C, Ar); m/z (ES) 292.0704 (M^+, $C_{15}H_{19}{}^{79}BrN$ requires 292.0701), 294 (89%), 292 (100), 187 (33), 185 (28).

(±)- (2*R*,5*S*)-3-Bromo-*N*-(4-methoxyphenyl)bicyclo[3.2.1]oct-3-en-2-amine **41b**

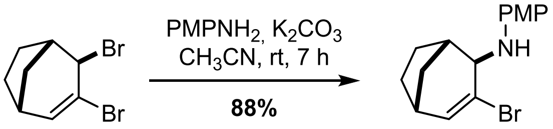

40 **41b**

A novel compound prepared according to a modified literature procedure.[10] A solution of 4-methoxyaniline (6.28 g, 38.9 mmol) in CH_3CN (51 mL) was treated with dibromide **40**[4a] (5.02 g, 18.9 mmol) and K_2CO_3 (2.87 g, 20.8 mmol). After 7 h at rt, the reaction mixture was quenched with H_2O (50 mL) and extracted with Et_2O (2 x 50 mL). The combined organic extracts were washed with brine (75 mL), dried over $MgSO_4$, filtered, evaporated under reduced pressure and purified by column chromatography (Pet ether to Pet ether:Et_2O 4:1) to give amine **41b** (5.11 g, 88%) as a beige solid. mp 105-108 °C; R_f 0.59 (Pet ether:Et_2O 4:1); ν_{max} (neat)/cm^{-1} : 3381, 2935, 1509, 1229, 1032, 815, 761; δ_H (400 MHz, $CDCl_3$) 1.28-1.35 (1H, m, CH_2), 1.41-1.51 (1H, m, CH_2), 1.60-1.73 (2H, m, CH_2), 1.79 (1H, ddt, *J* 2.3, 9.0 and 11.6 Hz, CH_2), 1.94 (1H, dddd, *J* 2.6, 7.9, 10.6 and 13.5 Hz, CH_2), 2.54-2.60 (1H, m, CH_2C*H*CH=), 2.63 (1H, t, *J* 6.3 Hz, CH_2C*H*CHN), 3.61 (1H, d, *J* 2.0 Hz, CHC*H*N), 3.70 (1H, br s, NH), 3.75 (3H, s, OCH_3), 6.39 (1H, d, *J* 7.1 Hz, CH=CBr), 6.62 (2H, d, *J* 8.9 Hz, Ar H), 6.80 (2H, d, *J* 8.9 Hz, Ar H); δ_C (100 MHz, $CDCl_3$) 26.0 (CH_2), 31.0 (CH_2), 32.4 (CH_2), 38.1 (CH, CH_2*C*HCH=), 38.9 (CH, CH_2*C*HCHN), 56.0 (CH_3, OCH_3), 65.5 (CH, CH*C*HN), 114.8 (2 x CH, Ar), 115.2 (2 x CH, Ar), 122.3 (C, =CBr), 139.3 (CH, CH*C*H=CBr), 141.4 (C, ArCN), 152.5 (C, ArCO); m/z (ES) 330.0453 ($[M+Na]^+$ $C_{15}H_{18}{}^{79}BrNNaO$ requires 330.0469), 332 (100%), 330 (100), 310 (43),308 (43).

(±)-(2*R*,5*S*)-*N*-(4-Methoxyphenyl)bicyclo[3.2.1]oct-3-en-2-amine **42**

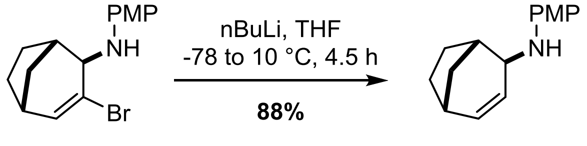

41b **42**

A novel compound prepared according to a modified literature procedure.[11] A solution of amine **41b** (1.46 g, 4.73 mmol) in THF (47 mL) was cooled to -78 °C. *n*BuLi (12.7 mL of a 1.12 M solution in hexane, 14.2 mmol) was added dropwise over 10 min. The reaction mixture was allowed to warm to 10 °C over 4.5 h, quenched with NH_4Cl (50 mL of a saturated aq. solution) and extracted with Et_2O (2 x 50 mL). The combined organic extracts were washed with brine (75 mL), dried over $MgSO_4$, filtered, evaporated under reduced pressure and purified by column chromatography (Pet ether to Pet ether:Et_2O 9:1) to give amine **42** (0.958 g, 88%) as a yellow solid. mp 78-79 °C; R_f 0.54 (Pet ether:Et_2O 4:1); ν_{max} (neat)/cm^{-1} : 3369, 2932, 2867, 1616, 1507, 1225, 1034, 814, 770; δ_H (400 MHz, $CDCl_3$) 1.22-1.30 (1H, m, CH_2), 1.30-1.41 (1H, m, CH_2), 1.59 (1H, d, *J* 11.1 Hz, CH_2), 1.62-1.75 (2H, m, CH_2), 1.85-1.97 (1H, m, CH_2), 2.46-2.57 (2H, m, CH_2C*H*CHN and CH_2C*H*CH=), 3.45 (1H, br s, NH), 3.52-3.57 (1H, m, CHC*H*N), 3.76 (3H, s, OCH_3), 5.47 (1H, ddd, *J* 1.7, 4.0 and 9.4 Hz, CHC*H*=CH), 6.09 (1H, ddt, *J* 1.2, 6.7 and 9.4 Hz, NCHC*H*=CH), 6.60 (2H, d, *J* 8.9 Hz, Ar H), 6.80 (2H, d, *J* 8.9 Hz, Ar H); δ_C (100 MHz, $CDCl_3$) 26.7 (CH_2), 31.4 (CH_2), 32.7 (CH_2), 35.8 (CH, CH_2*C*HCH=), 36.7 (CH, CH_2*C*HCHN), 56.0 (CH_3, OCH_3), 57.6 (CH, CH*C*HN), 114.6 (2 x CH, Ar), 115.2 (2 x CH, Ar), 124.8 (CH, CH*C*H=CH), 137.9 (CH, NCH*C*H=CH), 141.4 (C, ArCN), 152.0 (C, ArCO); m/z (ES) 230.1542 ($[M+H]^+$, $C_{15}H_{20}NO$ requires 230.1539), 532 (16%), 445 (15), 371 (52), 355 (16), 230 (100), 149 (11).

(±)-(2*R*,5*S*)-Bicyclo[3.2.1]oct-3-en-2-yl(4-methoxyphenyl)carbamic chloride **95**

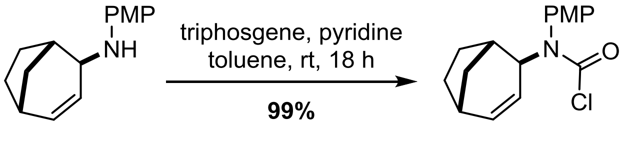

42 **95**

A novel compound prepared according to a modified literature procedure.[10] A solution of triphosgene (0.556 g, 1.87 mmol) in toluene (10 mL) was treated with pyridine (0.492 mL, 6.08 mmol) and subsequently with a solution of amine **42** (1.16 g, 5.07 mmol) in toluene (20 mL). The reaction mixture was stirred for 18 h at rt, quenched with NH_4Cl (20 mL of a saturated aq. solution) and extracted with Et_2O (2 x 30 mL). The combined organic extracts were washed sequentially with HCl (50 mL of a 0.25 M aq. solution) and brine (50 mL), dried over $MgSO_4$, filtered and evaporated under reduced pressure to give carbamoyl chloride **95** (1.46 g, 99%) as a brown solid. mp 73-74 °C; R_f 0.54 (Pet ether:Et_2O 4:1); ν_{max} (neat)/cm^{-1}: 2940, 1723, 1509, 1246, 1203, 1033, 735; δ_H (400 MHz, $CDCl_3$) 0.36 (1H, d, *J* 11.2 Hz, CH_2), 0.98 (1H, dddd, *J* 1.1, 3.8, 4.8 and 11.2 Hz, CH_2), 1.36-1.43 (1H, m, CH_2), 1.57-1.62 (2H, m, CH_2), 1.88-2.01 (1H, m, CH_2), 2.28-2.32 (1H, m, CH_2C*H*CH=), 2.58 (1H, t, *J* 6.1 Hz, CH_2C*H*CHN), 3.81 (3H, s, OCH_3), 4.61-4.69 (1H, m, CHC*H*N), 5.43 (1H, ddd, *J* 1.7, 3.6 and 9.5 Hz, CHC*H*=CH), 6.06 (1H, m, NCHC*H*=CH), 6.83-7.19 (4H, m, Ar H); δ_C (100 MHz, $CDCl_3$) (as a mixture of rotamers) 29.3 (CH_2), 30.3 (CH_2), 32.8 (CH_2), 34.7 (CH, CH_2*C*HCH=), 38.8 (CH, CH_2*C*HCHN), 55.6 (CH_3, OCH_3), 62.6 (CH, CH*C*HN), 113.9, 114.2 (2 x CH, Ar), 122.3 (CH, CH*C*H=CH), 131.2, 132.0 (2 x CH, Ar), 132.6 (C, ArCN), 139.5 (CH, NCH*C*H=CH), 150.1 (C, ArCO), 159.7 (C, NC(O)Cl); m/z (ES) 314.0934 ($[M+Na]^+$, $C_{16}H_{18}{}^{35}ClNNaO_2$ requires 314.0934), 316 (13%), 314 (76), 310 (26), 241 (18), 239 (100).

(±)-(2*R*,5*S*)-Diethyldithiocarbamic acid-[(4-methoxyphenyl)(bicyclo[3.2.1]oct-3-en-2-yl)carbamic acid]-thioanhydride **36**

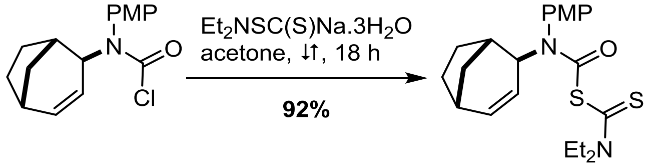

95 **36**

A novel compound prepared according to a modified literature procedure.[10] A solution of carbamoyl chloride **95** (0.223 g, 0.764 mmol) in acetone (5.9 mL) was treated with sodium diethyldithiocarbamate trihydrate (0.517 g, 2.29 mmol). The reaction mixture was heated at reflux for 18 h, quenched with H_2O (6 mL) and extracted with Et_2O (3 x 6 mL). The combined organic extracts were washed with brine (10 mL), dried over $MgSO_4$, filtered, evaporated under reduced pressure and purified by column chromatography (Pet ether to Pet ether:EtOAc 4:1) to give dithiocarbamate **36** (0.285 g, 92%) as a yellow solid. mp 131-132 °C (CH_2Cl_2); R_f 0.37 (Pet ether:EtOAc 4:1); ν_{max} (neat)/cm^{-1}: 2946, 1673, 1507, 1486, 1424, 1228, 1191, 821; δ_H (400 MHz, $CDCl_3$) (as a mixture of rotamers) 0.86 (1H, d, *J* 11.2 Hz, CH_2), 0.92-1.00 (1H, m, CH_2), 1.20 (3H, t, *J* 7.1 Hz, CH_2CH_3), 1.29 (3H, t, *J* 7.1 Hz, CH_2CH_3), 1.32-1.40 (1H, m, CH_2), 1.49-1.57 (2H, m, CH_2), 1.82-1.94 (1H, m, CH_2), 2.17-2.29 (1H, m, CH_2CHCH=), 2.53 (1H, t, *J* 6.0 Hz, CH_2CHCHN), 3.76 (2H, q, *J* 7.1 Hz, CH_2CH_3), 3.79 (3H, s, OCH_3), 3.97 (2H, q, *J* 7.1 Hz, CH_2CH_3), 4.76 (1H, br s, CHC*H*N), 5.36 (1H, ddd, *J* 1.7, 3.7 and 9.5 Hz, CHC*H*=CH), 6.00 (1H, app t, *J* 8.2 Hz, NCHC*H*=CH), 6.83 (2H, app t, *J* 6.8 Hz, Ar H), 7.03 (1H, d, *J* 8.4 Hz, Ar H), 7.21 (1H, d, *J* 8.4 Hz, Ar H); δ_C (100 MHz, $CDCl_3$) (as a mixture of rotamers) 11.1 (CH_3, CH_2CH_3), 13.6 (CH_3, CH_2CH_3), 29.1 (CH_2), 30.4 (CH_2), 32.7 (CH_2), 34.7 (CH, CH_2CHCH=), 38.9 (CH, CH_2CHCHN), 48.7 (CH_2, CH_2CH_3), 50.4 (CH_2, CH_2CH_3), 55.5 (CH_3, OCH_3), 60.0 (CH, CHCHN), 114.1 (2 x CH, Ar), 122.7 (CH, CHCH=CH), 130.0 (C, ArCN), 132.5, 133.1 (2 x CH, Ar), 139.1 (CH, NCHCH=CH), 160.2 (C, ArCO), 164.3 (C, C(O)N), 185.7 (C, C(S)S); m/z (ES) 427.1491 ($[M+Na]^+$, $C_{21}H_{28}N_2NaO_2S_2$ requires 427.1490), 854 (28%), 833 (42), 832 (74), 831 (100), 690 (11), 506 (56), 450 (45), 427 (46), 405 (17), 256 (19).

(±)-(1*S*,2*R*,5*S*,6*R*,7*R*)-3-(4-Methoxyphenyl)-4-oxo-3-azatricyclo[5.2.1.0^{2,5}]decan-6-yl diethyldithiocarbamate **50** and (±)-(1*S*,2*R*,5*S*,6*S*,7*R*)-3-(4-methoxyphenyl)-4-oxo-3-azatricyclo[5.2.1.0^{2,5}]decan-6-yl diethyldithiocarbamate **51**

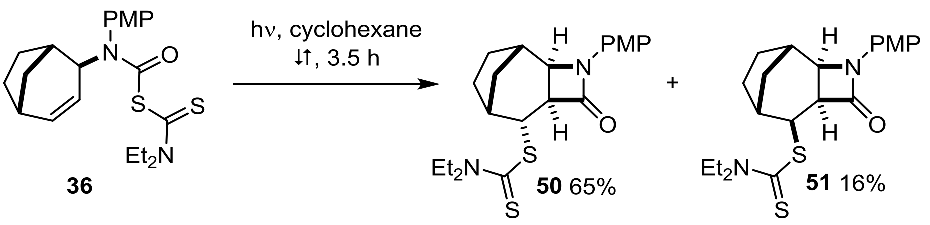

36 **50** 65% **51** 16%

Novel compounds prepared according to a modified literature procedure.[1] A solution of dithiocarbamate **36** (1.65 g, 4.07 mmol) in cyclohexane (41 mL) was degassed for 10 min and irradiated with a 500 W lamp which generated enough heat to bring the solution to reflux. After 3.5 h, the solvent was removed under reduced pressure and the crude product obtained was purified by column chromatography (Pet ether to Pet ether:EtOAc 85:15) to give *cis*-lactam **50** (1.07 g, 65%) and *trans*-lactam **51** (0.265 g, 16%) as pale yellow solids.

50: mp 160-162 °C; R_f 0.50 (Pet ether:EtOAc 7:3); ν_{max} (neat)/cm^{-1}: 2932, 1739, 1512, 1243, 825; δ_H (400 MHz, $CDCl_3$) 1.19-1.33 (6H, m, CH_2CH_3), 1.43-1.56 (2H, m, CH_2), 1.64-1.91 (4H, m, CH_2), 2.71 (1H,

t, J 5.2 Hz, CH_2CHCHS), 2.79 (1H, q, J 5.6 Hz, CH_2CHCHN), 3.19 (1H, t, J 5.1 Hz, CHC*H*C(O)), 3.70 (2H, q, J 7.0 Hz, CH_2CH_3), 3.75 (3H, s, OCH_3), 3.83-4.12 (3H, m, CH_2CH_3 and CHC*H*S), 4.71 (1H, t, J 4.8 Hz, CHC*H*N), 6.84 (2H, d, J 9.0 Hz, Ar H), 7.33 (2H, d, J 9.0 Hz, Ar H); δ_C (100 MHz, $CDCl_3$) 11.6 (CH_3, CH_2CH_3), 12.6 (CH_3, CH_2CH_3), 24.7 (CH_2), 27.5 (CH_2), 31.1 (CH_2), 34.5 (CH, CH_2CHCHS), 37.1 (CH, CH_2CHCHN), 46.7 (CH_2, CH_2CH_3), 48.2 (CH, CHC*H*C(O)), 49.2 (CH_2, CH_2CH_3), 50.8 (CH, CH*C*HN), 55.6 (CH_3, OCH_3), 57.9 (CH, CH*C*HS), 114.5 (2 x CH, Ar), 118.6 (2 x CH, Ar), 130.9 (C, ArCN), 156.1 (C, ArCO), 166.0 (C, C(O)N), 193.4 (C, C(S)S); m/z (ES) 427.1485 ($[M+Na]^+$ $C_{21}H_{28}N_2NaO_2S_2$ requires 427.1490), 833 (19%), 832 (37), 831 (59), 428 (27), 427 (100), 405 (34).

51: mp 134-136 °C; R_f 0.70 (Pet ether:EtOAc 7:3); ν_{max} (neat)/cm^{-1}: 2935, 1729, 1511, 1242, 1027, 835; δ_H (400 MHz, $CDCl_3$) 1.27 (3H, t, J 7.1 Hz, CH_2CH_3), 1.33 (3H, t, J 7.1 Hz, CH_2CH_3), 1.53-1.63 (1H, m, CH_2), 1.75-2.03 (5H, m, CH_2), 2.50 (1H, t, J 6.1 Hz, CH_2CHCHN), 2.73 (1H, t, J 5.7 Hz, CH_2CHCHS), 3.71-4.14 (6H, m, CH_2CH_3, CHC*H*C(O) and CHC*H*S), 3.77 (3H, s, OCH_3), 4.32 (1H, d, J 10.7 Hz, CHC*H*N), 6.86 (2H, d, J 9.0 Hz, Ar H), 7.35 (2H, d, J 9.0 Hz, Ar H); δ_C (100 MHz, $CDCl_3$) 11.7 (CH_3, CH_2CH_3), 12.8 (CH_3, CH_2CH_3), 26.7 (CH_2), 27.6 (CH_2), 31.9 (CH_2), 35.0 (CH, CH_2CHCHS), 41.9 (CH, CH_2CHCHN), 45.1 (CH, CH*C*HC(O)), 47.2 (CH_2, CH_2CH_3), 49.6 (CH_2, CH_2CH_3), 51.1 (CH, CH*C*HN), 55.7 (CH_3, OCH_3), 57.4 (CH, CH*C*HS), 114.6 (2 x CH, Ar), 118.6 (2 x CH, Ar), 130.9 (C, ArCN), 156.1 (C, ArCO), 165.2 (C, C(O)N), 195.2 (C, C(S)S); m/z (ES) 427.1485 ($[M+Na]^+$ $C_{21}H_{28}N_2NaO_2S_2$ requires 427.1490), 428 (18), 427 (100), 405 (15).

N-Isopropylcyclohexa-2,5-dienamine **44**

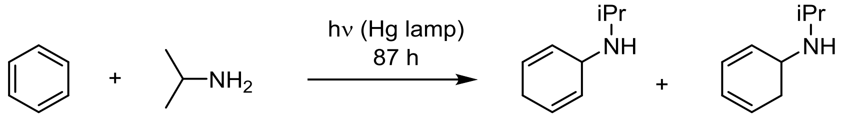

44 **45**

A novel compound prepared according to a modified literature procedure.[12] A solution of benzene (170 mL) and *i*$PrNH_2$ (170 mL) was degassed under argon for 30 min, before irradiating with a low pressure mercury lamp for 87 h. A few crystals of 3-*tert*-butyl-4-hydroxy-5-methylphenyl sulfide were added and the solvent was removed under reduced pressure. A brown oil was obtained and purification by Kugelrohr distillation gave a 5:3 mixture of amines **44:45** (2.81 g) as a yellow oil. δ_H (400 MHz, $CDCl_3$) (mixture of **44** and **45**) 1.00 (6H, app t, J 6.1 Hz, $CH(CH_3)_2$ for **45**), 1.03 (6H, d, J 6.2 Hz, $CH(CH_3)_2$ for **44**), 2.32 (2H, m, $=CHCH_2CHN$ for **45**), 2.50-2.70 (2H, m, $=CHCH_2CH=$ for **44**), 2.95 (1H, hept, J 6.1 Hz, $CH(CH_3)_2$ for **45**), 3.07 (1H, hept, J 6.2 Hz, $CH(CH_3)_2$ for **44**), 3.29-3.40 (1H, m, CHN for **45**), 3.59-3.72 (1H, m, CHN for **44**), 5.73-5.95 (8H, m, =CH); δ_C (100 MHz, $CDCl_3$) (mixture of **44:45**) 23.2 (CH_3, $CH(CH_3)_2$ for **45**), 23.4 (CH_3, $CH(CH_3)_2$ for **45**), 23.7 (2 x CH_3, $CH(CH_3)_2$ for **44**), 26.6 (CH_2, $=CHCH_2CH=$ for **44**), 30.0 (CH_2, $=CHCH_2CHN$ for **45**), 44.8 (CH, $CH(CH_3)_2$ for **45**), 45.1 (CH, $CH(CH_3)_2$ for **44**), 47.2 (CH, CH_2CHN for **45**), 47.8 (CH, CH_2CHN for **44**), 124.1 (CH, =CH for **45**), 124.3 (CH, =CH for **45**), 125.5 (2 x CH, =CH for **44**), 128.3 (2 x CH, =CH for **44**), 129.8 (2 x CH, =CH for **45**).

Diethyldithiocarbamic acid-[isopropyl(cyclohexa-2,5-dien-1-yl)carbamic acid]-thioanhydride **37**

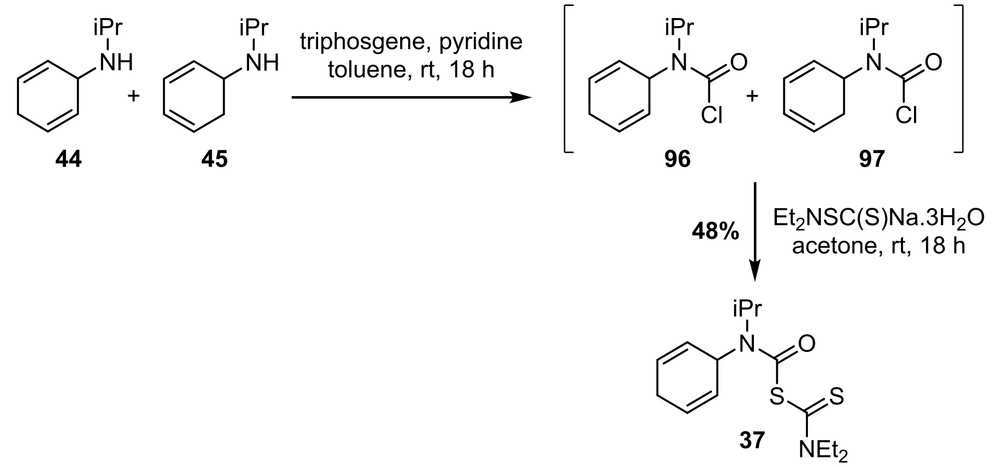

A novel compound prepared according to a modified literature procedure.[10] A solution of triphosgene (1.20 g, 4.03 mmol) in toluene (68 mL) was treated with pyridine (1.82 mL, 13.1 mmol) and subsequently with a solution of a 5:3 mixture of amines **44:45** (1.49 g, 10.9 mmol) in toluene (15 mL). The reaction mixture was stirred for 18 h at rt, quenched with NH_4Cl (50 mL of a saturated aq. solution) and extracted with Et_2O (2 x 50 mL). The combined organic extracts were washed sequentially with HCl (75 mL of a 0.5 M aq. solution) and brine (50 mL), dried over $MgSO_4$, filtered and evaporated under reduced pressure to give carbamoyl chlorides **96:97** (1.58 g, 73%) as a brown oil.

A solution of carbamoyl chlorides **96:97** (1.58 g, 7.92 mmol) in acetone (61 mL) was treated with sodium diethyldithiocarbamate trihydrate (7.13 g, 31.7 mmol). The reaction mixture was stirred at rt for 18 h, quenched with H_2O (60 mL) and extracted with Et_2O (3 x 60 mL). The combined organic extracts were washed with brine (100 mL), dried over $MgSO_4$, filtered, evaporated under reduced pressure and purified by column chromatography (Pet ether to Pet ether:EtOAc 9:1) to give carbamoyl dithiocarbamate **37** (1.63 g, 48% from the 5:3 mixture of amines **44:45**) as a yellow solid. mp 108-111 °C; R_f 0.50 (Pet ether:Et_2O 4:1); ν_{max} (neat)/cm^{-1}: 2969, 1682, 1673, 1486, 1414, 1250, 1198, 1183, 1071, 912, 812, 772; δ_H (400 MHz, $CDCl_3$) 1.25-1.38 (12H, m, $CH(CH_3)_2$ and CH_2CH_3), 2.53-2.83 (2H, m, =CHCH_2CH=), 3.46 (1H, s, CH($CH_3)_2$), 3.77 (2H, q, *J* 7.1 Hz, CH_2CH$_3$), 4.00 (2H, q, *J* 7.1 Hz, CH_2CH$_3$), 4.98 (1H, s, =CHC*H*N), 5.67 (2H, d, *J* 9.2 Hz, =CH), 5.99 (2H, d, *J* 8.6 Hz, =CH); δ_C (100 MHz, $CDCl_3$) 11.4 (CH_3, CH_2CH_3), 13.4 (CH_3, CH_2CH_3), 20.4 (2 x CH_3, $CH(CH_3)_2$), 26.0 (CH_2, =CH*C*H$_2$CH=), 48.9 (CH_2, CH_2CH_3), 49.6 (2 x CH, CHN), 50.0 (CH_2, CH_2CH_3), 124.8 (2 x CH, =CH), 128.9 (2 x CH, =CH), 186.4 (C, C(S)S); m/z (ES) 335.1221 ($[M+H]^+$ $C_{15}H_{25}N_2NaOS_2$ requires 335.1228), 394 (17%), 384 (10), 335 (100).

(±)-(1R,2S,6S)-7-Isopropyl-8-oxo-7-azabicyclo[4.2.0]oct-4-en-2-yl diethylcarbamodithioate **52**

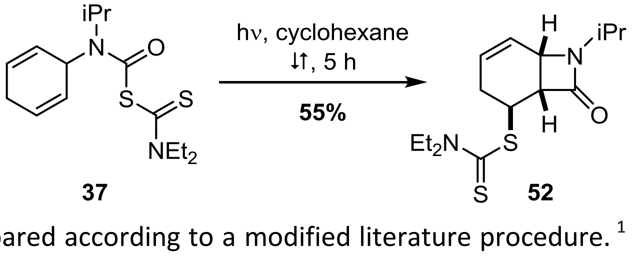

A novel compound prepared according to a modified literature procedure.[1] A solution of carbamoyl dithiocarbamate **37** (1.60 g, 5.10 mmol) in cyclohexane (51 mL) was degassed for 10 min and irradiated with a 500 W lamp which generated enough heat to bring the solvent to reflux. After 5 h,

the solvent was removed under reduced pressure and the crude product obtained was purified by column chromatography (Hexane to Hexane:EtOAc 7:3) to give lactam **52** (0.877 g, 55%) as as a yellow brown oil. R_f 0.56 (Pet ether:Et_2O 7:3); ν_{max} (neat)/cm^{-1}: 2972, 1732, 1486, 1415, 1267, 1205, 1005, 982; δ_H (400 MHz, $CDCl_3$) 1.16 (3H, d, *J* 6.7 Hz, CH_2CH_3), 1.18-1.28 (9H, m, CH_2CH_3 and $CH(CH_3)_2$), 2.50-2.71 (2H, m, $=CHCH_2CHS$), 3.66 (2H, q, *J* 7.0 Hz, CH_2CH_3), 3.70-3.72 (1H, m, CHS), 3.87 (1H, hept, *J* 6.7 Hz, $CH(CH_3)_2$), 3.93-4.07 (3H, m, CH_2CH_3 and CHC(O)), 4.67-4.79 (1H, m, =CHC*H*N), 6.00-6.15 (2H, m, =CH); δ_C (100 MHz, $CDCl_3$) 11.7 (CH_3, CH_2CH_3), 12.7 (CH_3, CH_2CH_3), 20.5 (CH_3, $CH(CH_3)_2$), 22.0 (CH_3, $CH(CH_3)_2$), 28.2 (CH_2, $=CHCH_2CH=$), 44.4 (CH, $CH(CH_3)_2$), 46.5 (2 x CH, CHN, *C*HC(O)), 46.8 (CH_2, CH_2CH_3), 49.3 (CH_2, CH_2CH_3), 53.3 (CH, CHS), 127.6 (CH, =CH), 132.7 (CH, =CH), 167.1 (C, C(O)N), 186.4 (C, C(S)S); m/z (ES) 313.1408 ($[M+H]^+$ $C_{15}H_{25}N_2OS_2$ requires 313.1403), 313 (100%).

N-Allyl-4-methoxyaniline **98**

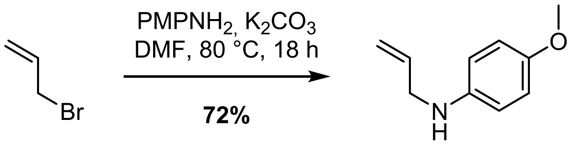

98

A known compound[13] prepared according to a modified literature procedure.[10] A solution of 4-methoxyaniline (3.21 g, 26.0 mmol) and K_2CO_3 (1.89 g, 13.6 mmol) in DMF (28 mL) was treated with allyl bromide (1.50 g, 12.4 mmol). After 18 h at 80 °C, the reaction mixture was quenched with H_2O (30 mL) and extracted with EtOAc (3 x 30 mL). The combined organic extracts were washed with brine (50 mL), dried over $MgSO_4$, filtered, evaporated under reduced pressure and purified by column chromatography (Pet ether to Pet ether:EtOAc 4:1) to give amine **98** (1.45 g, 72%) as a yellow oil. R_f 0.41 (Pet ether:Et_2O 9:1); ν_{max} (neat)/cm^{-1} : 3394, 2934, 1509, 1229, 1034, 816; δ_H (300 MHz, $CDCl_3$) 3.46 (1H, br s, NH), 3.74 (2H, dt, *J* 1.6 and 5.5 Hz, CH_2N), 3.75 (3H, s, OCH_3), 5.15 (1H, dq, *J* 1.6 and 10.3 Hz, CH_2=CH), 5.85 (1H, dq, *J* 1.6 and 17.2 Hz, CH_2=CH), 5.88-6.06 (1H, m, CH_2=C*H*), 6.61 (2H, d, *J* 9.0 Hz, Ar H), 6.79 (2H, d, *J* 9.0 Hz, Ar H); δ_C (100 MHz, $CDCl_3$) 47.7 (CH_2, NCH_2), 56.0 (CH_3, OCH_3), 114.5 (2 x CH, Ar), 115.0 (2 x CH, Ar), 116.3 (CH_2, CH_2=CH), 135.9 (CH, CH_2=*C*H), 142.4 (C, ArCN), 152.4 (C, ArCO); m/z (ES) 165 (13%), 164 (100), 163 (84).

Allyl(4-methoxyphenyl)carbamic chloride **99**

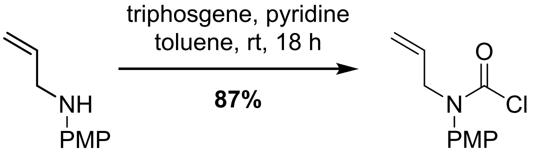

98 **99**

A novel compound prepared according to a modified literature procedure.[10] A solution of triphosgene (0.686 g, 2.31 mmol) in toluene (35 mL) was treated with pyridine (0.607 mL, 7.50 mmol) and subsequently with a solution of amine **98** (1.01 g, 6.25 mmol) in toluene (13 mL). After 18 h at rt, the reaction mixture was quenched with H_2O (40 mL) and extracted with Et_2O (2 x 40 mL). The combined organic extracts were washed sequentially with HCl (40 mL of a 0.25 M aq. solution) and brine (50 mL), dried over $MgSO_4$, filtered, evaporated under reduced pressure and purified by column chromatography (Pet ether to Pet ether:EtOAc 4:1) to give carbamoyl chloride **99** (1.23 g, 87%) as a pale orange oil. R_f 0.49 (Pet ether:EtOAc 9:1); ν_{max} (neat)/cm^{-1} : 2936, 1728, 1510, 1217, 1034, 834, 787; δ_H (300 MHz, $CDCl_3$) (mixture of rotamers) 3.82 (3H, s, OCH_3), 4.14-4.34, 4.39 (2H, m,

CH_2N), 5.09-5.32 (2H, m, CH_2=CH), 5.79-6.01 (1H, m, CH_2=C*H*), 6.90 (2H, d, *J* 8.9 Hz, Ar H), 7.07-7.24 (2H, m, Ar H); δ_C (100 MHz, $CDCl_3$) (mixture of rotamers) 54.3, 55.9 (CH_2, NCH_2), 55.6 (CH_3, OCH_3), 114.5, 114.7 (2 x CH, Ar), 119.2, 119.7 (CH_2, *C*H_2=CH), 128.4, 128.6 (2 x CH, Ar), 131.4, 131.9 (CH, CH_2=*C*H), 132.7, 134.8 (C, ArCN), 148.4, 149.7 (C, ArCO), 159.0, 159.6 (C, NC(O)Cl); m/z (ES) 248.0447 ($[M+H]^+$ $C_{11}H_{12}{}^{35}ClNNaO_2$ requires 248.0449), 250 (31%), 248 (100).

Diethylthiocarbamic acid-[allyl(4-methoxyphenyl)carbamic acid]thioanhydride **38**

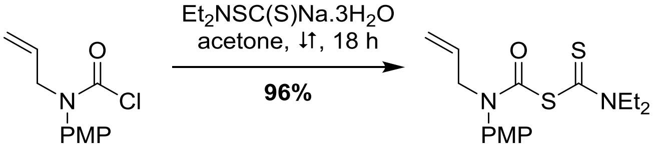

99 **38**

A novel compound prepared according to a modified literature procedure. [10] A solution of carbamoyl chloride **99** (1.06 g, 4.68 mmol) in acetone (36 mL) was treated with sodium diethyldithiocarbamate trihydrate (3.17 g, 14.1 mmol). The solution was heated at reflux for 18 h, quenched with H_2O (30 mL). Acetone was removed under reduced pressure. The aqueous layer was extracted with Et_2O (3 x 30 mL). The combined organic extracts were washed with brine (50 mL), dried over $MgSO_4$, filtered, evaporated under reduced pressure and purified by column chromatography (Pet ether to Pet ether:EtOAc 4:1) to give dithiocarbamate **38** (1.52 g, 96%) as a bright yellow oil. R_f 0.27 (Pet ether:EtOAc 4:1); ν_{max} (neat)/cm^{-1} : 2974, 1668, 1509, 1419, 1245, 1196, 918, 833, 730; δ_H (400 MHz, $CDCl_3$) 1.23 (3H, t, *J* 7.1 Hz, CH_2CH_3), 1.32 (3H, t, *J* 7.1 Hz, CH_2CH_3), 3.78 (2H, q, *J* 7.1 Hz, CH_2CH_3), 3.82 (3H, s, OCH_3), 4.00 (2H, q, *J* 7.1 Hz, CH_2CH_3), 4.27 (2H, d, *J* 6.4 Hz, NCH_2CH=), 4.99-5.21 (2H, m, CH_2=CH), 5.85 (1H, ddt, *J* 6.4, 10.2 and 16.7 Hz, CH_2=C*H*), 6.88 (2H, d, *J* 8.9 Hz, Ar H), 7.18 (2H, d, *J* 8.9 Hz, Ar H); δ_C (100 MHz, $CDCl_3$) 11.2 (CH_3, CH_2CH_3), 13.7 (CH_3, CH_2CH_3), 48.9 (CH_2, CH_2CH_3), 50.5 (CH_2, CH_2CH_3), 54.1 (CH_2, NCH_2CH=), 55.7 (CH_3, OCH_3), 114.8 (2 x CH, Ar), 119.1 (CH_2, *C*H_2=CH), 130.8 (2 x CH, Ar), 132.4 (CH, CH_2=*C*H), 133.6 (C, ArCN), 153.8 (C, ArCO), 160.1 (C, C(O)N), 185.3 (C, C(S)S); m/z (ES) 339.1197 ($[M+H]^+$ $C_{16}H_{23}N_2O_2S_2$ requires 339.1195), 341 (9%), 340 (18), 339 (100).

1-(4-Methoxyphenyl)-2-oxoazetidin-3-yl)methyl diethylcarbamodithioate **53**

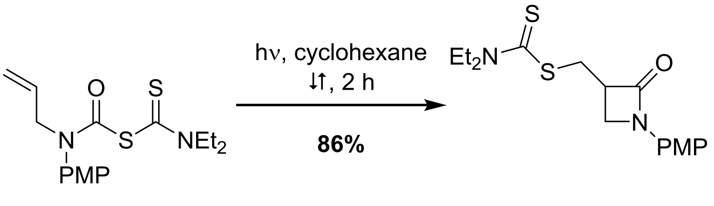

38 **53**

A novel compound prepared according to a modified literature procedure.[1] A solution of dithiocarbamate **38** (0.961 g, 2.84 mmol) in cyclohexane (28 mL) was degassed for 10 min and irradiated with a 500 W lamp which generated enough heat to bring the solution to reflux. After 2 h, the solvent was removed under reduced pressure and the crude product obtained was purified by column chromatography (Pet ether to Pet ether:EtOAc 4:1) to give lactam **53** (0.823 g, 86%) as a dark orange oil. R_f 0.27 (Pet ether:EtOAc 4:1); ν_{max} (neat)/cm^{-1} : 2974, 1735, 1510, 1241, 1143, 826; δ_H (400 MHz, $CDCl_3$) 1.23-1.29 (6H, m, CH_2CH_3), 3.50 (1H, dd, *J* 1.9 and 5.7 Hz, SCH_2CH), 3.68-4.16 (11H, m, CH_2CH_3, NCH_2CH, SCH_2CH, $CH_2CHC(O)$), 6.86 (2H, d, *J* 9.0 Hz, Ar H), 7.29 (2H, d, *J* 9.0 Hz, Ar H); δ_C (100 MHz, $CDCl_3$) 11.7 (CH_3, CH_2CH_3), 12.7 (CH_3, CH_2CH_3), 35.2 (CH_2), 44.0 (CH_2), 47.0 (CH_2), 48.3 (CH,

*C*HC(O)), 50.1 (CH_2), 55.7 (CH_3, OCH_3), 114.5 (2 x CH, Ar), 117.8 (2 x CH, Ar), 132.0 (C, ArCN), 156.3 (C, ArCO), 165.0 (C, C(O)N), 194.7 (C, C(S)S); m/z (ES) 339.1196 ($[M+H]^+$ $C_{16}H_{23}N_2O_2S_2$ requires 339.1195), 341 (8%), 340 (17), 339 (100).

N-Benzyl-3,5,5-trimethylcyclohex-2-enamine **100**

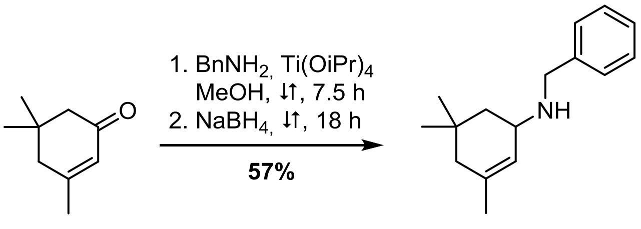

isophorone **100**

A known compound prepared according to a modified literature procedure.[14] A solution of benzylamine (14.2 mL, 130 mmol) in MeOH (100 mL) was treated with $Ti(O\textit{i}Pr)_4$ (4.51 mL, 15.2 mmol) and isophorone (2.17 mL, 14.5 mmol). After 7.5 h at reflux, the reaction mixture was cooled to rt, treated with $NaBH_4$ (2.19 g, 58.0 mmol) portionwise over 10 min and heated to reflux. After 18 h, the excess of $NaBH_4$ was carefully quenched with H_2O (40 mL) and the mixture was extracted with EtOAc (2 x 100 mL). The combined organic extracts were washed with NaOH (50 mL of a 5% aq. solution) and brine (50 mL), dried over $MgSO_4$, filtered, evaporated under reduced pressure and purified by column chromatography (Pet ether:EtOAc 9:1 to Pet ether:EtOAc 4:1) to give amine **100** (1.90 g, 57%) as a light yellow oil. R_f 0.70 (CH_2Cl_2:EtOH 9:1); ν_{max} (neat)/cm^{-1} : 2949, 2865, 1453, 1363, 1101, 729, 696; δ_H (400 MHz, $CDCl_3$) 0.93 (3H, s, $C(CH_3)_2$), 1.05 (3H, s, $C(CH_3)_2$), 1.15-1.20 (1H, m, CH_2), 1.45 (1H, br s, NH), 1.65, 1.92 (2H, ABq, *J* 17.2 Hz, CH_2), 1.73 (3H, s, $=CCH_3$), 1.77 (1H, dd, *J* 5.6 and 12.4 Hz, CH_2), 3.26-3.38 (1H, m, NC*H*CH_2), 3.91 (2H, s, CH_2Ph), 5.50 (1H, br s, =CH), 7.27-7.42 (5H, m, Ar H); δ_C (100 MHz, $CDCl_3$) 23.9 (CH_3, C(*C*$H_3)_2$), 25.9 (CH_3, C(*C*$H_3)_2$), 30.8 (C, *C*$(CH_3)_2$), 31.0 (CH_3, =C*C*H_3), 43.4 (CH_2), 44.5 (CH_2), 51.2 (CH_2, NH*C*H_2Ph), 52.6 (CH, CH_2*C*HN), 122.3 (CH, =CH), 127.0 (CH, Ar), 128.4 (2 x CH, Ar), 128.5 (2 x CH, Ar), 134.8 (C, =*C*CH_3), 141.0 (C, Ar); m/z (ES) 229.1826 (M^+ $C_{16}H_{23}N$ requires 229.1830), 229 (M^+, 35%), 214 (100), 173 (21), 158 (43), 107 (15), 91 (28).

Benzyl(3,5,5-trimethylcyclohex-2-en-1-yl)carbamic chloride **101**

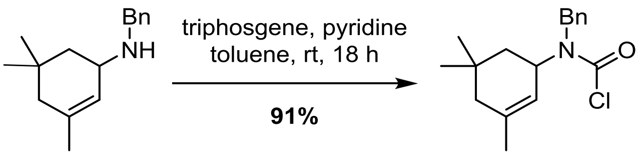

100 **101**

A novel compound prepared according to a literature procedure. [10] A solution of triphosgene (0.907 g, 3.06 mmol) in toluene (45 mL) was treated with pyridine (0.800 mL, 9.91 mmol) and subsequently with a solution of amine **100** (1.90 g, 8.26 mmol) in toluene (10 mL). The reaction mixture was stirred for 18 h at rt, quenched with H_2O (30 mL) and extracted with Et_2O (2 x 50 mL). The combined organic extracts were washed sequentially with HCl (50 mL of a 0.25 M aq. solution), H_2O (50 mL) and brine (50 mL), dried over $MgSO_4$, filtered and evaporated under reduced pressure to give carbamoyl chloride **101** (2.19 g, 91%) as a yellow oil. R_f 0.73 (Pet ether:EtOAc 95:5); ν_{max} (neat)/cm^{-1} : 2953, 1727, 1672, 1454, 1229, 1142, 969, 715, 694; δ_H (400 MHz, $CDCl_3$) 0.95 (6H, t, *J* 12.0 Hz, $C(CH_3)_2$), 1.28 (1H, q, *J* 11.4 Hz, CH_2), 1.54-1.73 (5H, m, $=CCH_3$ and CH_2), 1.81-1.96 (1H, m, CH_2), 4.38-4.71 (2H, m, CH_2Ph), 4.86-5.08 (1H, m, NC*H*CH_2), 5.14-5.27 (1H, m, =CH), 7.22-7.46 (5H, m, Ar H); δ_C (100 MHz, $CDCl_3$) (as a mixture of rotamers) 23.9 (CH_3, =C*C*H_3), 25.1, 25.2 (CH_3, C(*C*$H_3)_2$), 31.2, 31.3 (C, *C*$(CH_3)_2$),

31.8 (CH_3, C(CH_3)$_2$), 39.3, 40.1 (CH_2), 43.8 (CH_2), 49.7, 51.1 (CH_2, NCH_2Ph), 57.0, 58.6 (CH, CH_2*C*HN), 119.2 (CH, =CH), 126.4 (CH, Ar), 127.3, 127.4 (2 x CH, Ar), 128.6, 128.7 (2 x CH, Ar), 137.7, 137.8 (C, =*C*CH_3), 138.9, 139.0 (C, Ar), 150.1, 150.4 (C, NC(O)Cl); m/z (ES) 314.1282 ($[M+Na]^+$ $C_{17}H_{22}{}^{35}ClNNaO$requires 314.1282), 314 (4%), 282 (8), 230 (100).

Diethyldithiocarbamic acid-[benzyl(3,5,5-trimethylcyclohex-2-en-1-yl)carbamic acid]-thioanhydride **39**

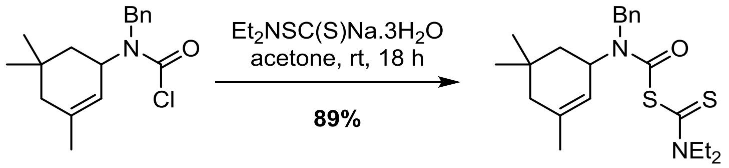

A novel compound prepared according to a literature procedure. [10] A solution of carbamoyl chloride **101** (2.19 g, 7.53 mmol) in acetone (47 mL) was treated with sodium diethyldithiocarbamate trihydrate (6.79 g, 30.1 mmol). The reaction mixture was stirred for 18 h at rt, quenched with H_2O (40 mL) and extracted with Et_2O (3 x 40 mL). The combined organic extracts were washed with brine (100 mL), dried over $MgSO_4$, filtered, evaporated under reduced pressure and purified by column chromatography (Pet ether to Pet ether:EtOAc 95:5) to give dithiocarbamate **39** (2.70 g, 89%) as a yellow oil. R_f 0.57 (Pet ether:EtOAc 85:15); ν_{max} (neat)/cm^{-1} : 2935, 1659, 1488, 1418, 1270, 1143, 962, 718, 695; δ_H (400 MHz, $CDCl_3$) (as a mixture of rotamers) 0.93 (3H, s, C(CH_3)$_2$), 0.95 (3H, s, C(CH_3)$_2$), 1.11-1.40 (8H, m, CH_2C*H$_3$* and CH_2), 1.53-1.77 (4H, m, =CCH_3 and CH_2), 1.86 (1H, d, *J* 17.3 Hz, CH_2), 3.62-4.15 (4H, m, C*H$_2$*CH_3 and NCH_2Ph), 4.48-4.66 (3H, m, C*H$_2$*CH_3 and CHN), 5.22 (1H, br s, =CH), 7.29 (5H, br s, Ar H); δ_C (100 MHz, $CDCl_3$) (as a mixture of rotamers) 11.1 (CH_3, CH_2*C*H_3), 13.3 (CH_3, CH_2*C*H_3), 23.6 (CH_3, =C*C*H_3), 24.8 (CH_3, C(*C*H_3)$_2$), 30.9 (C, *C*(CH_3)$_2$), 31.6 (CH_3, C(*C*H_3)$_2$), 39.2, 40.3 (CH_2), 43.5 (CH_2), 48.7 (CH_2, NCH_2Ph), 49.9 (CH_2, *C*H_2CH_3), 50.3 (CH_2, *C*H_2CH_3), 54.4, 56.5 (CH, CH_2*C*HN), 119.5 (CH, =CH), 126.1 (CH, Ar), 126.9, 127.2 (2 x CH, Ar), 128.3 (2 x CH, Ar), 138.3 (C, Ar), 138.4 (C, CH=*C*CH_3), 162.9, 163.1 (C, C(O)N), 184.5, 185.3 (C, C(S)S); m/z (ES) 427.1847 ($[M+Na]^+$ $C_{22}H_{32}N_2NaOS_2$ requires 427.1854), 443 (22%), 427 (100).

(±)-(1*R*,6*S*)-7-(4-Methoxybenzyl)-7-azabicyclo[4.2.0]oct-2-en-8-one **58a**

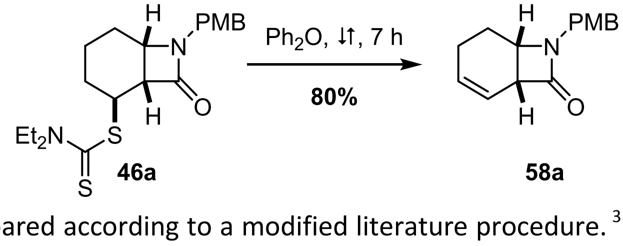

A novel compound prepared according to a modified literature procedure. [3] A solution of lactam **46a** (3.10 g, 7.89 mmol) in diphenyl ether (61 mL) was heated to reflux. After 7 h, the reaction mixture was directly purified by column chromatography (Pet ether to Pet ether:EtOAc 3:1) to give lactam **58a** (1.54 g, 80%) as a pale yellow oil. R_f 0.22 (Pet Ether: EtOAc 7:3); ν_{max} (neat)/cm^{-1} : 2930, 1734, 1611, 1511, 1241, 1175, 1030, 831, 693; δ_H (400 MHz, $CDCl_3$) 1.21-1.34 (1H, m, CH_2), 1.79-1.87 (2H, m, CH_2), 1.94-2.01 (1H, m, CH_2), 3.62 (1H, t, *J* 5.4 Hz, CHC*H*C(O)), 3.80-3.84 (4H, m, CH_2C*H*N and OCH_3), 4.17, 4.45 (2H, ABq, *J* 14.8 Hz, NCH_2Ar), 5.80-5.85 (1H, m, CH_2C*H*=CH), 5.95-6.00 (1H, m, CHC*H*=CH), 6.87 (2H, d, *J* 8.4 Hz, Ar H), 7.21 (2H, d, *J* 8.4 Hz, Ar H); δ_C (100 MHz, $CDCl_3$) 19.7 (CH_2), 22.8 (CH_2), 43.6 (CH_2, NCH_2Ar), 48.6 (CH, =CH*C*HC(O)), 51.1 (CH, CH_2*C*HN), 55.5 (CH_3, OCH_3), 114.3 (2 x

CH, Ar), 121.7 (CH, CH_2*C*H=CH), 128.5 (C, Ar*C*CH_2N), 129.9 (2 x CH, Ar), 131.5 (CH, CH=*C*HCH), 159.3 (C, ArCO), 167.2 (C, C(O)N); m/z (ES) 266.1153 ($[M+Na]^+$ $C_{15}H_{17}NNaO_2$ requires 266.1157), 267 (10%), 266 (100).

(±)-(1*R*,2*S*,5*S*)-6-(4-Methoxyphenyl)-1-methyl-7-oxo-6-azabicyclo[3.2.0]heptan-2-yl diethylcarbamodithioate **59**

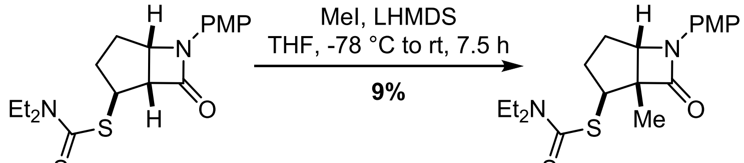

A novel compound prepared according to a modified literature procedure.[15] A solution of lactam **47** (99 mg, 0.27 mmol) in THF (2.7 mL) was cooled to -78 °C and treated with methyl iodide (85 µL, 1.4 mmol) and LHMDS (1.4 mL of a 1.0 M solution in THF, 1.4 mmol). After 3 h at -78 °C, the reaction mixture was allowed to warm to rt. After 4.5 h at rt, the solvent was removed under reduced pressure and the crude product obtained was purified by column chromatography (Pet ether to Pet ether:EtOAc 4:1) to give methyl lactam **59** (9 mg, 9%) as a colourless oil. R_f 0.27 (Pet ether:EtOAc 4:1); δ_H (400 MHz, $CDCl_3$) 1.23-1.33 (6H, m, 2 x CH_2*CH_3*), 1.53 (3H, s, CH_3), 1.74-1.86 (1H, m, CH_2), 2.06-2.21 (2H, m, CH_2), 2.30-2.42 (1H, m, CH_2), 3.75 (2H, q, *J* 6.9 Hz, *CH_2*CH_3), 3.79 (3H, s, OCH_3), 4.02 (2H, q, *J* 6.9 Hz, *CH_2*CH_3), 4.08 (1H, d, *J* 4.4 Hz, CH_2C*H*S), 4.78 (1H, d, *J* 5.6 Hz, CH_2C*H*N), 6.88 (2H, d, *J* 9.0 Hz, Ar H), 7.35 (2H, d, *J* 9.0 Hz, Ar H); δ_C (100 MHz, $CDCl_3$) 11.8 (CH_3, CH_2*CH_3*), 12.8 (CH_3, CH_2*CH_3*), 15.2 (CH_3, C*CH_3*), 26.1 (CH_2), 33.0 (CH_2), 46.9 (CH_2, *C*H_2CH_3), 49.6 (CH_2, *C*H_2CH_3), 53.9 (CH, CH_2*C*HN), 55.7 (CH_3, OCH_3), 63.9 (CH, CH_2*C*HS), 64.1 (C, CH*C*(CH_3)C(O)), 114.7 (2 x CH, Ar), 118.4 (2 x CH, Ar), 131.0 (C, ArCN), 156.2 (C, ArCO), 167.3 (C, C(O)N), 194.1 (C, SC(S)); m/z (ES) 379.1507 ($[M+H]^+$ $C_{19}H_{27}N_2O_2S_2$ requires 379.1508), 379 (27), 366 (21), 365 (100).

(±)-(1*R*,5*S*)-8-(4-Methoxyphenyl)-6-azabicyclo[3.2.0]hept-2-en-7-one **60**

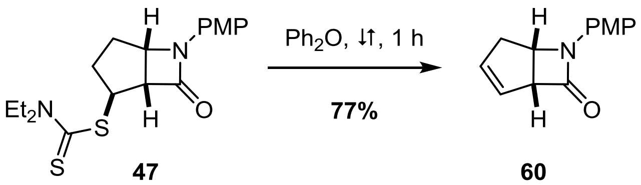

A novel compound prepared according to a modified literature procedure. [3] A solution of lactam **47** (73 mg, 0.20 mmol) in diphenyl ether (2.0 mL) was heated to reflux. After 1 h, the reaction mixture was directly purified by column chromatography (Pet ether to Pet ether:EtOAc 7:3) to give lactam **60** (33 mg, 77%) as a green solid. mp 89-91 °C (CH_2Cl_2); R_f 0.54 (Pet ether:EtOAc 7:3); ν_{max} (neat)/cm^{-1}: 2937, 1732, 1511, 1297, 1231, 1031, 826, 806, 691; δ_H (400 MHz, $CDCl_3$) 2.51-2.58 (2H, m, CH_2), 3.77 (3H, s, OCH_3), 4.15-4.24 (1H, m, CH_2C*H*N), 4.55 (1H, t, *J* 5.4 Hz, CHC*H*C(O)), 5.85-5.87 (1H, m, CH_2C*H*=CH), 5.92-5.95 (1H, m, CH=C*H*C(O)), 6.85 (2H, d, *J* 9.2 Hz, Ar H), 7.29 (2H, d, *J* 9.2 Hz, Ar H); δ_C (100 MHz, $CDCl_3$) 33.7 (CH_2), 54.3 (CH, =CH*C*HC(O)), 55.6 (CH_3, OCH_3), 62.7 (CH, CH_2*C*HN), 114.6 (2 x CH, Ar), 118.0 (2 x CH, Ar), 126.4 (CH, CH_2*C*H=CH), 130.9 (C, ArCN), 133.3 (C, HC=*C*HCHC(O)), 156.0 (C, ArCO), 167.1 (C, C(O)N); m/z (EI) 215.0943 (M^+ $C_{13}H_{13}NO_2$ requires 215.0946), 215 (8%), 149 (100), 134 (25).

(±)-(1*R*,7*S*)-8-(4-Methoxyphenyl)-8-azabicyclo[5.2.0]non-2-en-9-one **62**

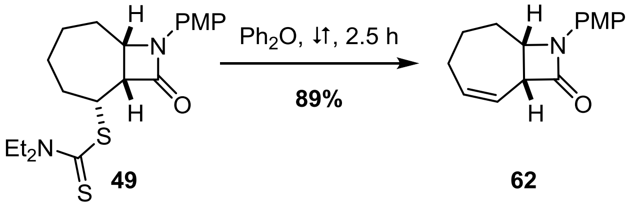

A novel compound prepared according to a literature procedure.[3] A solution of lactam **49** (0.106 g, 0.270 mmol) in diphenyl ether (2.7 mL) was heated to reflux. After 2.5 h, the reaction mixture was directly purified by column chromatography (Pet ether to Pet ether:EtOAc 7:3) to give lactam **62** (59 mg, 89%) as a light green solid. mp 94-96 °C; R_f 0.54 (Pet ether:EtOAc 7:3); ν_{max} (neat)/cm^{-1}: 2939, 1723, 1510, 1382, 1243, 1030, 825; δ_H (400 MHz, $CDCl_3$) 1.50-1.80 (3H, m, CH_2), 2.15-2.29 (3H, m, CH_2), 3.76 (3H, s, OCH_3), 4.05-4.08 (1H, m, =CHC*H*C(O)), 4.15-4.20 (1H, m, CH_2C*H*N), 5.64-5.72 (2H, m, CH=CH), 6.85 (2H, d, *J* 9.0 Hz, Ar H), 7.30 (2H, d, *J* 9.0 Hz, Ar H); δ_C (100 MHz, $CDCl_3$) 19.2 (CH_2), 27.2 (CH_2), 28.2 (CH_2), 52.7 (CH, =CH*C*HC(O)), 55.2 (CH, CH_2*C*HN), 55.6 (CH_3, OCH_3), 114.5 (2 x CH, Ar), 118.5 (2 x CH, Ar), 121.0 (CH, CH_2*C*H=CH), 128.9 (CH, CH=*C*HCH), 131.2 (C, ArCN), 156.0 (C, ArCO), 165.5 (C, C(O)N); m/z (ES) 266.1156 ([M+Na]$^+$ $C_{15}H_{17}NNaO_2$ requires 266.1157), 415 (17%), 298 (10), 267 (5), 266 (100).

(±)-(1*S*,2*R*,7*R*)-3-(4-Methoxyphenyl)-3-azatricyclo[5.2.1.0^{2,5}]decan-5-en-4-one **63**

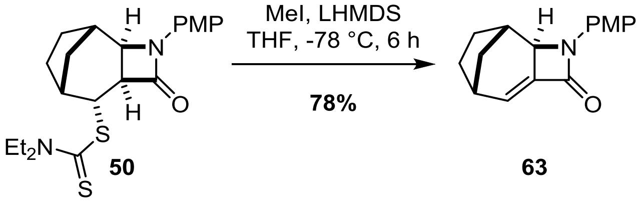

A novel compound prepared according to a modified literature procedure.[15] A solution of lactam **50** (0.139 g, 0.344 mmol) in THF (3.4 mL) was cooled to -78 °C and treated with methyl iodide (32 µL, 0.52 mmol). The reaction mixture was stirred at -78 °C for 30 min, LHMDS (0.52 mL of a 1.0 M solution in THF, 0.52 mmol) was added over 2 min. After 5.5 h at -78 °C, the solvent was removed under reduced pressure and the crude product obtained was purified by column chromatography (Pet ether to Pet ether:EtOAc 9:1) to give lactam **63** (69 mg, 78%) as a white solid. mp 120-121 °C; R_f 0.69 (Pet ether:EtOAc 7:3); ν_{max} (neat)/cm^{-1}: 2942, 1738, 1507, 1370, 1235, 833; δ_H (400 MHz, $CDCl_3$) 1.42 (1H, d, *J* 11.6 Hz, CH_2), 1.51 (1H, ddd, *J* 3.1, 4.4 and 11.6 Hz, CH_2), 1.60-1.75 (2H, m, CH_2), 1.75-1.88 (1H, m, CH_2), 1.94-2.07 (1H, m, CH_2), 2.72-2.82 (2H, m, CH_2C*H*CH= and CH_2C*H*CHN), 3.78 (3H, s, OCH_3), 4.18 (1H, d, *J* 1.4 Hz, CHC*H*N), 6.87 (2H, d, *J* 9.0 Hz, Ar H), 6.95 (1H, dd, *J* 1.4 and 7.5 Hz, CHC*H*=C), 7.31 (2H, d, *J* 9.0 Hz, Ar H); δ_C (100 MHz, $CDCl_3$) 30.1 (CH_2), 31.8 (CH_2), 34.2 (CH), 34.6 (CH), 35.8 (CH_2), 55.7 (CH_3, OCH_3), 65.5 (CH, CH*C*HN), 114.7 (2 x CH, Ar), 117.4 (2 x CH, Ar), 132.9 (C, ArCN), 137.2 (C, =C), 140.4 (CH, =CH), 155.8 (C, ArCO), 162.1 (C, C(O)N); m/z (ES) 278.1154 ([M+Na]$^+$, $C_{16}H_{17}NNaO_2$ requires 278.1154), 279 (12%), 278 (100).

(±)-(1*S*,2*R*,5*S*,6*S*,7*R*)-3-(4-Methoxyphenyl)-5-methyl-4-oxo-3-azatricyclo[5.2.1.0^{2,5}] decan-6-yl diethyldithiocarbamate **64**

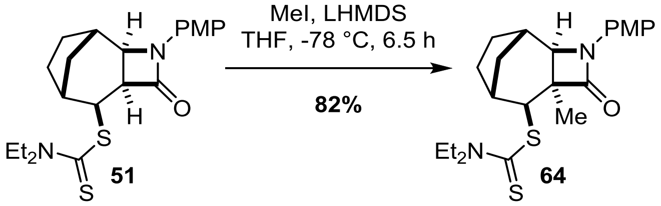

A novel compound prepared according to a modified literature procedure.[15] A solution of lactam **51** (64 mg, 0.16 mmol) in THF (1.6 mL) was cooled to -78 °C, treated with methyl iodide (4.9 µL, 0.79 mmol) and LHMDS (0.79 mL of a 1.0 M solution in THF, 0.79 mmol). After 6.5 h at -78 °C, the solvent was removed under reduced pressure and the crude product obtained was purified by column chromatography (Pet ether to Pet ether:EtOAc 7:3) to give lactam **64** (54 mg, 82%) as a yellow solid. mp 150-151 °C; R_f 0.39 (Pet ether:EtOAc 4:1); ν_{max} (neat)/cm^{-1}: 2931, 1733, 1514, 1487, 1250, 829; δ_H (400 MHz, $CDCl_3$) 1.17-1.37 (7H, m, CH_2 and CH_2CH_3), 1.53 (3H, s, $CH_3CC(O)$), 1.55-1.61 (1H, m, CH_2), 1.76-2.01 (4H, m, CH_2), 2.45 (1H, t, *J* 5.9 Hz, CH_2CHCHN), 2.71 (1H, t, *J* 5.2 Hz, CH_2CHCHS), 3.76 (1H, s, CHC*H*S), 3.78 (3H, s, OCH_3), 3.76-4.10 (4H, m, CH_2CH_3), 4.12 (1H, s, CHC*H*N), 6.87 (2H, d, *J* 8.9 Hz, Ar H), 7.35 (2H, d, *J* 8.9 Hz, Ar H); δ_C (100 MHz, $CDCl_3$) 11.8 (CH_3, CH_2CH_3), 12.8 (CH_3, CH_2CH_3), 24.6 (CH_3, $CH_3CC(O)$), 26.5 (CH_2), 26.7 (CH_2), 31.6 (CH_2), 35.1 (CH, CH_2CHCHS), 42.7 (CH, CH_2CHCHN), 47.0 (CH_2, CH_2CH_3), 49.6 (CH_2, CH_2CH_3), 51.1 (C, $CH_3CC(O)$), 55.7 (CH_3, OCH_3), 59.1 (CH, CH*C*HN), 65.5 (CH, CH*C*HS), 114.6 (2 x CH, Ar), 118.9 (2 x CH, Ar), 130.9 (C, ArCN), 156.2 (C, ArCO), 168.2 (C, C(O)N), 195.8 (C, C(S)S); m/z (ES) 419.1823 ([M+H]$^+$, $C_{22}H_{31}N_2O_2S_2$ requires 419.1821), 854 (18%), 441 (12), 419 (100).

N-Isopropylbenzamide **65**

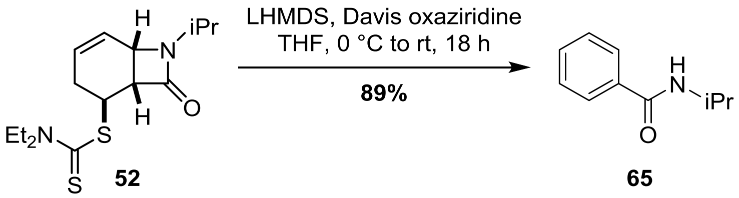

A known compound[16] prepared according to a modified literature procedure.[17] A solution of β-lactam **52** (35 mg, 0.11 mmol) in THF (1.1 mL) was cooled to 0 °C and LHMDS (0.17 mL of a 1.0 M solution in THF, 0.17 mmol) was added dropwise over 1 min. After 1 h at 0 °C, Davis oxaziridine (44 mg, 0.17 mmol) was added. The reaction mixture was allowed to warm to rt and after 18 h, it was evaporated under reduced pressure. The crude material obtained was purified by column chromatography (Pet ether to Pet ether:EtOAc 3:2) to give amide **65** (16 mg, 89%) as a white solid. mp 101-103 °C; R_f 0.58 (Pet ether:EtOAc 65:35); ν_{max} (neat)/cm^{-1}: 3295, 2971, 1630, 1531, 1346, 1288, 1168, 1137, 692; δ_H (400 MHz, $CDCl_3$) 1.25 (6H, d, *J* 6.6 Hz, $CH(CH_3)_2$), 4.18-4.73 (1H, m, $CH(CH_3)_2$), 6.04 (1H, br s, NH), 7.36-7.43 (2H, m, Ar H), 7.44-7.49 (1H, m, Ar H), 7.71-7.77 (2H, m, Ar H); δ_C (100 MHz, $CDCl_3$) 23.0 (2 x CH_3, $CH(CH_3)_2$), 42.0 (CH, $CH(CH_3)_2$), 127.0 (2 x CH, Ar), 128.6 (2 x CH, Ar), 131.4 (CH, Ar), 135.1 (C, Ar), 166.9 (C, C(O)N).

1,1-Diethyl-3-isopropylurea **66**

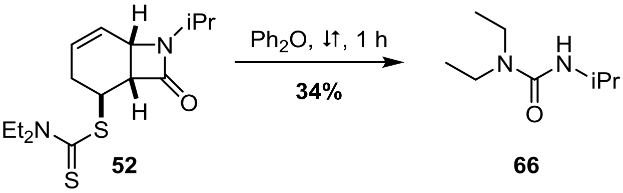

A novel compound prepared according to a modified literature procedure. [3] A solution of lactam **52** (86 mg, 0.28 mmol) in Ph_2O (2.8 mL) was heated to reflux. After 1 h, the reaction mixture was directly purified by column chromatography (Pet ether to Pet ether:EtOAc 85:15) to give urea **66** (15 mg, 34%) as a white crystalline solid. mp 54-57 °C; R_f 0.18 (Pet ether:EtOAc 7:3); ν_{max} (neat)/cm^{-1}: 3390, 2972, 1609, 1529, 1491, 1268, 1200, 1079, 765; δ_H (400 MHz, $CDCl_3$) 1.06-1.17 (12H, m, CH_2CH_3 and $CH(CH_3)_2$), 3.22 (4H, q, *J* 7.1 Hz, CH_2CH_3), 3.98 (1H, hept, *J* 6.7 Hz, $CH(CH_3)_2$), 4.04 (1H, br s, NH); δ_C (100 MHz, $CDCl_3$) 14.0 (2 x CH_3, CH_2CH_3), 23.8 (2 x CH_3, $CH(CH_3)_2$), 41.2 (2 x CH_2, CH_2CH_3), 42.5 (CH, $CH(CH_3)_2$), 156.8 (C, C(O)N); m/z (EI) 158.1412 (M^+ $C_8H_{18}N_2O$ requires 158.1419), 158 (73%), 100 (90), 72 (39), 70 (47), 58 (100).

1-(4-Methoxyphenyl)-3-methyleneazetidin-2-one **67**

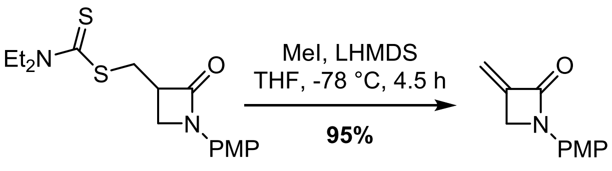

A known compound[18] prepared according to a modified literature procedure.[15] A solution of lactam **53** (0.312 g, 0.922 mmol) in THF (9.2 mL) was cooled to -78 °C and treated with methyl iodide (86 μL, 1.4 mmol) and LHMDS (1.4 mL of a 1.0 M solution in THF, 1.4 mmol). After 4.5 h at -78 °C, the solvent was removed under reduced pressure and the crude product obtained was purified by column chromatography (Pet ether to Pet ether:EtOAc 7:3) to give lactam **67** (0.173 g, 95% by NMR analysis) as a pale yellow solid. mp 87-90 °C; R_f 0.64 (Pet ether:EtOAc 1:1); ν_{max} (neat)/cm^{-1} : 2951, 1720, 1510, 1383, 1239, 1144, 1030, 933, 824; δ_H (300 MHz, $CDCl_3$) 3.79 (3H, s, OCH_3), 4.09 (2H, t, *J* 1.4 Hz, CH_2N), 5.31 (1H, dd, *J* 1.4 and 3.2 Hz, CH_2=C), 5.83 (1H, dd, *J* 1.4 and 3.2 Hz, CH_2=C), 6.89 (2H, d, *J* 9.0 Hz, Ar H), 7.34 (2H, d, *J* 9.0 Hz, Ar H); δ_C (100 MHz, $CDCl_3$) 48.0 (CH_2, NCH_2), 55.7 (CH_3, OCH_3), 110.7 (CH_2, CH_2=C), 114.6 (2 x CH, Ar), 117.8 (2 x CH, Ar), 132.1 (C, CH_2=*C*), 143.7 (C, ArCN), 156.4 (C, ArCO), 159.9 (C, C(O)N); m/z (ES) 190.0862 ($[M+H]^+$ $C_{11}H_{12}NO_2$ requires 190.0863), 190 (100%).

(±)-(3a*R*,5a*S*,8a*S*)-5-Benzylhexahydro-4*H*-[1,3]dioxolo[4',5':2,3]benzo[1,2-*b*]azete-2,4-dione **92**

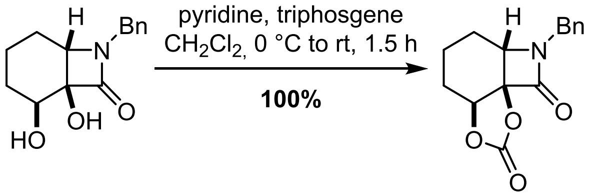

A novel compound prepared according to a literature procedure.[19] A solution of diol **73a** (81 mg, 0.33 mmol) in CH_2Cl_2 (1.6 mL) at 0 °C was treated with pyridine (132 μL, 1.64 mmol) and triphosgene (79 mg, 0.27 mmol). The reaction mixture was allowed to warm to rt after 40 min at 0 °C, stirred at rt for

50 min, quenched with NH_4Cl (5 mL of a saturated aq. solution) and extracted with CH_2Cl_2 (3 x 5 mL). The combined organic extracts were washed sequentially with HCl (10 mL of a 1 M aq. solution) and brine (15 mL), dried over $MgSO_4$, filtered, evaporated under reduced pressure and purified by column chromatography (Pet ether to Pet ether:EtOAc 1:1) to give cyclic carbonate **92** (90 mg, 100%) as a white solid. mp 100-101 °C; R_f 0.52 (Pet ether:EtOAc 1:1); ν_{max} (neat)/cm^{-1}: 2955, 1812, 1766, 1362, 1165, 1067, 732; δ_H (400 MHz, $CDCl_3$) 1.19-1.38 (1H, m, CH_2), 1.45-1.84 (4H, m, CH_2), 2.20-2.35 (1H, m, CH_2), 3.92 (1H, dd, *J* 2.0 and 5.6 Hz, CH_2C*H*N), 4.15, 4.65 (2H, ABq, *J* 15.2 Hz, NCH_2Ph), 4.97 (1H, dd, *J* 7.0 and 8.7 Hz, CH_2C*H*O), 7.21-7.42 (5H, m, Ar H); δ_C (100 MHz, $CDCl_3$) 14.7 (CH_2), 24.2 (CH_2), 28.1 (CH_2), 44.4 (CH_2, NCH_2Ph), 57.9 (CH, CH_2*C*HN), 74.4 (CH, CH_2*C*HO), 85.0 (C, CH*C*OC(O)), 128.3 (2 x CH, Ar), 128.4 (CH, Ar), 129.2 (2 x CH, Ar), 134.2 (C, Ar), 152.6 (C, OC(O)O), 164.1 (C, C(O)N); m/z (ES) 274.1074 ($[M+H]^+$ $C_{15}H_{16}NO_4$ requires 274.1074), 292 (16%), 291 (100), 274 (51).

(±)-(1*S*,2*R*,4*S*,7*S*)-8-(4-Methoxyphenyl)-3-oxa-8-azatricyclo[5.2.0.0^{2,4}]nonan-9-one **70**

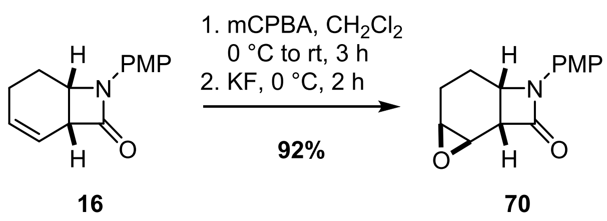

A novel compound prepared according to a modified literature procedure. [5] A solution of lactam **16** (0.158 g, 0.690 mmol) in CH_2Cl_2 (4.6 mL) was cooled to 0 °C, treated with recrystallised *m*CPBA (0.357 g, 2.07 mmol) and allowed to warm to rt over 1 h. After 2 h at rt, the reaction mixture was cooled to 0 °C, diluted with CH_2Cl_2 (10 mL) and treated with KF (0.361 g, 6.21 mmol). After stirring at 0 °C for 2 h, the reaction mixture was filtered through a pad of celite (eluent CH_2Cl_2), the filtrate was evaporated under reduced pressure and purified by column chromatography (Pet ether to Pet ether:EtOAc 7:3) to give epoxy lactam **70** (0.156 g, 92%) as a yellow solid. mp 98-100 °C (CH_2Cl_2); R_f 0.43 (Pet ether:EtOAc 3:1); ν_{max} (neat)/cm^{-1} : 2955, 1725, 1513, 1398, 1243, 1226, 1028, 828; δ_H (400 MHz, $CDCl_3$) 1.90-2.02 (4H, m, CH_2), 3.29 (1H, t, *J* 3.6 Hz, CH_2C*H*O), 3.41 (1H, dd, *J* 3.2 and 4.0 Hz, CHC*H*O), 3.73 (1H, dd, *J* 2.8 and 5.2 Hz, CHC*H*C(O)), 3.79 (3H, s, OCH_3), 4.17-4.19 (1H, m, CH_2C*H*N), 6.89 (2H, d, *J* 9.0 Hz, Ar H), 7.30 (2H, d, *J* 9.0 Hz, Ar H); δ_C (100 MHz, $CDCl_3$) 18.0 (CH_2), 18.2 (CH_2), 47.1 (CH, *C*HC(O)), 49.2 (CH, CH*C*HO), 49.6 (CH, *C*HN), 51.9 (CH, CH_2*C*HO), 55.6 (CH_3, OCH_3), 114.6 (2 x CH, Ar), 118.8 (2 x CH, Ar), 130.4 (C, ArCN), 156.4 (C, ArCO), 163.2 (C, C(O)N); m/z (ES) 268.0938 ($[M+Na]^+$, $C_{14}H_{15}NNaO_3$ requires 258.0950), 314 (9%), 300 (8), 269 (7), 268 (100).

(±)-(3*S*,6*S*)-3-Hydroxy-7-(4-methoxyphenyl)-7-azabicyclo[4.2.0]oct-1-en-8-one **71**

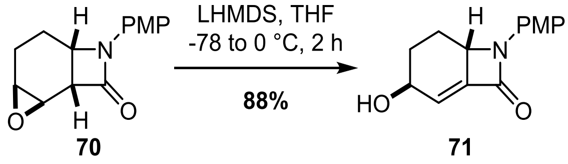

A novel compound prepared according to a modified literature procedure. [20] A solution of epoxy lactam **70** (0.496 g, 2.02 mmol) in THF (13 mL) was cooled to -78 °C and LHMDS (3.2 mL of a 1.0 M solution in THF, 3.2 mmol) was added dropwise over 2 min. The reaction mixture was allowed to warm to 0 °C over 2 h, quenched with H_2O (10 mL) and extracted with EtOAc (3 x 10 mL). The combined organic extracts were washed with brine (15 mL), dried over $MgSO_4$, filtered and evaporated under reduced pressure to give hydroxyl lactam **71** (0.438 g, 88%) as a white solid. mp

164-165 °C (CH_2Cl_2); R_f 0.26 (Pet ether:EtOAc 3:1); ν_{max} (neat)/cm^{-1}: 3416, 2960, 1714, 1689, 1512, 1245, 1121, 984, 825; δ_H (300 MHz, $CDCl_3$) 1.47-1.69 (2H, m, CH_2), 1.84 (1H, d, *J* 6.7 Hz, OH), 2.30-2.55 (2H, m, CH_2), 3.79 (3H, s, OCH_3), 4.26-4.38 (1H, m, CH_2C*H*N), 4.47-4.61 (1H, m, CH_2C*H*OH), 6.29 (1H, dd, *J* 0.6 and 4.2 Hz, CHC*H*=C), 6.88 (2H, d, *J* 9.0 Hz, Ar H), 7.32 (2H, d, *J* 9.0 Hz, Ar H); δ_C (100 MHz, $CDCl_3$) 29.3 (CH_2), 30.8 (CH_2), 55.7 (CH_3, OCH_3), 57.5 (CH, CH_2CHN), 68.5 (CH, CH_2*C*HOH), 114.8 (2 x CH, Ar), 117.7 (2 x CH, Ar), 125.2 (CH, CH*C*H=C), 132.1 (C, ArCN), 145.2 (C, CH=*C*C(O)), 156.4 (C, ArCO), 159.6 (C, C(O)N); m/z (ES) 246.1128 ($[M+H]^+$ $C_{14}H_{16}NO_3$ requires 246.1125), 268 (M^+, 75%), 246 (100), 199 (19), 149 (56).

(±)-(3*S*,6*S*)-7-(4-Methoxyphenyl)-8-oxo-7-azabicyclo[4.2.0]oct-1-en-3-yl benzoate **72**

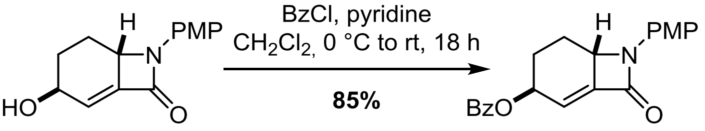

71 **72**

A novel compound prepared according to a modified literature procedure. [21] A solution of hydroxyl lactam **71** (0.109 g, 0.444 mmol) in CH_2Cl_2 (5.6 mL) was cooled to 0 °C and treated with BzCl (206 µL, 1.78 mmol) and pyridine (0.180 µL, 2.22 mmol). The reaction mixture was allowed to warm to rt over 18 h, quenched with $NaHCO_3$ (6 mL of a saturated aq. solution) and extracted with CH_2Cl_2 (3 x 6 mL). The combined organic extracts were dried over $MgSO_4$, filtered, evaporated under reduced pressure and purified by column chromatography (Pet ether to Pet ether:EtOAc 4:1) to give benzoyl protected lactam **72** (0.132 mg, 85%) as a white solid. mp 167-169 °C; R_f 0.48 (Pet ether:EtOAc 4:1); ν_{max} (neat)/cm^{-1}: 2943, 1747, 1710, 1506, 1240, 1111, 1020, 947, 832, 705; δ_H (400 MHz, $CDCl_3$) 1.61-1.89 (2H, m, CH_2), 2.47-2.63 (2H, m, CH_2), 3.79 (3H, s, OCH_3), 4.34-4.45 (1H, m, CH_2C*H*N), 5.72-5.84 (1H, m, CH_2C*H*O), 6.38 (1H, dd, *J* 1.2 and 4.5 Hz, CHC*H*=C), 6.89 (2H, d, *J* 9.0 Hz, Ar H), 7.35 (2H, d, *J* 9.0 Hz, Ar H), 7.42-7.51 (2H, m, Ar H), 7.54-7.64 (1H, m, Ar H), 8.02-8.15 (2H, m, Ar H); δ_C (100 MHz, $CDCl_3$) 26.8 (CH_2), 29.1 (CH_2), 55.7 (CH_3, OCH_3), 57.1 (CH, CH_2*C*HN), 70.5 (CH, CH_2*C*HOBz), 114.8 (2 x CH, Ar), 117.8 (2 x CH, Ar), 121.6 (CH, CH*C*H=C), 128.6 (2 x CH, Ar), 129.9 (2 x CH, Ar), 130.0 (C, Ar), 132.1 (C, ArCN), 133.4 (CH, Ar), 147.4 (C, CH=*C*C(O)), 156.5 (C, ArCO), 159.0 (C, C(O)N), 166.3 (C, C(O)O); m/z (ES) 372.1218 ($[M+Na]^+$ $C_{21}H_{19}NNaO_4$ requires 372.1212), 721 (100%), 404 (32), 372 (75), 228 (17).

(±)-(1*R*,2*S*,3*S*,6*S*)-1,2-Dihydroxy-7-(4-methoxyphenyl)-8-oxo-7-azabicyclo[4.2.0]octan-3-yl benzoate **74**

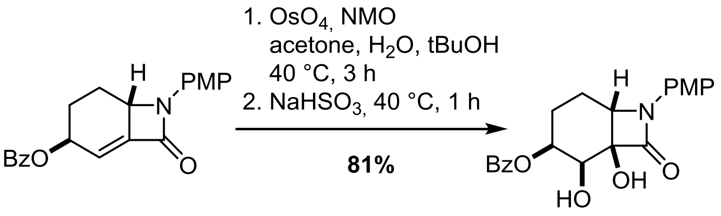

72 **74**

A novel compound prepared according to a modified literature procedure. [22] A solution of lactam **72** (0.162 g, 0.464 mmol) in a 5:5:2:2 mixture of H_2O:acetone:*t*BuOH:CH_2Cl_2 (3.0 mL) was treated with NMO (0.130 g, 1.11 mmol) and OsO_4 (2 µL of a 4 wt% aq. solution, cat). After 3 h at 40 °C, sodium bisulfite (0.264 g, 1.39 mmol) was added to quench the reaction. After 1 h at 40 °C, the reaction mixture was diluted with H_2O (3 mL) and extracted with CH_2Cl_2 (3 x 5 mL). The combined organic extracts were washed with brine (10 mL), dried over $MgSO_4$, filtered, evaporated under reduced pressure and purified by column chromatography (Pet ether:EtOAc 3:1 to Pet ether:EtOAc 1:3) to

give dihydroxy lactam **74** (0.144 g, 81%) as a colourless oil. R_f 0.26 (Pet ether:EtOAc 1:3); ν_{max} (neat)/cm^{-1}: 3396, 2938, 1717, 1512, 1273, 1246, 1112, 905, 725; δ_H (400 MHz, $CDCl_3$) 1.71-1.97 (2H, m, CH_2), 1.99-2.13 (1H, m, CH_2), 2.17-2.34 (1H, m, CH_2), 3.75 (3H, s, OCH_3), 4.29 (1H, dd, *J* 2.3 and 4.0 Hz, CH_2C*H*N), 4.44 (1H, d, *J* 2.7 Hz, CHC*H*OH), 5.11 (2H, br s, OH), 5.30 (1H, td, *J* 2.7 and 6.5 Hz, CH_2C*H*OBz), 6.81 (2H, d, *J* 9.0 Hz, Ar H), 7.22 (2H, d, *J* 9.0 Hz, Ar H), 7.34 (2H, t, *J* 7.7 Hz, Ar H), 7.43-7.52 (1H, m, Ar H), 7.99-8.09 (2H, m, Ar H); δ_C (100 MHz, $CDCl_3$) 19.3 (CH_2), 21.8 (CH_2), 55.6 (CH_3, OCH_3), 61.0 (CH, CH_2*C*HN), 68.7 (CH, CH*C*HOH), 70.6 (CH, CH_2*C*HOBz), 82.4 (C, CH*C*OH), 114.6 (2 x CH, Ar), 119.6 (2 x CH, Ar), 128.4 (2 x CH, Ar), 129.5 (2 x CH, Ar), 129.9 (C, ArCN), 130.0 (C, Ar), 133.1 (CH, Ar), 156.9 (C, ArCO), 166.1 (C, C(O)O), 167.3 (C, C(O)N); m/z (ES) 406.1266 ($[M+Na]^+$ $C_{21}H_{21}NNaO_6$ requires 406.1267), 790 (48%), 789 (74), 407 (33), 406 (100), 384 (10).

(±)-(3a*R*,5a*S*,8*S*,8a*S*)-5-(4-Methoxyphenyl)-2-oxido-4-oxohexahydro-4*H*-[1,3,2] dioxathiolo[4',5':2,3] benzo[1,2-*b*]azet-8-yl benzoate **102a** and **102b**

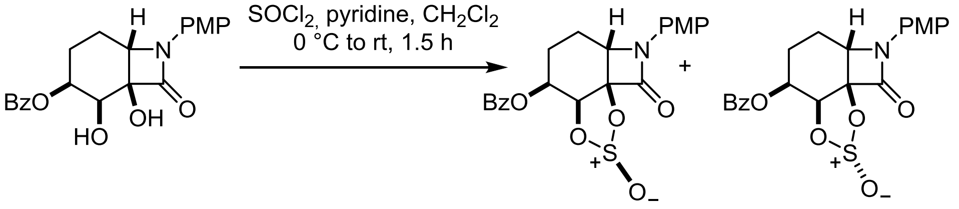

Novel compounds prepared according to a literature procedure.[23] A solution of dihydroxy lactam **74** (93 mg, 0.26 mmol) in CH_2Cl_2 (1.4 mL) at 0 °C was treated with pyridine (93 µL, 1.2 mmol) and $SOCl_2$ (37 µL, 0.51 mmol). The ice bath was removed after 1 h and stirring continued for 30 min at rt. The reaction mixture was diluted with H_2O (2 mL) and extracted with CH_2Cl_2 (2 x 3 mL). The combined organic extracts were washed sequentially with HCl (3 mL of a 1 M aq. solution) and brine (3 mL), dried over $MgSO_4$, filtered, evaporated under reduced pressure and purified by column chromatography (Pet ether to Pet ether:EtOAc 7:3) to give **102a** (49 mg, 47%) as a white solid and **102b** (41 mg, 40%) as a colourless oil.

102a: mp 190-191 °C; R_f 0.59 (Pet ether:EtOAc 7:3); ν_{max} (neat)/cm^{-1}: 2935, 1729, 1511, 1243, 1027, 834; δ_H (400 MHz, $CDCl_3$) 1.83-1.93 (1H, m, CH_2), 1.95-2.07 (1H, m, CH_2), 2.22-2.33 (1H, m, CH_2), 2.43-2.54 (1H, m, CH_2), 3.81 (3H, s, OCH_3), 4.61 (1H, dd, *J* 3.6 and 7.6 Hz, CH_2C*H*N), 5.19 (1H, d, *J* 3.9 Hz, CHC*H*OS), 5.53 (1H, ddd, *J* 2.4, 3.9 and 9.4 Hz, CH_2C*H*OBz), 6.93 (2H, d, *J* 9.0 Hz, Ar H), 7.32 (2H, d, *J* 9.0 Hz, Ar H), 7.46 (2H, t, *J* 7.7 Hz, Ar H), 7.59 (1H, tt, *J* 1.2 and 7.4 Hz, Ar H), 8.07-8.15 (2H, m, Ar H); δ_C (100 MHz, $CDCl_3$) 21.4 (CH_2), 21.4 (CH_2), 55.7 (CH_3, OCH_3), 59.5 (CH, CH_2*C*HN), 67.0 (CH, CH_2*C*HOBz), 81.4 (CH, CH*C*HOS), 90.3 (C, CH*C*OS), 115.0 (2 x CH, Ar), 119.6 (2 x CH, Ar), 128.6 (2 x CH, Ar), 129.1 (C, Ar), 129.4 (C, Ar), 130.2 (2 x CH, Ar), 133.7 (CH, Ar), 157.5 (C, ArCO), 158.5 (C, C(O)N), 165.6 (C, C(O)O); m/z (ES) 452.0781 ($[M+Na]^+$ $C_{21}H_{19}NNaO_7S$ requires 452.0780), 881 (13%), 453 (25), 452 (100), 258 (39), 212 (38).

102b: R_f 0.30 (Pet ether:EtOAc 7:3); ν_{max} (neat)/cm^{-1}: 2935, 1754, 1728, 1511, 1245, 1027, 830, 820; δ_H (400 MHz, $CDCl_3$) 1.68-1.80 (1H, m, CH_2), 2.12-2.27 (3H, m, CH_2), 3.80 (3H, s, OCH_3), 4.67 (1H, t, *J* 3.9 Hz, CH_2C*H*N), 5.40 (1H, d, *J* 3.9 Hz, CHC*H*OS), 5.61-5.68 (1H, m, CH_2C*H*OBz), 6.92 (2H, d, *J* 9.0 Hz, Ar H), 7.33 (2H, d, *J* 9.0 Hz, Ar H), 7.48 (2H, t, *J* 7.6 Hz, Ar H), 7.62 (1H, tt, *J* 1.2 and 7.6 Hz, Ar H), 7.95-8.04 (2H, m, Ar H); δ_C (100 MHz, $CDCl_3$) 18.0 (CH_2), 20.9 (CH_2), 55.7 (CH_3, OCH_3), 57.8 (CH, CH_2*C*HN), 66.3 (CH, CH_2*C*HOBz), 79.4 (CH, CH*C*HOS), 89.7 (C, CH*C*OS), 115.0 (2 x CH, Ar), 119.9 (2 x CH, Ar), 128.9 (2 x CH, Ar), 128.9 (C, Ar), 129.3 (C, Ar), 129.8 (2 x CH, Ar), 133.9 (CH, Ar), 157.4 (C, ArCO),

160.9 (C, C(O)N), 165.2 (C, C(O)O); m/z (ES) 452.0786 ([M+Na]$^+$ $C_{21}H_{19}NNaO_7S$ requires 452.0780), 484 (16%), 453 (25), 452 (100).

(±)-(1*R*,2*S*,5*S*)-6-(4-Methoxyphenyl)-7,8-dioxo-6-azabicyclo[3.2.1]octan-2-yl benzoate **80**

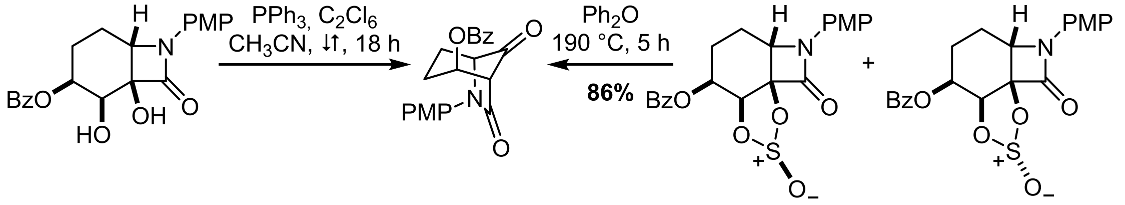

74 **80** **102a** **102b**

A novel compound prepared according to a modified literature procedure.[24] A solution of PPh_3 (50 mg, 0.19 mmol) and hexachloroethane (45 mg, 0.19 mmol) in CH_3CN (0.7 mL) was stirred at rt for 30 min before being added dropwise to a solution of diol **74** (49 mg, 0.13 mmol) in CH_3CN (0.3 mL). The reaction mixture was heated at reflux for 18 h, concentrated under reduced pressure and purified directly by column chromatography (Pet ether to Pet ether:EtOAc 4:1) to give bicyclic ketone **80** (36 mg, 77%) as a white solid.

A solution of cyclic sulfites **102** (66 mg, 0.15 mmol) in diphenyl ether (1.5 mL) was heated to 190 °C. After 5 h, the reaction mixture was directly purified by column chromatography (Pet ether to Pet ether:EtOAc 7:3) to give bicyclic ketone **80** (48 mg, 86%) as a white solid.

mp 163-164 °C (CH_2Cl_2); R_f 0.68 (Pet ether:EtOAc 1:1); ν_{max} (neat)/cm^{-1}: 2933, 1784, 1718, 1695, 1512, 1389, 1245, 1090, 816, 701; δ_H (400 MHz, $CDCl_3$) 2.08-2.36 (3H, m, CH_2), 2.43-2.54 (1H, m, CH_2), 3.49 (1H, dd, *J* 1.3 and 5.6 Hz, C(O)CHC(O)), 3.81 (3H, s, OCH_3), 4.42 (1H, m, C(O)CHN), 5.83-5.92 (1H, m, CH_2C*H*O), 6.94 (2H, d, *J* 9.1 Hz, Ar H), 7.42-7.53 (4H, m, Ar H), 7.58 (1H, tt, *J* 1.2 and 7.4 Hz, Ar H), 7.97-8.03 (2H, m, Ar H); δ_C (100 MHz, $CDCl_3$) 23.9 (CH_2), 27.6 (CH_2), 55.7 (CH_3, OCH_3), 57.9 (CH, C(O)*C*HC(O)), 67.1 (CH, CH_2*C*HN), 74.1 (CH, CH_2CHO), 114.8 (2 x CH, Ar), 123.0 (2 x CH, Ar), 128.7 (2 x CH, Ar), 129.5 (2 x C, ArCN and Ar), 129.9 (2 x CH, Ar), 133.7 (CH, Ar), 157.8 (C, ArCO), 165.2 (C, C(O)O), 165.7 (C, C(O)N), 202.6 (C, CH*C*(O)CH); m/z (ES) 388.1158 ([M+Na]$^+$ $C_{21}H_{19}NNaO_5$ requires 388.1161), 420 (15%), 389 (29), 388 (100).

(±)-(1*S*,2*R*,5*S*,6*R*,7*R*)-5,6-Dihydroxy-3-(4-methoxyphenyl)-3-azatricyclo[5.2.1.0^{2,5}] decan-4-one **76** and (±)-(1*S*,2*R*,5*S*,7*R*)-5-Hydroxy-3-(4-methoxyphenyl)-3-azatricyclo[5.2.1.0^{2,5}] decane-4,6-dione **77**

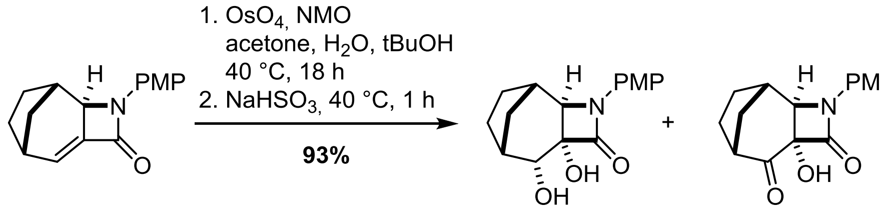

63 **76** 1.0:0.16 **77**

Novel compounds prepared according to a modified literature procedure.[22] A solution of lactam **63** (95 mg, 0.37 mmol) in a 5:5:2 mixture of H_2O:acetone:*t*BuOH (2.1 mL) was treated with NMO (0.105 g, 0.893 mmol) and OsO_4 (2 µL of a 4 wt% aq. solution, cat). After 18 h at 40 °C, sodium bisulfite (0.212 g, 1.12 mmol) was added to quench the reaction. After 1 h at 40 °C, the reaction mixture was diluted with H_2O (5 mL) and extracted with EtOAc (2 x 5 mL). The combined organic extracts were washed with brine (10 mL), dried over $MgSO_4$, filtered, evaporated under reduced pressure and purified by column chromatography (Pet ether:EtOAc 3:1 to Pet ether:EtOAc 1:1) to give a 1.0:0.16 mixture of diol **76**:hydroxyl ketone **77** (0.100 g, 93%) as a yellow solid (for analytical data for **77**, see

below). mp 162-165 °C; R_f 0.35 (Pet ether:EtOAc 1:1); ν_{max} (neat)/cm^{-1}: 3492, 3274, 2925, 1702, 1515, 1399, 1251, 1082, 1021, 827, 816; δ_H (400 MHz, $CDCl_3$) 1.35-1.47 (1H, m, CH_2), 1.54 (1H, d, *J* 13.0 Hz, CH_2), 1.57-1.71 (2H, m, CH_2), 1.72-1.93 (2H, m, CH_2), 2.45 (1H, dd, *J* 6.4 and 11.6 Hz, CH_2C*H*CHOH), 2.67 (1H, t, *J* 4.4 Hz, CH_2C*H*CHN), 3.78 (3H, s, OCH_3), 4.02 (1H, t, *J* 1.6 Hz, CHC*H*N), 4.23 (1H, s, C*H*OH), 4.99 (2H, br s, OH), 6.87 (2H, d, *J* 9.0 Hz, Ar H), 7.32 (2H, d, *J* 9.0 Hz, Ar H); δ_C (100 MHz, $CDCl_3$) 20.5 (CH_2), 24.9 (CH_2), 27.7 (CH_2), 29.6 (CH, CH_2*C*HCHO), 33.9 (CH, CH_2*C*HCHN), 55.7 (CH_3, OCH_3), 68.3 (CH, CH*C*HOH), 69.2 (CH, CH*C*HN), 79.4 (C, CH*C*OH), 114.6 (2 x CH, Ar), 119.8 (2 x CH, Ar), 130.1 (C, ArCN), 156.9 (C, ArCO), 170.6 (C, C(O)N); m/z (ES) 290.1389 ($[M+H]^+$, $C_{16}H_{20}NO_4$ requires 290.1387), 312 (24%), 307 (20), 305 (16), 291 (17),290 (100).

(±)-(3a*S*,5a*R*,6*S*,9*R*,9a*S*)-5-(4-Methoxyphenyl)hexahydro-6,9-methanol[1,3,2] dioxathiolo[4',5':2,3] cyclohepta[1,2-*b*]azet-4(5H)-one 2-oxide **103a** and **103b**

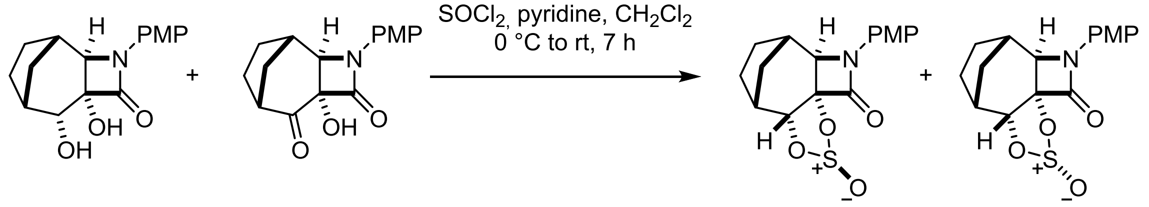

Novel compounds prepared according to a literature procedure. [23] A solution of a 1.0:0.16 mixture of diol **76**:hydroxyl ketone **77** (65 mg, 0.23 mmol) in CH_2Cl_2 (1.2 mL) at 0 °C was treated with pyridine (82 µL, 1.0 mmol) and $SOCl_2$ (33 µL, 0.45 mmol). The ice bath was removed after 30 min and stirring continued at rt for 6.5 h. The reaction mixture was diluted with H_2O (2 mL) and extracted with CH_2Cl_2 (2 x 3 mL). The combined organic extracts were washed sequentially with HCl (3 mL of a 1 M aq. solution) and brine (3 mL), dried over $MgSO_4$, filtered, evaporated under reduced pressure and purified by column chromatography (Pet ether to Pet ether:EtOAc 3:2) to give **103a** (31 mg, 41%) and **103b** (26 mg, 35%) as white solids and hydroxyl ketone **77** (9 mg, 14% based on recovered SM), an impurity from the dihydroxylation reaction, as a colourless oil.

103a: mp 170-173 °C; R_f 0.37 (Pet ether:EtOAc 7:3); ν_{max} (neat)/cm^{-1}: 2957, 1745, 1510, 1244, 971, 798, 660; δ_H (300 MHz, $CDCl_3$) 1.51-1.75 (5H, m, CH_2), 1.83-1.97 (1H, m, CH_2), 2.67-2.75 (1H, m, CH_2C*H*CHO), 2.78 (1H, t, *J* 5.5 Hz, CH_2C*H*CHN), 3.79 (3H, s, OCH_3), 4.16-4.19 (1H, m, CHC*H*N), 5.33 (1H, d, *J* 5.8 Hz, CHC*H*O), 6.90 (2H, d, *J* 9.0 Hz, Ar H), 7.37 (2H, d, *J* 9.0 Hz, Ar H); δ_C (100 MHz, $CDCl_3$) 21.1 (CH_2), 26.8 (CH_2), 27.9 (CH_2), 33.6 (CH, CH_2*C*HCHO), 35.1 (CH, CH_2*C*HCHN), 55.6 (CH_3, OCH_3), 66.1 (CH, CH*C*HN), 81.6 (CH, CH*C*HOS), 88.3 (C, CH*C*OS), 114.8 (2 x CH, Ar), 119.5 (2 x CH, Ar), 129.6 (C, ArCN), 157.1 (C, ArCO), 161.6 (C, C(O)N); m/z (ES) 336.0906 ($[M+H]^+$ $C_{16}H_{18}NO_5S$ requires 336.0900), 371 (17%), 353 (45), 336 (100).

103b: mp 152-153 °C; R_f 0.62 (Pet ether:EtOAc 7:3); ν_{max} (neat)/cm^{-1}: 2943, 1749, 1511, 1206, 974, 823, 791, 649; δ_H (300 MHz, $CDCl_3$) 1.50-1.78 (4H, m, CH_2), 1.87-1.99 (1H, m, CH_2), 2.14-2.30 (1H, m, CH_2), 2.71 (1H, q, *J* 6.2 Hz, CH_2C*H*CHO), 2.78 (1H, t, *J* 6.0 Hz, CH_2C*H*CHN), 3.80 (3H, s, OCH_3), 4.25-4.30 (1H, m, CHC*H*N), 5.12 (1H, d, *J* 6.8 Hz, CHC*H*O), 6.91 (2H, d, *J* 9.0 Hz, Ar H), 7.36 (2H, d, *J* 9.0 Hz, Ar H); δ_C (100 MHz, $CDCl_3$) 23.8 (CH_2), 25.5 (CH_2), 27.4 (CH_2), 34.0 (CH, CH_2*C*HCHO), 35.5 (CH, CH_2*C*HCHN), 55.7 (CH_3, OCH_3), 68.1 (CH, CH*C*HN), 85.8 (CH, CH*C*HOS), 88.1 (C, CH*C*OS), 114.8 (2 x CH, Ar), 119.7 (2 x CH, Ar), 129.4 (C, ArCN), 157.3 (C, ArCO), 160.9 (C, C(O)N); m/z (ES) 336.0906 ($[M+H]^+$ $C_{16}H_{18}NO_5S$ requires 336.0900), 532 (14%), 445 (12), 353 (35), 336 (100).

77: mp 149-150 °C; R_f 0.41 (Pet ether:EtOAc 1:1); ν_{max} (neat)/cm^{-1}: 3308, 1753, 1687, 1514, 1251, 1151, 1015, 833; δ_H (300 MHz, $CDCl_3$) 1.52-1.87 (3H, m, CH_2), 2.00-2.19 (3H, m, CH_2), 2.91-3.06 (2H,

m, CH_2CHCHN and $CH_2CHC(O)$), 3.80 (3H, s, OCH_3), 3.96-4.28 (2H, m, CHC*H*N and OH), 6.91 (2H, d, *J* 9.0 Hz, Ar H), 7.38 (2H, d, *J* 9.0 Hz, Ar H); δ_C (100 MHz, $CDCl_3$) 24.9 (CH_2), 27.7 (CH_2), 29.6 (CH_2), 34.1 (CH), 48.8 (CH), 55.7 (CH_3, OCH_3), 63.2 (CH, CH*C*HN), 85.8 (C, CH*C*OH), 114.9 (2 x CH, Ar), 119.5 (2 x CH, Ar), 129.9 (C, ArCN), 157.1 (C, ArCO), 161.4 (C, C(O)N), 207.3 (C, CH*C*(O)C); m/z (ES) 305.1503 ($[M+NH_4]^+$ $C_{16}H_{21}N_2O_4$ requires 305.1496), 371 (19%), 305 (100), 139 (68).

(±)-(1*S*,2*R*,6*R*)-3-(4-Methoxyphenyl)-3-azatricyclo[4.2.1.1^{2,5}]decan-4,10-dione **82**

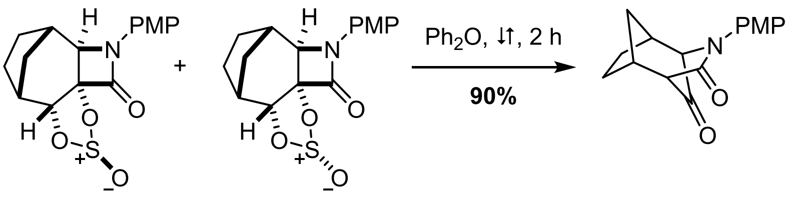

103a **103b** **82**

A novel compound prepared according to a modified literature procedure.[25] A solution of cyclic sulfites **103** (59 mg, 0.18 mmol) in diphenyl ether (1.8 mL) were heated to reflux. After 2 h, the reaction mixture was directly purified by column chromatography (Pet ether to Pet ether:EtOAc 4:1) to give tricyclic ketone **82** (43 mg, 90%) as pale yellow crystals. mp 112-114 °C; R_f 0.35 (Pet ether:EtOAc 7:3); ν_{max} (neat)/ cm^{-1}: 2959, 1774, 1692, 1510, 1375, 1215, 1027, 831; δ_H (400 MHz, $CDCl_3$) 1.45 (1H, dt, *J* 3.2 and 12.7 Hz, CH_2), 1.64-1.90 (4H, m, CH_2), 1.93 (1H, d, *J* 12.7 Hz, CH_2), 2.77-2.85 (1H, m, CH_2CHCHN), 2.97-3.03 (1H, m, $CH_2CHCHC(O)$), 3.05 (1H, dd, *J* 2.3 and 7.1 Hz, CHC*H*C(O)), 3.79 (3H, s, OCH_3), 4.08 (1H, dd, *J* 2.3 and 6.2 Hz, CHC*H*N), 6.89 (2H, d, *J* 9.1 Hz, Ar H), 7.44 (2H, d, *J* 9.1 Hz, Ar H); δ_C (100 MHz, $CDCl_3$) 27.0 (CH_2), 27.7 (CH_2), 31.7 (CH_2), 40.4 (CH, $CH_2CHCHC(O)$), 41.8 (CH, CH_2CHCHN), 55.6 (CH_3, OCH_3), 60.1 (CH, CH*C*HC(O)), 69.9 (CH, CH*C*HN), 114.5 (2 x CH, Ar), 122.2 (2 x CH, Ar), 130.5 (C, ArCN), 157.1 (C, ArCO), 168.0 (C, C(O)N), 201.3 (C, CH*C*(O)CH); m/z (ES) 272.1283 ($[M+H]^+$, $C_{16}H_{18}NO_3$ requires 272.1281), 565 (10%), 543 (56), 272 (100).

(±)-(1*S*,2*R*,5*S*,6*S*,7*R*)-6-Chloro-5-hydroxy-3-(4-methoxyphenyl)-3-azatricyclo[5.2.1.0^{2,5}] decan-4-one **83**

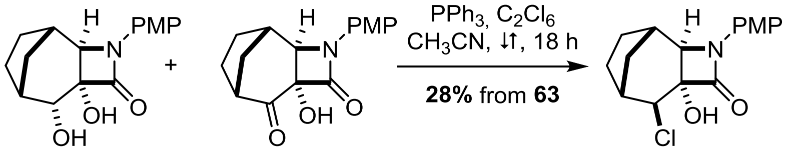

76 **77** **83**

A novel compound prepared according to a modified literature procedure.[24] A solution of PPh_3 (0.111 g, 0.425 mmol) and hexachloroethane (0.100 g, 0.425 mmol) in CH_3CN (1.0 mL) was stirred at rt for 20 min before being added dropwise to a solution of a 1.0:0.16 mixture of diol **76**:hydroxyl ketone **77** (82 mg, 0.28 mmol) in CH_3CN (0.9 mL). The reaction mixture was heated at reflux for 18 h, evaporated under reduced pressure and purified directly by column chromatography (Pet ether to Pet ether:EtOAc 1:1) to give chloro hydroxyl lactam **83** (26 mg, 28% from alkene **63**) as a white solid. mp 224-225 °C; R_f 0.67 (Pet ether:EtOAc 3:2); ν_{max} (neat)/cm^{-1}: 3294, 2936, 1696, 1513, 1248, 1159, 1026, 819; δ_H (400 MHz, $CDCl_3$) 1.50-1.66 (2H, m, CH_2), 1.86 (1H, dd, *J* 5.3 and 15.4 Hz, CH_2), 2.05 (1H, tdd, *J* 2.6, 5.6 and 13.4 Hz, CH_2), 2.18-2.36 (2H, m, CH_2), 2.42 (1H, d, *J* 3.1 Hz, $CH_2CHCHCl$), 2.54 (1H, t, *J* 4.2 Hz, CH_2CHCHN), 3.51 (1H, br s, OH), 3.78 (3H, s, OCH_3), 4.02 (1H, d, *J* 1.2 Hz, CHC*H*N), 4.64 (1H, s, CHC*H*Cl), 6.83 (2H, d, *J* 9.0 Hz, Ar H), 7.28 (2H, d, *J* 9.0 Hz, Ar H); δ_C (100 MHz, $CDCl_3$) 24.5 (CH_2), 27.8 (CH_2), 27.8 (CH_2), 33.8 (CH, $CH_2CHCHCl$), 43.4 (CH, CH_2CHCHN), 55.8 (CH_3, OCH_3), 59.0 (CH, CH*C*HCl),

67.3 (CH, CH*C*HN), 91.4 (C, CH*C*OH), 114.7 (2 x CH, Ar), 119.0 (2 x CH, Ar), 130.0 (C, ArCN), 156.9 (C, ArCO), 165.8 (C, C(O)N); m/z (ES) 330.0867 ($[M+Na]^+$, $C_{16}H_{18}{}^{35}ClNNaO_3$ requires 330.0873), 364 (13%), 362 (59), 332 (42), 330 (100), 308 (8), 294 (7).

3-Hydroxy-3-(hydroxymethyl)-1-(4-methoxyphenyl)azetidin-2-one **78**

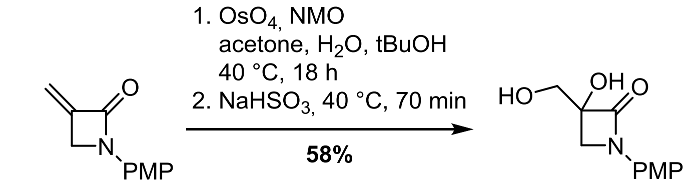

67 **78**

A novel compound prepared according to a modified literature procedure.[22] A solution of lactam **67** (0.152 g, 0.803 mmol) in a 5:5:2 mixture of H_2O:acetone:*t*BuOH (4.5 mL) was treated with NMO (0.226 g, 1.93 mmol) and OsO_4 (2 µL of a 4 wt% aq. solution, cat). After 18 h at 40 °C, sodium bisulfite (0.456 g, 2.41 mmol) was added to quench the reaction. After 70 min at 40 °C, the reaction mixture was diluted with H_2O (5 mL) and extracted with CH_2Cl_2 (2 x 10 mL). The combined organic extracts were washed with brine (15 mL), dried over $MgSO_4$, filtered, evaporated under reduced pressure and purified by column chromatography (Pet ether:EtOAc 1:1 to Pet ether:EtOAc 1:3) to give dihydroxy lactam **78** (0.104 g, 58%) as a white solid. mp 138-140 °C; R_f 0.19 (Pet ether:EtOAc 1:3); ν_{max} (neat)/cm^{-1} : 3352, 3294, 2969, 2488, 2447, 1713, 1513, 1233, 1044, 825; δ_H (400 MHz, CD_3OD) 3.54, 3.92 (2H, ABq, *J* 5.8 Hz, CH_2N), 3.72, 3.82 (2H, ABq, *J* 11.4 Hz, C*H_2*OH), 3.77 (3H, s, OCH_3), 4.87 (2H, br s, OH), 6.92 (2H, d, *J* 9.0 Hz, Ar H), 7.35 (2H, d, *J* 9.0 Hz, Ar H); δ_C (100 MHz, CD_3OD) 53.0 (CH_2, NCH_2), 55.9 (CH_3, OCH_3), 63.3 (CH_2, CH_2OH), 85.0 (C, COH), 115.4 (2 x CH, Ar), 119.3 (2 x CH, Ar), 132.8 (C, ArCN), 158.1 (C, ArCO), 168.7 (C, C(O)N); m/z (ES) 224.0915 ($[M+H]^+$ $C_{11}H_{14}NO_4$ requires 224.0917), 246 (55%), 241 (18), 224 (100), 199 (17).

(±)-(4*S*)-2-(4-Methoxyphenyl)-5,7-dioxa-6-thia-2-azaspiro[3.4]octan-1-one 6-oxide **104a** and **104b**

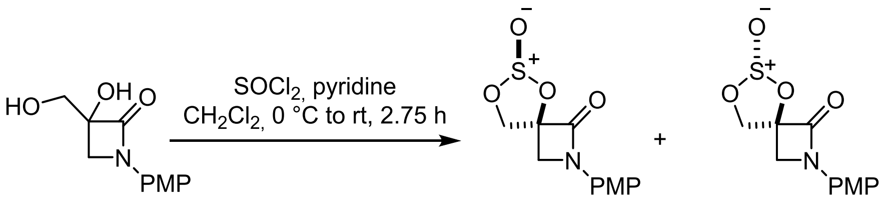

78 **104a** 30% **104b 38%**

Novel compounds prepared according to a modified literature procedure.[23] A solution of dihydroxy lactam **78** (52 mg, 0.23 mmol) in CH_2Cl_2 (1.2 mL) at 0 °C was treated with pyridine (85 µL, 1.1 mmol) and $SOCl_2$ (34 µL, 0.47 mmol). The ice bath was removed after 1 h and stirring was continued for 1.75 h at rt. The reaction mixture was diluted with H_2O (2 mL) and extracted with CH_2Cl_2 (2 x 3 mL). The combined organic extracts were washed sequentially with HCl (3 mL of a 1 M aq. solution) and brine (3 mL), dried over $MgSO_4$, filtered, evaporated under reduced pressure and purified by column chromatography (Pet ether to Pet ether:EtOAc 4:1) to give **104a** (19 mg, 30%) and **104b** (24 mg, 38%) as white solids.

104a: mp 157-159 °C; R_f 0.19 (Pet ether:EtOAc 7:3); ν_{max} (neat)/cm^{-1} : 2953, 1744, 1511, 1216, 948, 828, 766; δ_H (400 MHz, $CDCl_3$) 3.80 (3H, s, OCH_3), 3.89, 3.91 (2H, ABq, *J* 6.5 Hz, CH_2N), 4.84, 5.05 (2H, ABq, *J* 9.6 Hz, CH_2OS), 6.90 (2H, d, *J* 9.0 Hz, Ar H), 7.31 (2H, d, *J* 9.0 Hz, Ar H); δ_C (100 MHz, $CDCl_3$) 52.4 (CH_2, NCH_2), 55.7 (CH_3, OCH_3), 70.3 (CH_2, CH_2OS), 90.5 (C, COS), 114.8 (2 x CH, Ar), 118.5 (2 x CH, Ar), 130.8 (C, ArCN), 157.3 (C, ArCO), 159.0 (C, C(O)N); m/z (CI) 287.0694 ($[M+NH_4]^+$ $C_{11}H_{15}N_2O_5S$ requires 287.0696), 287 (100%), 207 (49).

104b: mp 147-148 °C; R_f 0.38 (Pet ether:EtOAc 7:3); ν_{max} (neat)/cm^{-1} : 2953, 1744, 1511, 1216, 948, 828, 766; δ_H (400 MHz, $CDCl_3$) 3.81 (3H, s, OCH_3), 4.03, 4.18 (2H, ABq, *J* 6.8 Hz, CH_2N), 4.75, 5.02 (2H, ABq, *J* 9.1 Hz, CH_2OS), 6.91 (2H, d, *J* 9.0 Hz, Ar H), 7.31 (2H, d, *J* 9.0 Hz, Ar H); δ_C (100 MHz, $CDCl_3$) 52.4 (CH_2, NCH_2), 55.7 (CH_3, OCH_3), 71.5 (CH_2, CH_2OS), 88.8 (C, COS), 114.8 (2 x CH, Ar), 118.6 (2 x CH, Ar), 130.7 (C, ArCN), 157.4 (C, ArCO), 158.9 (C, C(O)N); m/z (CI) 287.0694 ($[M+NH_4]^+$ $C_{11}H_{15}N_2O_5S$ requires 287.0696), 287 (100%), 207 (58).

3-(Chloromethyl)-3-hydroxy-1-(4-methoxyphenyl)azetidin-2-one **84**

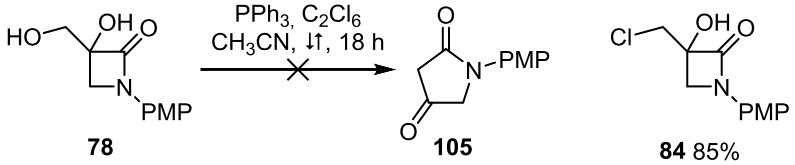

78 **105** **84** 85%

A novel compound prepared according to a literature procedure.[24] A solution of PPh_3 (65 mg, 0.25 mmol) and hexachloroethane (59 mg, 0.25 mmol) in CH_3CN (0.71 mL) was stirred at rt for 30 min before being added dropwise to a solution of diol **78** (37 mg, 0.17 mmol) in CH_3CN (0.40 mL). The reaction mixture was heated at reflux for 18 h, concentrated under reduced pressure and purified directly by column chromatography (Pet ether to Pet ether:EtOAc 7:3) to give chloro hydroxyl lactam **84** (34 mg, 85%) as pale yellow crystals. mp 117-118 °C; R_f 0.54 (Pet ether:EtOAc 1:1); ν_{max} (neat)/cm^{-1} : 3192, 2958, 1705, 1510, 1242, 1167, 1025, 822; δ_H (400 MHz, $CDCl_3$) 3.71, 3.90 (2H, ABq, *J* 6.0 Hz, CH_2N), 3.79 (3H, s, OCH_3), 3.88 (2H, s, CH_2Cl), 5.04 (1H, br s, OH), 6.86 (2H, d, *J* 9.0 Hz, Ar H), 7.27 (2H, d, *J* 9.0 Hz, Ar H); δ_C (100 MHz, $CDCl_3$) 45.3 (CH_2, CH_2Cl), 52.6 (CH_2, NCH_2), 55.7 (CH_3, OCH_3), 83.1 (C, COH), 114.6 (2 x CH, Ar), 118.5 (2 x CH, Ar), 130.8 (C, ArCN), 157.0 (C, ArCO), 165.0 (C, C(O)N); m/z (ES) 242.0579 ($[M+H]^+$ $C_{11}H_{13}{}^{35}ClNO_3$ requires 242.0578), 264 (39%), 244 (32), 242 (100).

(±)-(1*R*,5*S*,8*S*)-8-Hydroxy-6-(4-methoxyphenyl)-6-azabicyclo[3.2.1]octan-7-one **85**

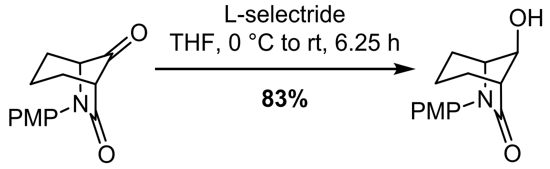

21 **85**

A novel compound prepared according to a modified literature procedure.[26] A solution of bicyclic ketone **21** (47 mg, 0.19 mmol) in THF (1 mL) was cooled to 0 °C and treated with L-selectride (0.29 mL of a 1.0 M solution in THF, 0.29 mmol). The reaction mixture was progressively allowed to warm to rt. After 6.25 h, the reaction mixture was quenched with H_2O (1 mL) and extracted with EtOAc (2 x 3 mL). The combined organic extract were washed with brine (2 mL), dried over $MgSO_4$, filtered, evaporated under reduced pressure and purified by column chromatography (Pet ether:EtOAc 7:3) to give hydroxy lactam **85** (39 mg, 83%) as a white solid. mp 116-118 °C; R_f 0.33 (Pet ether:EtOAc 2:3); ν_{max} (neat)/cm^{-1} : 3327, 2947, 1661, 1511, 1245, 1113, 803; δ_H (400 MHz, $CDCl_3$) 1.46-1.59 (1H, m, CH_2), 1.62-1.73 (3H, m, CH_2), 1.92-2.04 (2H, m, CH_2), 2.55 (1H, br s, C(O)C*H*CH_2),3.43 (1H, br s, OH), 3.77 (3H, s, OCH_3), 3.97-4.00 (1H, m, CH_2C*H*N), 4.34-4.37 (1H, m, CHC*H*(OH)CH), 6.87 (2H, d, *J* 8.8 Hz, Ar H), 7.45 (2H, d, *J* 8.8 Hz, Ar H); δ_C (100 MHz, $CDCl_3$) 17.3 (CH_2), 18.5 (CH_2), 19.6 (CH_2), 46.4 (CH, C(O)*C*HCH_2), 55.6 (CH_3, OCH_3), 61.2 (CH, CH_2CHN), 68.2 (CH, CH*C*HOH), 114.4 (2 x CH, Ar), 122.9 (2 x CH, Ar), 131.3 (C, ArCN), 156.9 (C, ArCO), 174.3 (C, C(O)N); m/z (ES) 270.1111 ($[M+Na]^+$ $C_{14}H_{17}NNaO_3$ requires 270.1106), 270 (100%).

(±)-(1*R*,5*S*)-6-(4-Methoxyphenyl)-8-methylene-6-azabicyclo[3.2.1]octane-7-one **86**

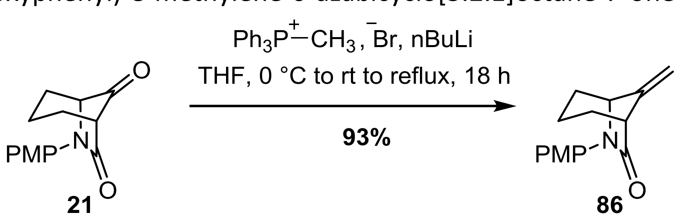

A novel compound prepared according to a modified literature procedure.[27] A solution of $Ph_3P^+–CH_3,Br^-$ (0.291 g, 0.816 mmol) in THF (1.0 mL) was cooled to 0 °C, *n*BuLi (0.51 mL of a 1.6 M solution in hexane, 0.82 mmol) was added dropwise over 2 min. The reaction mixture was stirred for 25 min at rt and a solution of rearranged product **21** (95 mg, 0.39 mmol) in THF (1.6 mL) was added. The reaction mixture was heated at reflux for 18 h, evaporated under reduced pressure and purified directly by column chromatography (Pet ether to Pet ether:EtOAc 7:3) to give lactam **86** (87 mg, 93%) as a pale yellow solid. mp 105-107 °C; R_f 0.78 (Pet ether:EtOAc 1:1); ν_{max} (neat)/cm^{-1}: 2934, 1676, 1610, 1509, 1381, 1243, 1031, 817, 829; δ_H (300 MHz, $CDCl_3$) 1.62-1.81 (4H, m, CH_2), 2.04-2.23 (2H, m, CH_2), 3.02 (1H, m, CCHC(O)), 3.76 (3H, s, OCH_3), 4.49 (1H, d, *J* 3.7 Hz, CH_2C*H*N), 4.76 (1H, s, =CH_2), 4.84 (1H, s, =CH_2), 6.87 (2H, d, *J* 9.1 Hz, Ar H), 7.52 (2H, d, *J* 9.1 Hz, Ar H); δ_C (100 MHz, $CDCl_3$) 18.4 (CH_2), 30.3 (CH_2), 30.6 (CH_2), 50.1 (CH, C*C*HC(O)), 55.7 (CH_3, OCH_3), 64.4 (CH, CH_2*C*HN), 101.1 (CH_2, =CH_2), 114.5 (2 x CH, Ar), 122.2 (2 x CH, Ar), 131.4 (C, ArCN), 151.0 (C, =C), 156.7 (C, ArCO), 173.8 (C, C(O)N); m/z (ES) 266.1145 ($[M+Na]^+$ $C_{15}H_{17}NNaO_2$ requires 266.1157), 266 (100%).

(±)-(1*R*,5*S*)-6-(4-Methoxyphenyl)-8-methylene-6-azabicyclo[3.2.1]octane-7-thione **87**

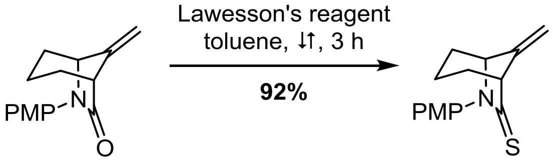

86 **87**

A novel compound prepared according to a modified literature procedure.[28] A solution of Lawesson's reagent (48 mg, 0.12 mmol) and lactam **86** (48 mg, 0.20 mmol) in toluene (2.0 mL) was heated to reflux. After 3 h, the reaction mixture was concentrated under reduced pressure and purified directly by column chromatography (Pet ether to Pet ether:EtOAc 9:1) to give thiolactam **87** (47 mg, 92%) as a pale yellow solid. mp 76-78 °C; R_f 0.76 (Pet ether:EtOAc 7:3); ν_{max} (neat)/cm^{-1}: 2941, 1685, 1606, 1584, 1510, 1431, 1250, 1021, 890, 827, 738; δ_H (400 MHz, $CDCl_3$) 1.67-1.88 (4H, m, CH_2), 2.01-2.12 (1H, m, CH_2), 2.17-2.31 (1H, m, CH_2), 3.53 (1H, br s, CCHC(S)), 3.81 (3H, s, OCH_3), 4.50 (1H, d, *J* 4.0 Hz, CH_2C*H*N), 4.83 (1H, s, =CH_2), 4.86 (1H, s, =CH_2), 6.95 (2H, d, *J* 9.0 Hz, Ar H), 7.44 (2H, d, *J* 9.0 Hz, Ar H); δ_C (100 MHz, $CDCl_3$) 18.3 (CH_2), 29.6 (CH_2), 32.1 (CH_2), 55.6 (CH_3, OCH_3), 61.0 (CH, C*C*HC(S)), 72.0 (CH, CH_2*C*HN), 101.4 (CH_2, =CH_2), 114.6 (2 x CH, Ar), 126.8 (2 x CH, Ar), 132.1 (C, ArCN), 152.0 (C, =C), 158.8 (C, ArCO), 203.8 (C, C(S)N); m/z (ES) 282.0922 ($[M+Na]^+$ $C_{15}H_{17}NNaOS$ requires 282.0929), 283 (12%), 282 (100).

(±)-(1*R*,5*S*)-6-(4-Methoxyphenyl)-8-methylene-7-(methylthio)-6-azabicyclo[3.2.1] octan-6-en-6-ium iodide **88**

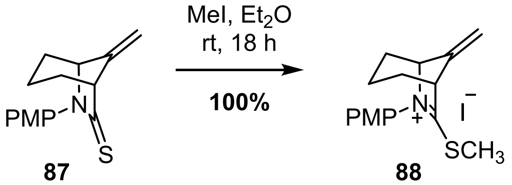

87 **88**

A novel compound prepared according to a modified literature procedure.[27] A solution of thiolactam **87** (94 mg, 0.36 mmol) and iodomethane (0.383 mL, 6.15 mmol) in Et_2O (0.77 mL) was stirred at rt. After 18 h, the reaction mixture was diluted with CH_2Cl_2 (1.0 mL) and concentrated under reduced pressure (3 times) to give iminium salt **88** (0.145 g, 100%) as a bright yellow foam. mp: degradation above 60 °C; ν_{max} (neat)/cm^{-1}: 3434, 2949, 1602, 1505, 1255, 1019, 845, 827; δ_H (400 MHz, $CDCl_3$) 1.66-1.84 (1H, m, CH_2), 1.85-1.97 (1H, m, CH_2), 1.99-2.18 (3H, m, CH_2), 2.24-2.36 (1H, m, CH_2), 3.06 (3H, s, SCH_3), 3.83 (3H, s, OCH_3), 4.80 (1H, br s, C*CH*$CSCH_3$)), 4.99 (1H, d, *J* 3.7 Hz, CH_2C*H*N), 5.28 (1H, s, =CH_2), 5.39 (1H, s, =CH_2), 7.04 (2H, d, *J* 9.0 Hz, Ar H), 7.47 (2H, d, *J* 9.0 Hz, Ar H); δ_C (100 MHz, $CDCl_3$) 18.3 (CH_3, SCH_3), 18.6 (CH_2), 28.4 (CH_2), 29.2 (CH_2), 54.0 (CH, CCH$CSCH_3$), 56.0 (CH_3, OCH_3), 76.8 (CH, CH_2CHN), 107.9 (CH_2, =CH_2), 116.1 (2 x CH, Ar), 125.8 (2 x CH, Ar), 127.5 (C, ArCN), 145.6 (C, =C), 161.6 (C, ArCO), 196.9 (C, =*C*SCH_3); m/z (ES) 292.1369 ($[M-I+H_2O]^+$ $C_{16}H_{22}NO_2S$ requires 292.1366), 314 (52%), 298 (100), 292 (40), 282 (39), 276 (72), 244 (56), 216 (62).

(±)-(1*R*,5*S*,7*R*)-7-Ally-6-(4-methoxyphenyl)-8-methylene-6-azabicyclo[3.2.1]octane **89**

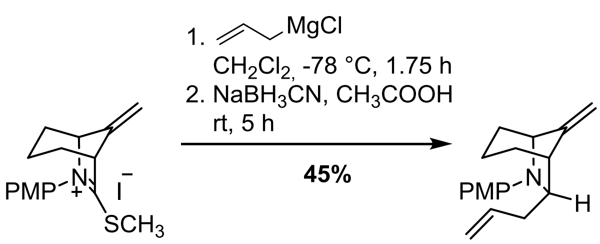

88 **89**

A novel compound prepared according to a modified literature procedure.[27] A solution of iminium salt **88** (73 mg, 0.18 mmol) in CH_2Cl_2 (2.0 mL) was cooled to -78 °C, allylmagnesium chloride (0.14 mL of a 2.0 M solution in THF, 0.28 mmol) was added dropwise over 1 min. After 1.75 h at -78 °C, $NaBH_3CN$ (0.36 mL of a 1.0 M solution in THF, 0.36 mmol) and CH_3COOH (1.0 mL) were added and the reaction mixture was allowed to warm to rt. After 5 h, NaOH (5 mL of a 10% aq. solution) was added and the reaction mixture was extracted with CH_2Cl_2 (3 x 2 mL). The combined organic extracts were washed with brine (5 mL), dried over $MgSO_4$, filtered, evaporated under reduced pressure and purified by column chromatography (Pet ether to Pet ether:Et_2O 9:1) to give bicyclic amine **89** (22 mg, 45%) as a colourless oil. R_f 0.68 (Pet ether:EtOAc 9:1); ν_{max} (neat)/cm^{-1}: 2941, 1506, 1236, 1040, 815; δ_H (400 MHz, $CDCl_3$) 1.62-1.79 (3H, m, CH_2), 1.84-1.97 (1H, m, CH_2), 1.99-2.15 (2H, m, CH_2), 2.32 (1H, ddd, *J* 7.5, 10.9 and 14.6 Hz, CHC*H₂*CH=CH_2), 2.65-2.78 (2H, m, CH_2C*H*C=CH_2 and CHC*H₂*CH=CH_2), 3.52-3.62 (1H, m, CHC*H*N), 3.77 (3H, s, OCH_3), 4.00 (1H, d, *J* 4.2 Hz, CH_2C*H*N), 4.57 (1H, s, =CH_2), 4.64 (1H, s, =CH_2), 5.09-5.26 (2H, m, CH=C*H₂*), 5.87 (1H, ddt, *J* 7.1, 10.2 and 17.1 Hz, C*H*=CH_2), 6.61 (2H, d, *J* 9.0 Hz, Ar H), 6.85 (2H, d, *J* 9.0 Hz, Ar H); δ_C (100 MHz, $CDCl_3$) 19.8 (CH_2), 29.4 (CH_2), 31.6 (CH_2, *C*H₂CH=CH_2), 35.7 (CH_2), 44.2 (CH, *C*HC=CH_2), 56.1 (CH_3, OCH_3), 61.6 (CH, CH*C*HN), 68.2 (CH, CH_2*C*HN), 99.1 (CH_2, C=*C*H₂), 114.6 (2 x CH, Ar), 115.0 (2 x CH, Ar), 116.9 (CH_2, CH=*C*H₂), 135.6 (CH, *C*H=CH_2),

143.0 (C, Ar*C*N), 151.7 (C, Ar*C*O), 154.3 (C, =C); m/z (ES) 270.1853 ($[M+H]^+$ $C_{18}H_{24}NO$ requires 270.1852), 270 (100%), 228 (37), 199 (97).

(±)-(3a*R*,5a*S*,8a*S*)-5-Benzyl-3-mesityl-5,5a,6,7,8,8a-hexahydro-4*H*-azeto[3',2':1,6] benzo[1,2-*d*] isoxazol-4-onemesitaldehyde oxime **91**

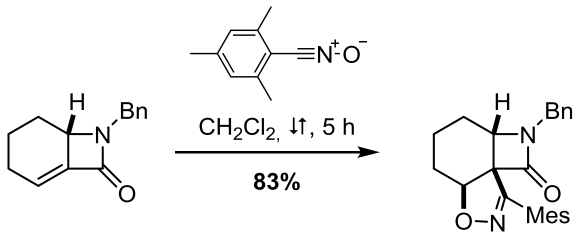

57c **91**

A novel compound prepared according to a modified literature procedure.[29] A solution of conjugated lactam **57c** (66 mg, 0.31 mmol) in CH_2Cl_2 (3.1 mL) was treated with nitrile oxide **90**[4b] (55 mg, 0.34 mmol) and heated to reflux. After 5 h, the reaction mixture was evaporated under reduced pressure and the crude residue obtained was purified by column chromatography (Pet ether to Pet ether:EtOAc 7:3) to give oxazoline **91** (96 mg, 83%) as a white solid. mp 123-127 °C; R_f 0.55 (Pet ether:EtOAc 7:3); ν_{max} (neat)/cm^{-1}: 2921, 1751, 1450, 1407, 871, 851, 734, 700; δ_H (400 MHz, $CDCl_3$) 1.33-1.65 (4H, m, CH_2), 1.79-1.90 (1H, m, CH_2), 2.10 (3H, s, CH_3), 2.26-2.36 (1H, m, CH_2), 2.38 (3H, s, CH_3), 2.43 (3H, s, CH_3), 3.56 (1H, dd, *J* 1.5 and 5.7 Hz, CHN), 3.89, 4.65 (2H, ABq, *J* 15.9 Hz, NCH_2Ph), 5.13 (1H, dd, *J* 7.1 and 9.2 Hz, CHO), 6.69 (2H, d, *J* 7.4 Hz, Ar H), 6.89 (1H, s, Ar H), 7.01 (1H, s, Ar H), 7.09 (2H, t, *J* 7.5 Hz, Ar H), 7.19 (1H, t, *J* 7.4 Hz, Ar H); δ_C (100 MHz, $CDCl_3$) 15.0 (CH_2), 20.1 (CH_3), 20.3 (CH_3), 21.2 (CH_3), 24.9 (CH_2), 28.2 (CH_2), 43.5 (CH_2, NCH_2Ph), 52.9 (CH, CH_2*C*HN), 71.8 (C, *C*C(O)), 79.6 (CH, CH_2*C*HO), 123.9 (C, Ar), 127.1 (2 x CH, Ar), 127.5 (CH, Ar), 128.4 (CH, Ar), 128.7 (2 x CH, Ar), 129.0 (CH, Ar), 134.4 (C, Ar), 137.1 (C, Ar), 138.6 (C, Ar), 139.2 (C, Ar), 154.1 (C, C(N)), 165.9 (C, C(O)N); m/z (ES) 375.2071 ($[M+H]^+$ $C_{24}H_{27}N_2O_2$ requires 375.2067), 771 (10%), 749 (33), 397 (26), 375 (100), 242 (47).

References

1. Grainger, R. S.; Innocenti, P. *Angew. Chem. Int. Ed.* **2004**, *43*, 3445-3448.
2. Grainger, R. S.; Betou, M.; Male, L.; Pitak, M. B.; Coles, S. J. *Org. Lett.* **2012**, *14*, 2234-2237.
3. Ahmed, S.; Baker, L. A.; Grainger, R. S.; Innocenti, P.; Quevedo, C. E. *J. Org. Chem.* **2008**, *73*, 8116-8119.
4. a) Jefford, C. W.; Mahajan, S.; Waslyn, J.; Waegell, B. *J. Am. Chem. Soc.* **1965**, *87*, 2183-2190; b) Bode, J. W.; Hachisu, Y.; Matsuura, T.; Suzuki, K. *Tetrahedron Lett.* **2003**, *44*, 3555-3558
5. Adams, H.; C. Anderson, J.; Bell, R.; Neville Jones, D.; R. Peel, M.; C. O. Tomkinson, N. *J. Chem. Soc., Perkin Trans. 1* **1998**, 3967-3974.
6. Williams, E. L. *Synth. Commun.* **1992**, *22*, 1017-1021.
7. Shing, T. K. M.; Lee, C. M.; Lo, H. Y. *Tetrahedron* **2004**, *60*, 9179-9197.
8. Heathcock, C. H.; DelMar, E. G.; Graham, S. L. *J. Am. Chem. Soc.* **1982**, *104*, 1907-1917.
9. Kita, Y.; Yoshida, Y.; Mihara, S.; Fang, D.-F.; Higuchi, K.; Furukawa, A.; Fujioka, H. *Tetrahedron Lett.* **1997**, *38*, 8315-8318.
10. Innocenti, P. *PhD Thesis*, University of London, **2004**.
11. Kittaka, A.; Tanaka, H.; Odanaka, Y.; Ohnuki, K.; Yamaguchi, K.; Miyasaka, T. *J. Org. Chem.* **1994**, *59*, 3636-3641.
12. Gibson, S. *PhD Thesis*, University of Reading, UK, **2004**.
13. Ranu, B. C.; Dutta, J.; Guchhait, S. K. *J. Org. Chem.* **2001**, *66*, 5624-5626.
14. Ranu, B. C.; Majee, A.; Sarkar, A. *J. Org. Chem.* **1998**, *63*, 370-373.
15. Hayashi, T.; Sakurai, A.; Oishi, T. *Chem. Lett.* **1977**, *6*, 1483-1486.
16. Jiang, H.; Liu, B.; Li, Y.; Wang, A.; Huang, H. *Org. Lett.* **2011**, *13*, 1028-1031.
17. Davis, F. A.; Mancinelli, P. A.; Balasubramanian, K.; Nadir, U. K. *J. Am. Chem. Soc.* **1979**, *101*, 1044-1045.
18. Ihara, M.; Haga, Y.; Yonekura, M.; Oshawa, T.; Fukumoto, K.; Kametani, T. *J. Am. Chem. Soc.* **1983**, *105*, 7345-7352.
19. Gentric, L.; Le Goff, X.; Ricard, L.; Hanna, I. *J. Org. Chem.* **2009**, *74*, 9337-9344.
20. O'Mahony, G.; Nieuwenhuyzen, M.; Armstrong, P.; Stevenson, P. J. *J. Org. Chem.* **2004**, *69*, 3968-3971.
21. Matsuo, J.-i.; Takeuchi, K.; Ishibashi, H. *Org. Lett.* **2008**, *10*, 4049-4052.
22. Kumari, N.; Vankar, Y. D. *Org. Biomol. Chem.* **2009**, *7*, 2104-2109.
23. van Woerden, H. F. *Chem. Rev.* **1963**, *63*, 557-571.
24. DeCamp, A. E.; Mills, S. G.; Kawaguchi, A. T.; Desmond, R.; Reamer, R. A.; DiMichele, L.; Volante, R. P. *J. Org. Chem.* **1991**, *56*, 3564-3571.
25. Nemoto, H.; Miyata, J.; Hakamata, H.; Nagamochi, M.; Fukumoto, K. *Tetrahedron* **1995**, *51*, 5511-5522.
26. Oppolzer, W.; Radinov, R. N. *Tetrahedron Lett.* **1988**, *29*, 5645-5648.
27. Klaver, W. J.; Hiemstra, H.; Speckamp, W. N. *J. Am. Chem. Soc.* **1989**, *111*, 2588-2595.
28. Michael, J. P.; Hosken, G. D.; Howard, A. S. *Tetrahedron* **1988**, *44*, 3025-3036.
29. Bode, J. W.; Carreira, E. M. *J. Org. Chem.* **2001**, *66*, 6410-6424.

(±)-(1*R*,6*S*)-7-(4-Methoxyphenyl)-7-azabicyclo[4.2.0]oct-2-en-8-one **16**; $CDCl_3$, 400 MHz

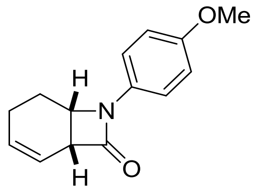

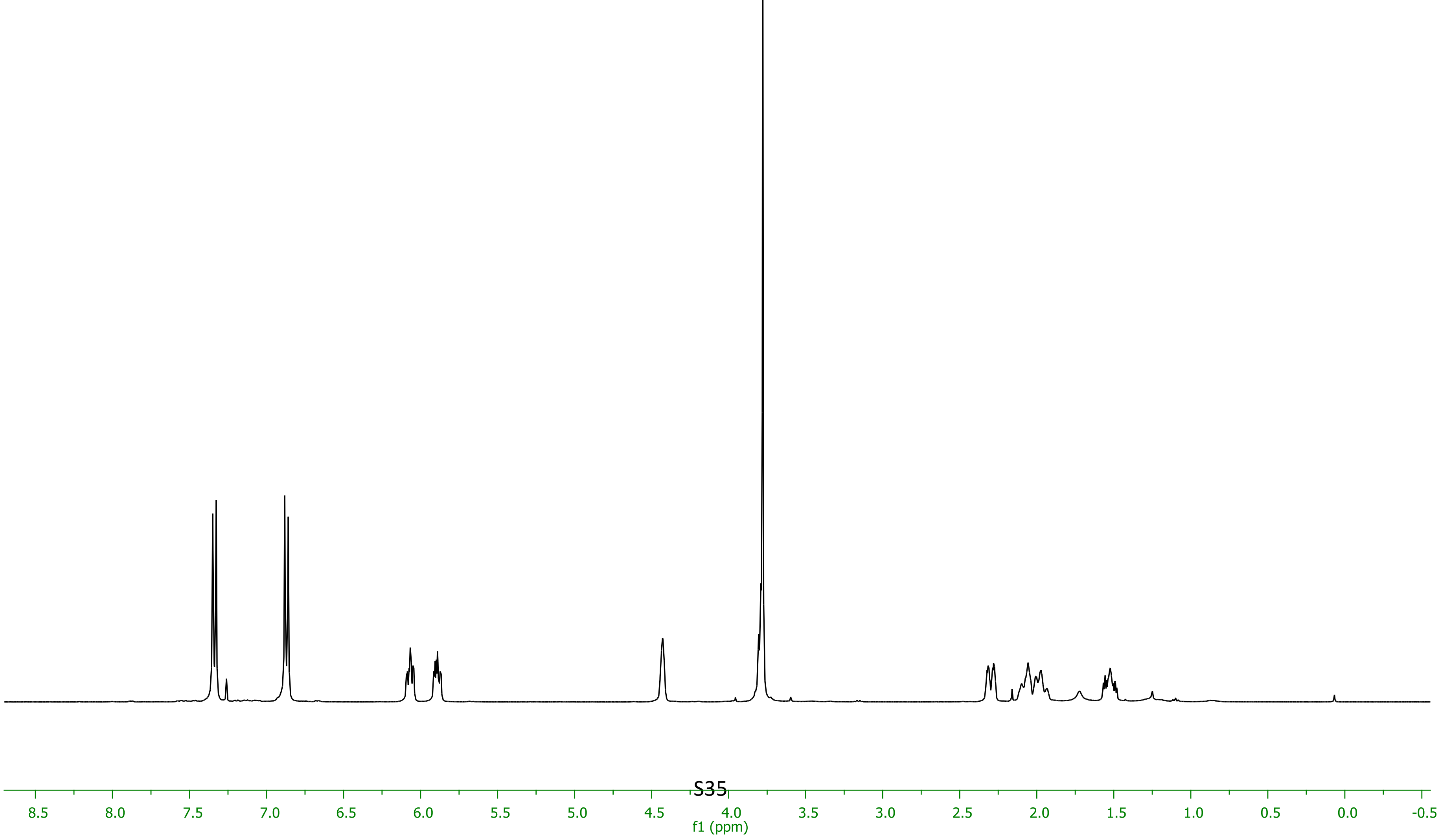

(±)-(1*R*,6*S*)-7-(4-Methoxyphenyl)-7-azabicyclo[4.2.0]oct-2-en-8-one **16;** $CDCl_3$, 100 MHz

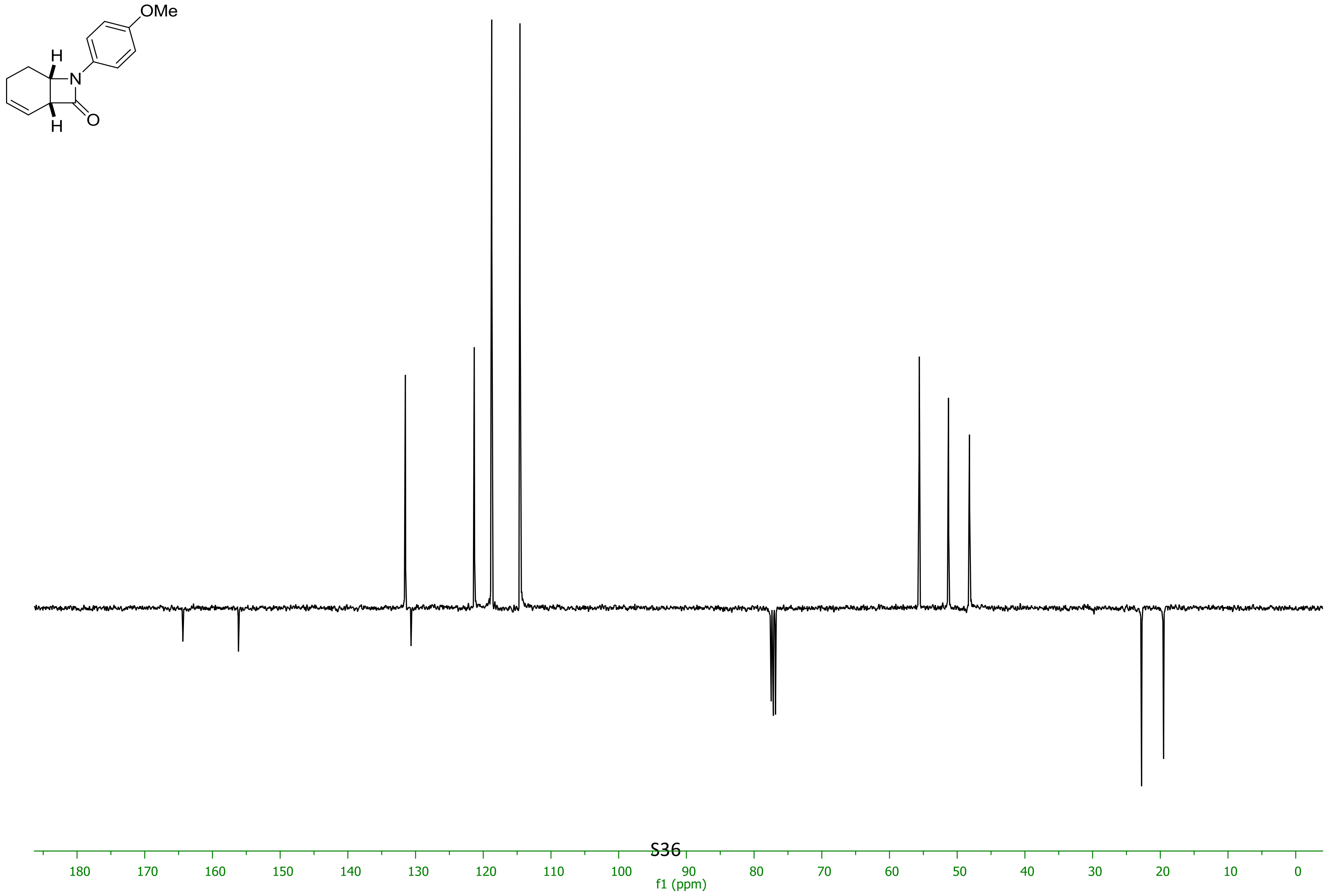

(±)-(1*R*,3*S*,7*S*)-8-(4-Methoxyphenyl)-2-oxa-8-azatricyclo[5.2.0.0^{1,3}]nonan-9-one **17**; $CDCl_3$, 300 MHz

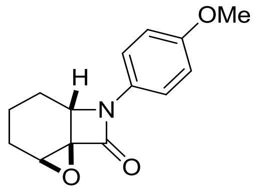

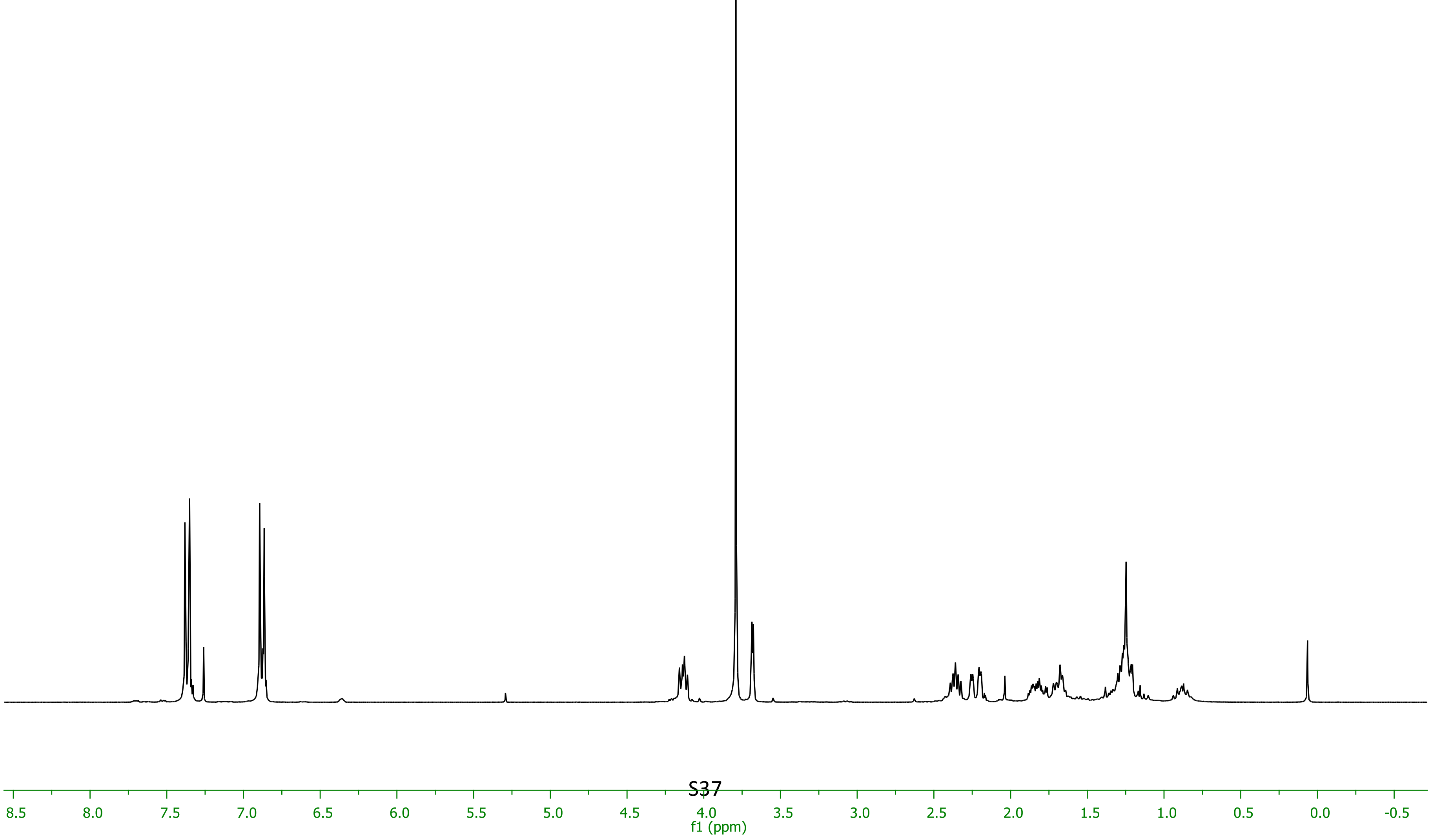

(±)-(1*R*,3*S*,7*S*)-8-(4-Methoxyphenyl)-2-oxa-8-azatricyclo[5.2.0.0^{1,3}]nonan-9-one **17;** $CDCl_3$, 100 MHz

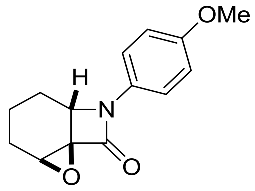

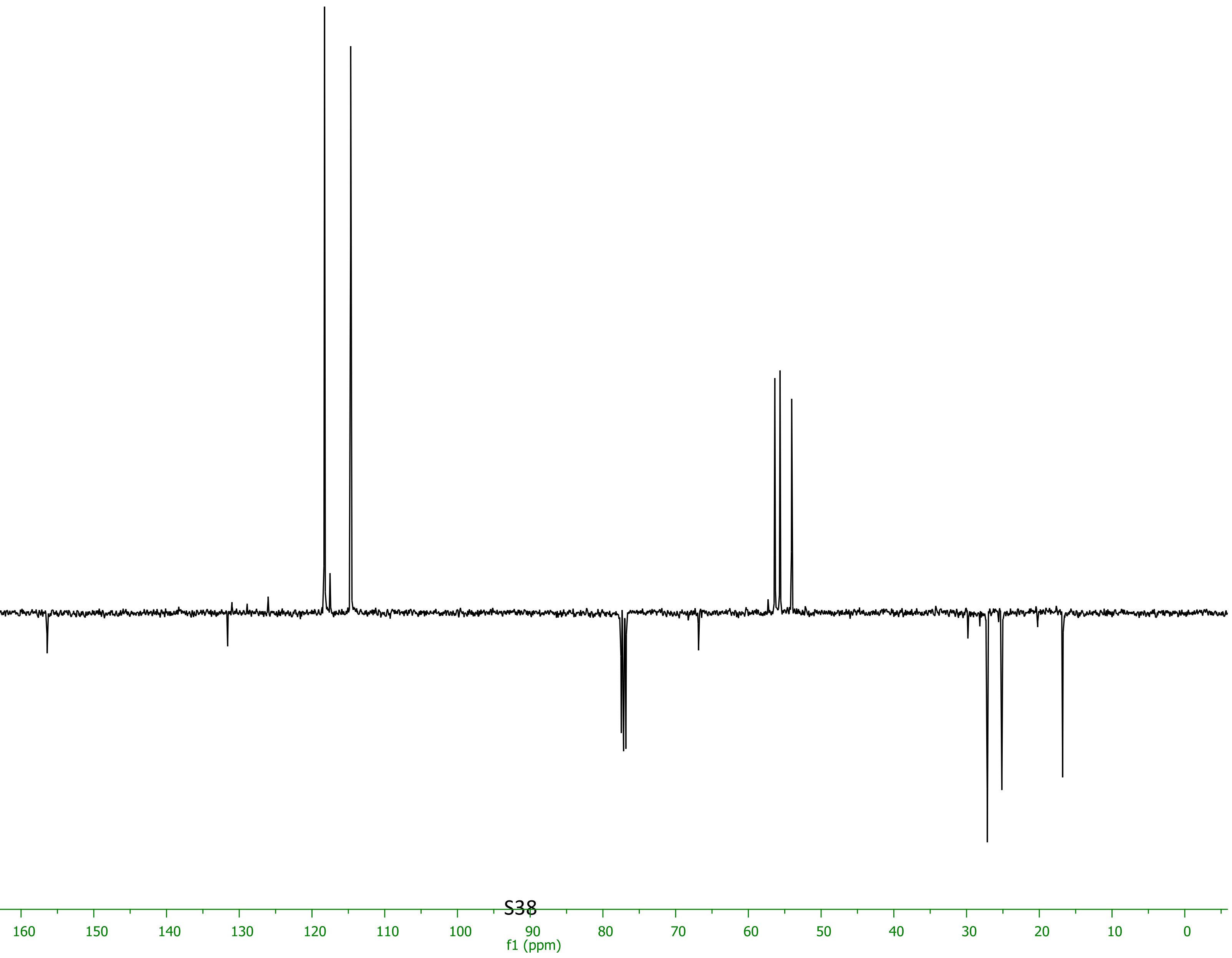

(±)-(1*S*,2*R*,6*S*)-2-Chloro-1-hydroxy-7-(4-methoxyphenyl)-7-azabicyclo[4.2.0]octan-8-one **19**; $CDCl_3$, 400 MHz

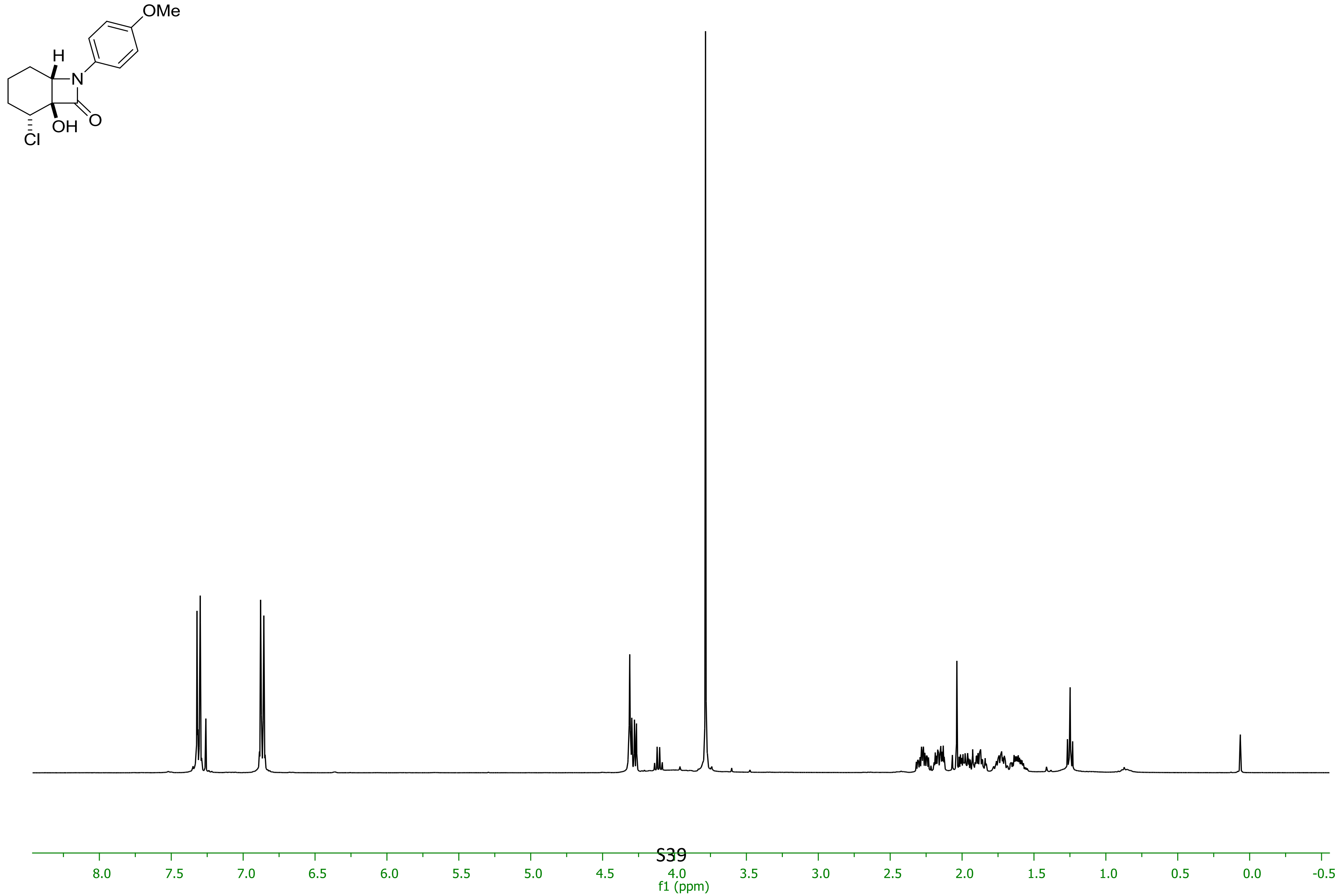

(±)-(1*S*,2*R*,6*S*)-2-Chloro-1-hydroxy-7-(4-methoxyphenyl)-7-azabicyclo[4.2.0]octan-8-one **19*;*** $CDCl_3$, 100 MHz

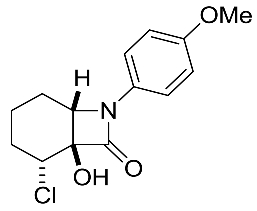

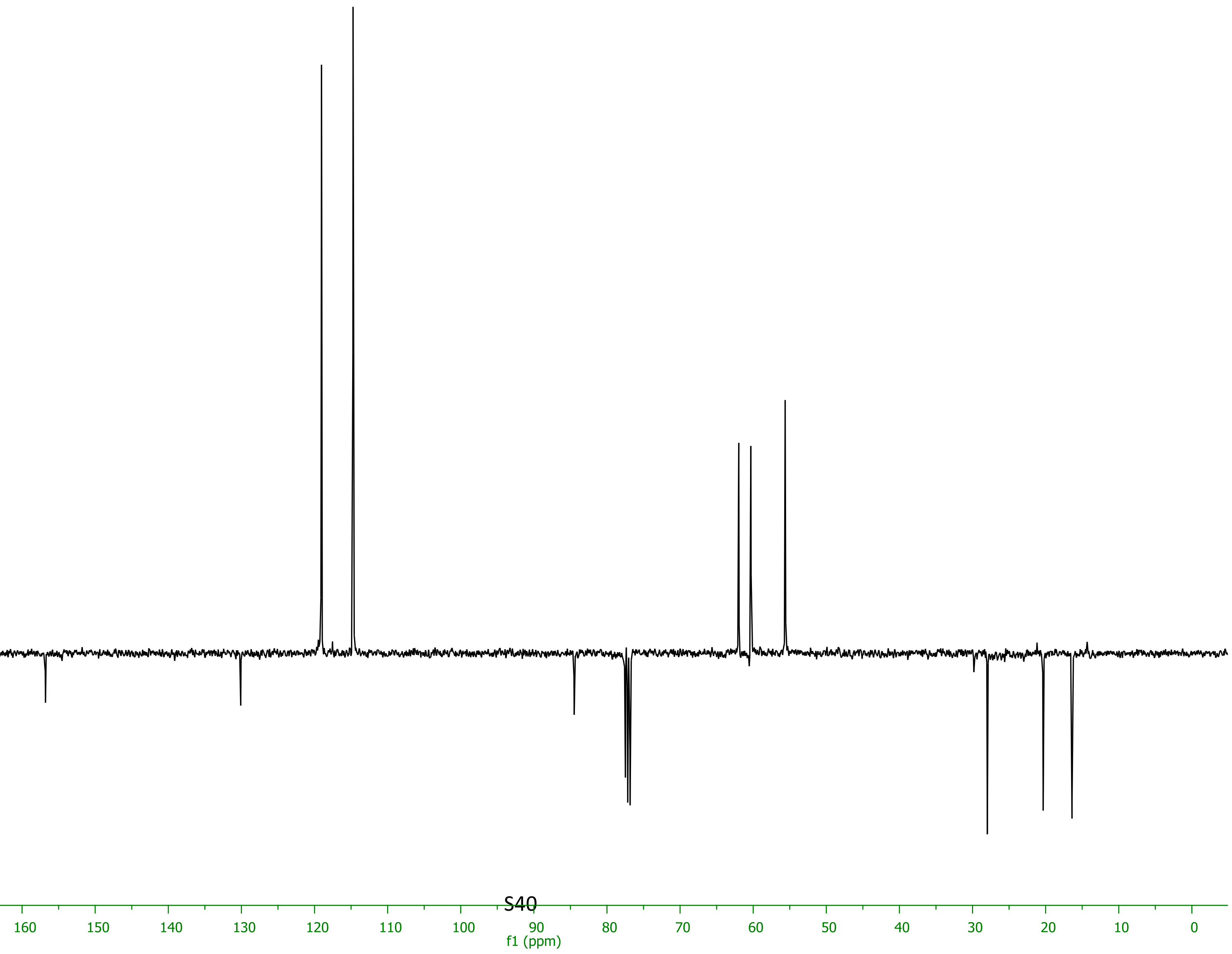

(±)-(1*R*,2*R*,6*S*)-1-Hydroxy-7-(4-methoxyphenyl)-8-oxo-7-azabicyclo[4.2.0]octan-2-yl 4-methyl benzene sulfonate **20**; $CDCl_3$, 400 MHz

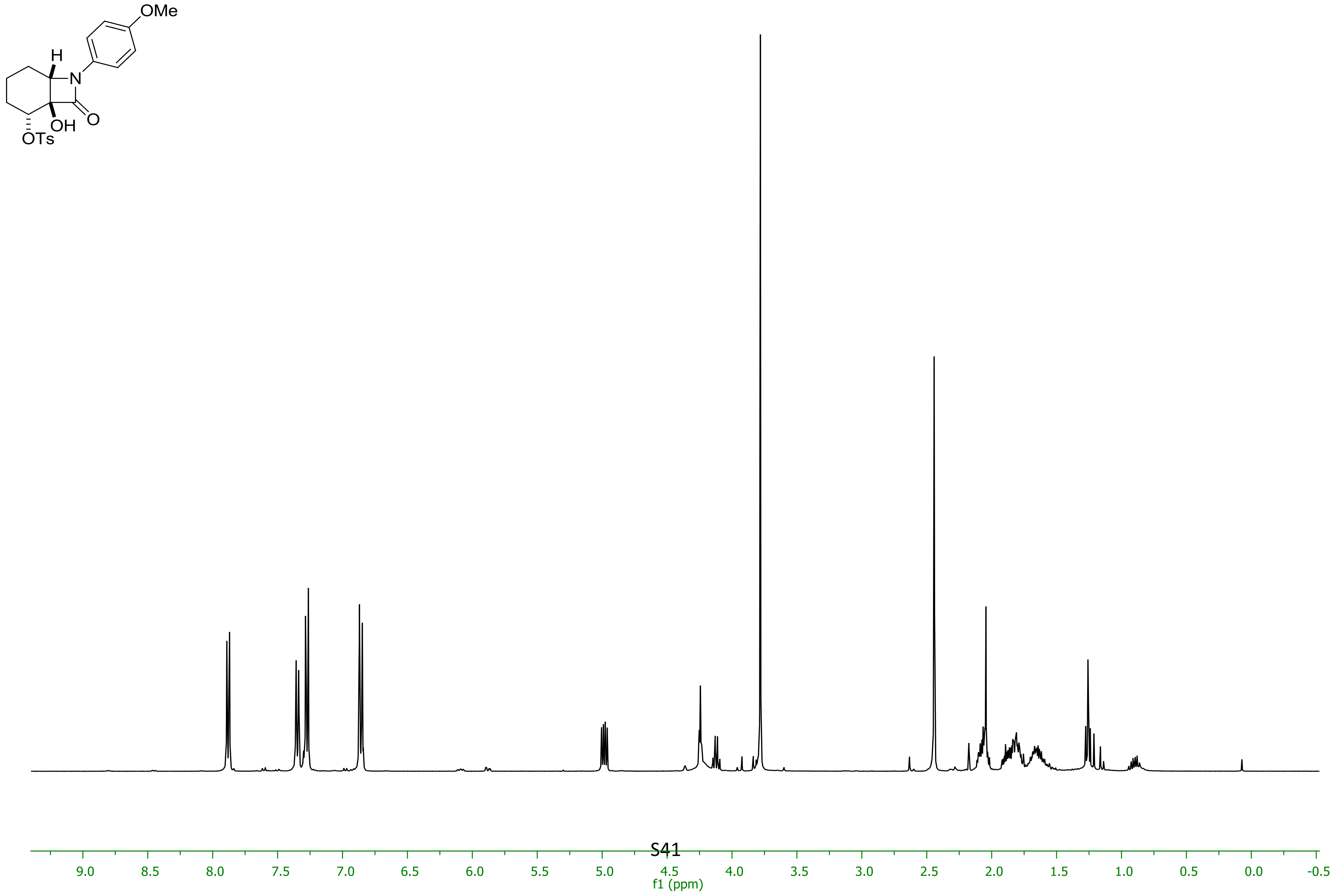

(±)-(1*R*,2*R*,6*S*)-1-Hydroxy-7-(4-methoxyphenyl)-8-oxo-7-azabicyclo[4.2.0]octan-2-yl 4-methyl benzene sulfonate **20**; $CDCl_3$, 100 MHz

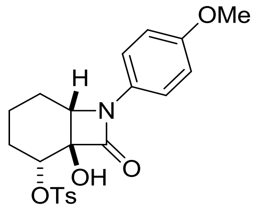

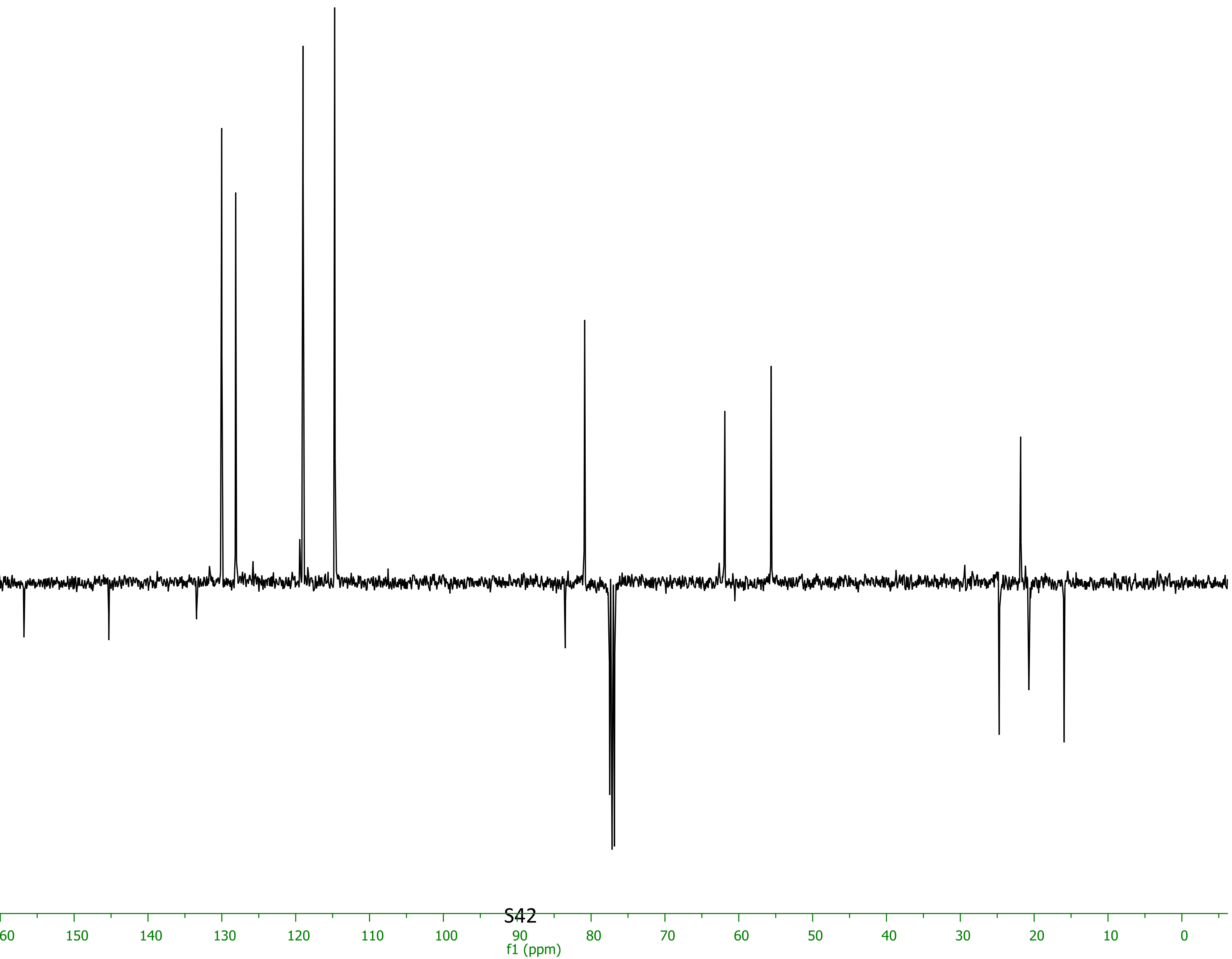

(±)-(1*R*,2*S*,6*S*)-2-Hydroxy-7-(4-methoxyphenyl)-8-oxo-7-azabicyclo[4.2.0]octan-1-yl methane sulfonate **24**; $CDCl_3$, 400 MHz

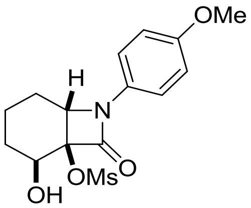

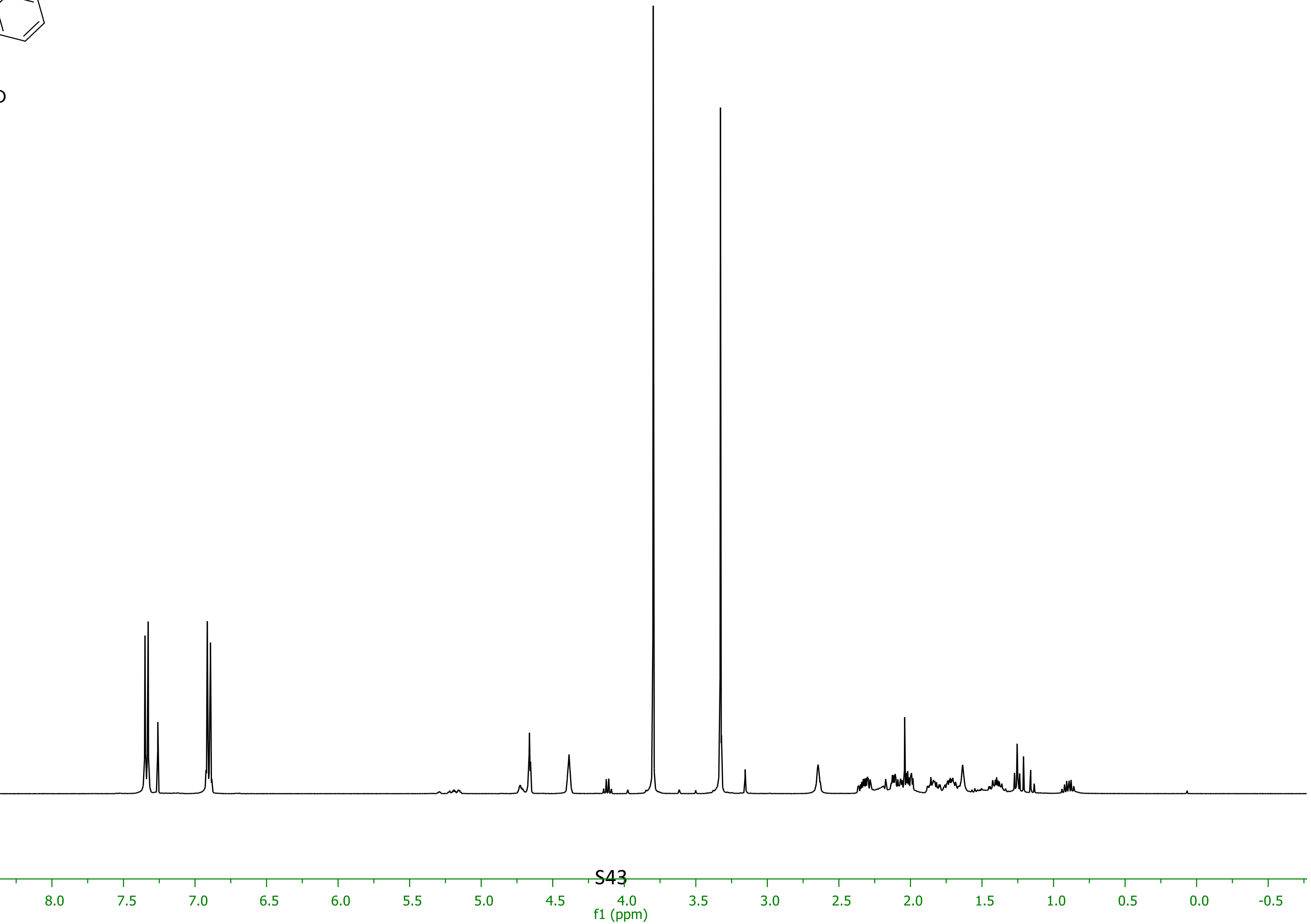

(±)-(1*R*,2*S*,6*S*)-2-Hydroxy-7-(4-methoxyphenyl)-8-oxo-7-azabicyclo[4.2.0]octan-1-yl methane sulfonate **24**; $CDCl_3$, 100 MHz

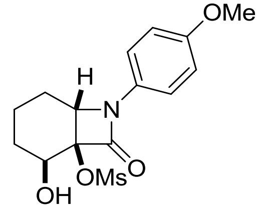

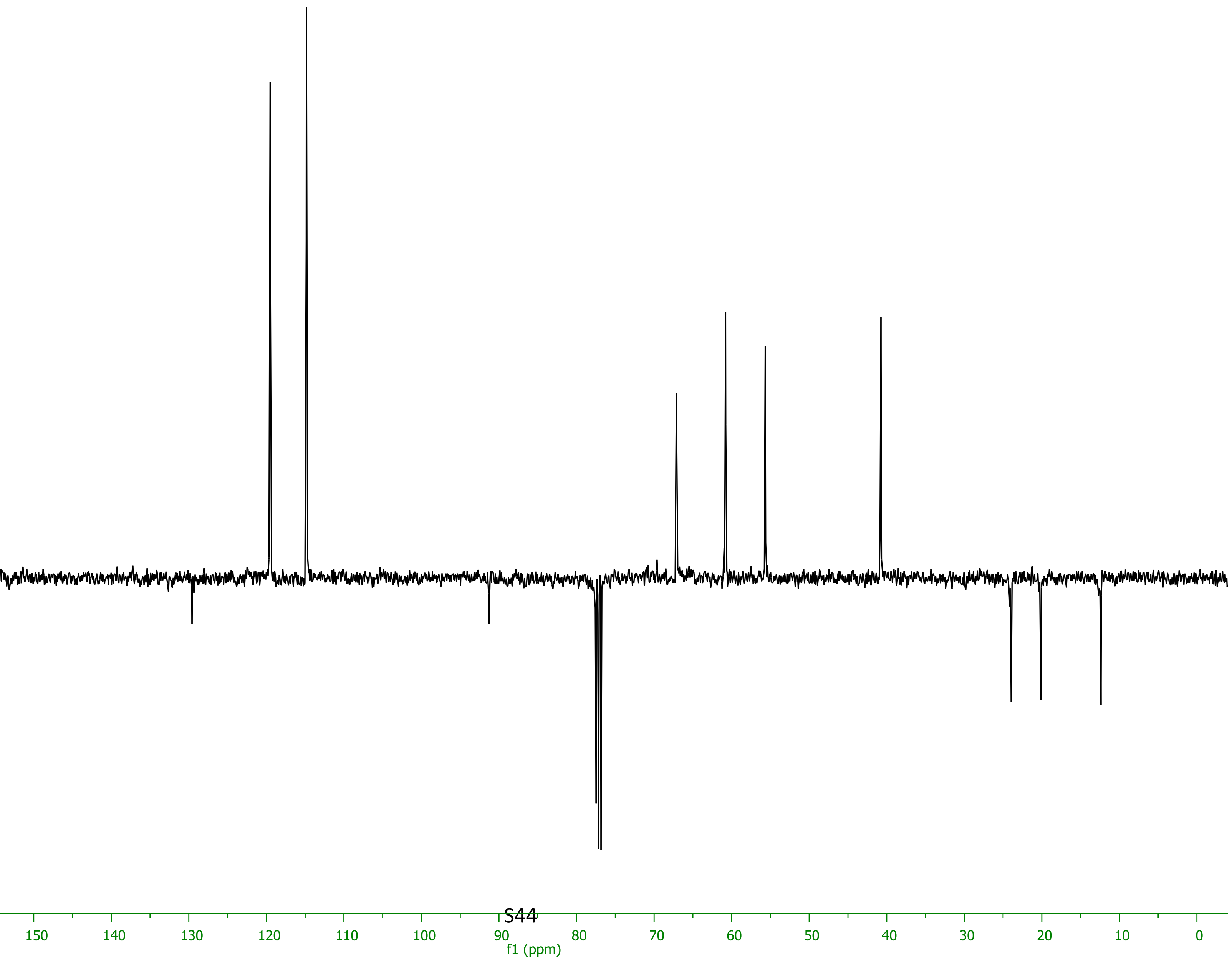

(±)-(1*R*,2*S*,6*S*)-7-(4-methoxyphenyl)-8-oxo-7-azabicyclo[4.2.0]octan-1,2-diyl dimethanesulfonate **25**; $CDCl_3$, 400 MHz

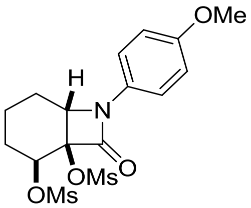

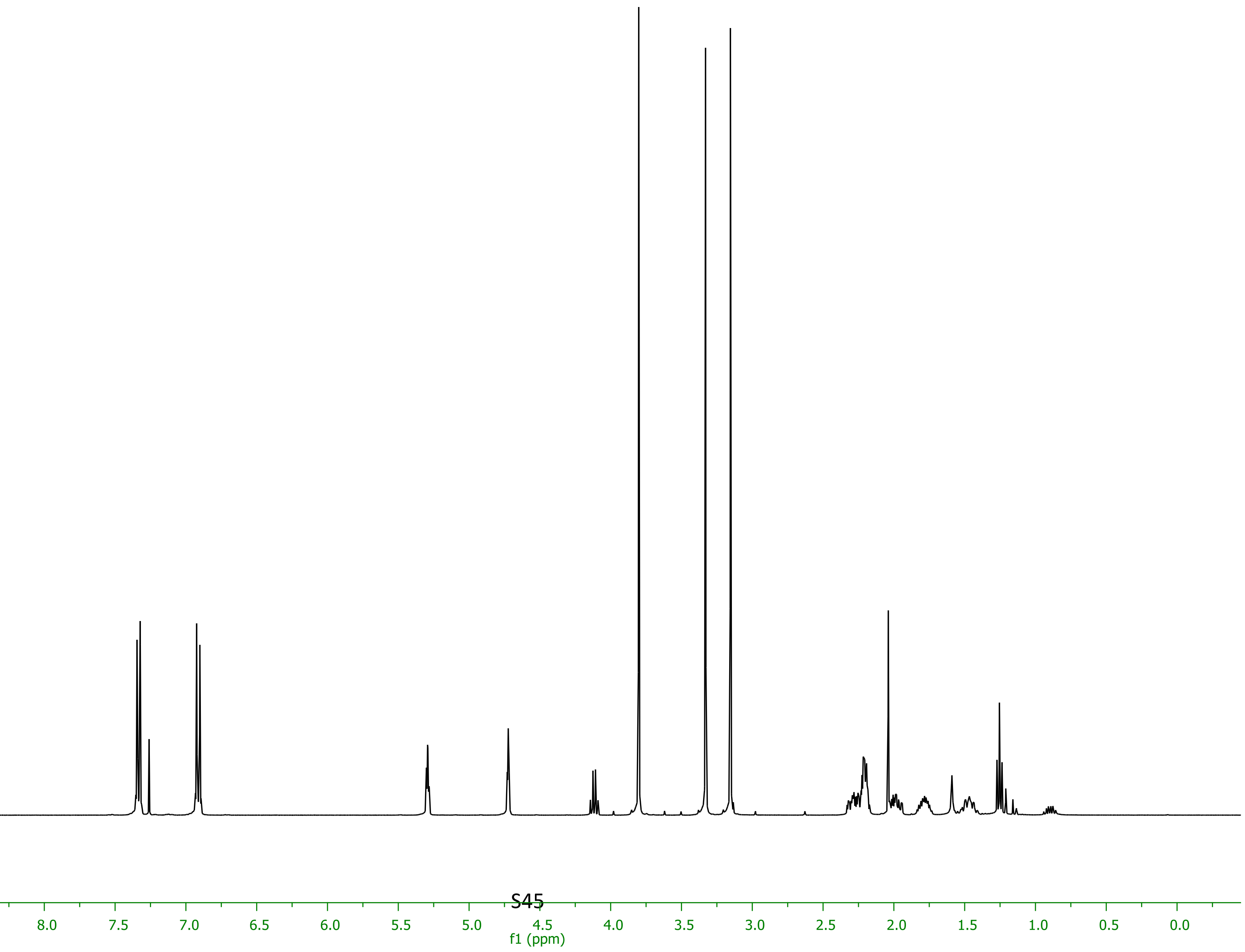

(±)-(1*R*,2*S*,6*S*)-7-(4-methoxyphenyl)-8-oxo-7-azabicyclo[4.2.0]octan-1,2-diyl dimethanesulfonate **25**; $CDCl_3$, 100 MHz

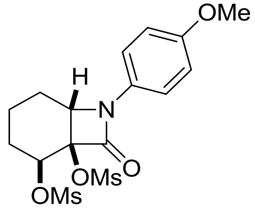

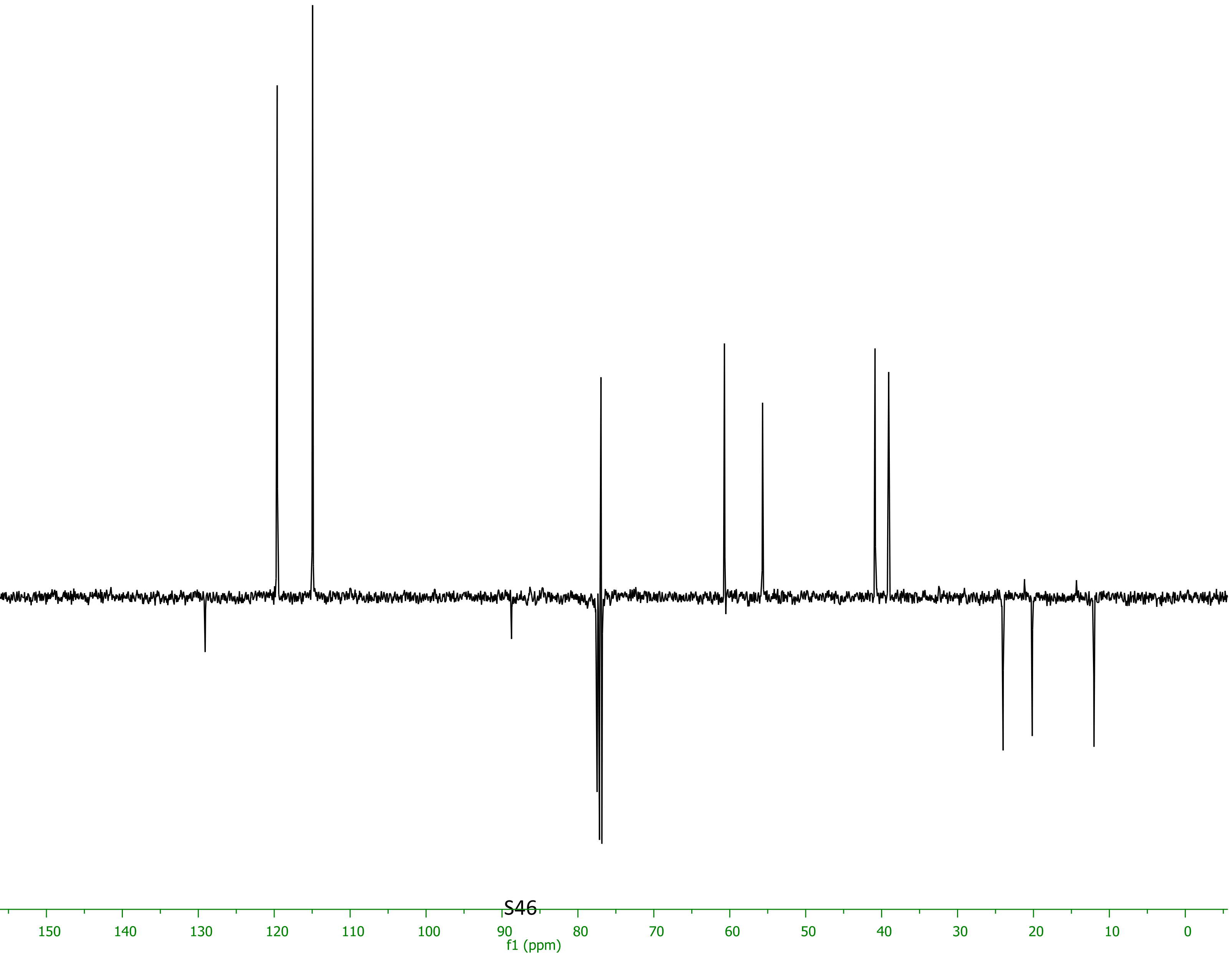

(±)-(1*R*,2*S*,6*S*)-2-Hydroxy-7-(4-methoxyphenyl)-8-oxo-7-azabicyclo[4.2.0]octan-1-yl methylbenzene sulfonate **26**; $CDCl_3$, 300 MHz

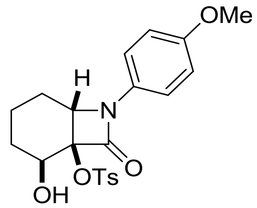

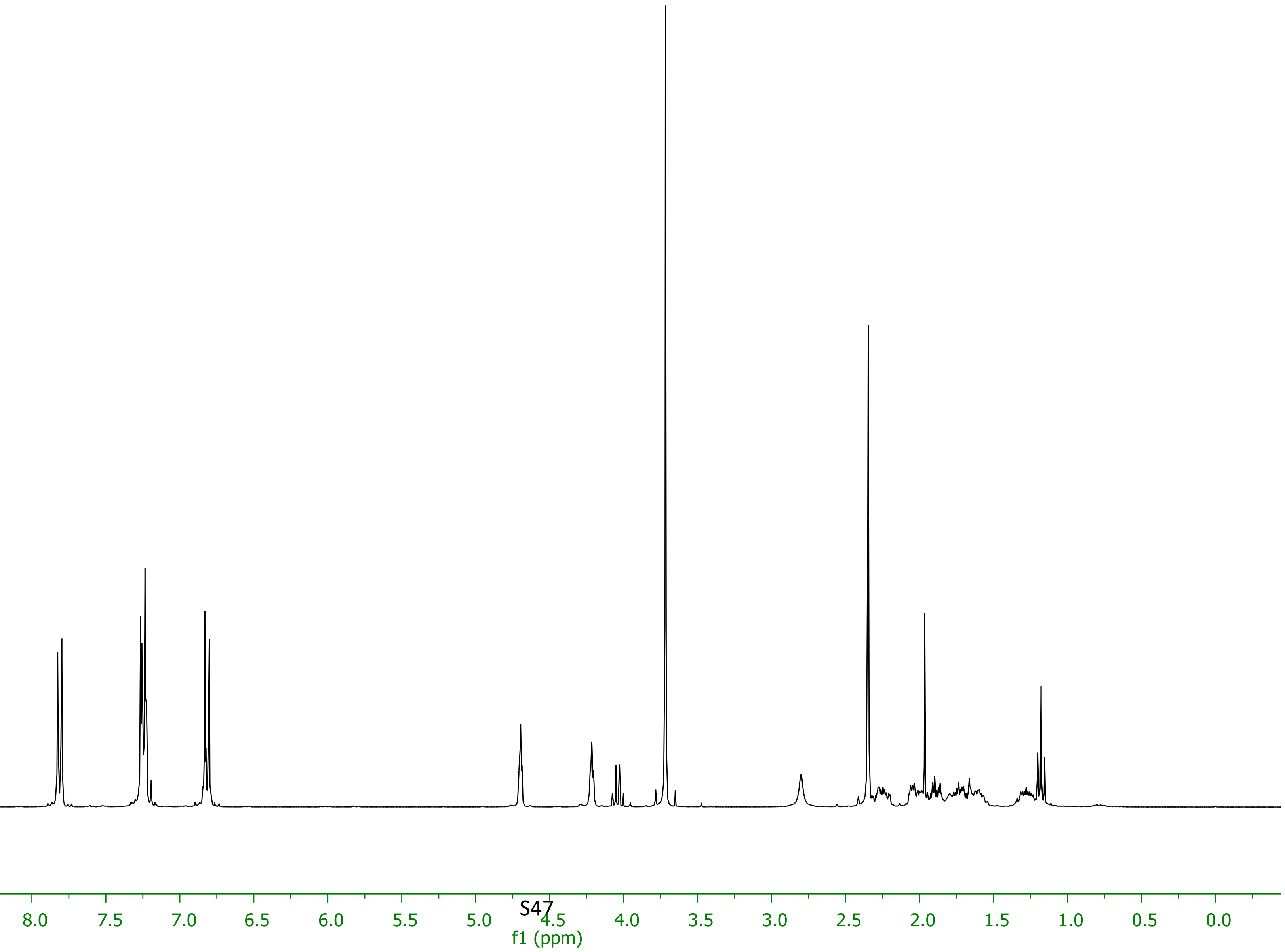

(±)-(1*R*,2*S*,6*S*)-2-Hydroxy-7-(4-methoxyphenyl)-8-oxo-7-azabicyclo[4.2.0]octan-1-yl methylbenzene sulfonate **26**; $CDCl_3$, 100 MHz

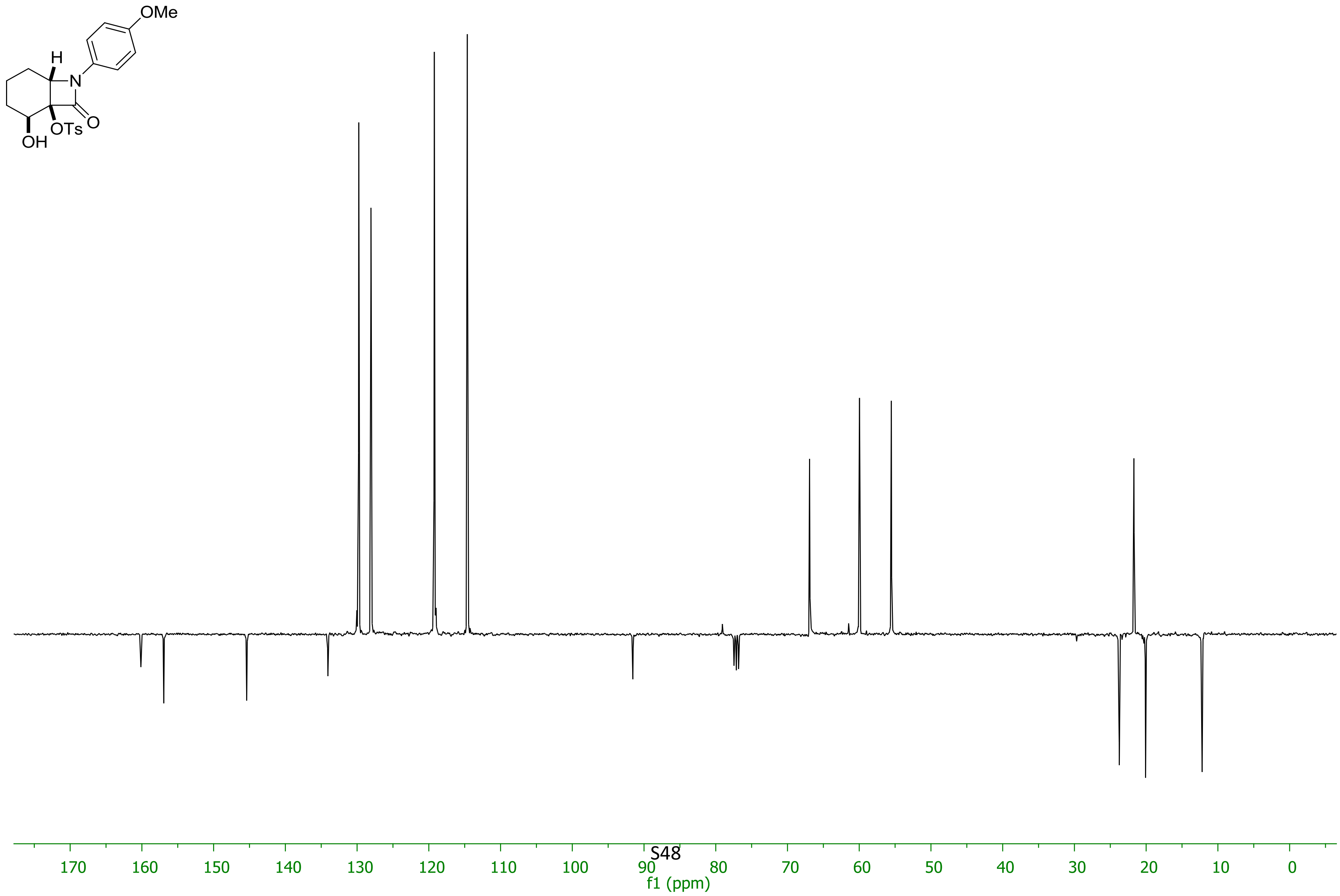

(±)-(1*R*,2*S*,6*S*)-1-Hydroxy-7-(4-methoxyphenyl)-8-oxo-7-azabicyclo[4.2.0]octan-2-yl methylbenzene sulfonate **27**; $CDCl_3$, 300 MHz

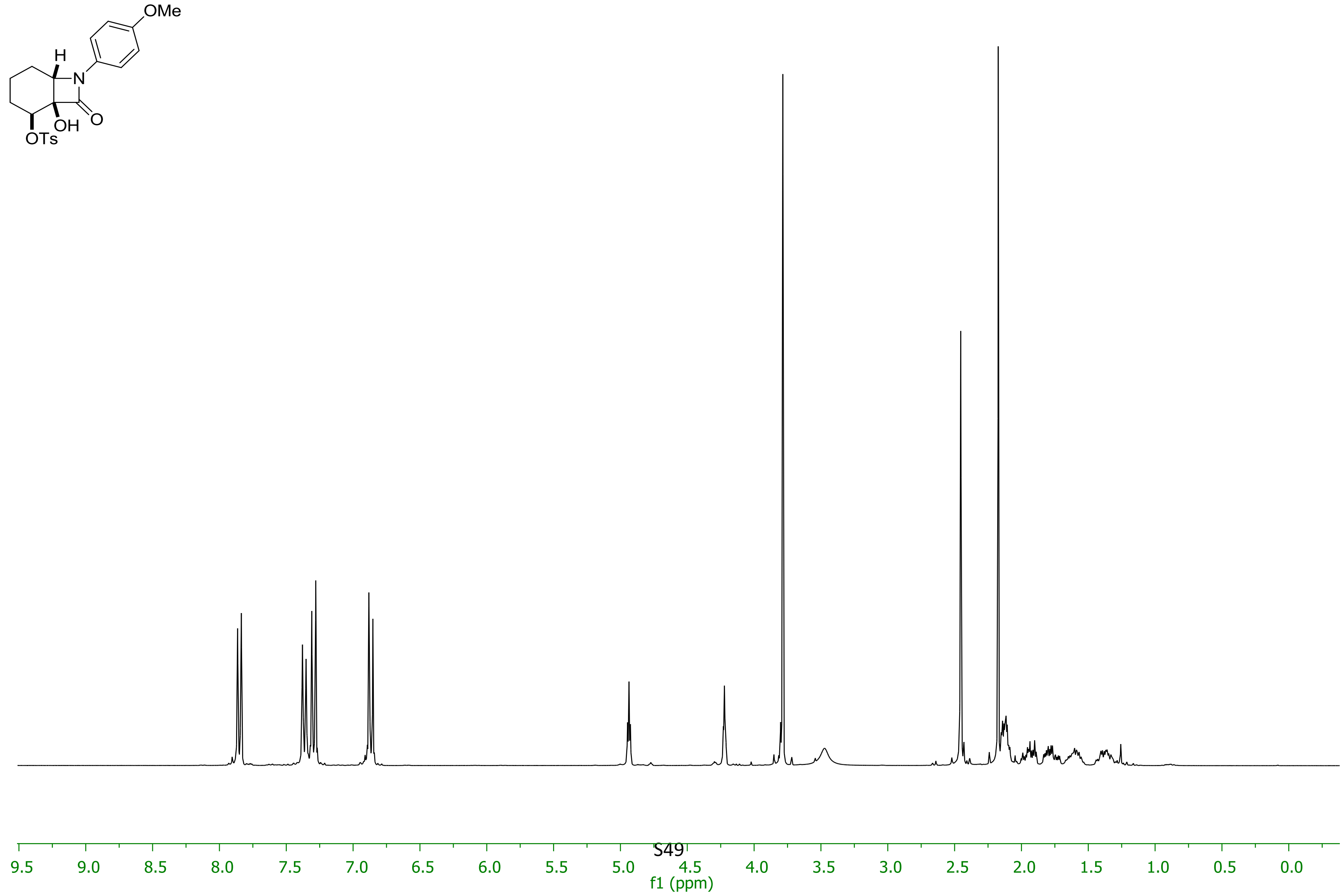

(±)-(1*R*,2*S*,6*S*)-1-Hydroxy-7-(4-methoxyphenyl)-8-oxo-7-azabicyclo[4.2.0]octan-2-yl methylbenzene sulfonate **27**; $CDCl_3$, 100 MHz

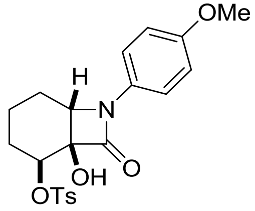

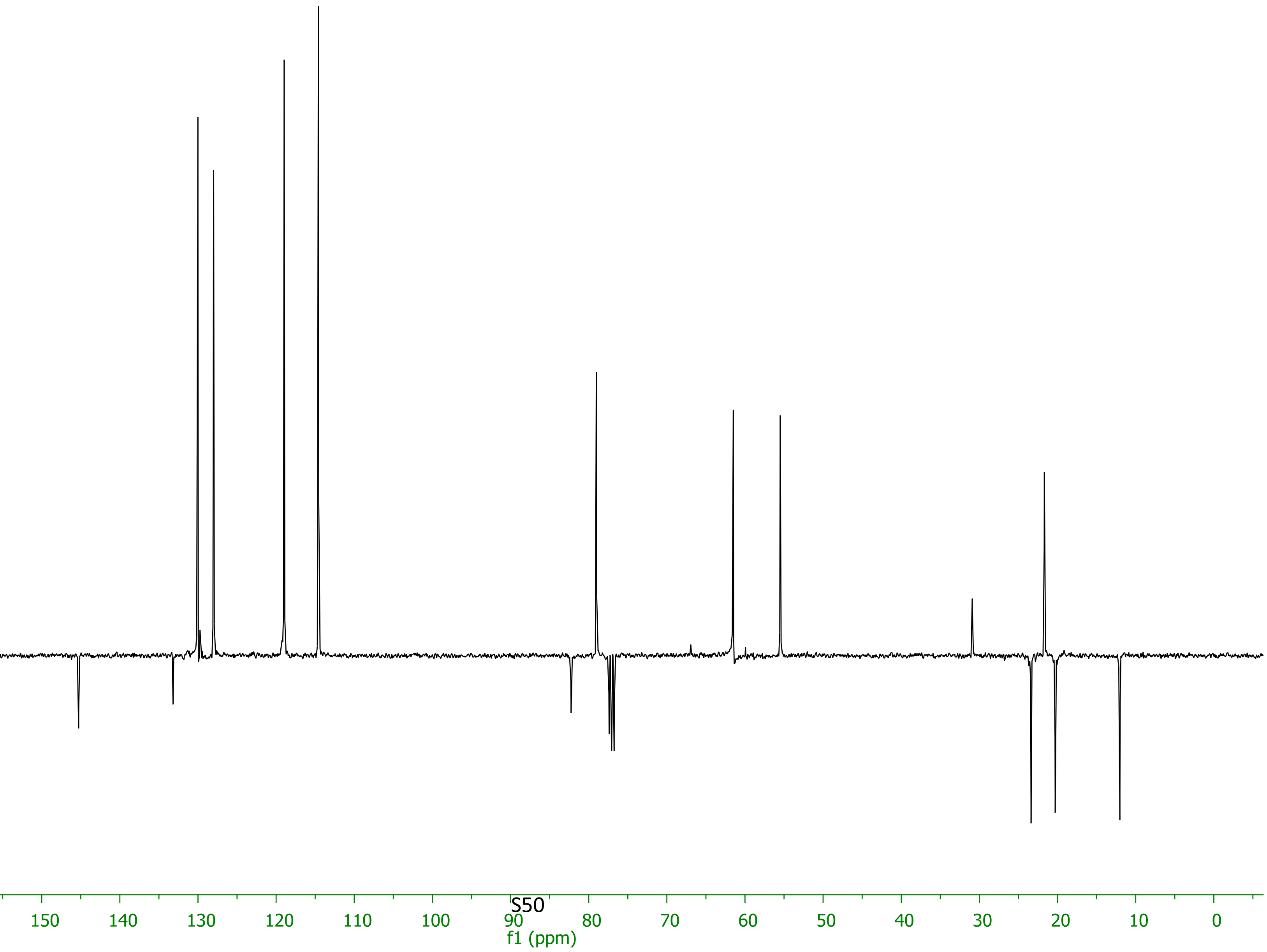

(±)-(1*R*,2*S*,6*S*)-7-(4-methoxyphenyl)-8-oxo-7-azabicyclo [4.2.0]octan-1,2-diyl bis(4-methylbenzenesulfonate) **28**; $CDCl_3$, 400 MHz

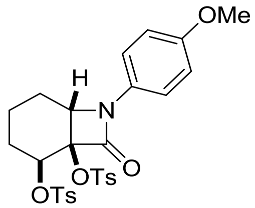

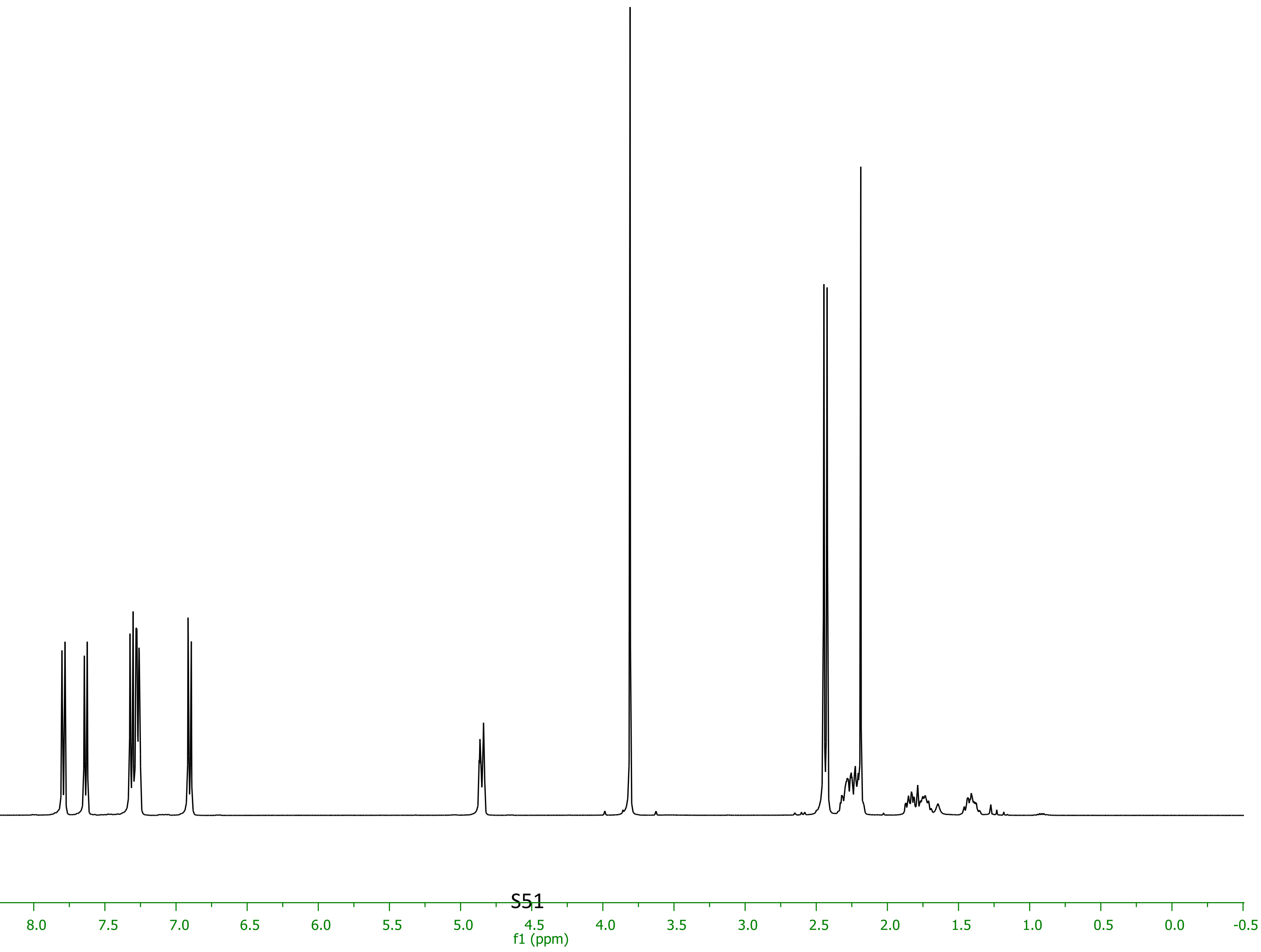

(±)-(1*R*,2*S*,6*S*)-7-(4-methoxyphenyl)-8-oxo-7-azabicyclo [4.2.0]octan-1,2-diyl bis(4-methylbenzenesulfonate) **28**; $CDCl_3$, 100 MHz

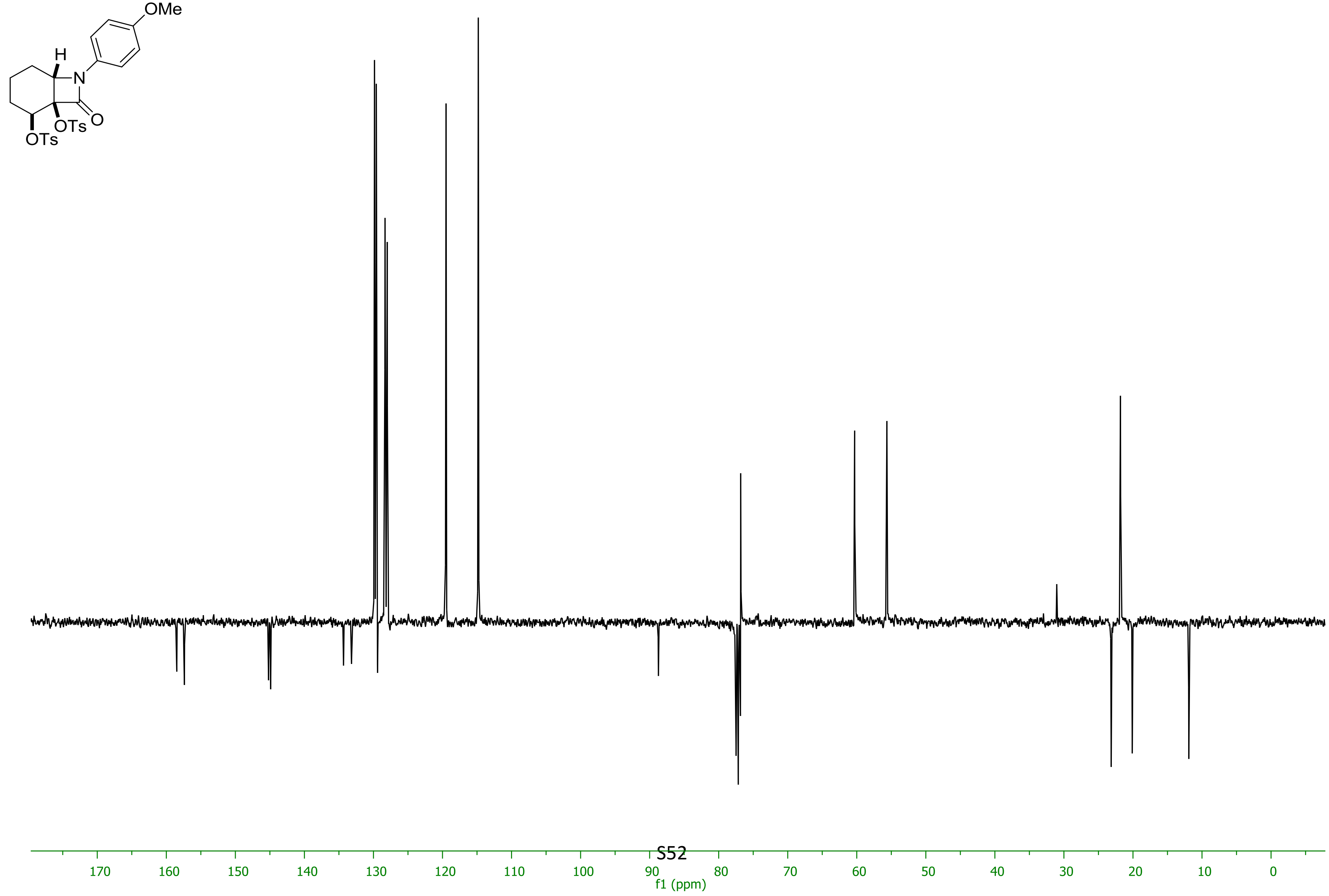

(±)-(1*R*,2*S*,6*S*)-1-Hydroxy-7-(4-methoxyphenyl)-8-oxo-7-azabicyclo[4.2.0]octan-2-yl formate **29**; $CDCl_3$, 400 MHz

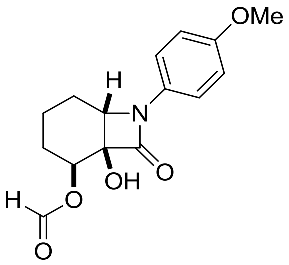

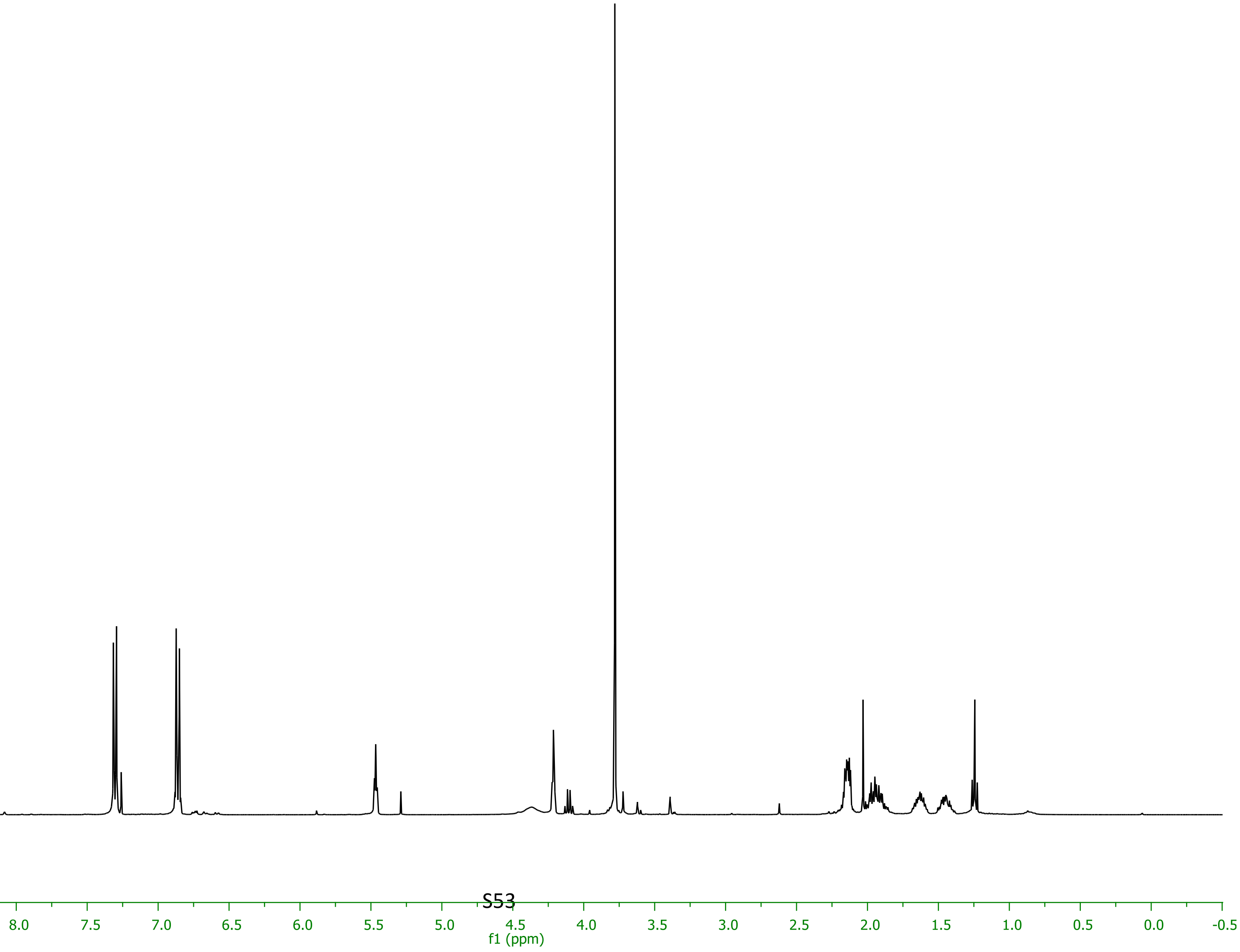

(±)-(1*R*,2*S*,6*S*)-1-Hydroxy-7-(4-methoxyphenyl)-8-oxo-7-azabicyclo[4.2.0]octan-2-yl formate **29**; $CDCl_3$, 100 MHz

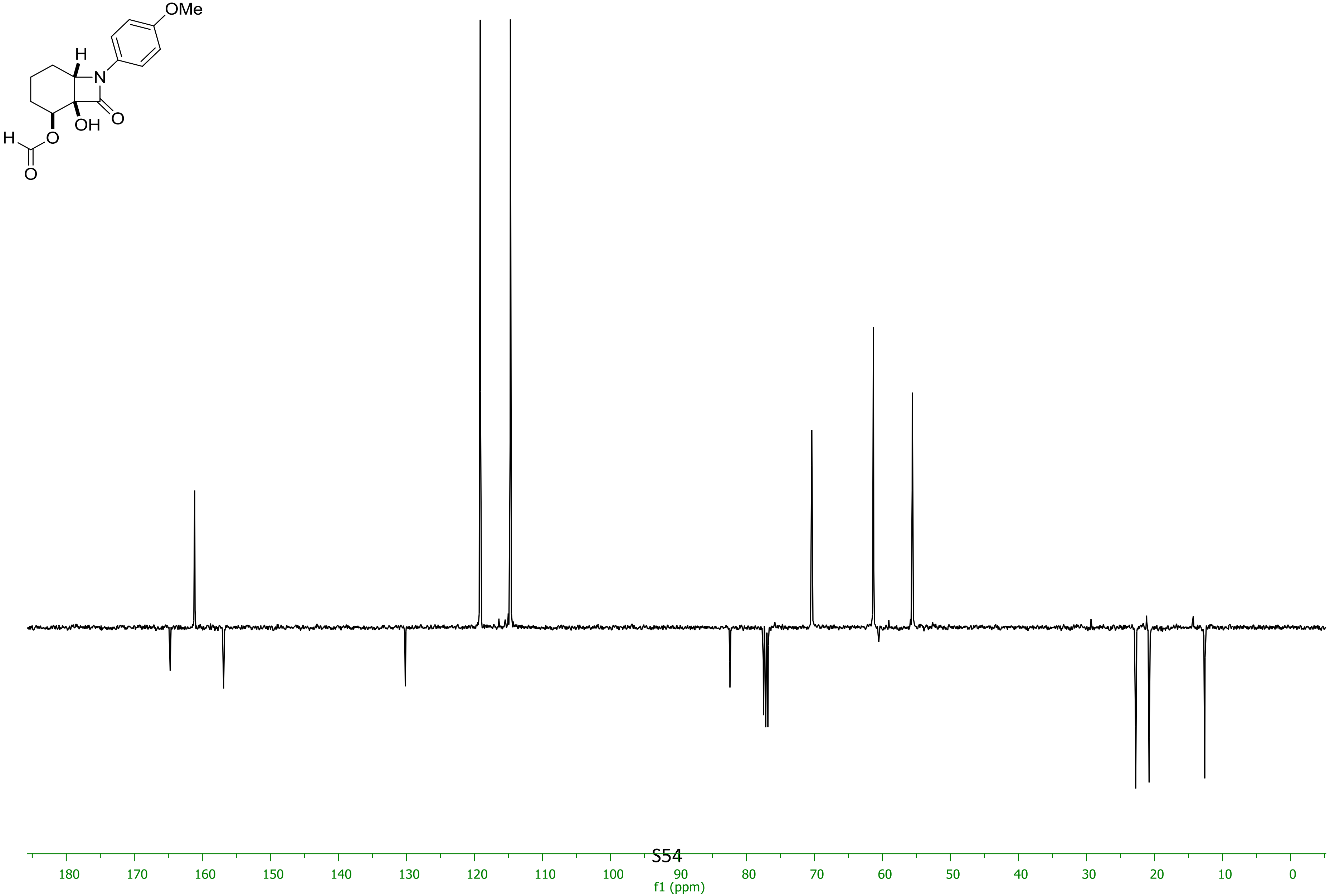

N-(Cyclopent-2-en-1-yl)-4-methoxyaniline **93**; $CDCl_3$, 400 MHz

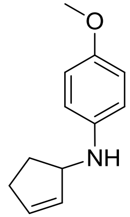

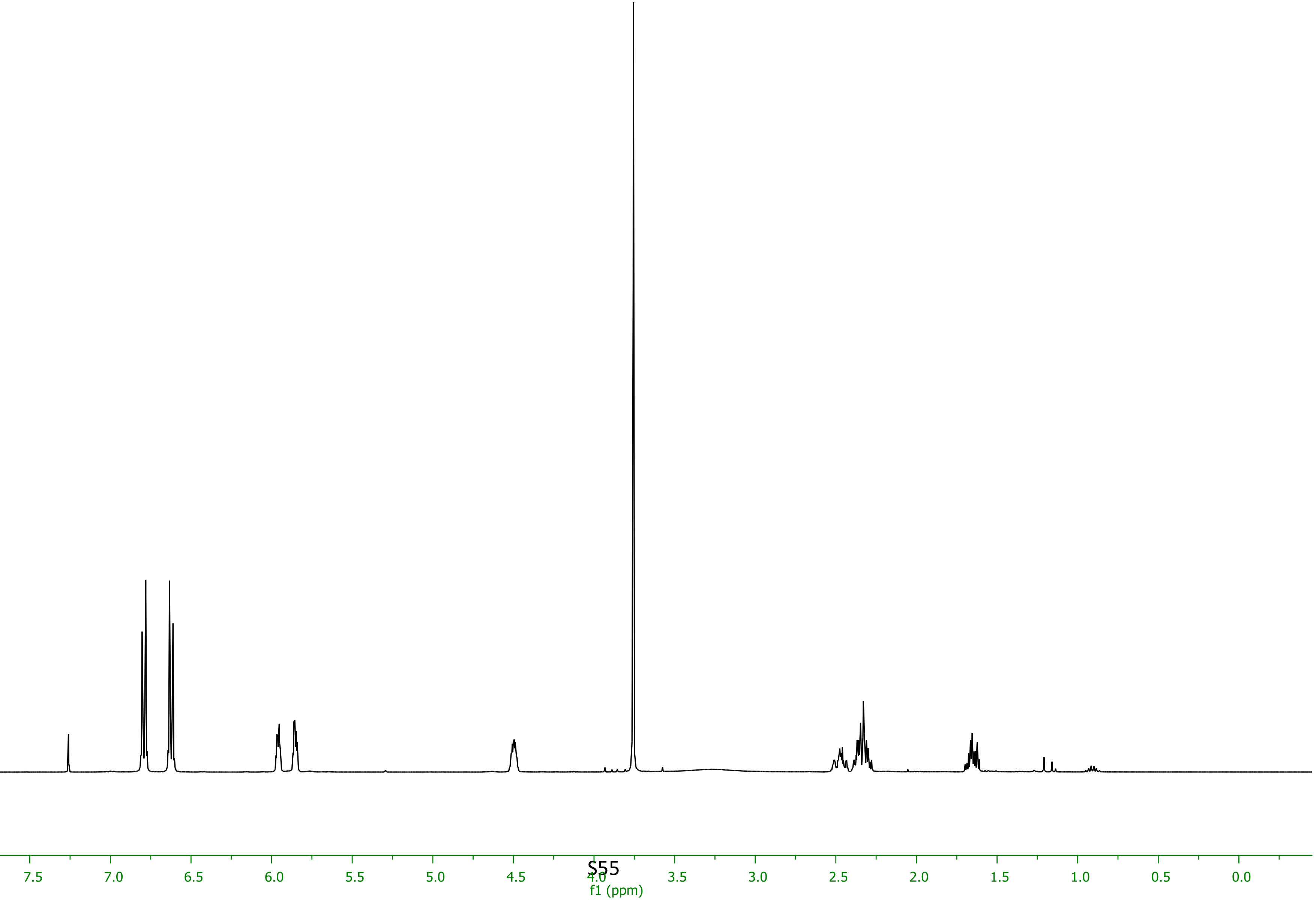

N-(Cyclopent-2-en-1-yl)-4-methoxyaniline **93**; $CDCl_3$, 100 MHz

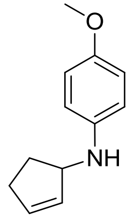

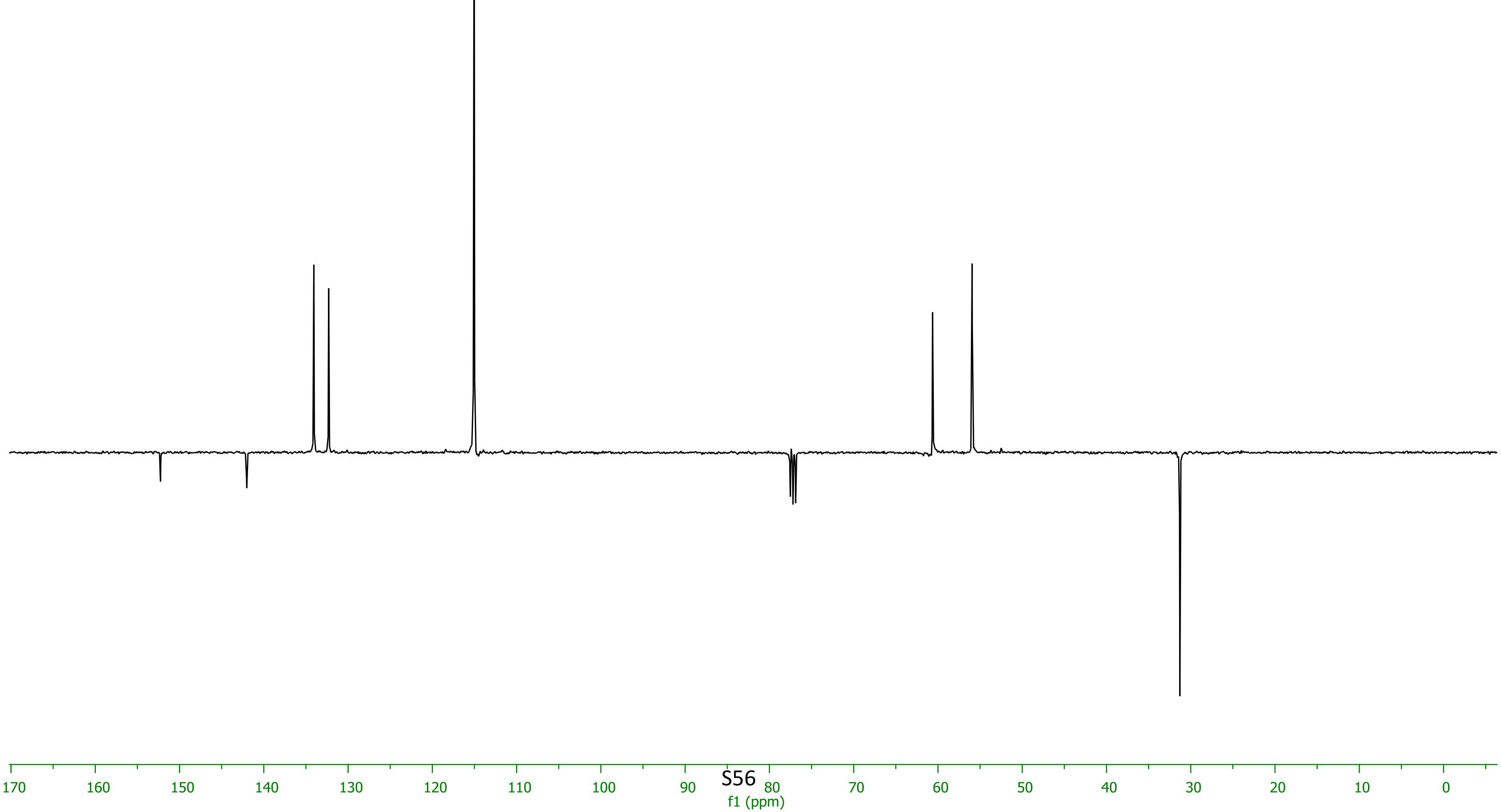

Cyclopent-2-en-1-yl(4-methoxyphenyl)carbamic chloride **94**; $CDCl_3$, 400 MHz

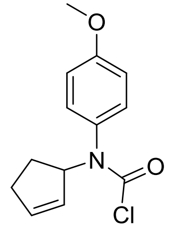

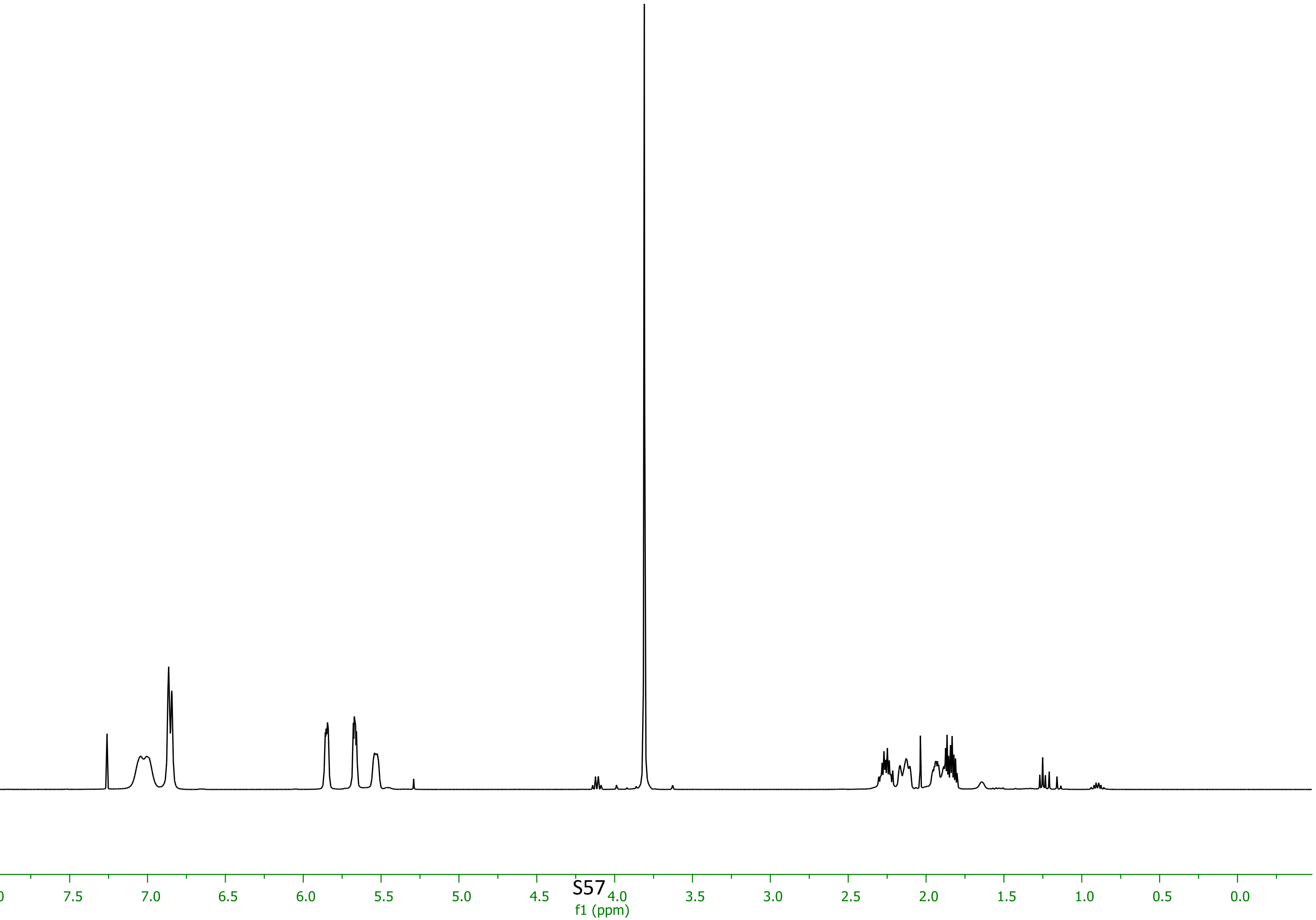

Cyclopent-2-en-1-yl(4-methoxyphenyl)carbamic chloride **94**; $CDCl_3$, 100 MHz

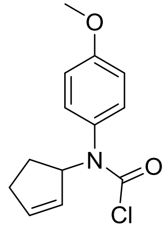

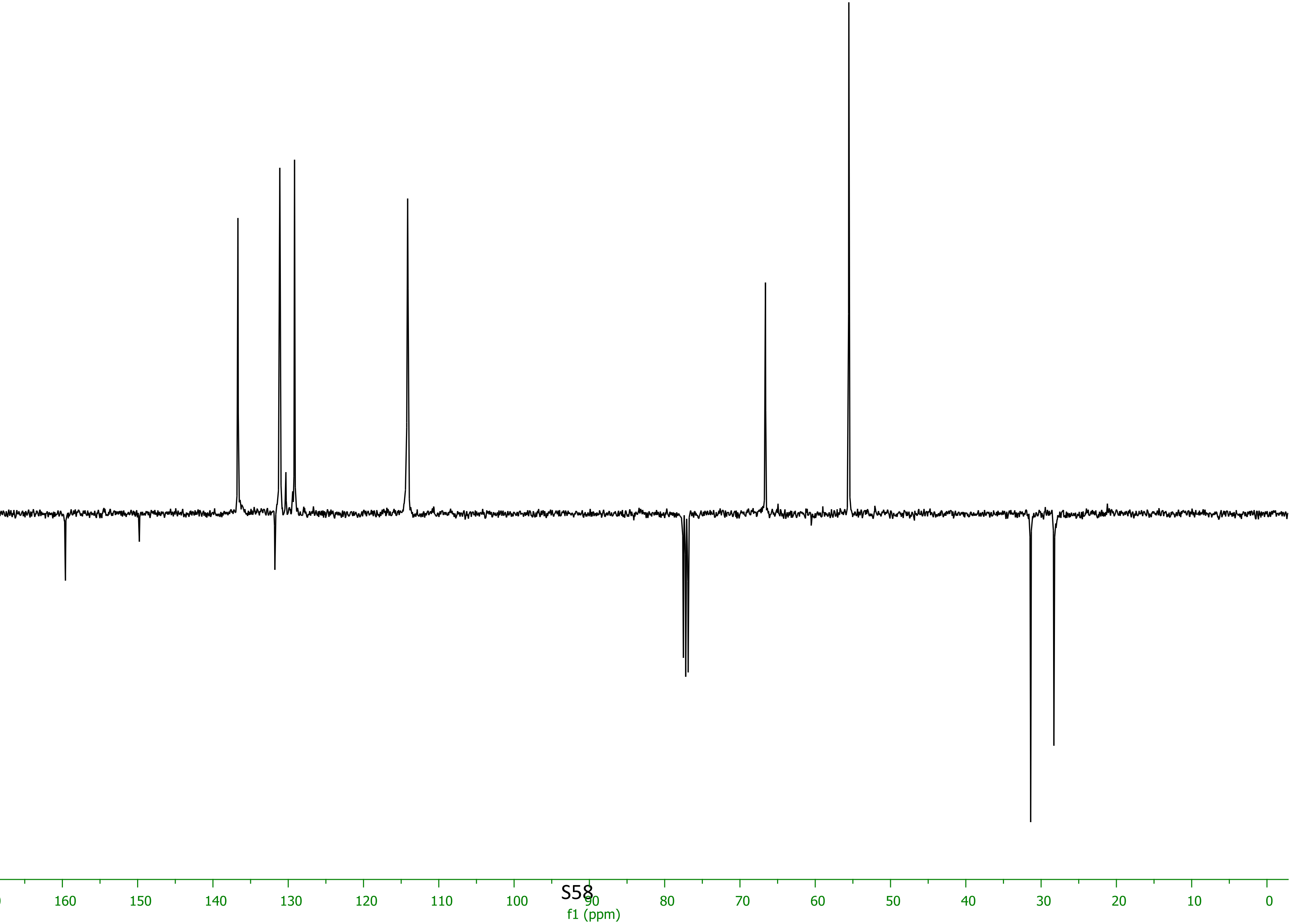

Diethylthiocarbamic acid-[(4-methoxyphenyl)(cyclopent-2-enyl)carbamic acid]-thioanhydride **34**; $CDCl_3$, 400 MHz

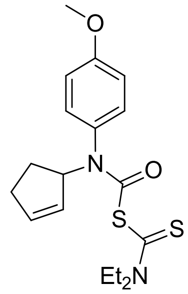

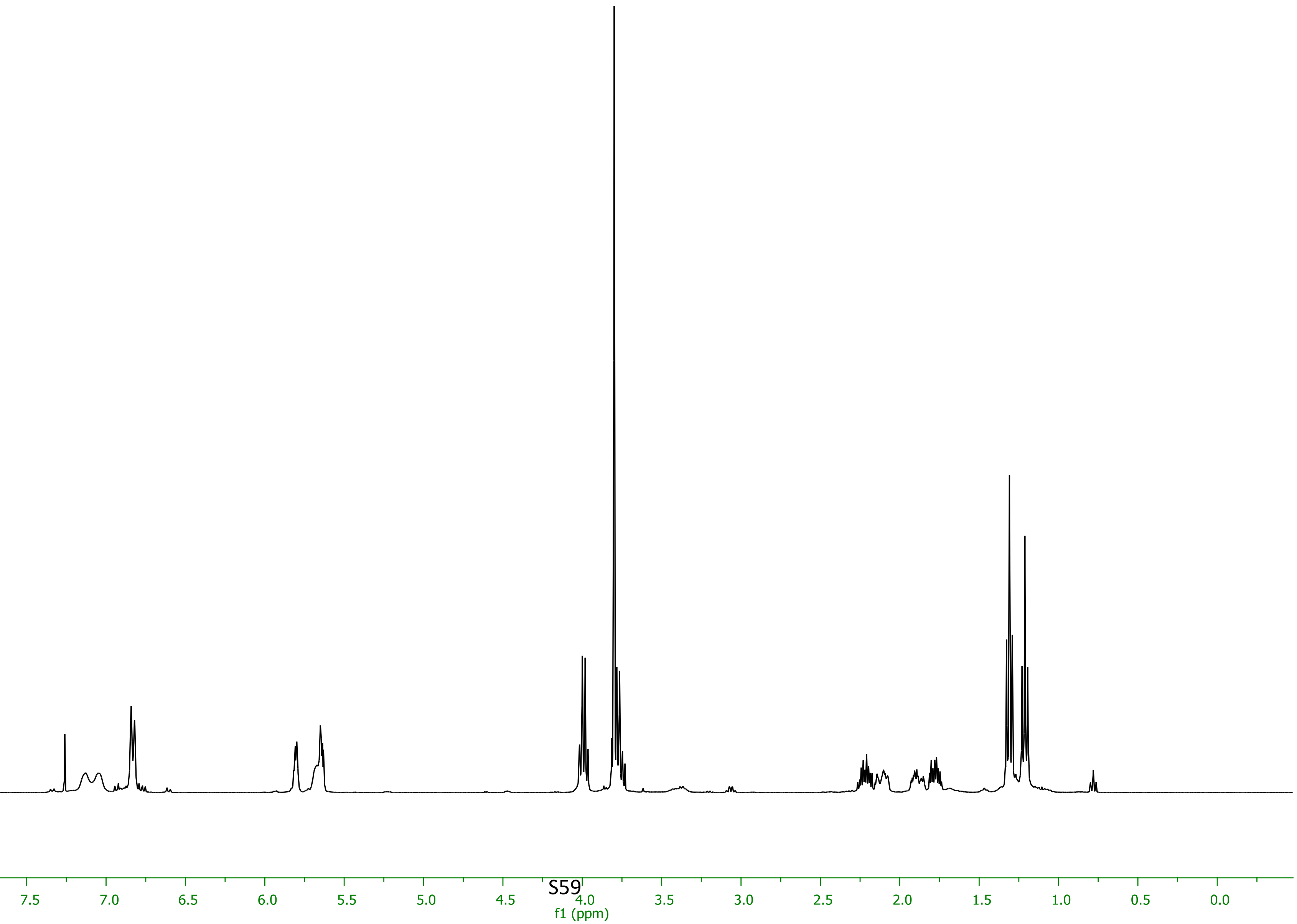

Diethylthiocarbamic acid-[(4-methoxyphenyl)(cyclopent-2-enyl)carbamic acid]-thioanhydride **34**; $CDCl_3$, 100 MHz

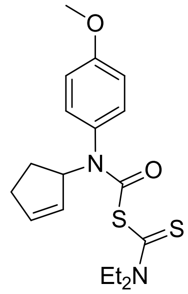

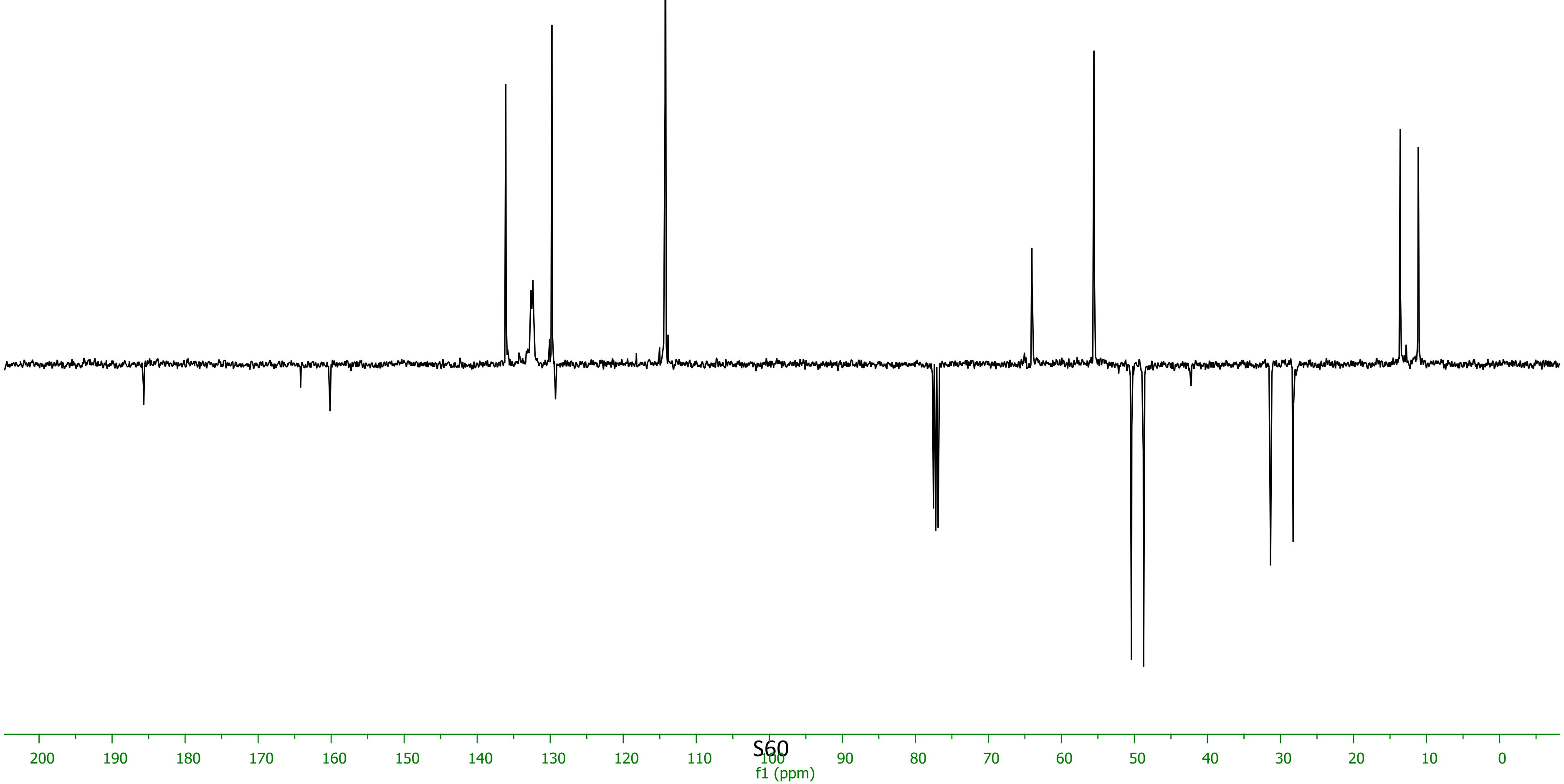

(±)-(1*R*,2*S*,5*S*)-6-(4-Methoxyphenyl)-7-oxo-6-azabicyclo[3.2.0]heptan-2-yl diethylcarbamodithioate **47**; $CDCl_3$, 300 MHz

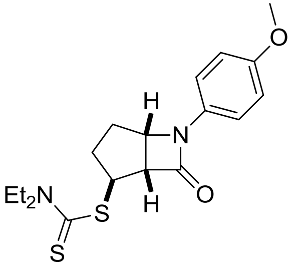

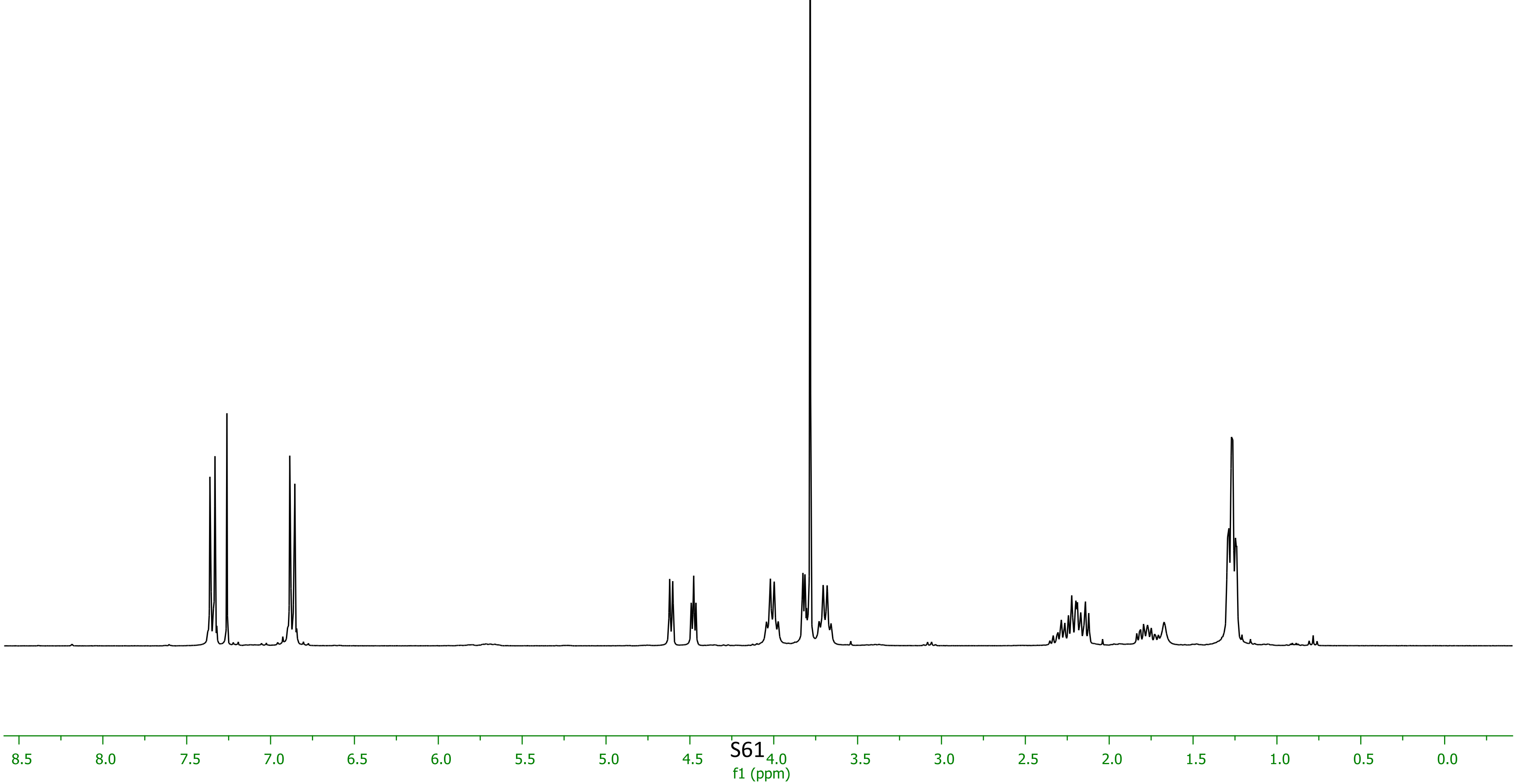

(±)-(1*R*,2*S*,5*S*)-6-(4-Methoxyphenyl)-7-oxo-6-azabicyclo[3.2.0]heptan-2-yl diethylcarbamodithioate **47**; $CDCl_3$, 100 MHz

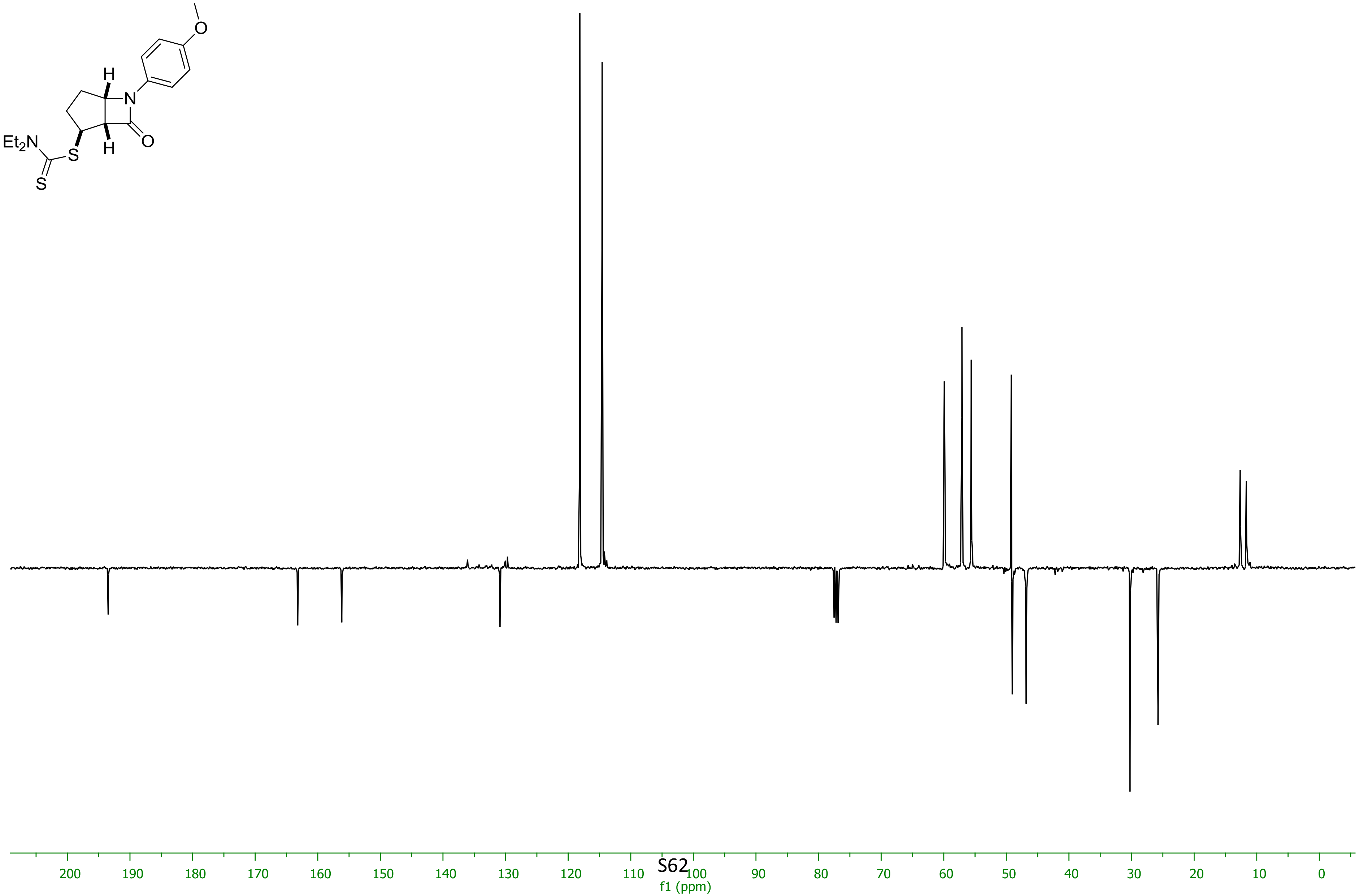

(±)-(2*R*,5*S*)-*N*-Benzyl-3-bromobicyclo[3.2.1]oct-3-en-2-amine **41a**; $CDCl_3$, 400 MHz

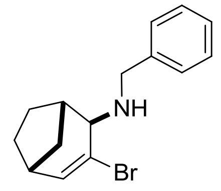

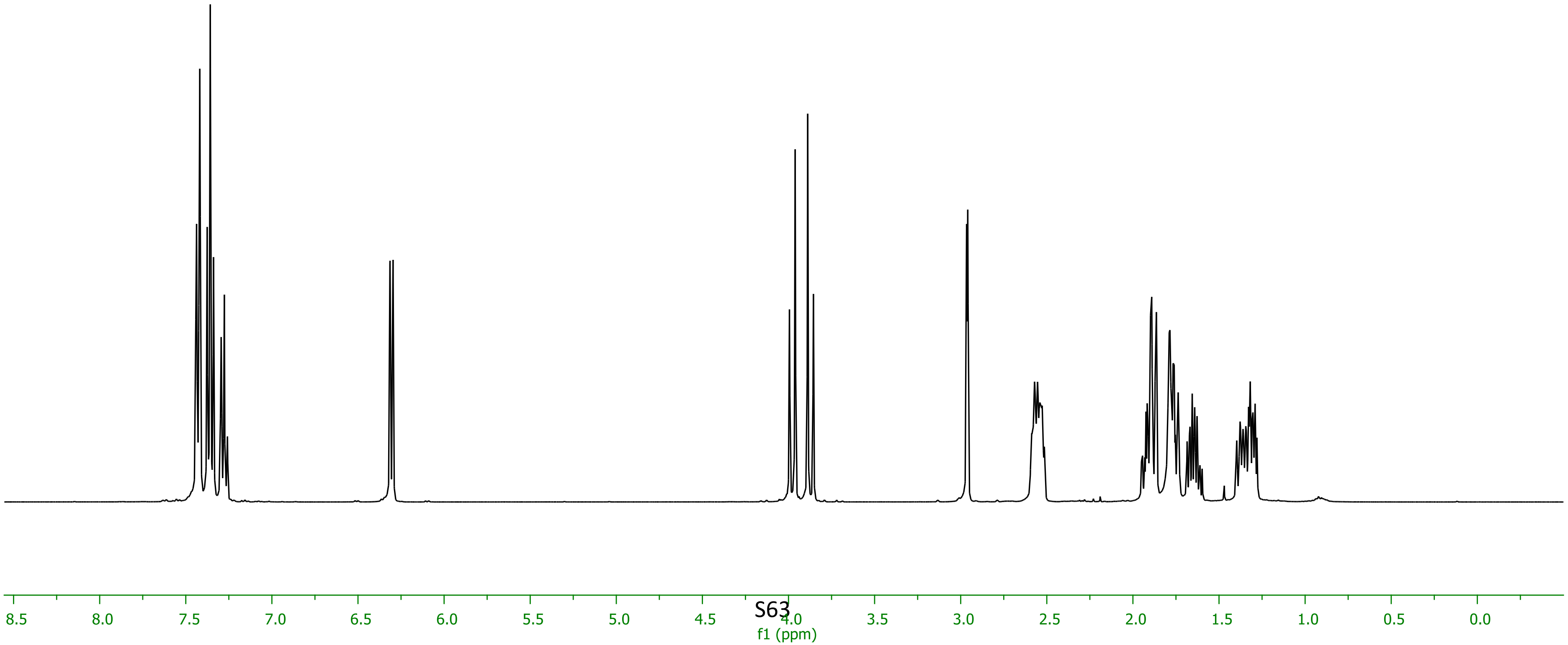

(±)-(2*R*,5*S*)-*N*-Benzyl-3-bromobicyclo[3.2.1]oct-3-en-2-amine **41a**; $CDCl_3$, 100 MHz

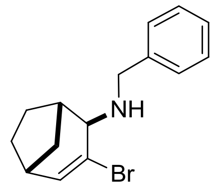

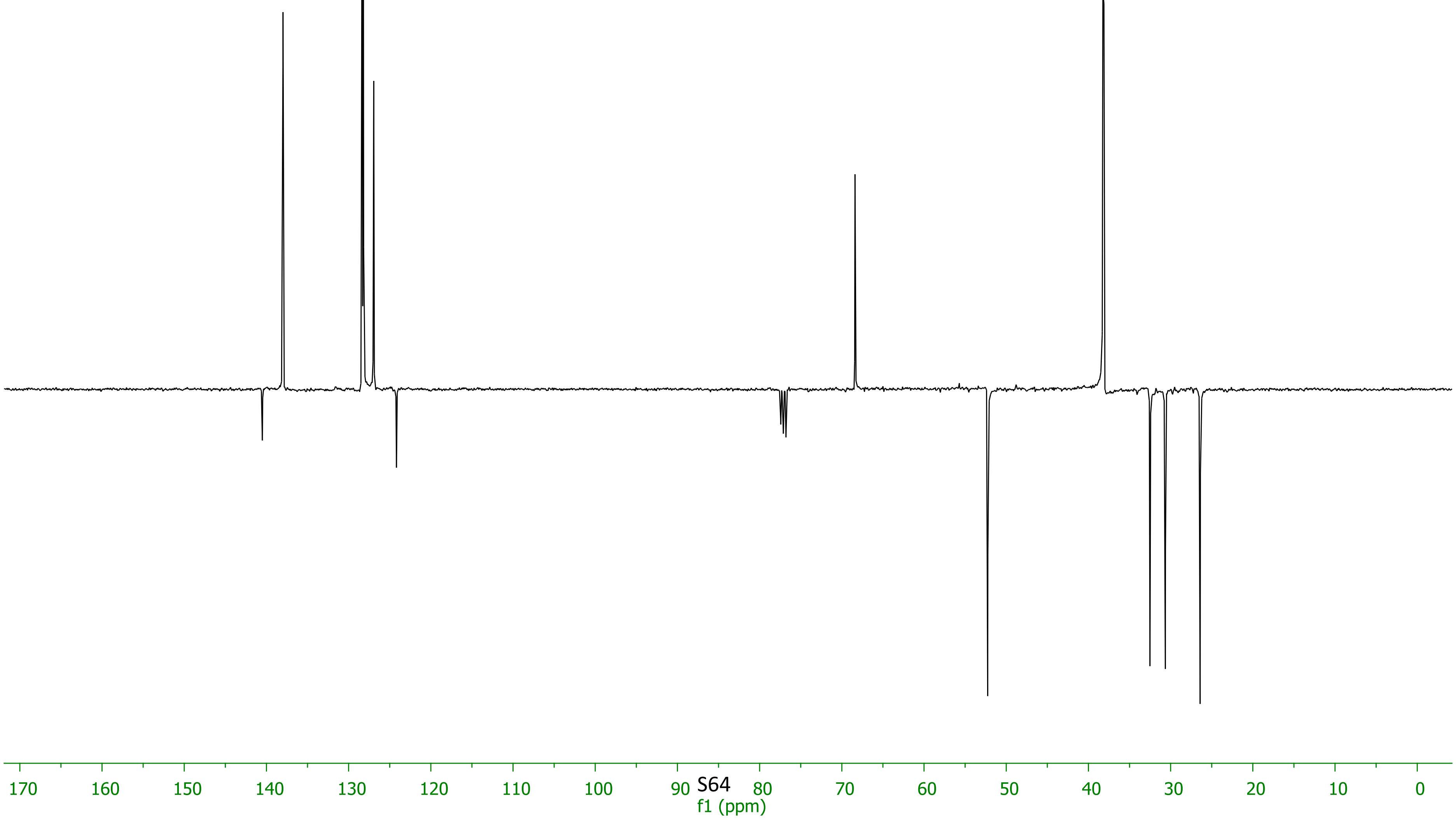

(±)- (2*R*,5*S*)-3-Bromo-*N*-(4-methoxyphenyl)bicyclo[3.2.1]oct-3-en-2-amine **41b**; $CDCl_3$, 400 MHz

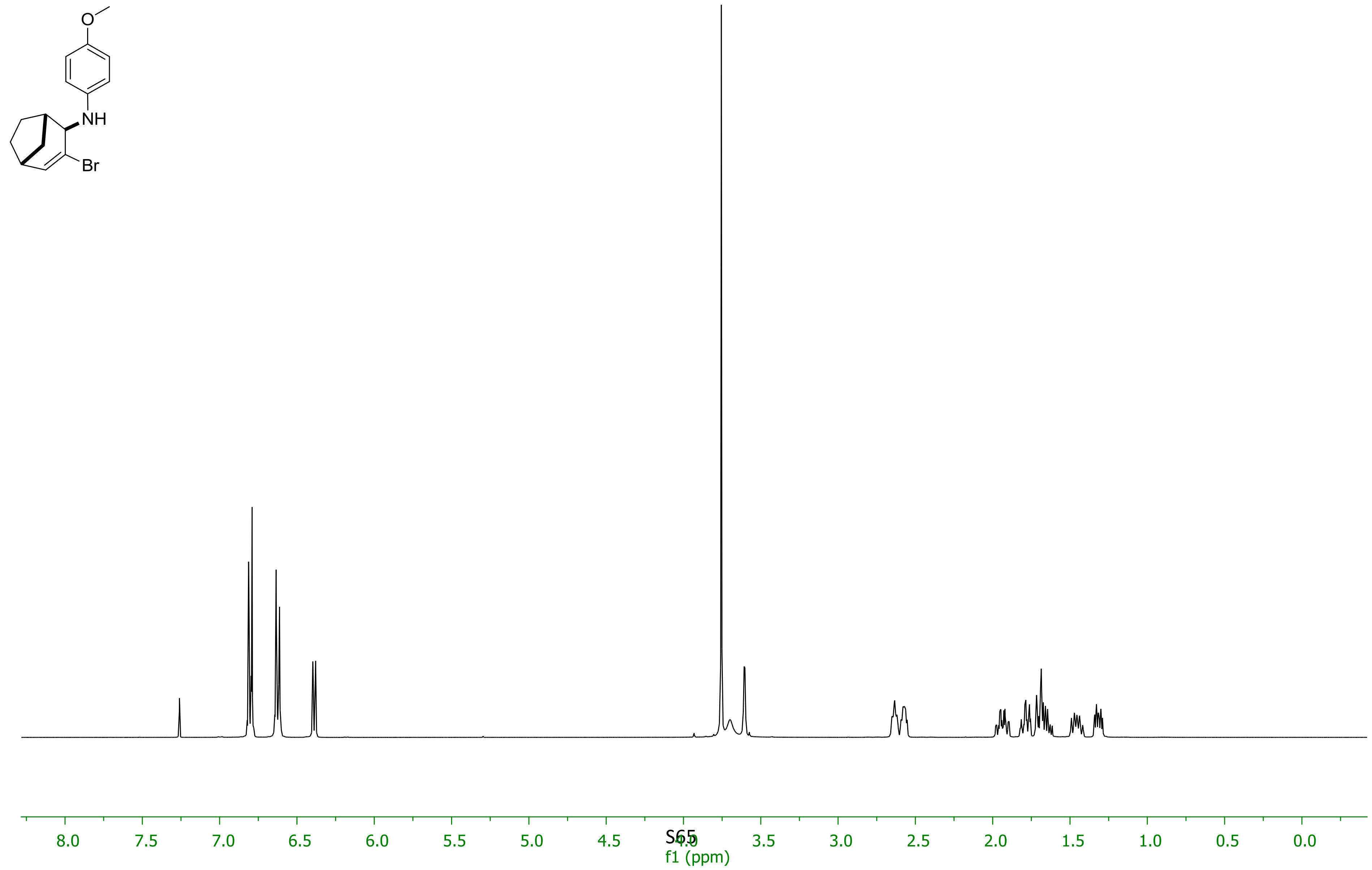

(±)- (2*R*,5*S*)-3-Bromo-*N*-(4-methoxyphenyl)bicyclo[3.2.1]oct-3-en-2-amine **41b**; $CDCl_3$, 100 MHz

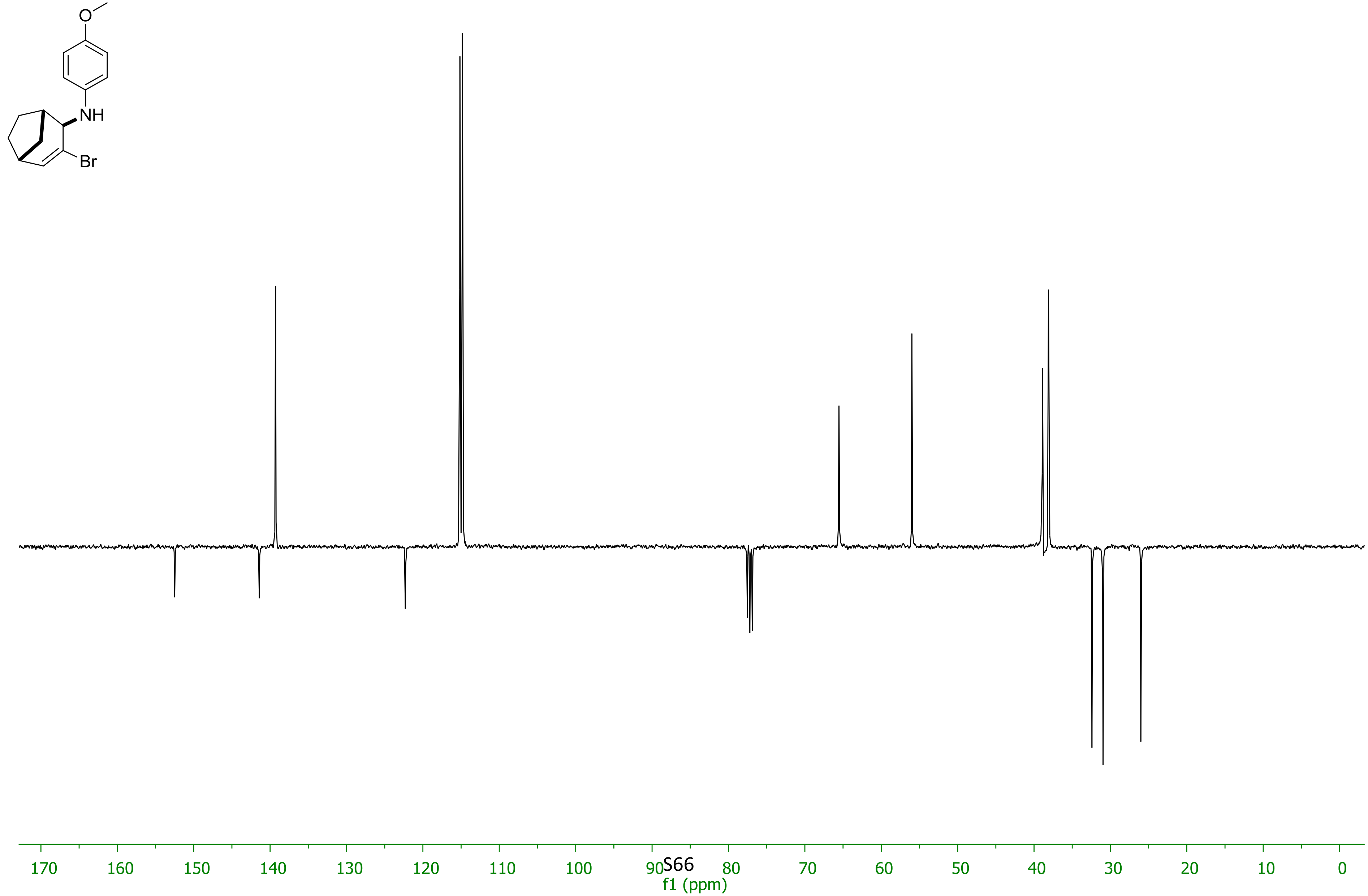

(±)-(2*R*,5*S*)-*N*-(4-Methoxyphenyl)bicyclo[3.2.1]oct-3-en-2-amine **42**; $CDCl_3$, 400 MHz

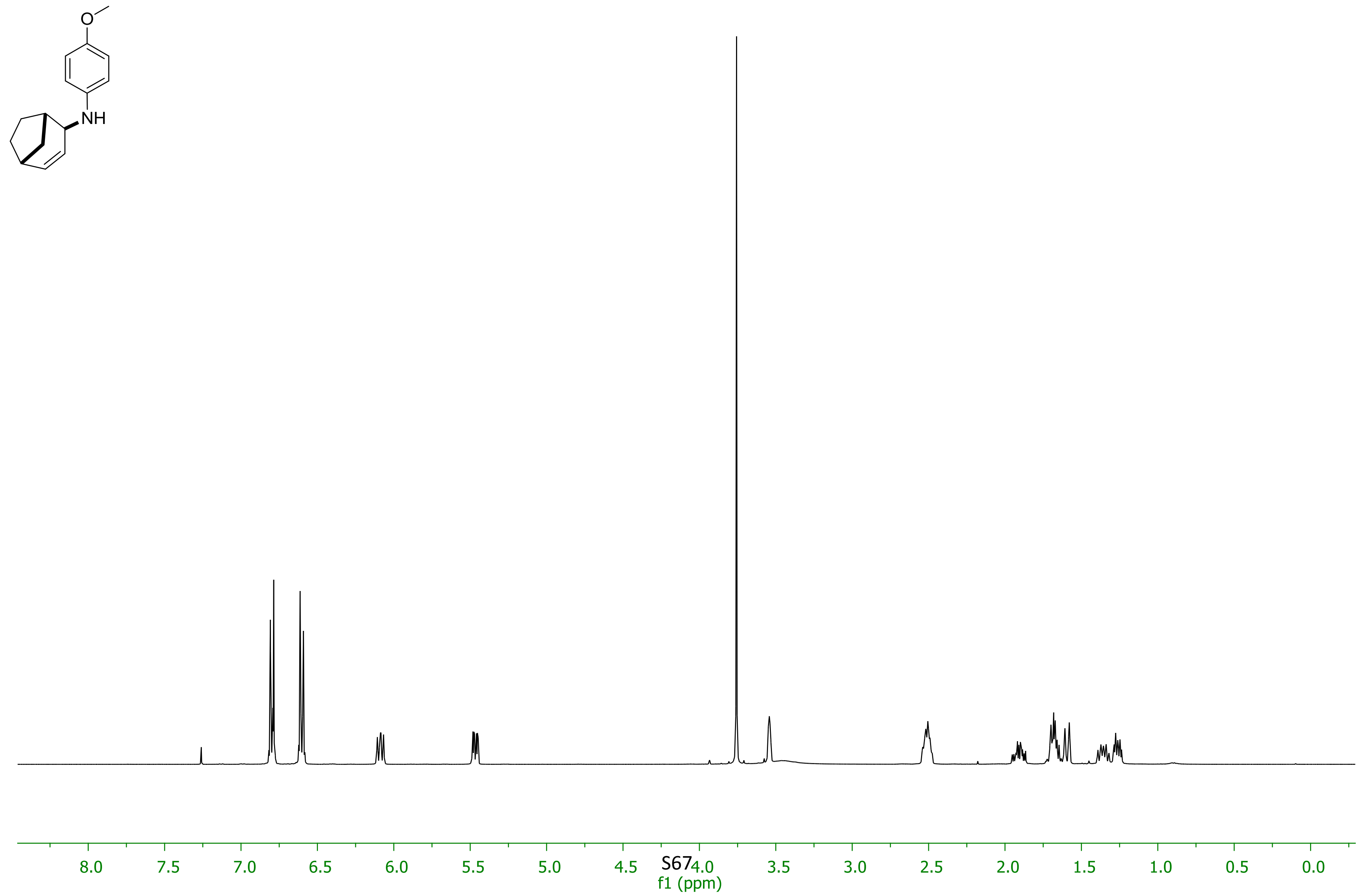

(±)-(2*R*,5*S*)-*N*-(4-Methoxyphenyl)bicyclo[3.2.1]oct-3-en-2-amine **42**; $CDCl_3$, 100 MHz

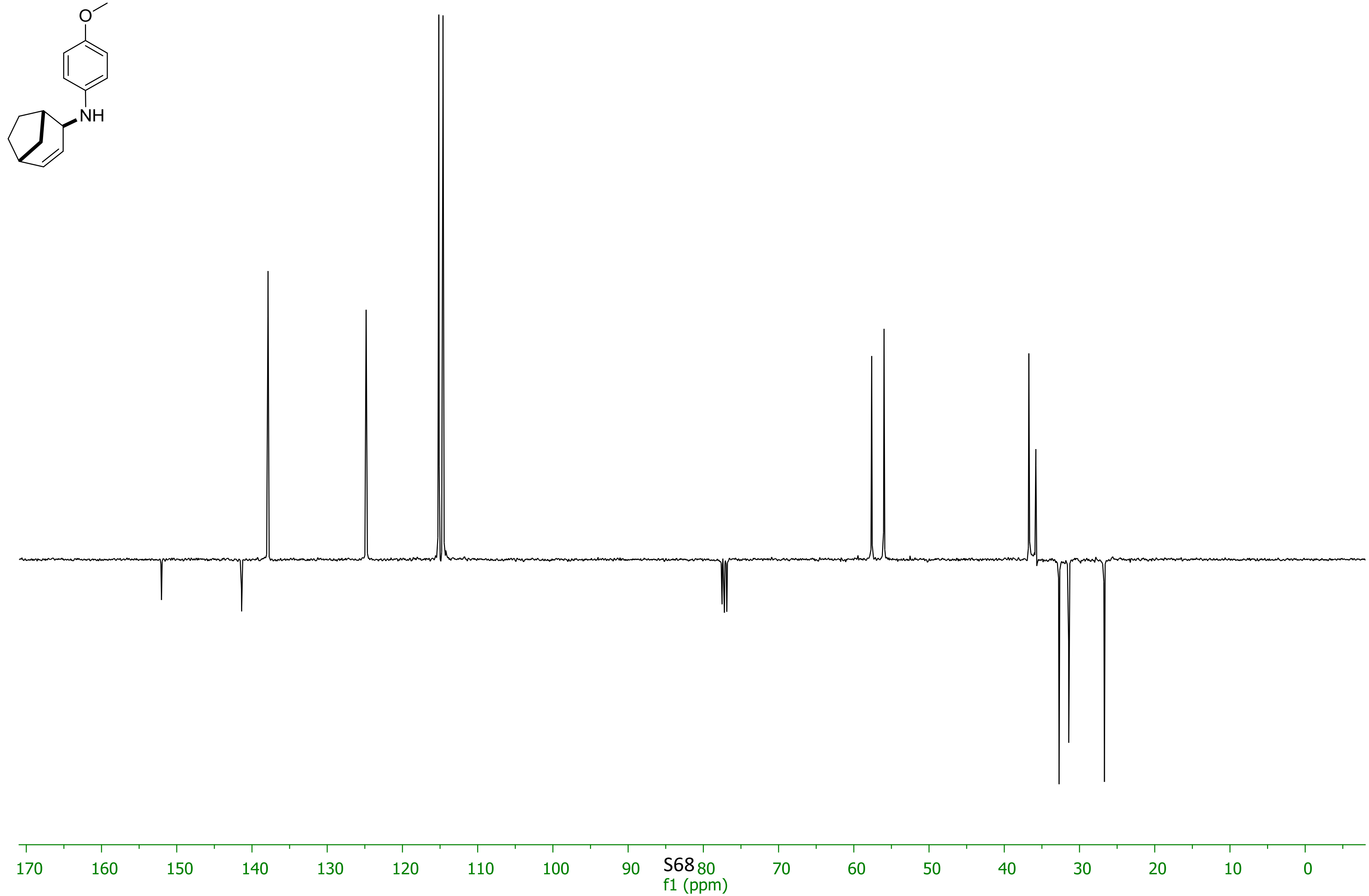

(±)-(2*R*,5*S*)-Bicyclo[3.2.1]oct-3-en-2-yl(4-methoxyphenyl)carbamic chloride **95**; $CDCl_3$, 400 MHz

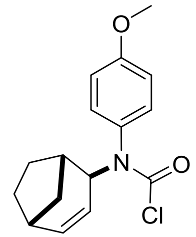

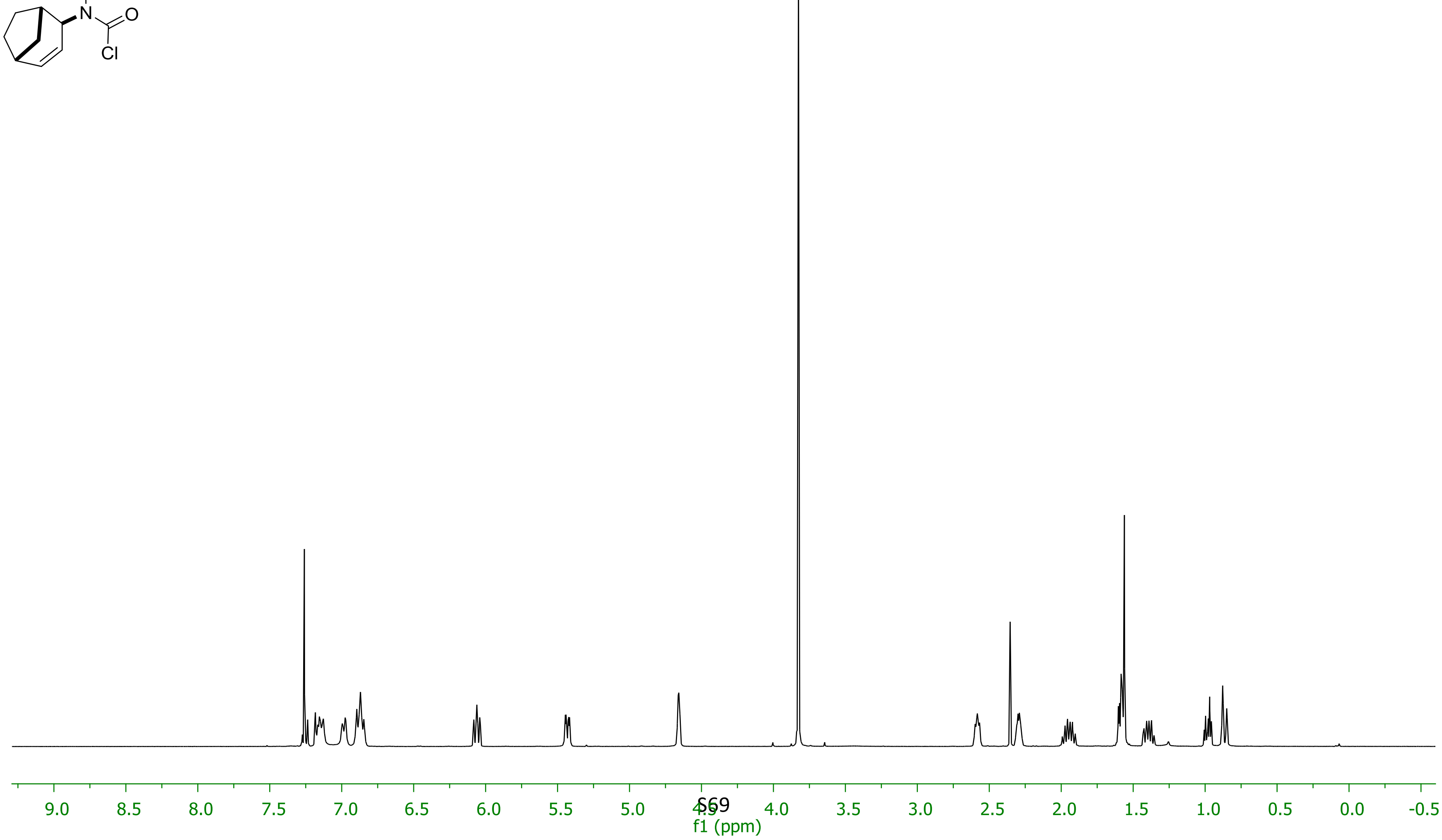

(±)-(2*R*,5*S*)-Bicyclo[3.2.1]oct-3-en-2-yl(4-methoxyphenyl)carbamic chloride **95**; $CDCl_3$, 100 MHz

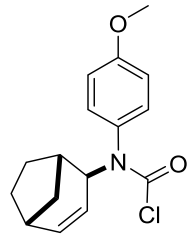

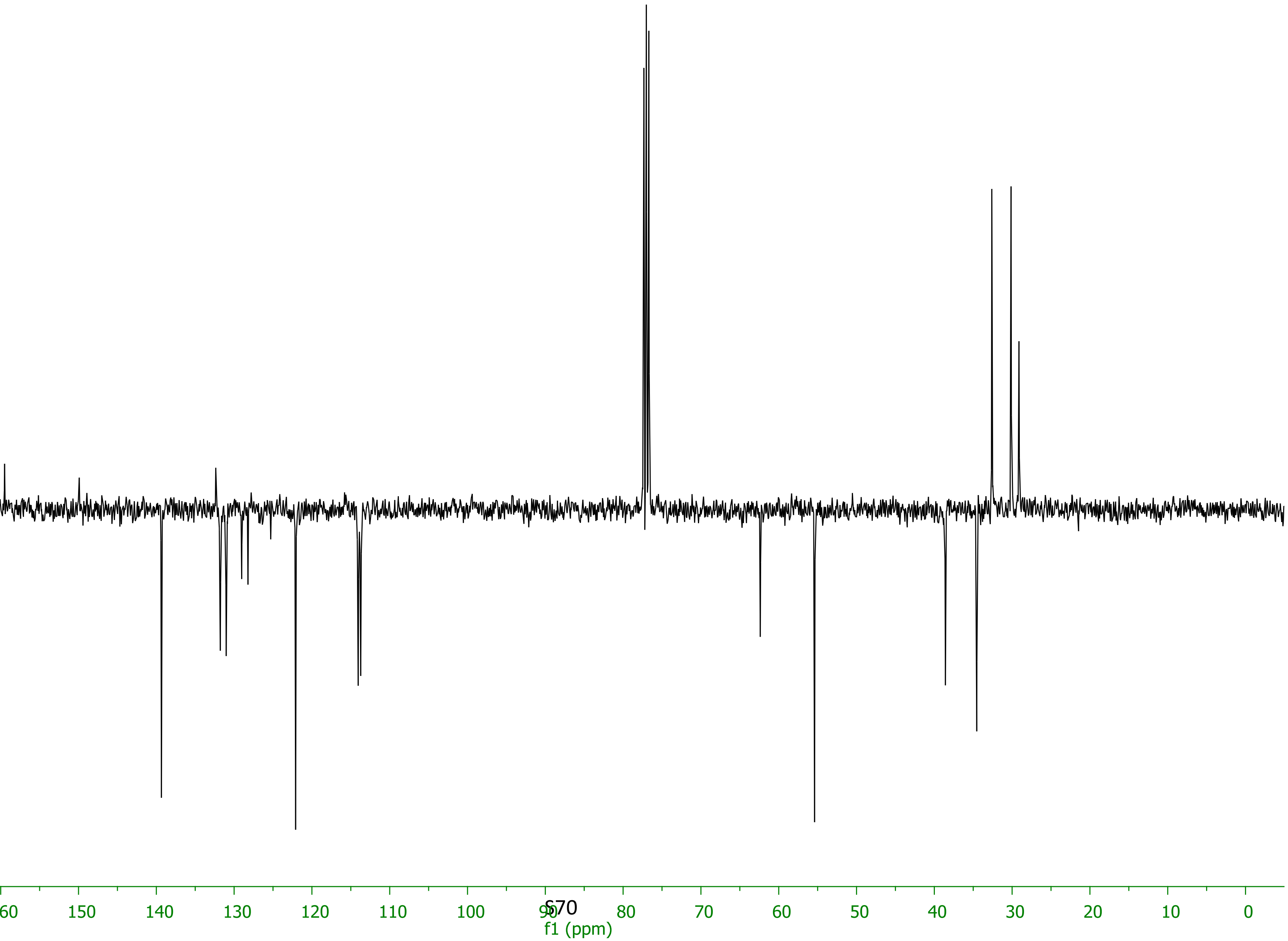

(±)-(2*R*,5*S*)-Diethyldithiocarbamic acid-[(4-methoxyphenyl)(bicyclo[3.2.1]oct-3-en-2-yl)carbamic acid]-thioanhydride **36**; $CDCl_3$, 400 MHz

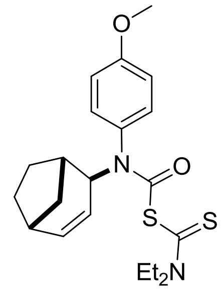

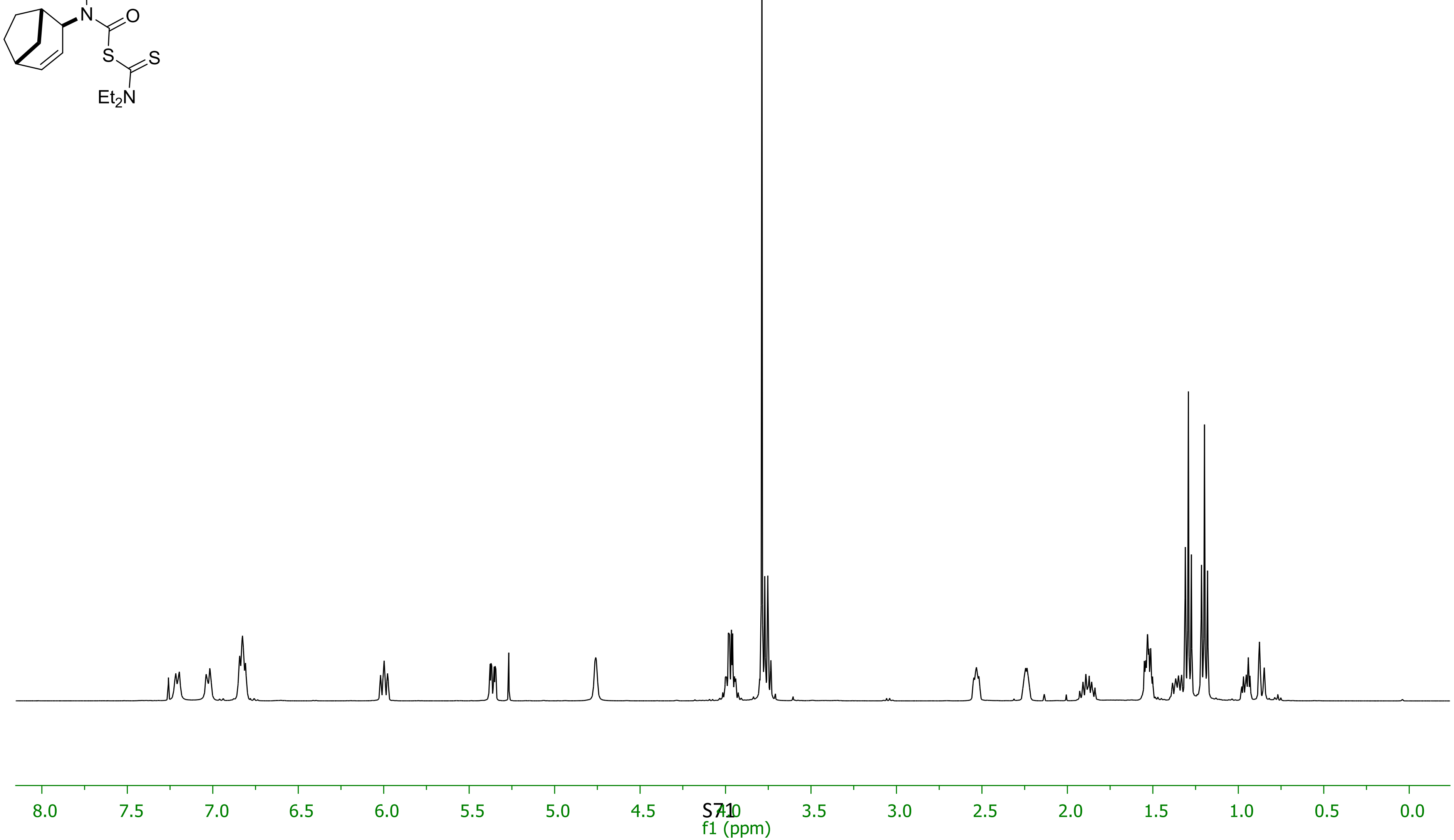

(±)-(2*R*,5*S*)-Diethyldithiocarbamic acid-[(4-methoxyphenyl)(bicyclo[3.2.1]oct-3-en-2-yl)carbamic acid]-thioanhydride **36**; $CDCl_3$, 100 MHz

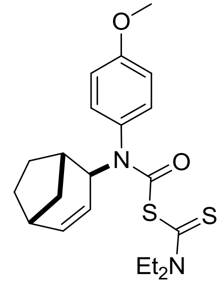

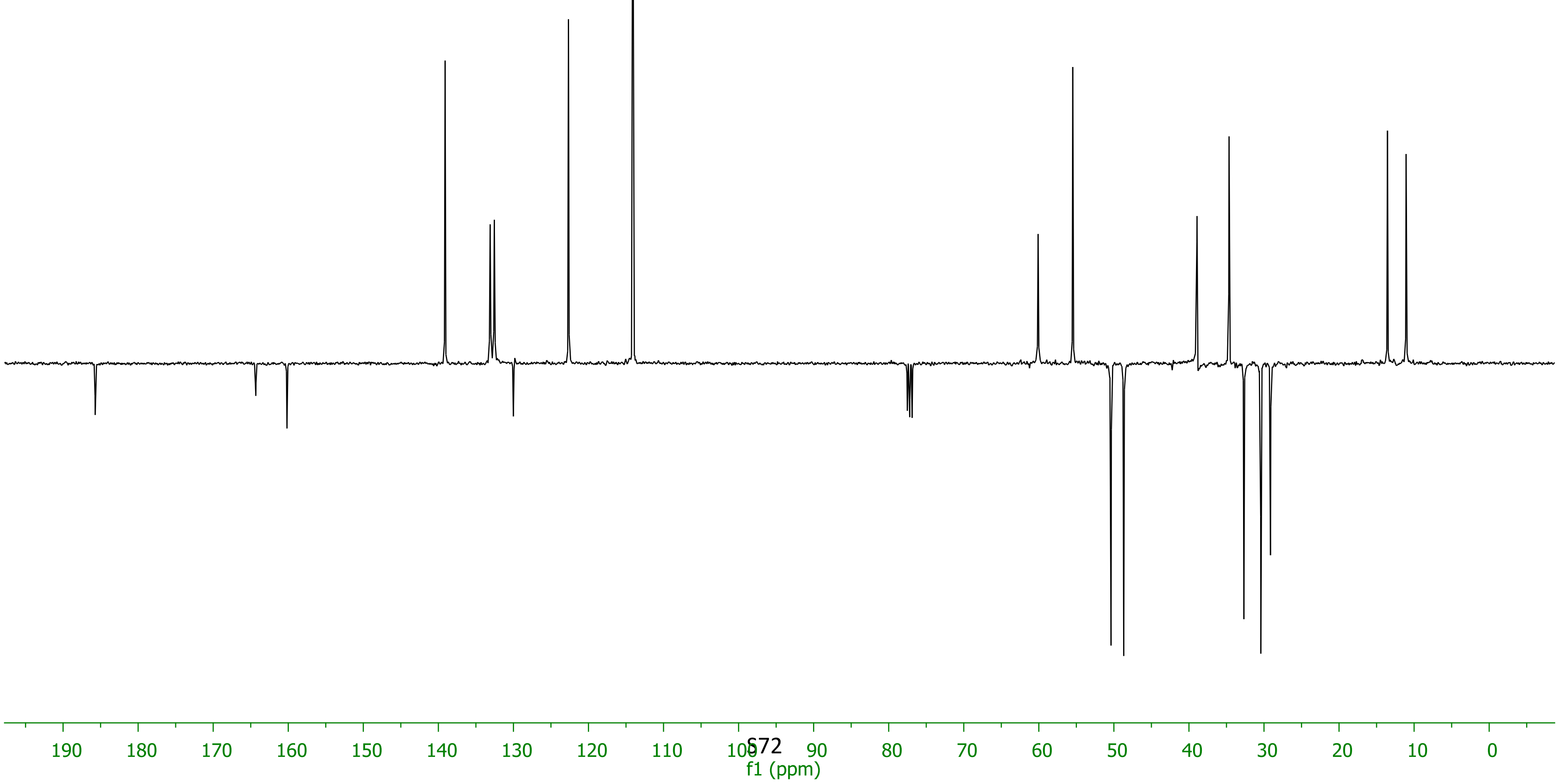

(±)-(1*S*,2*R*,5*S*,6*R*,7*R*)-3-(4-Methoxyphenyl)-4-oxo-3-azatricyclo[5.2.1.0^{2,5}]decan-6-yl diethyldithiocarbamate **50**; $CDCl_3$, 400 MHz

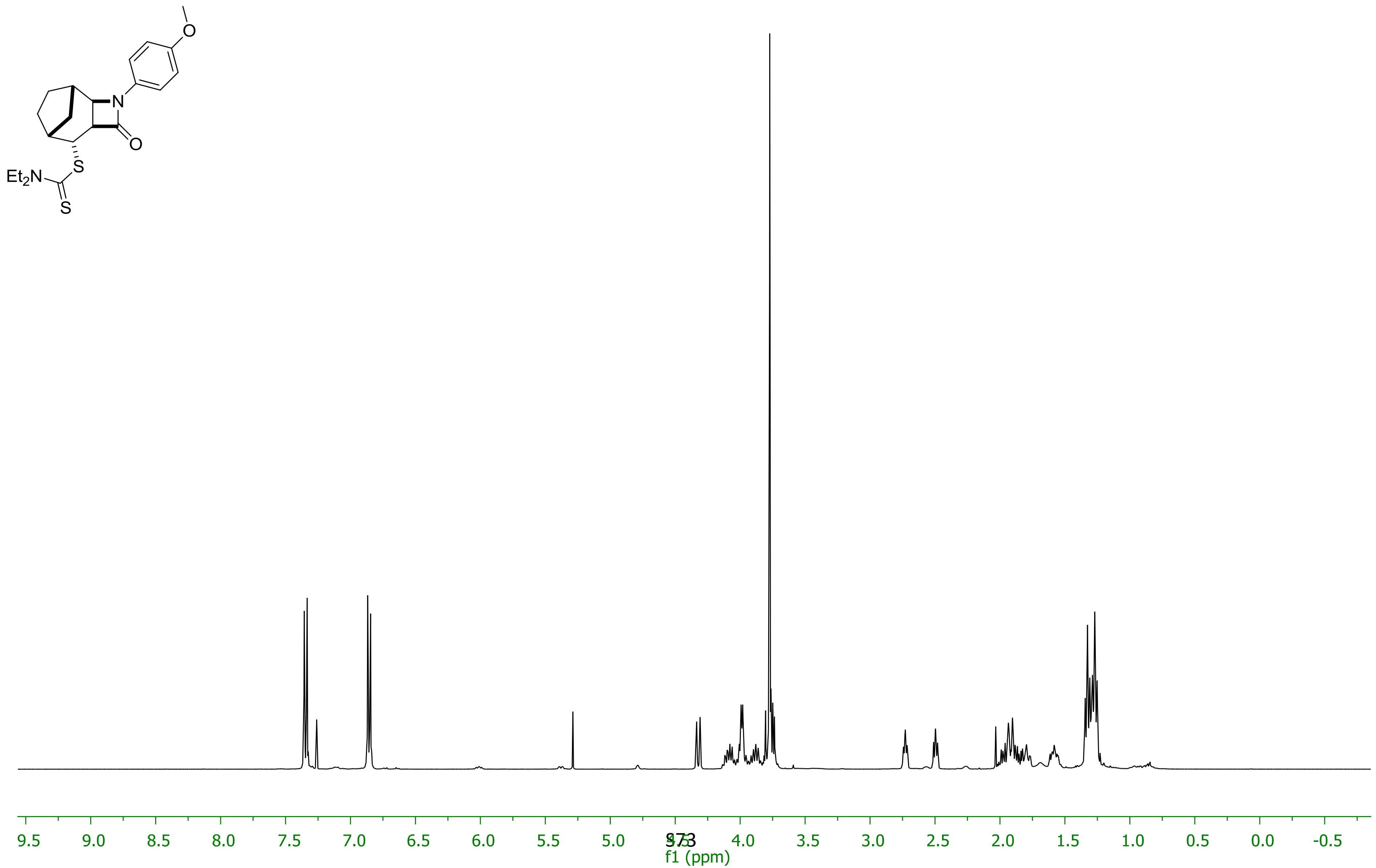

(±)-(1*S*,2*R*,5*S*,6*R*,7*R*)-3-(4-Methoxyphenyl)-4-oxo-3-azatricyclo[5.2.1.0^{2,5}]decan-6-yl diethyldithiocarbamate **50**; $CDCl_3$, 100 MHz

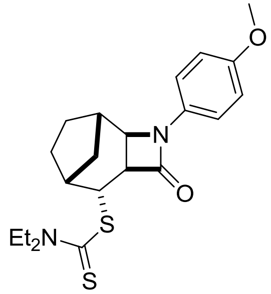

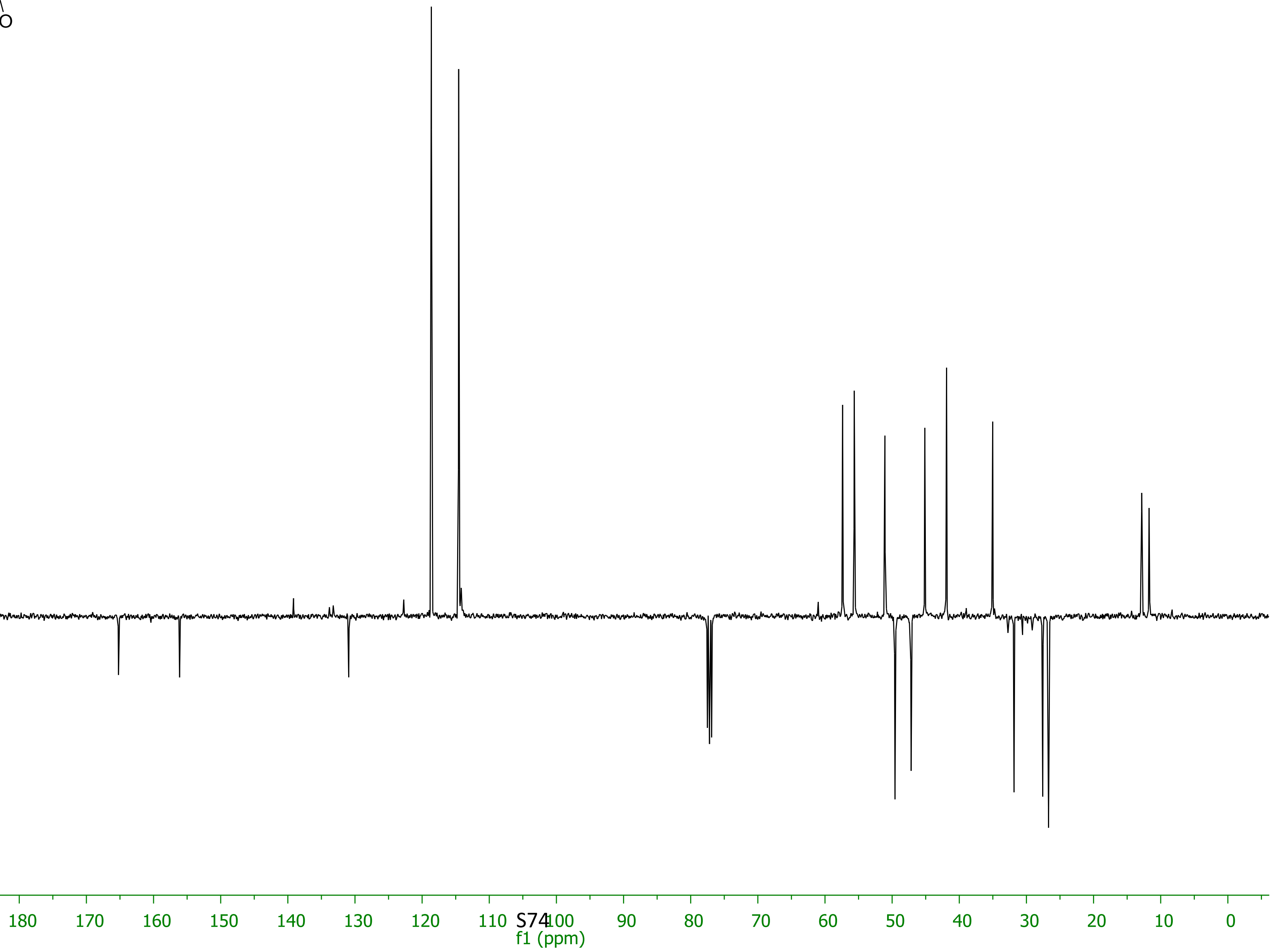

(±)-(1*S*,2*R*,5*S*,6*S*,7*R*)-3-(4-methoxyphenyl)-4-oxo-3-azatricyclo[5.2.1.0^{2,5}]decan-6-yl diethyldithiocarbamate **51**; $CDCl_3$, 400 MHz

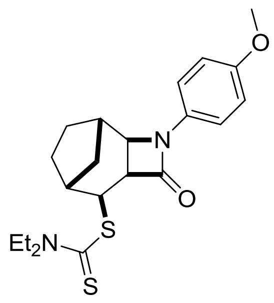

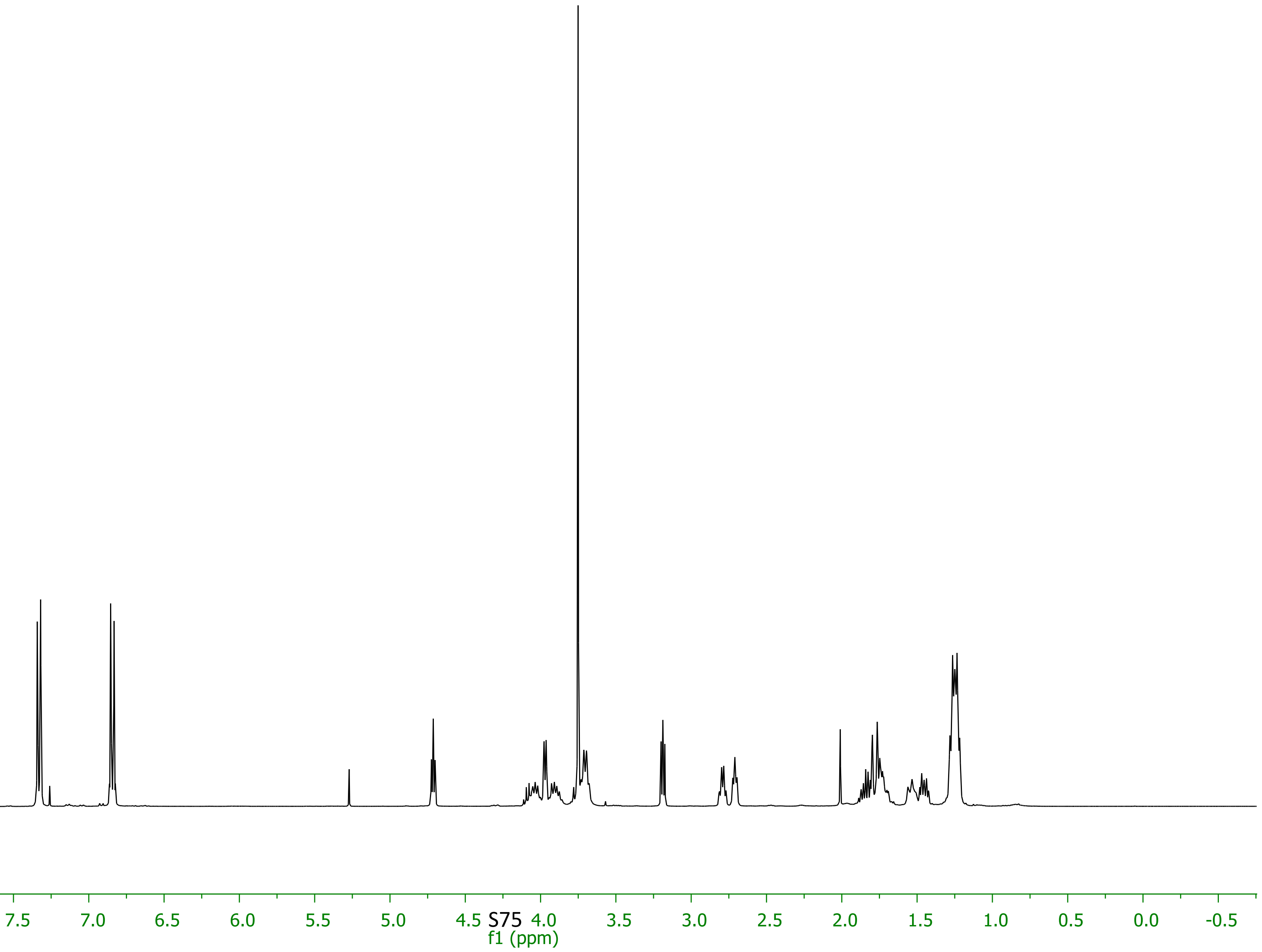

(±)-(1*S*,2*R*,5*S*,6*S*,7*R*)-3-(4-methoxyphenyl)-4-oxo-3-azatricyclo[5.2.1.$0^{2,5}$]decan-6-yl diethyldithiocarbamate **51**; $CDCl_3$, 100 MHz

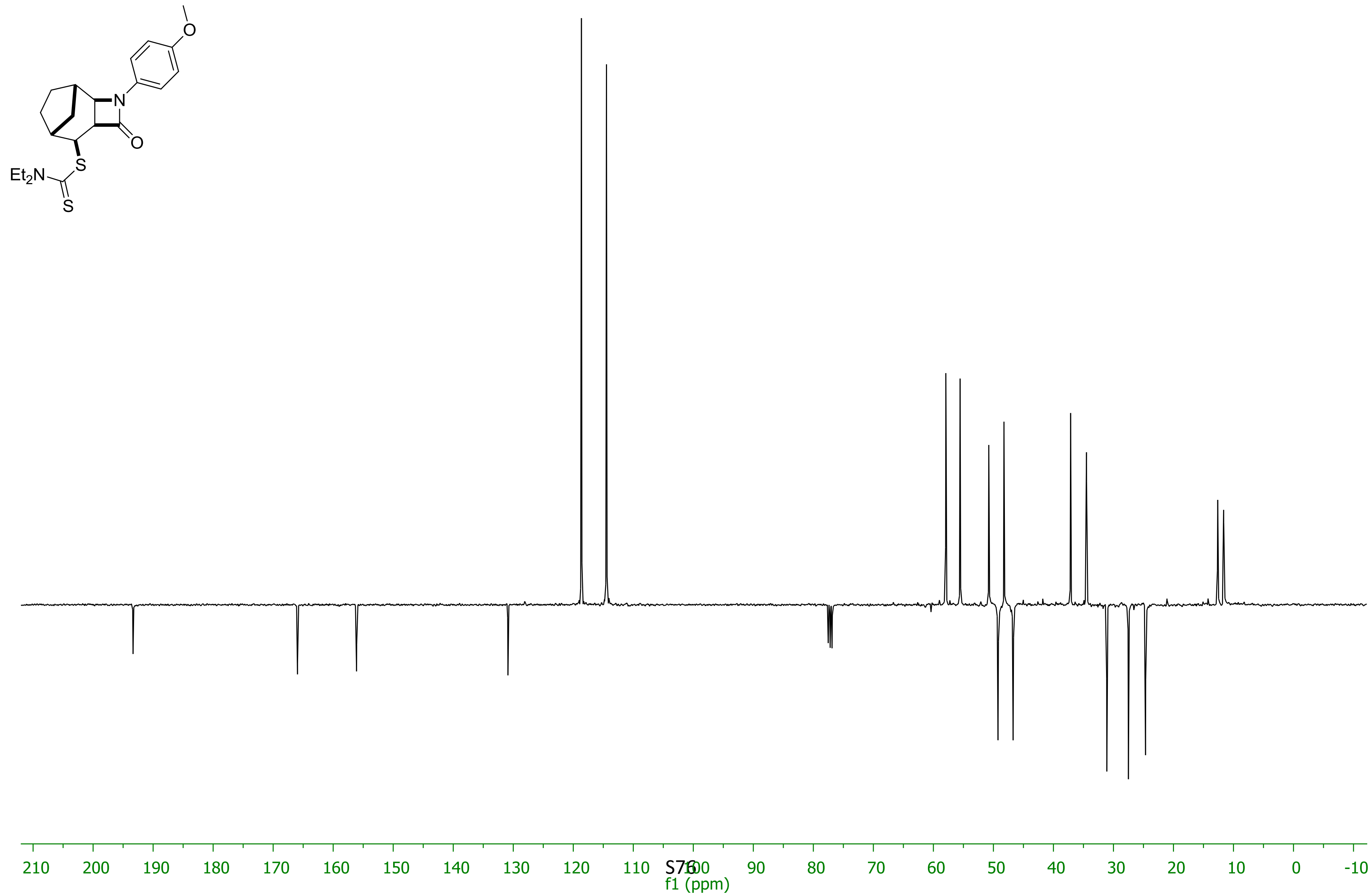

N-Isopropylcyclohexa-2,5-dienamine **44:45**; $CDCl_3$, 400 MHz

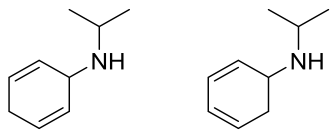

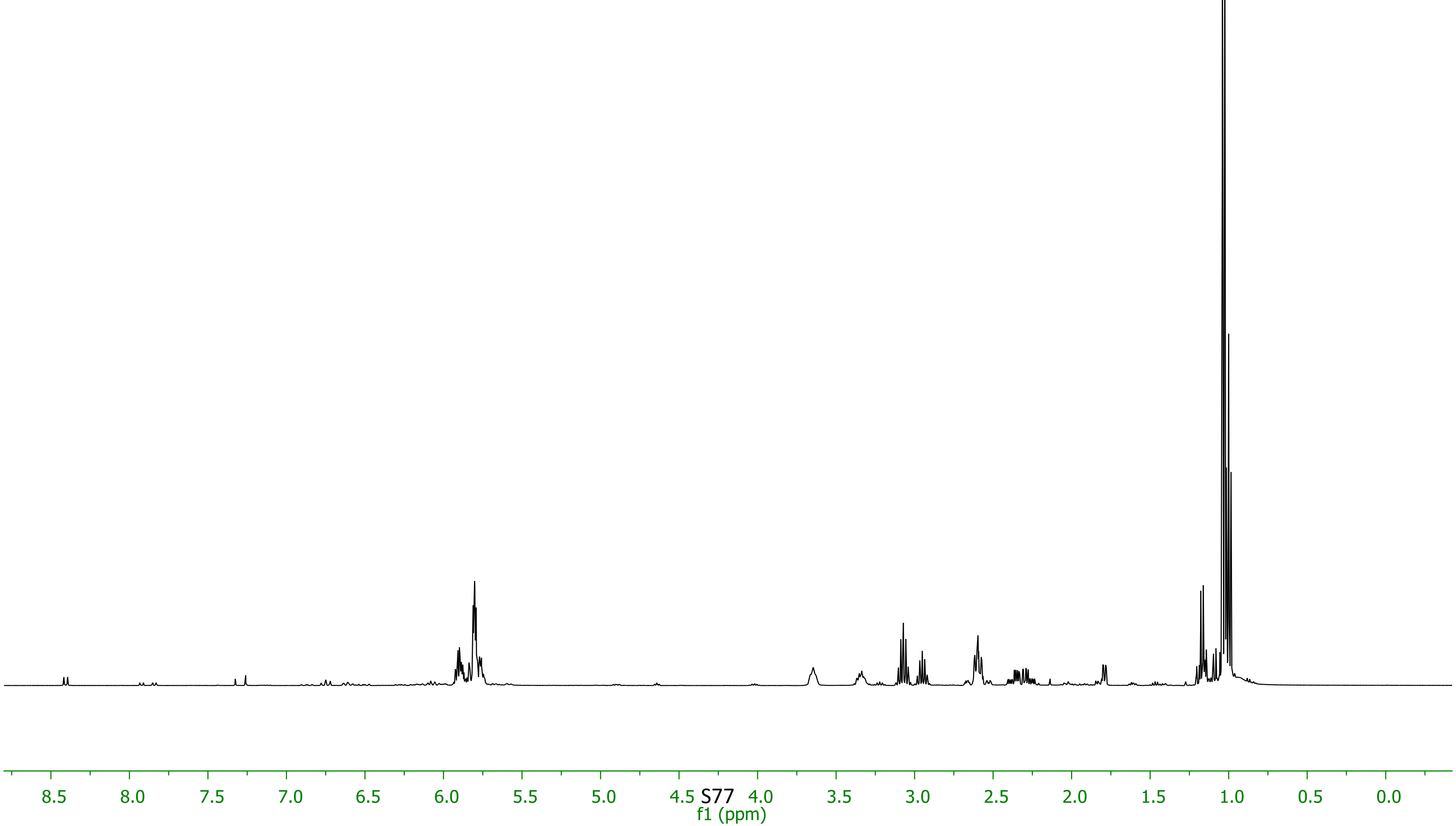

N-Isopropylcyclohexa-2,5-dienamine **44:45**; $CDCl_3$, 100 MHz

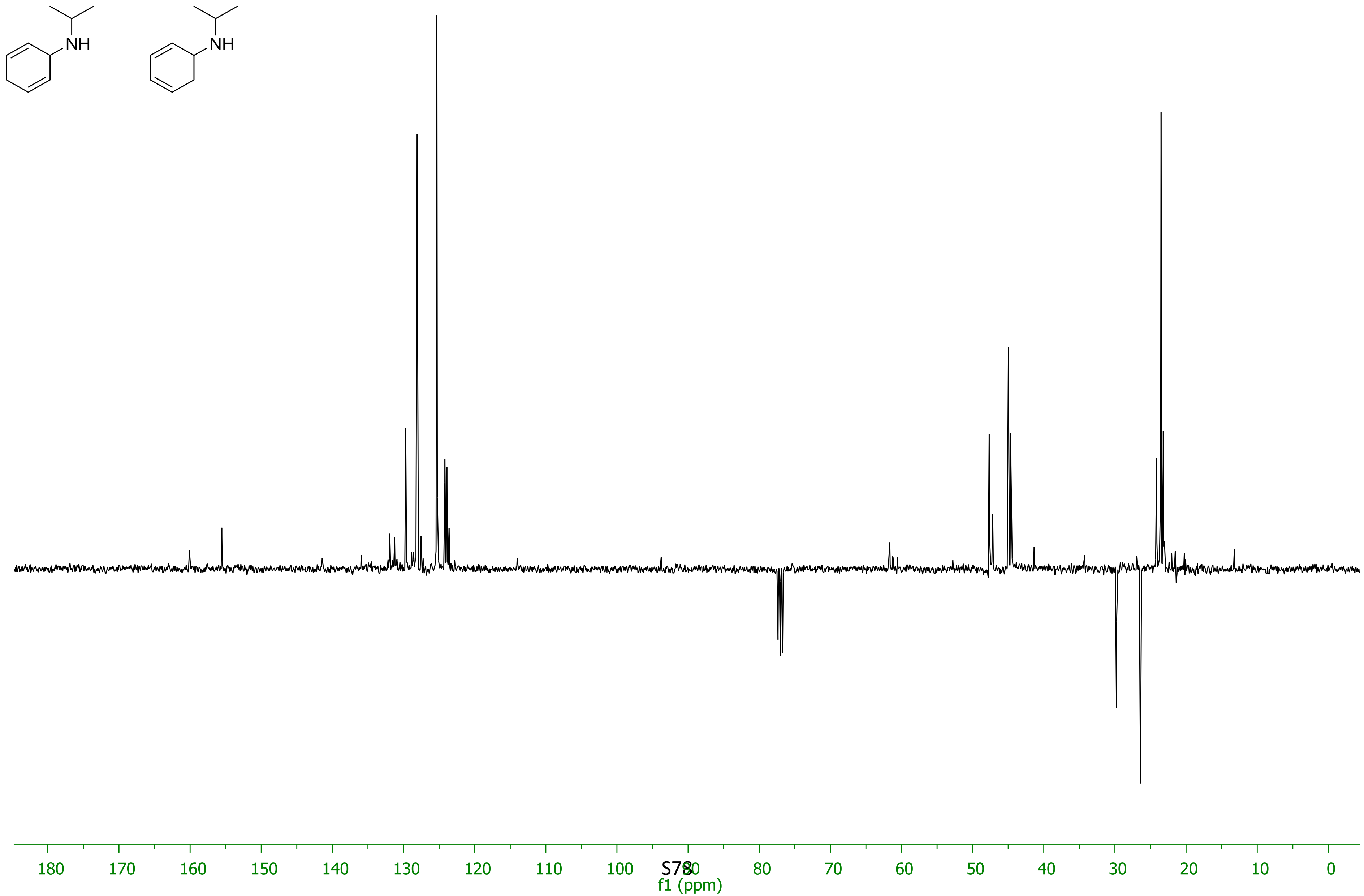

Diethyldithiocarbamic acid-[isopropyl(cyclohexa-2,5-dien-1-yl)carbamic acid]-thioanhydride **37**; $CDCl_3$, 400 MHz

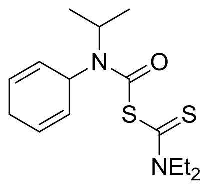

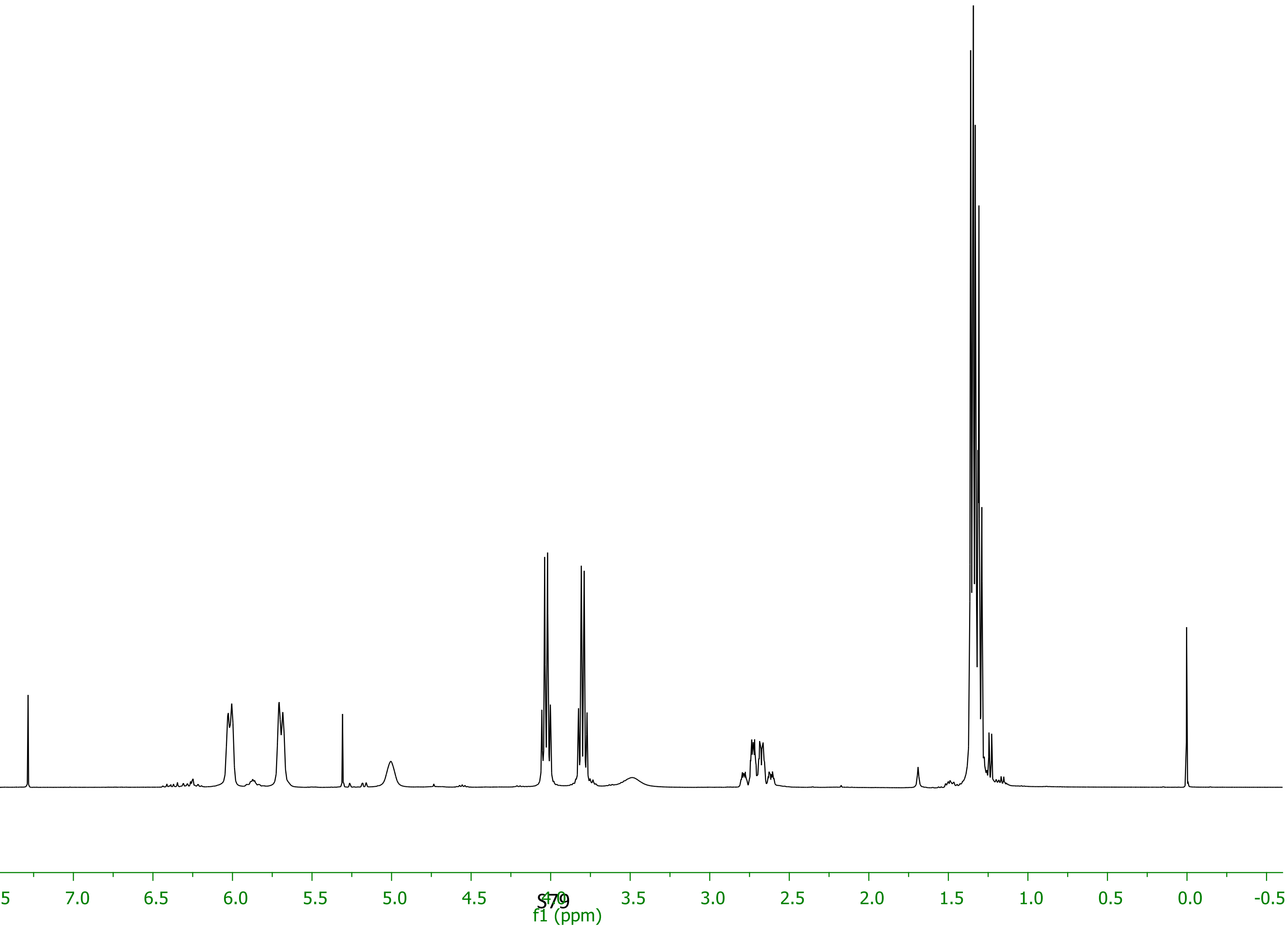

Diethyldithiocarbamic acid-[isopropyl(cyclohexa-2,5-dien-1-yl)carbamic acid]-thioanhydride **37**; $CDCl_3$, 100 MHz

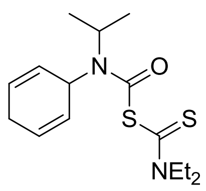

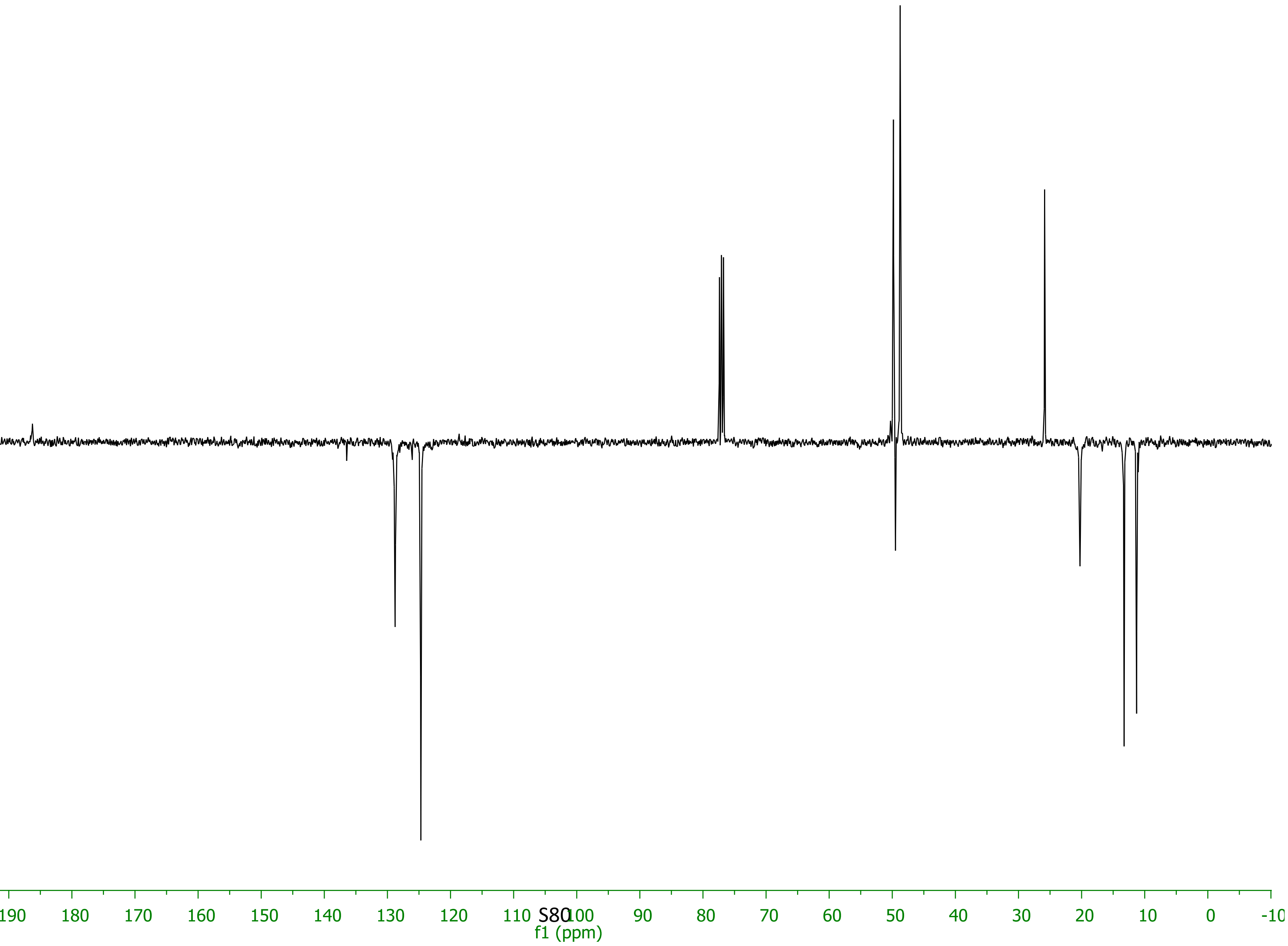

7-Isopropyl-8-oxo-7-azabicyclo[4.2.0]oct-4-en-2-yl diethylcarbamodithioate **52**; $CDCl_3$, 400 MHz

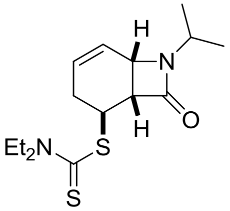

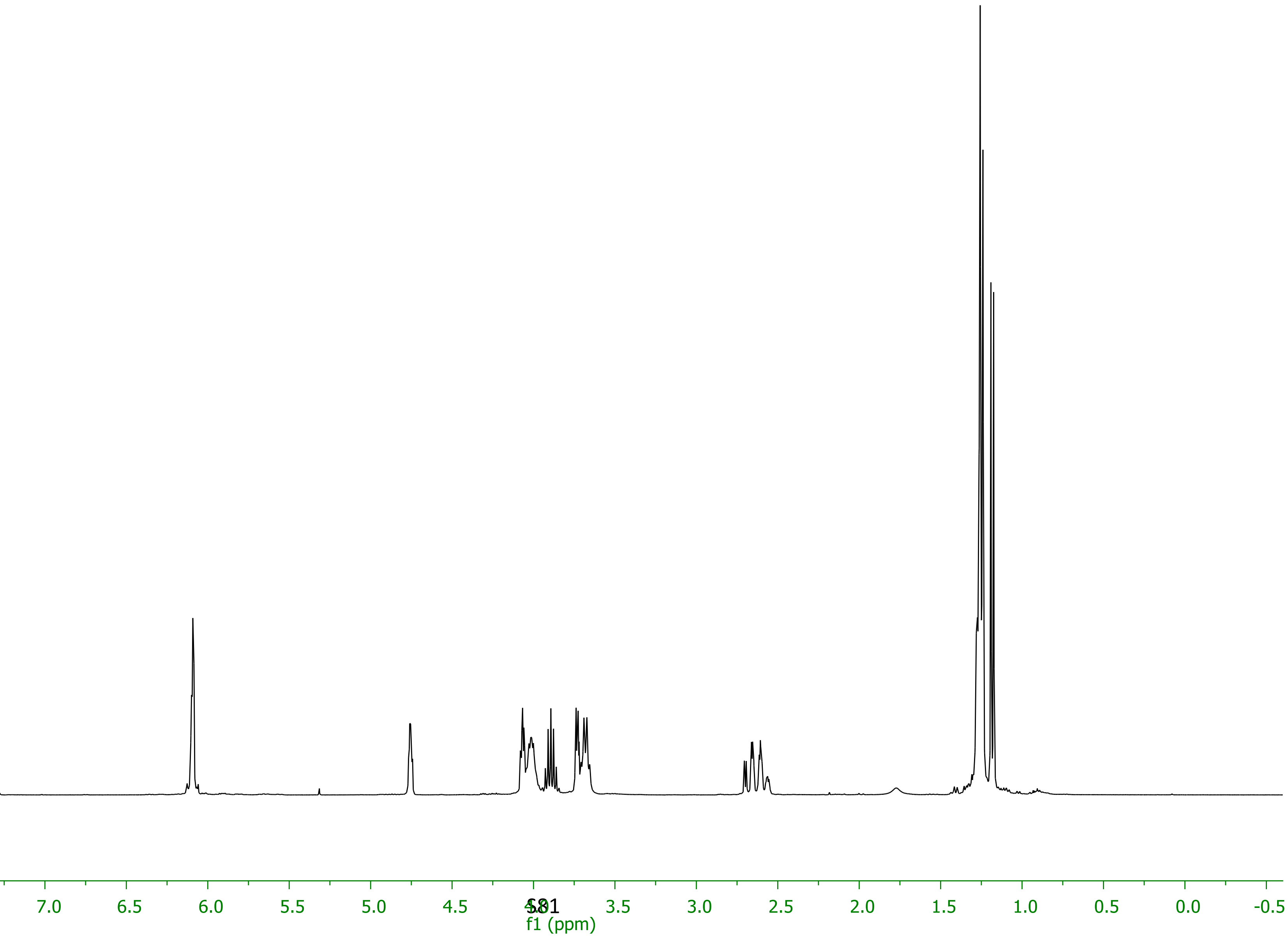

7-Isopropyl-8-oxo-7-azabicyclo[4.2.0]oct-4-en-2-yl diethylcarbamodithioate **52**; $CDCl_3$, 100 MHz

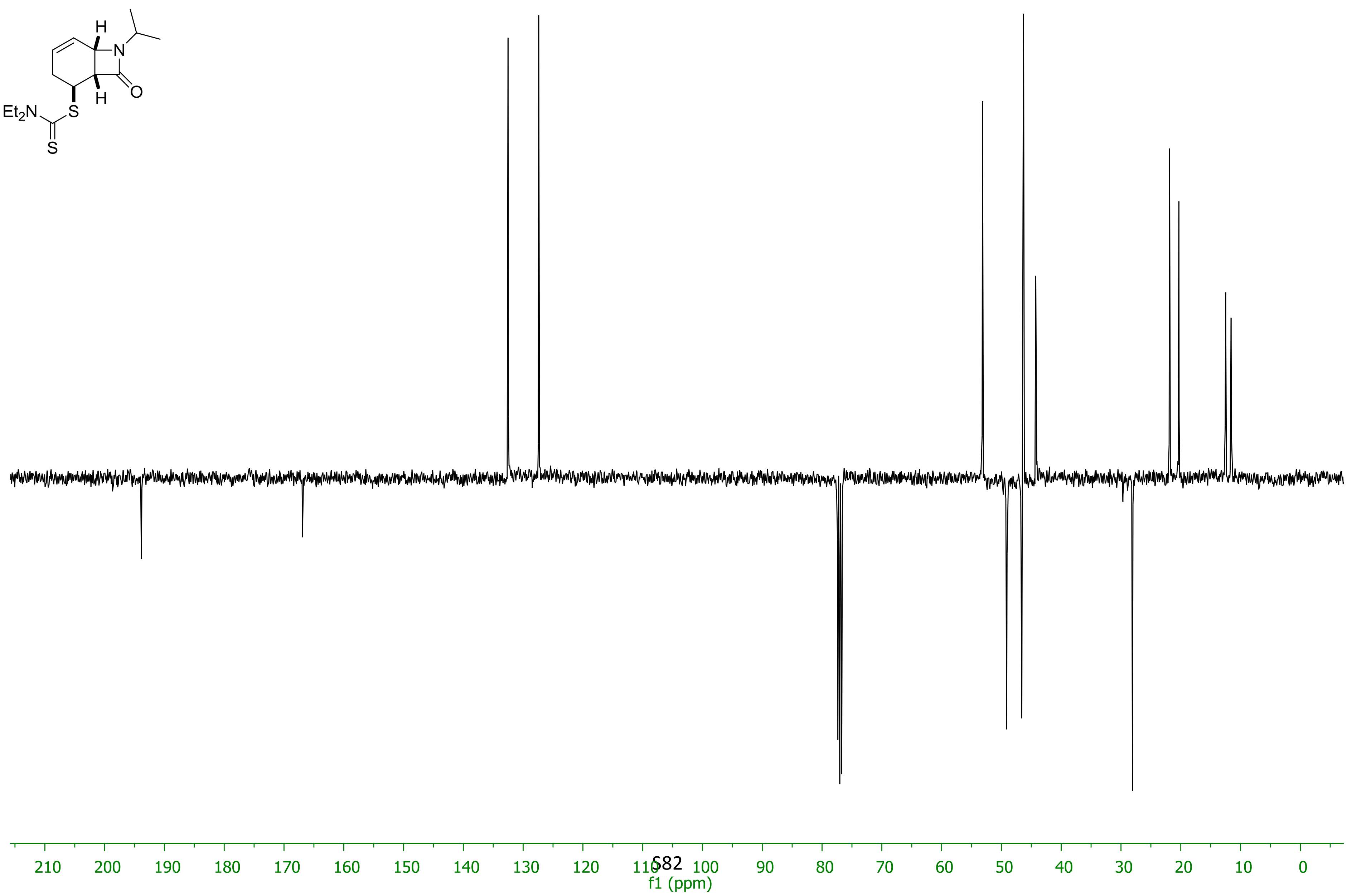

N-Allyl-4-methoxyaniline **98**; $CDCl_3$, 300 MHz

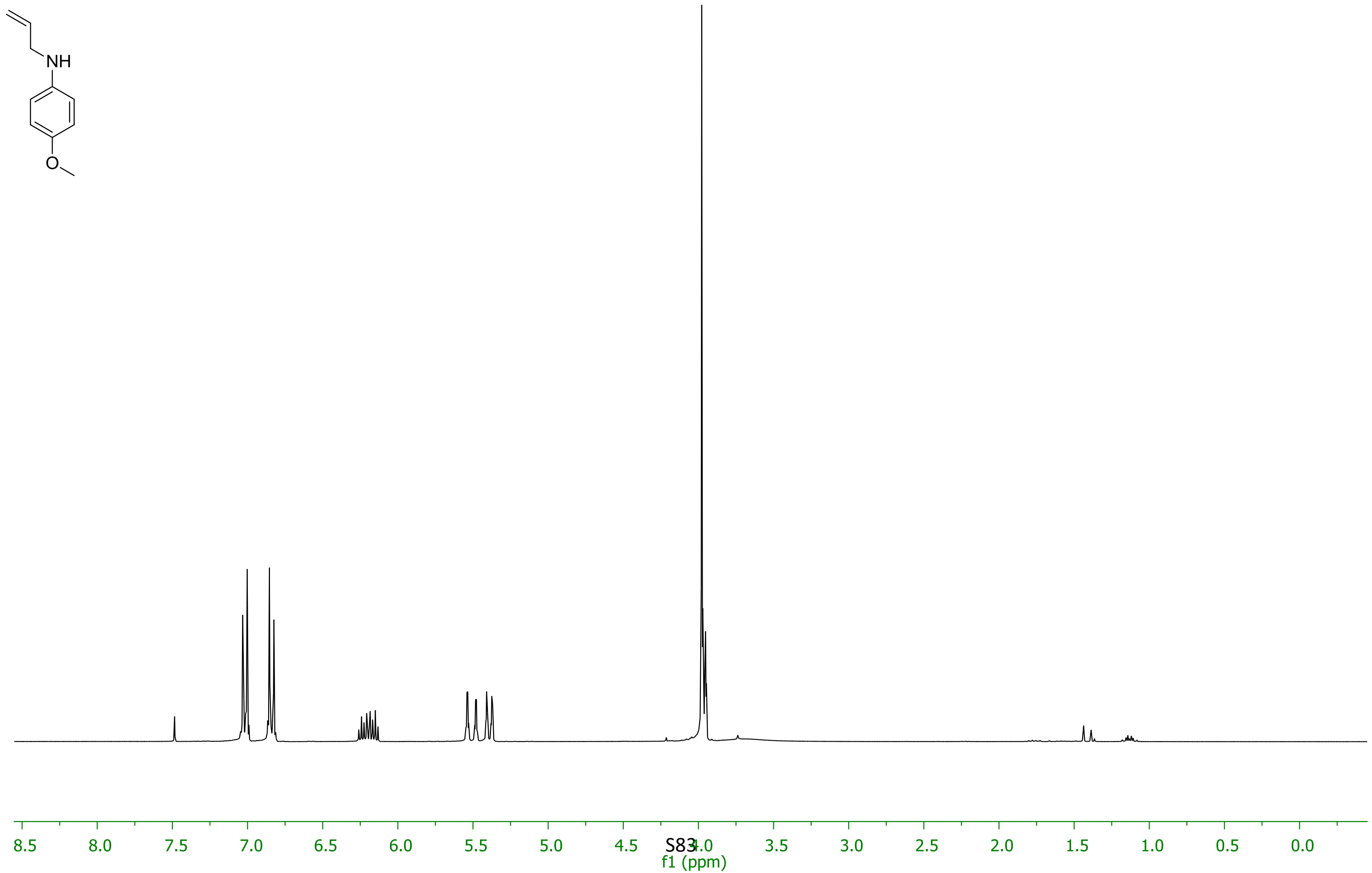

N-Allyl-4-methoxyaniline **98**; $CDCl_3$, 100 MHz

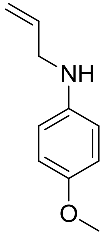

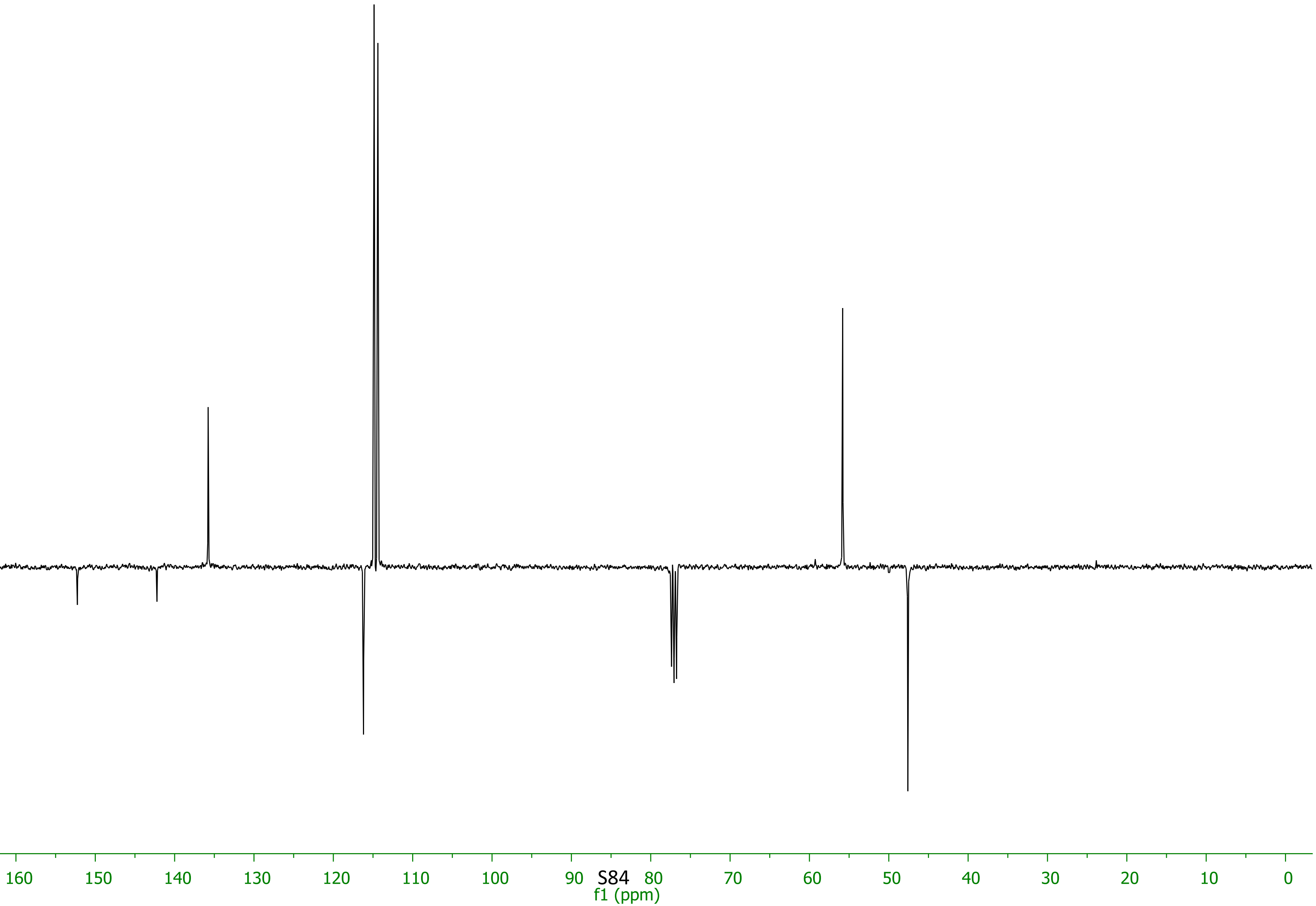

Allyl(4-methoxyphenyl)carbamic chloride **99**; $CDCl_3$, 300 MHz

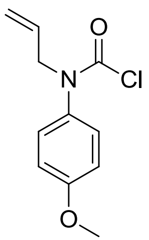

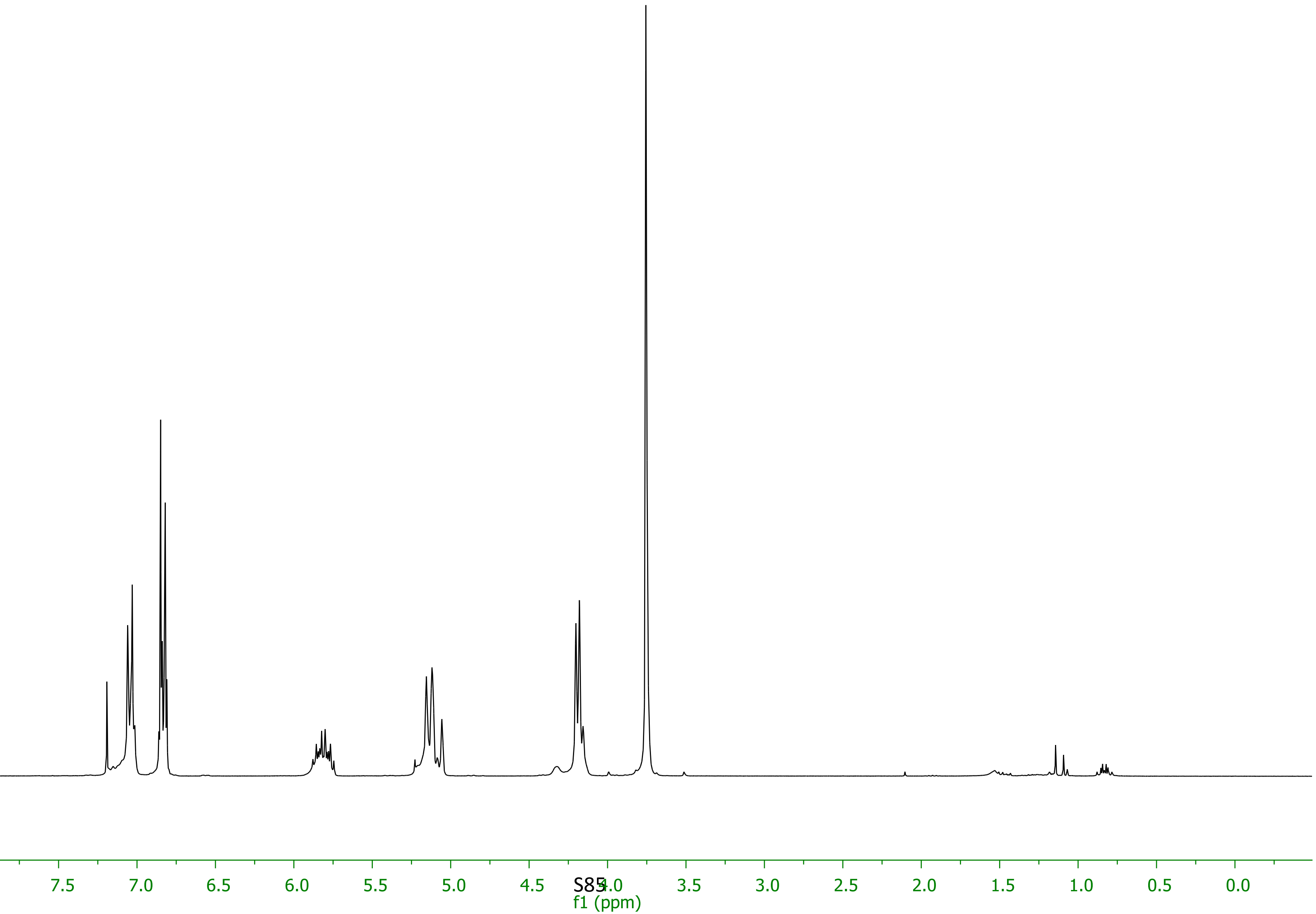

Allyl(4-methoxyphenyl)carbamic chloride **99**; $CDCl_3$, 100 MHz

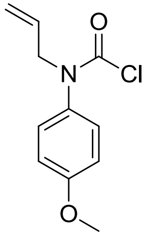

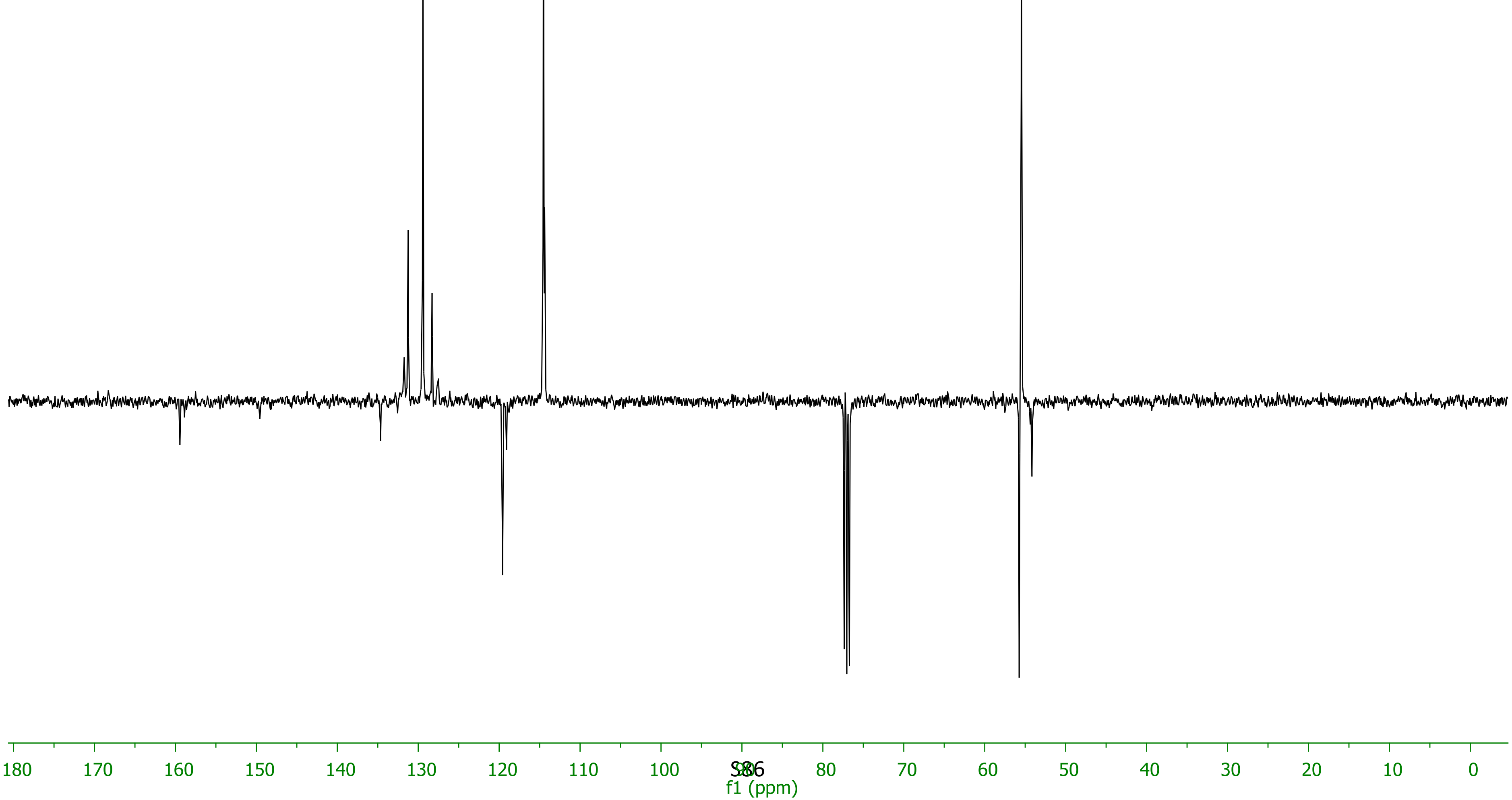

Diethylthiocarbamic acid-[allyl(4-methoxyphenyl)carbamic acid]thioanhydride **38**; $CDCl_3$, 400 MHz

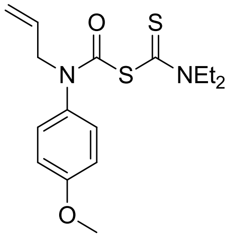

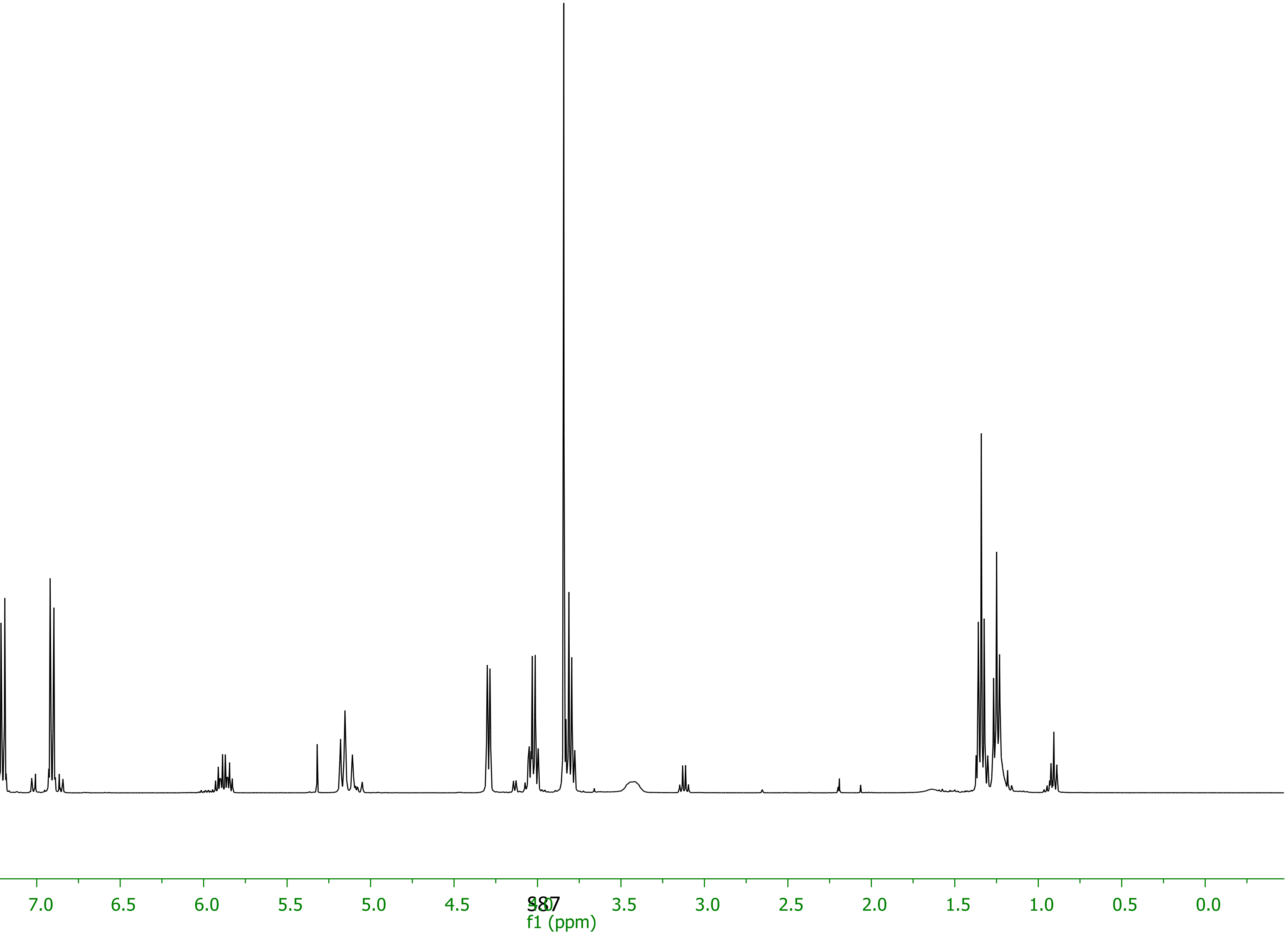

Diethylthiocarbamic acid-[allyl(4-methoxyphenyl)carbamic acid]thioanhydride **38**; $CDCl_3$, 100 MHz

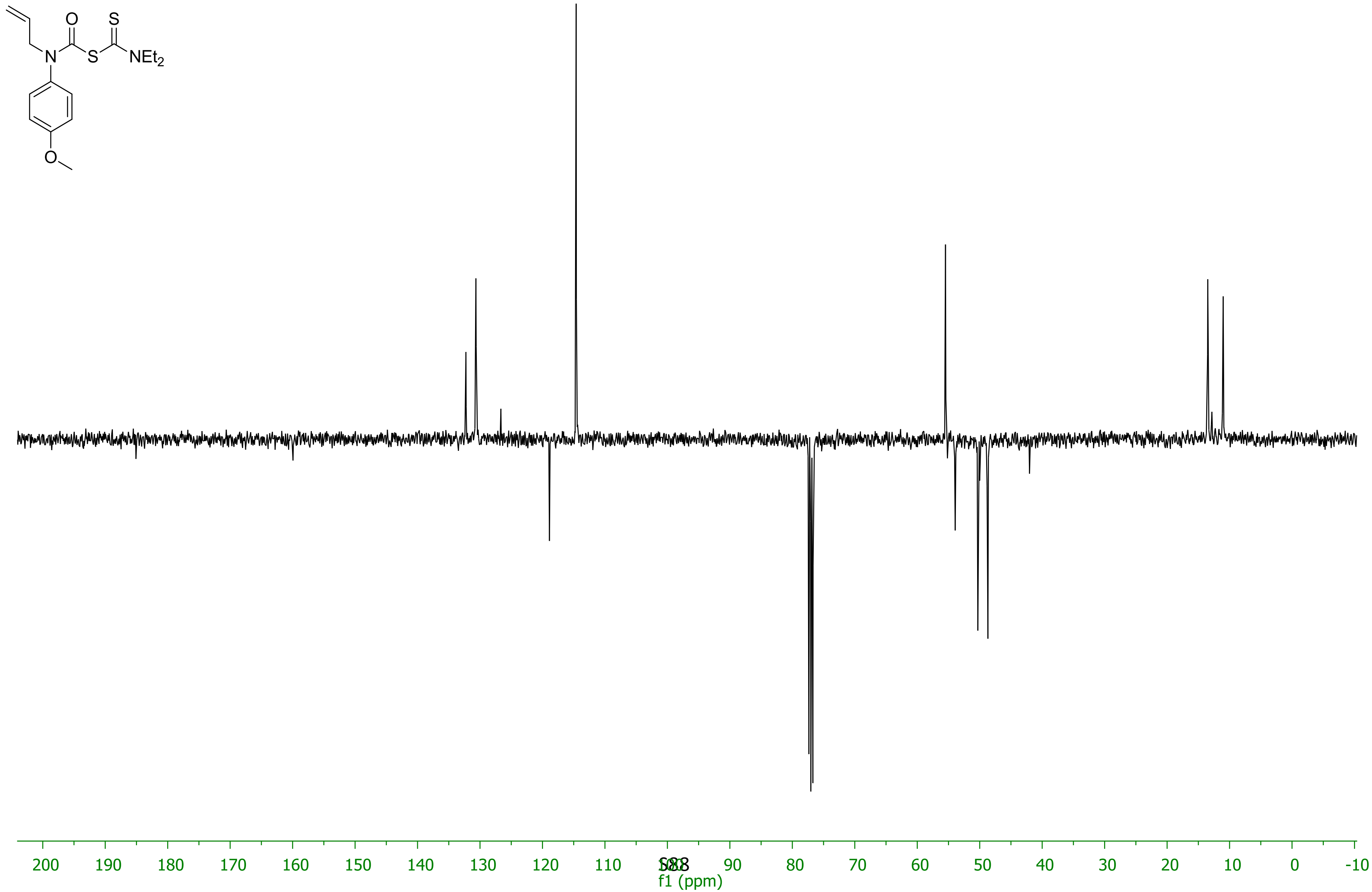

1-(4-Methoxyphenyl)-2-oxoazetidin-3-yl)methyl diethylcarbamodithioate **53**; $CDCl_3$, 400 MHz

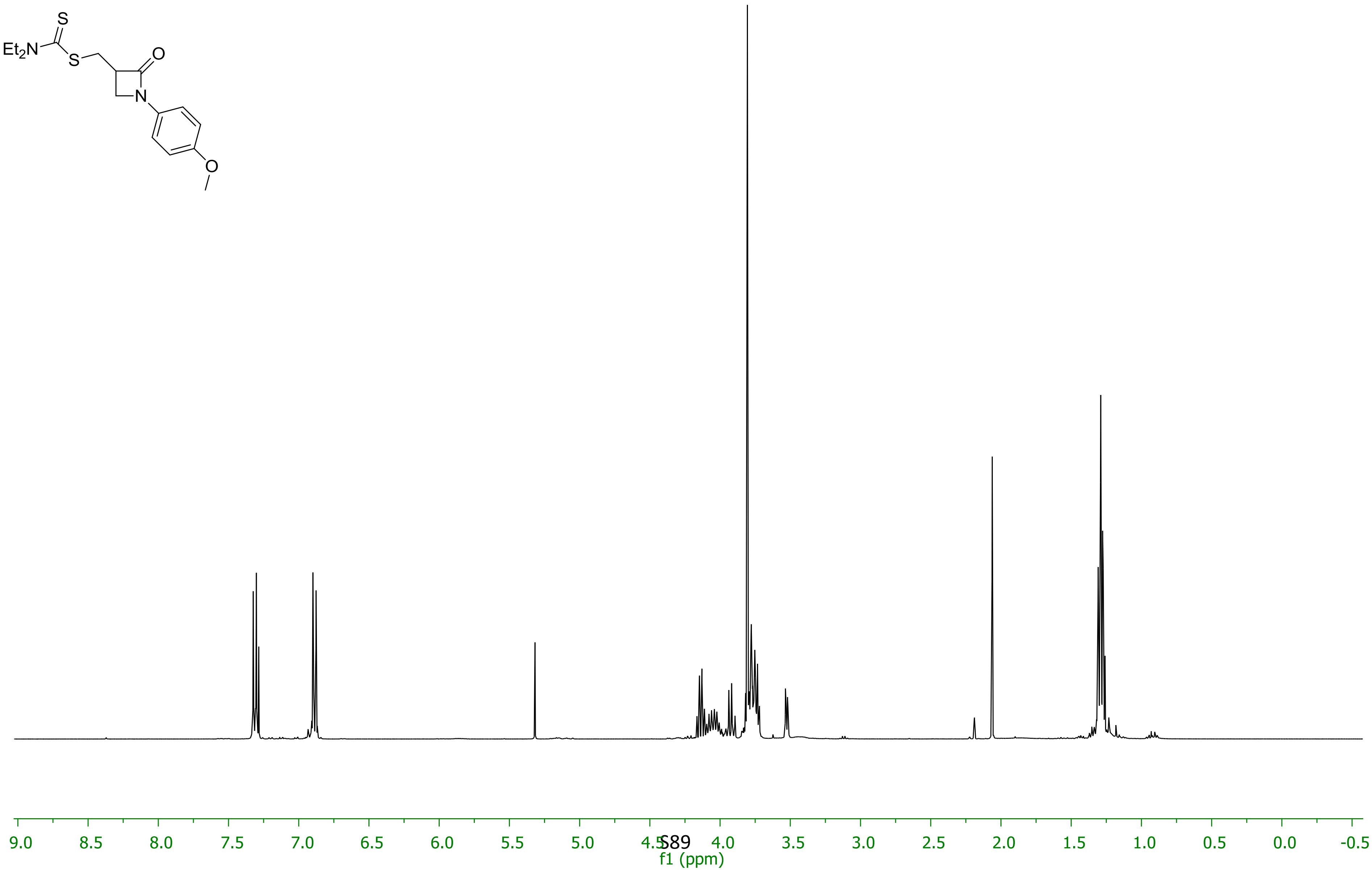

1-(4-Methoxyphenyl)-2-oxoazetidin-3-yl)methyl diethylcarbamodithioate **53**; $CDCl_3$, 100 MHz

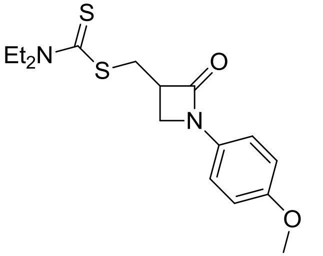

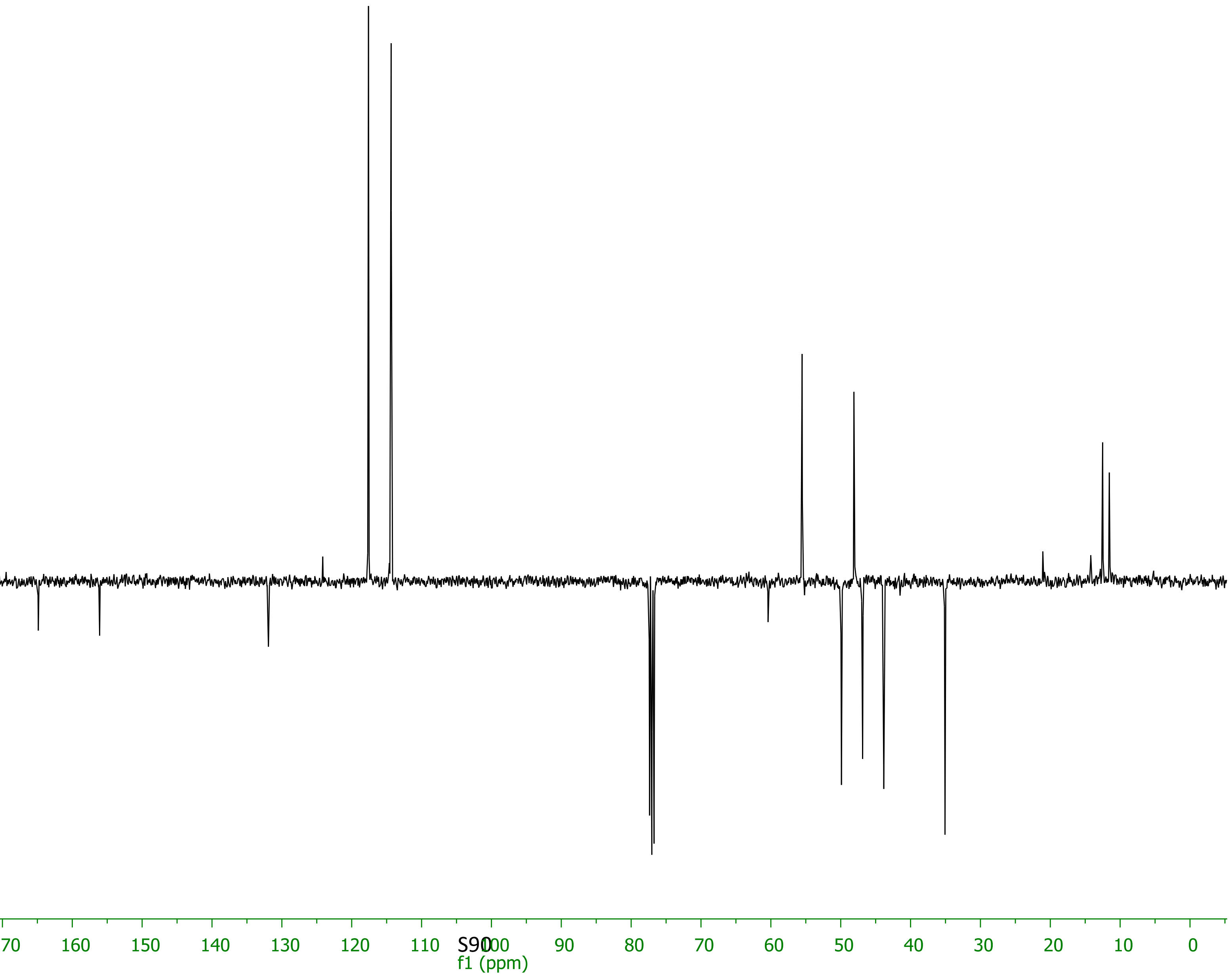

N-Benzyl-3,5,5-trimethylcyclohex-2-enamine **100**; $CDCl_3$, 400 MHz

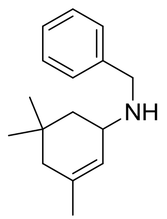

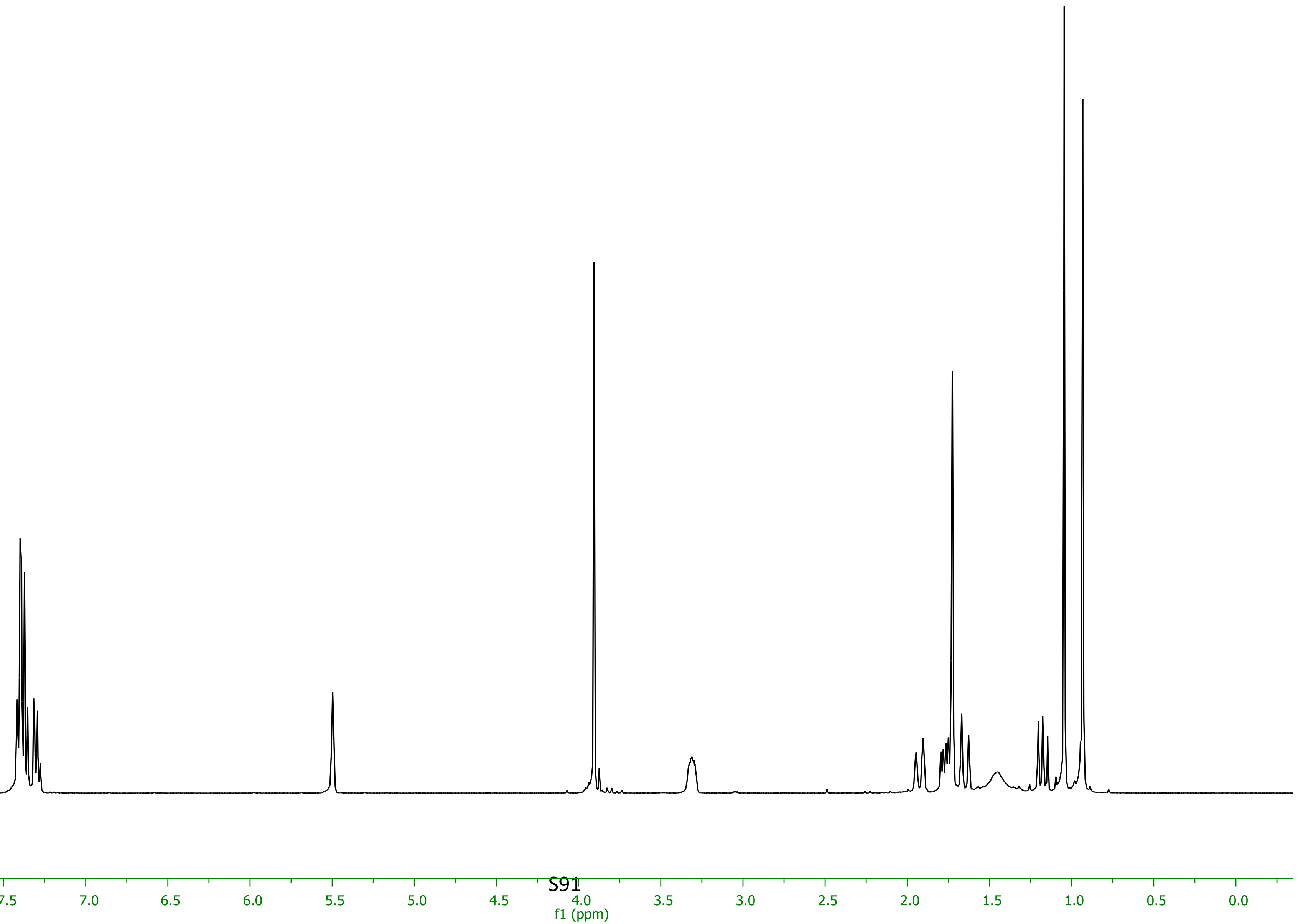

N-Benzyl-3,5,5-trimethylcyclohex-2-enamine **100**; $CDCl_3$, 100 MHz

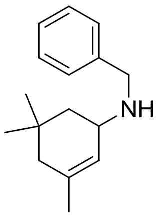

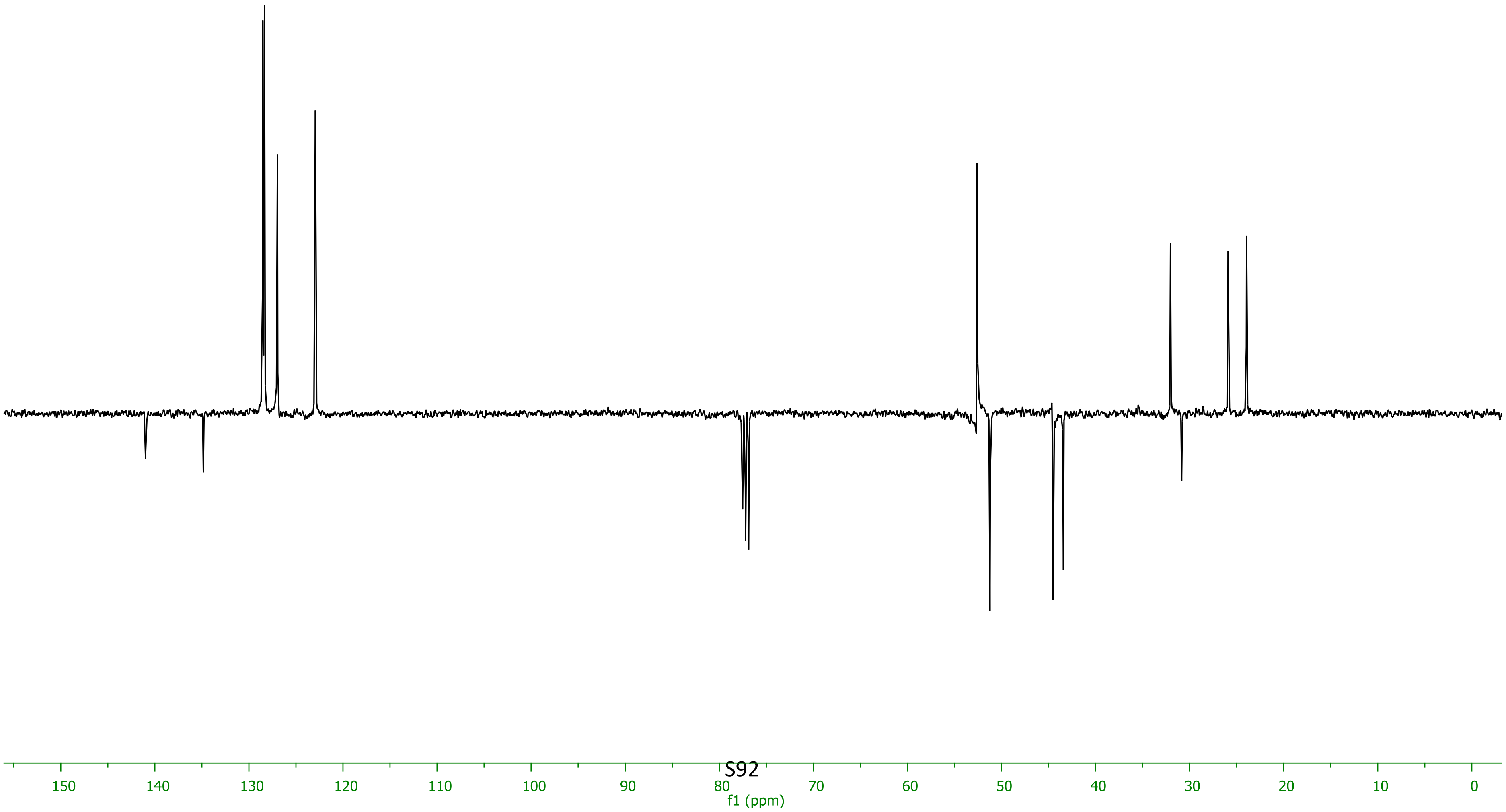

Benzyl(3,5,5-trimethylcyclohex-2-en-1-yl)carbamic chloride **101**; $CDCl_3$, 400 MHz

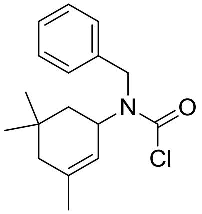

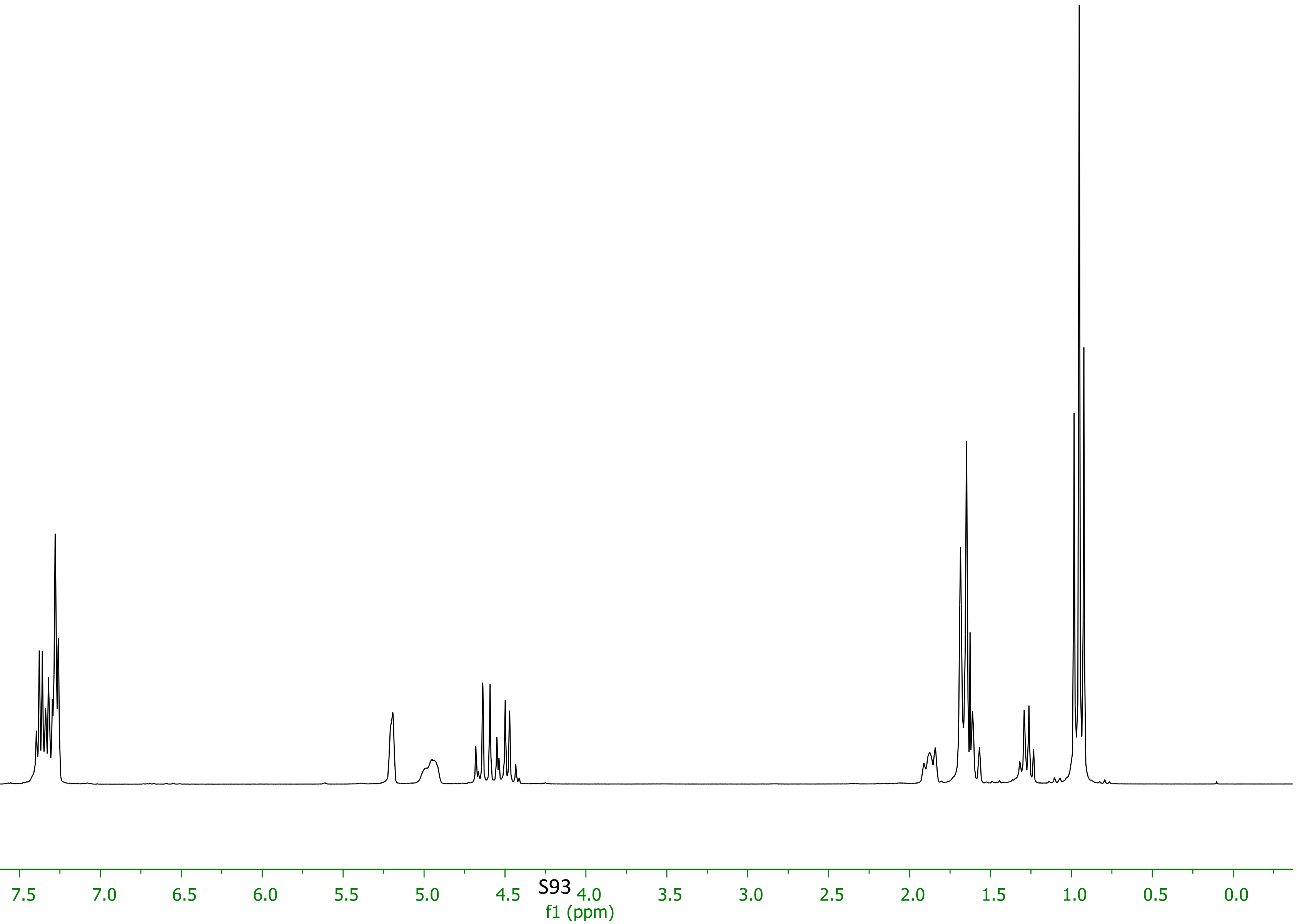

Benzyl(3,5,5-trimethylcyclohex-2-en-1-yl)carbamic chloride **101**; $CDCl_3$, 100 MHz

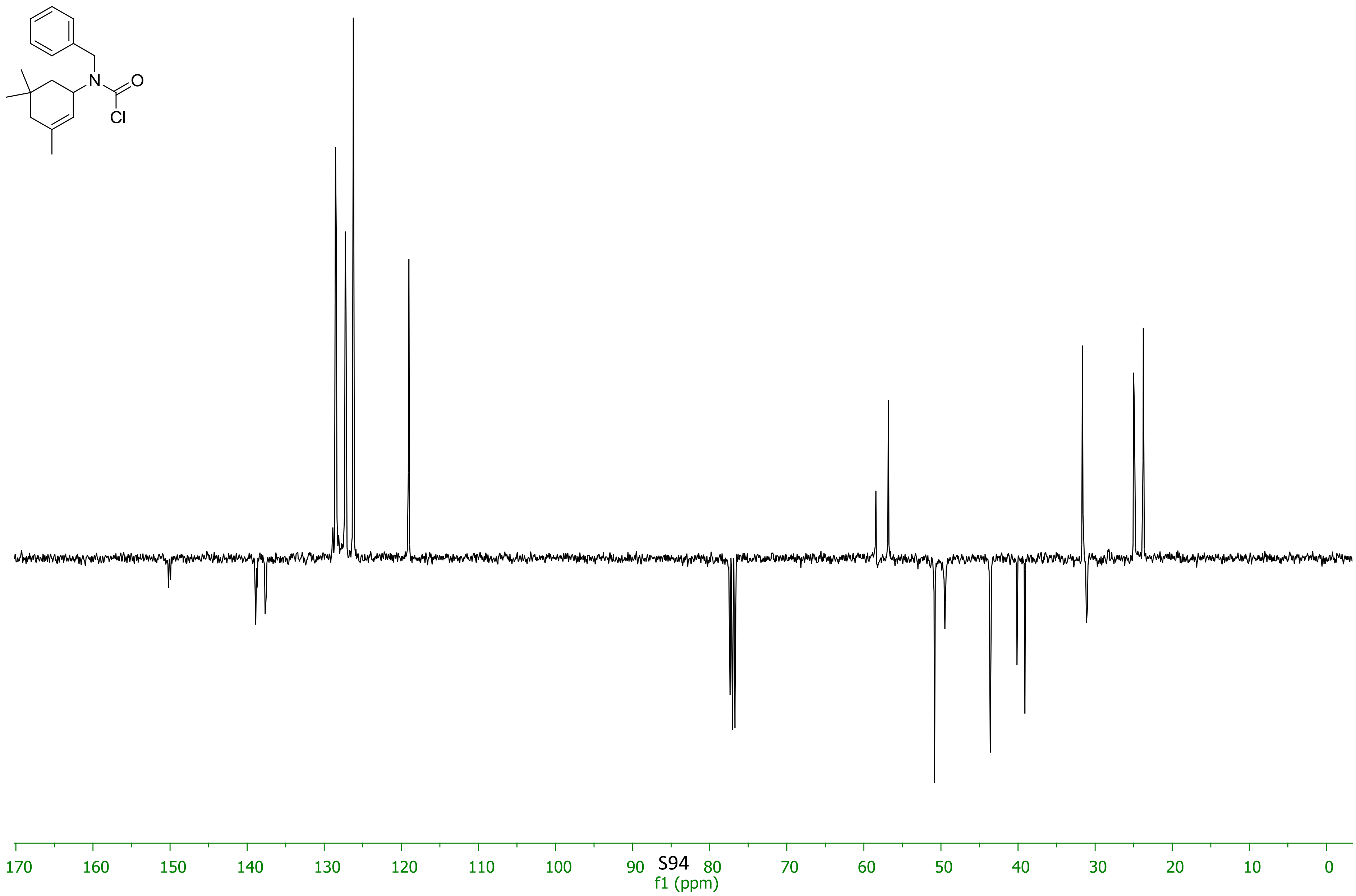

Diethyldithiocarbamic acid-[benzyl(3,5,5-trimethylcyclohex-2-en-1-yl)carbamic acid]-thioanhydride **39**; $CDCl_3$, 400 MHz

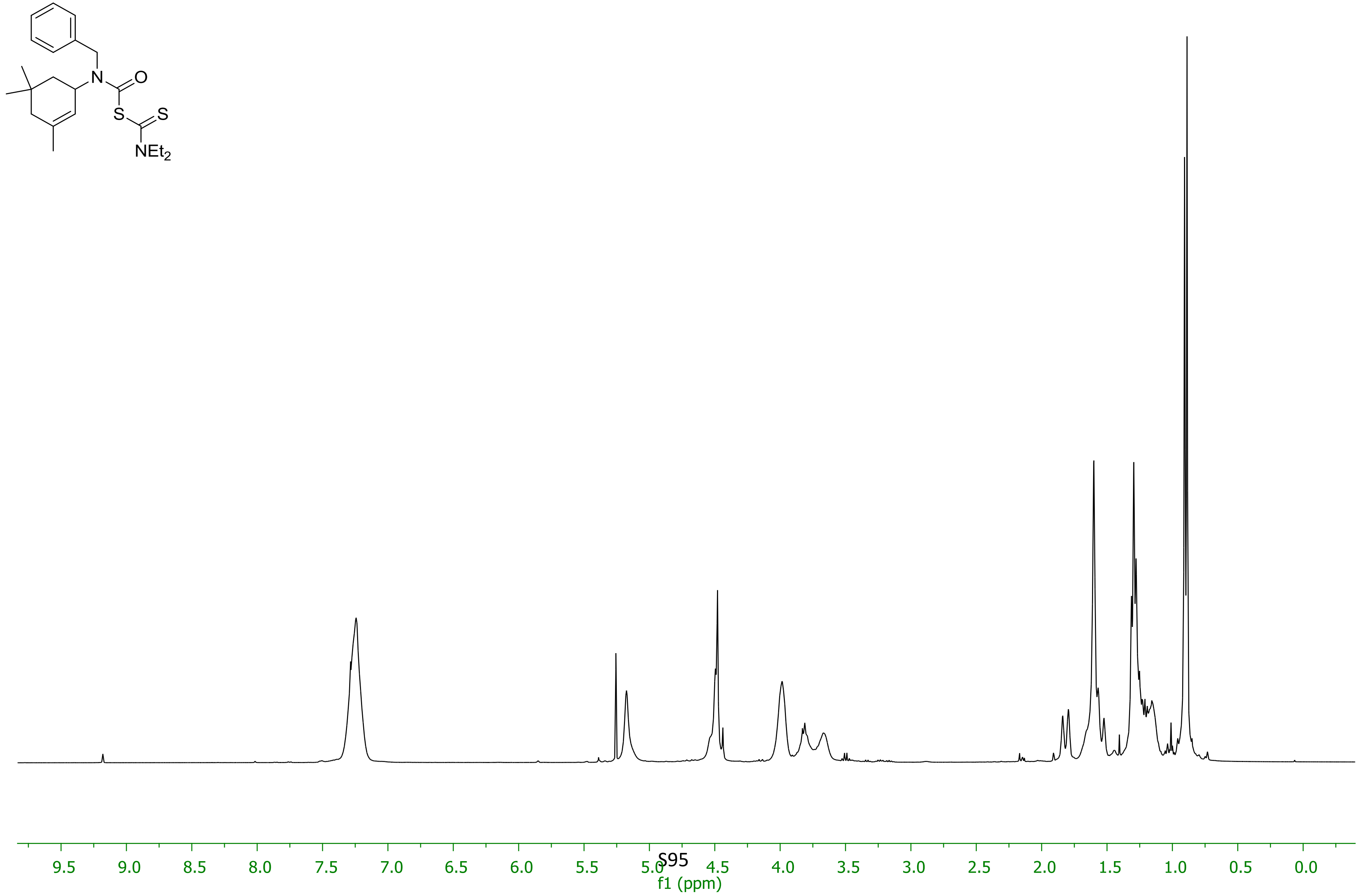

Diethyldithiocarbamic acid-[benzyl(3,5,5-trimethylcyclohex-2-en-1-yl)carbamic acid]-thioanhydride **39**; $CDCl_3$, 100 MHz

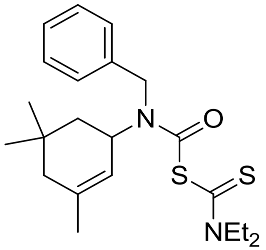

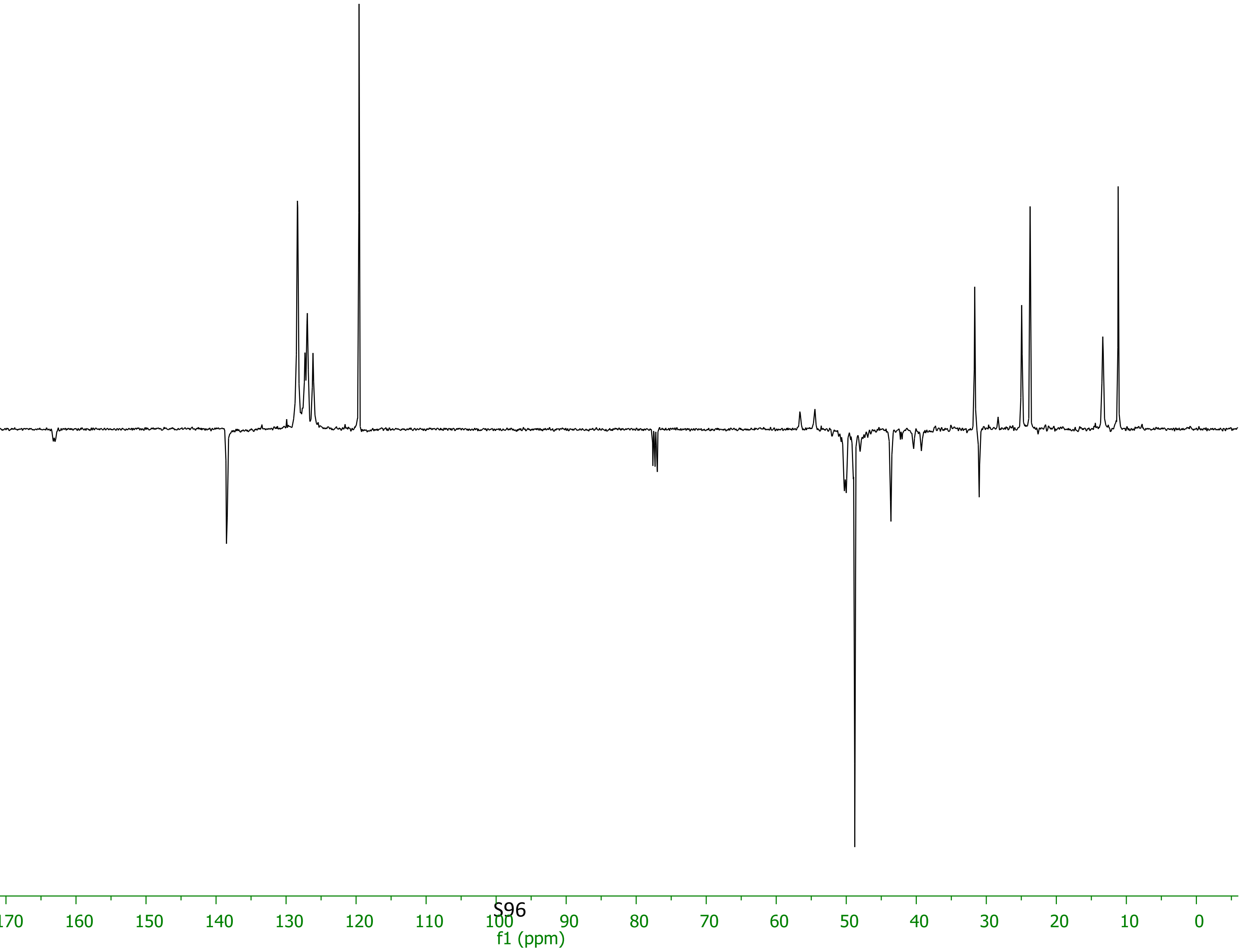

(±)-(1*R*,6*S*)-7-(4-Methoxybenzyl)-7-azabicyclo[4.2.0]oct-2-en-8-one **58a**; $CDCl_3$, 400 MHz

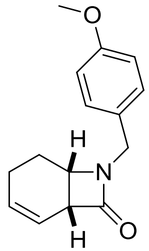

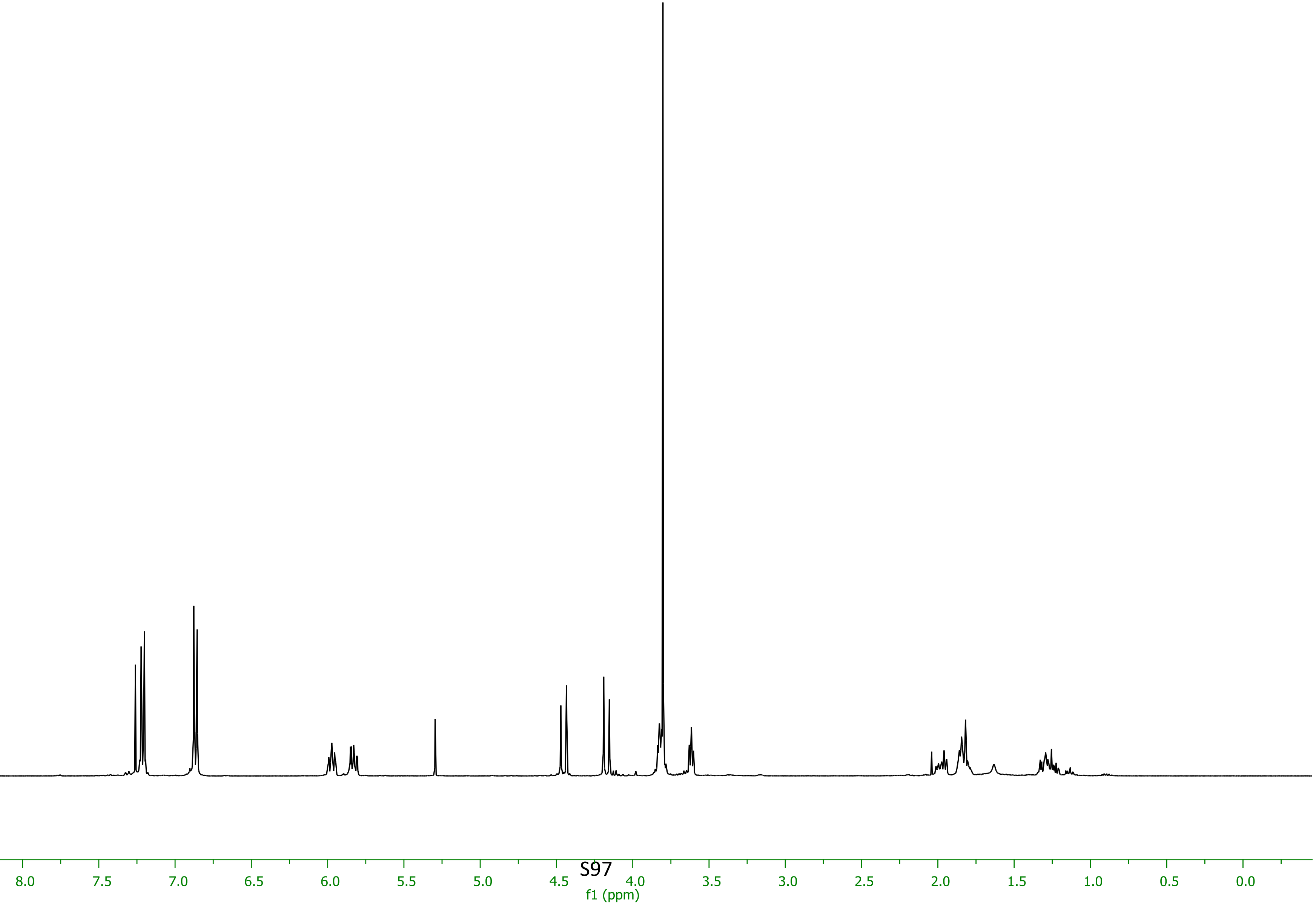

(±)-(1*R*,6*S*)-7-(4-Methoxybenzyl)-7-azabicyclo[4.2.0]oct-2-en-8-one **58a**; $CDCl_3$, 100 MHz

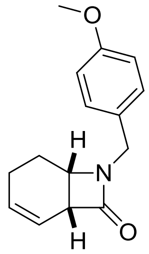

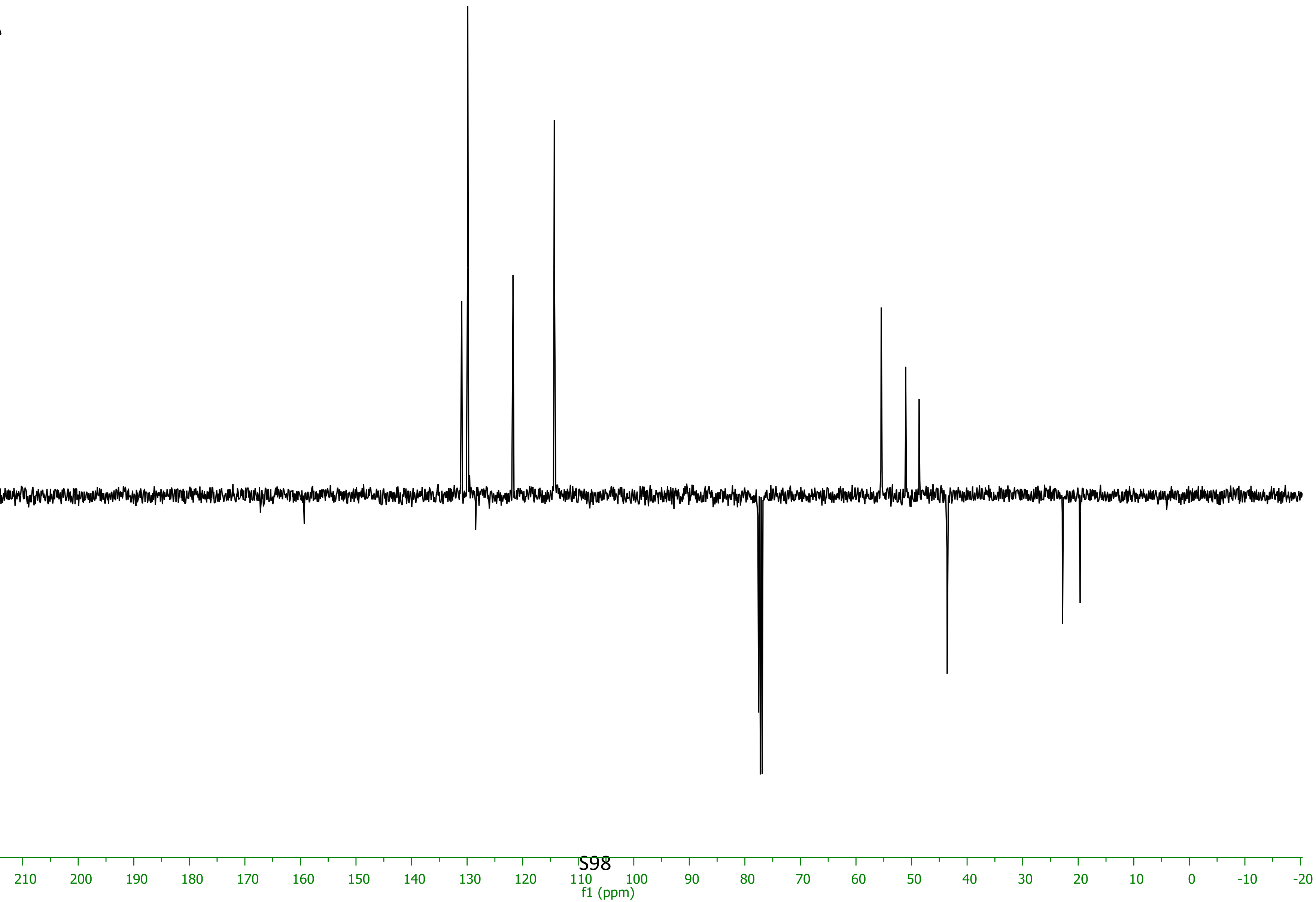

(±)-(1*R*,2*S*,5*S*)-6-(4-Methoxyphenyl)-1-methyl-7-oxo-6-azabicyclo[3.2.0]heptan-2-yl diethylcarbamodithioate **59**; $CDCl_3$, 400 MHz

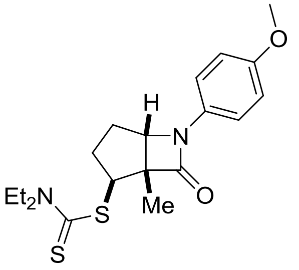

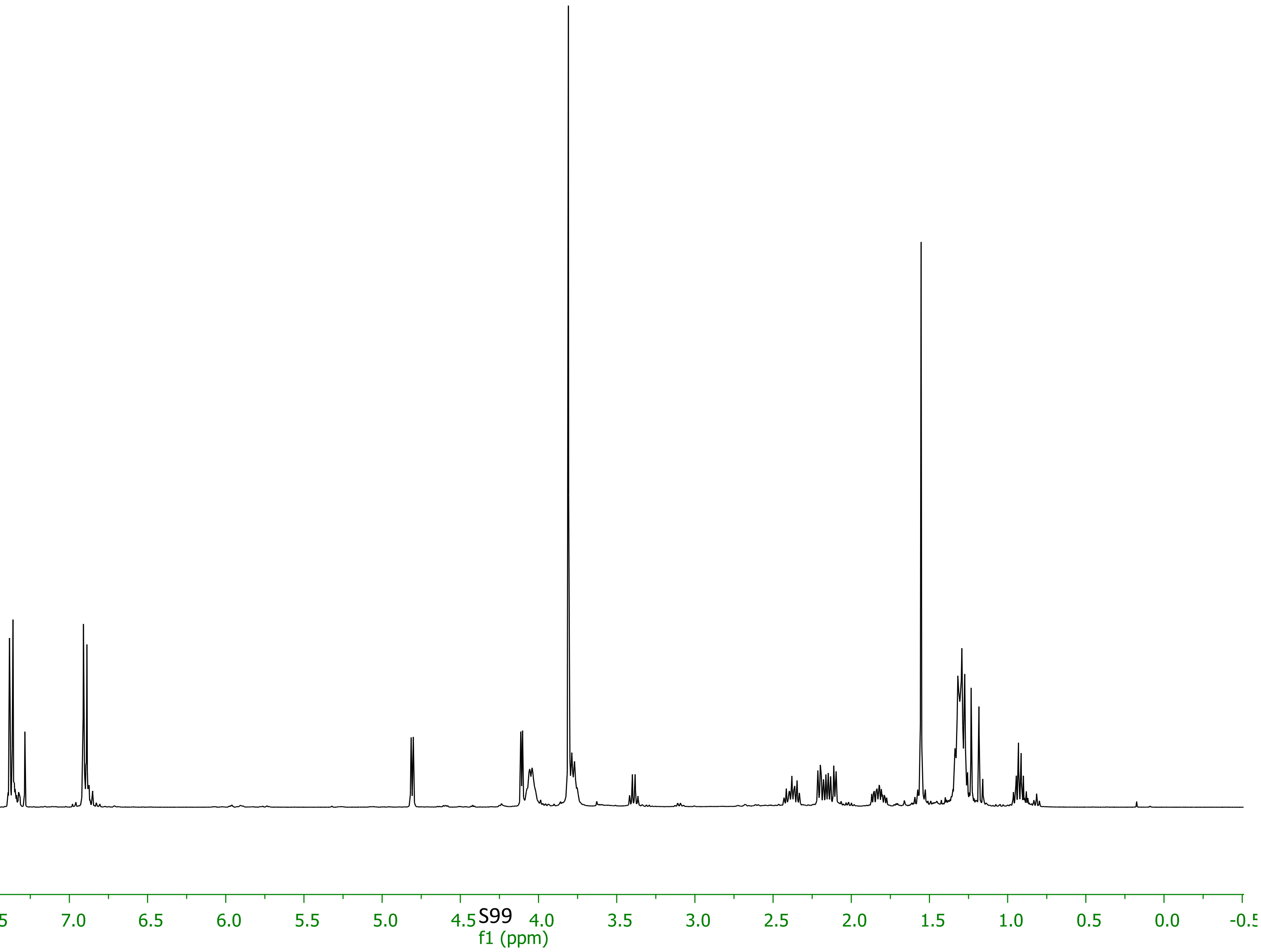

(±)-(1*R*,2*S*,5*S*)-6-(4-Methoxyphenyl)-1-methyl-7-oxo-6-azabicyclo[3.2.0]heptan-2-yl diethylcarbamodithioate **59**; $CDCl_3$, 100 MHz

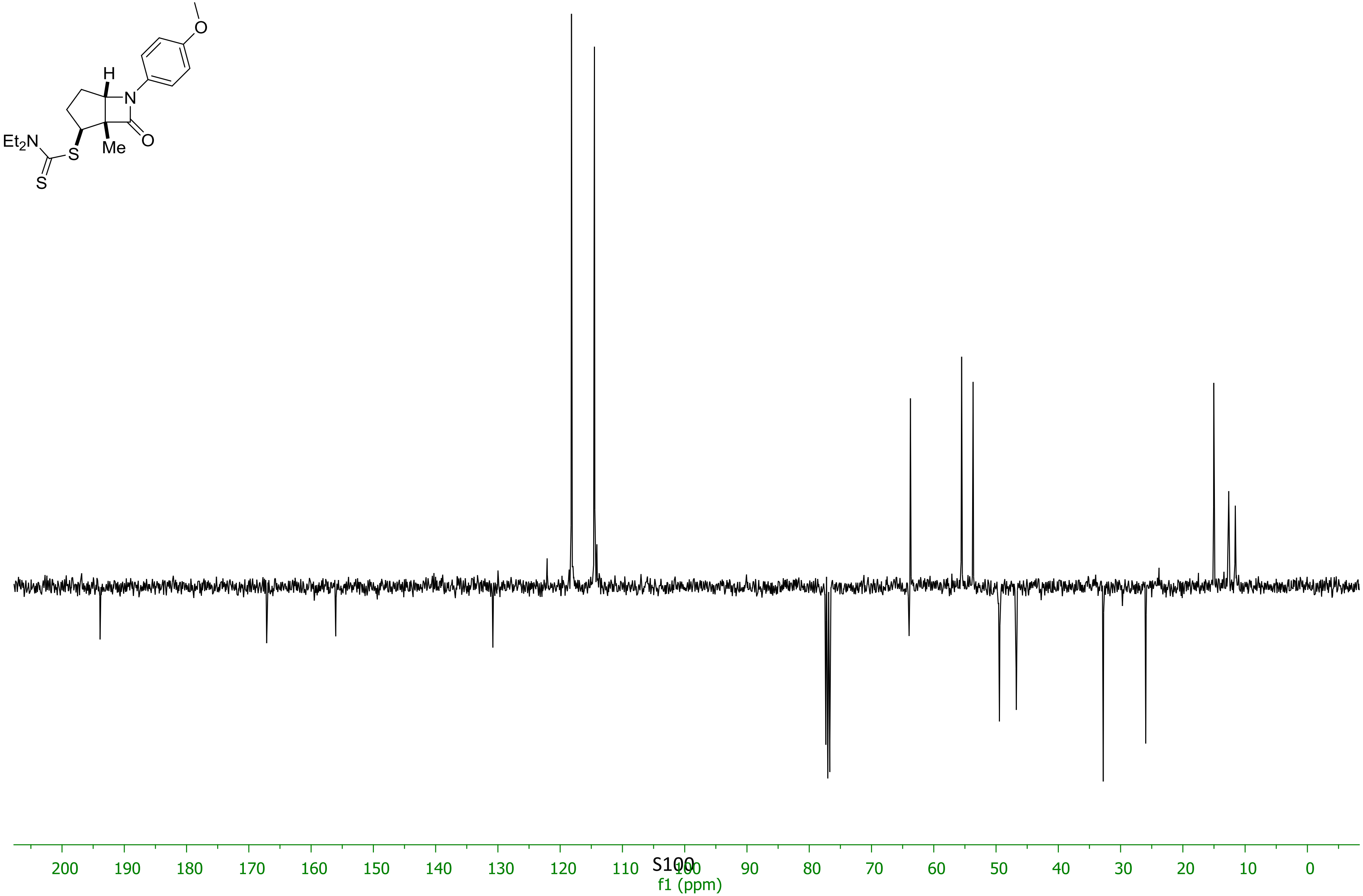

(±)-(1*R*,5*S*)-8-(4-Methoxyphenyl)-6-azabicyclo[3.2.0]hept-2-en-7-one **60**; $CDCl_3$, 400 MHz

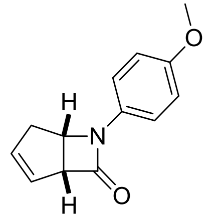

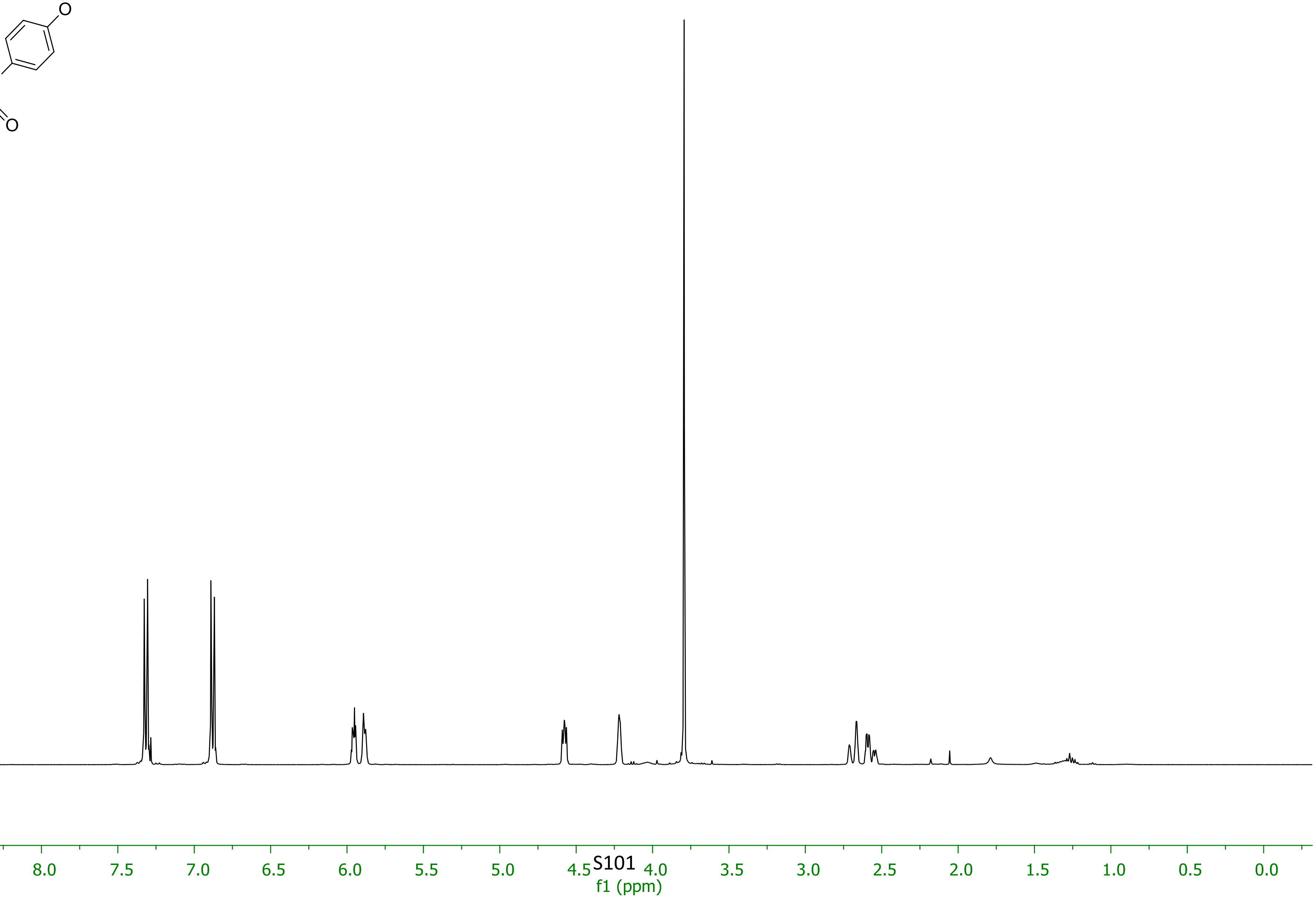

(±)-(1*R*,5*S*)-8-(4-Methoxyphenyl)-6-azabicyclo[3.2.0]hept-2-en-7-one **60**; $CDCl_3$, 100 MHz

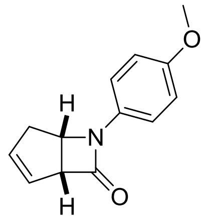

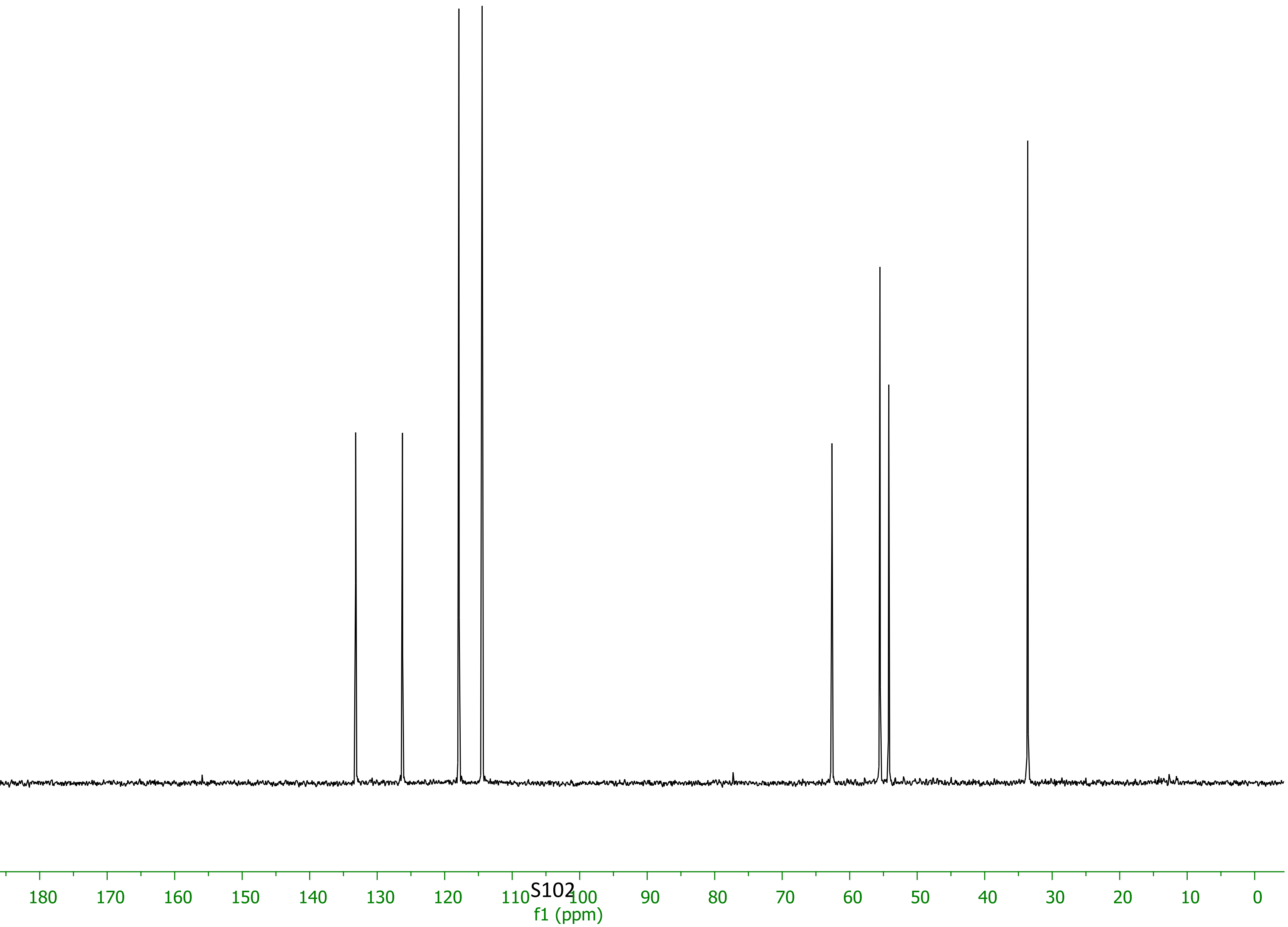

(±)-(1*S*,2*R*,7*R*)-3-(4-Methoxyphenyl)-3-azatricyclo[5.2.1.0^{2,5}]decan-5-en-4-one **63**; $CDCl_3$, 400 MHz

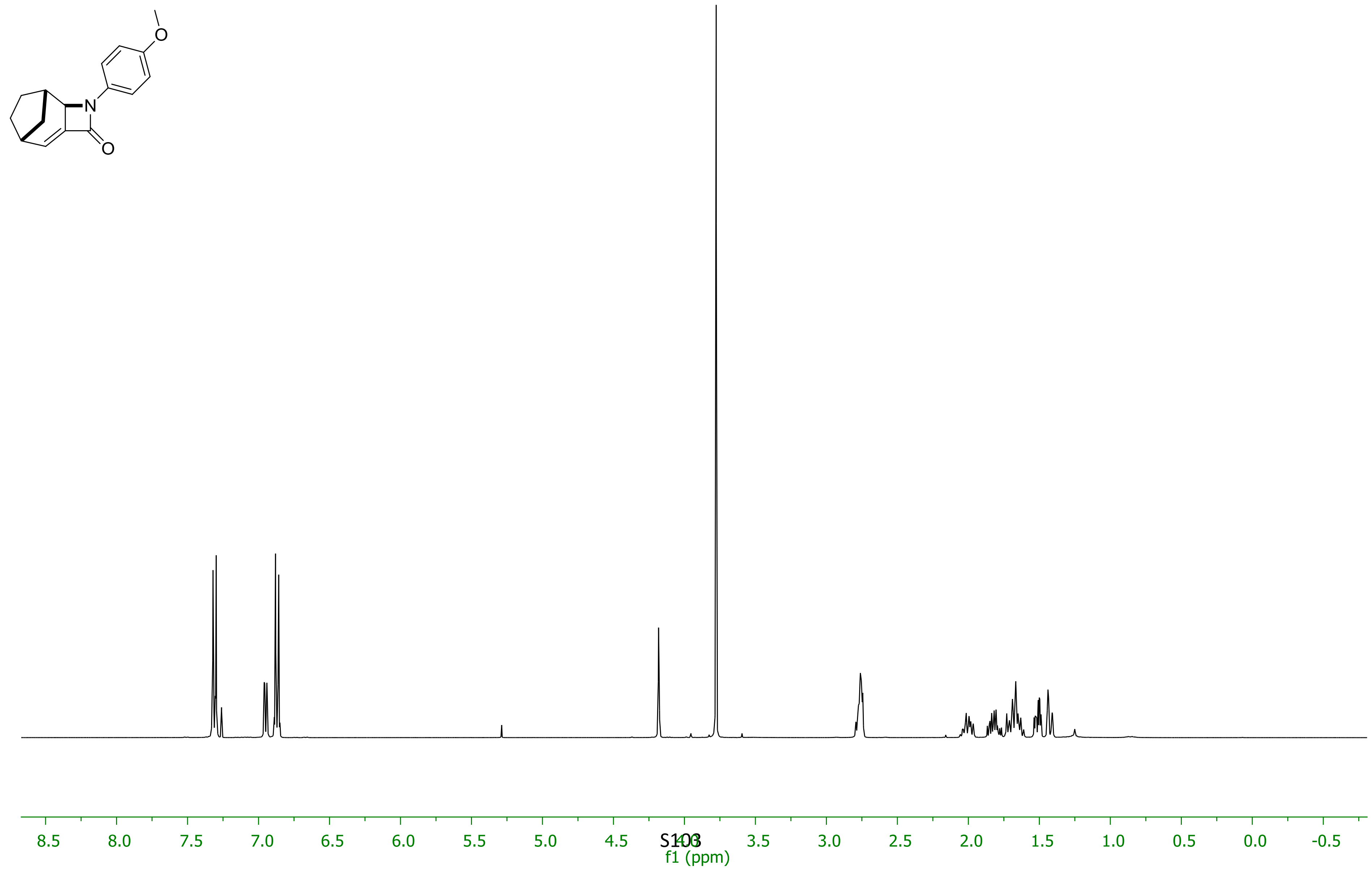

(±)-(1*S*,2*R*,7*R*)-3-(4-Methoxyphenyl)-3-azatricyclo[5.2.1.0^{2,5}]decan-5-en-4-one **63**; $CDCl_3$, 100 MHz

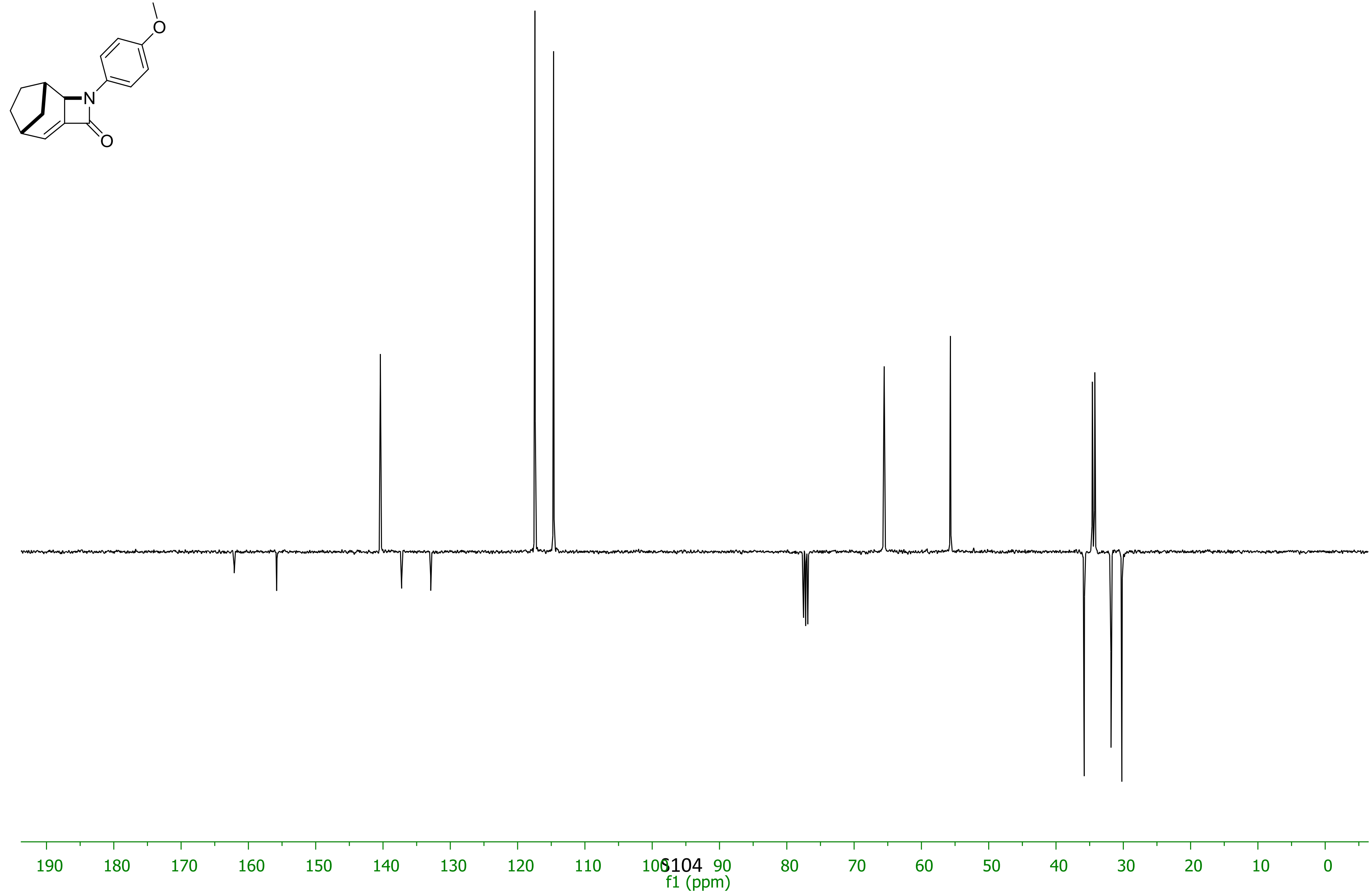

(±)-(1*S*,2*R*,5*S*,6*S*,7*R*)-3-(4-Methoxyphenyl)-5-methyl-4-oxo-3-azatricyclo[5.2.1.$0^{2,5}$] decan-6-yl diethyldithiocarbamate **64**; $CDCl_3$, 400 MHz

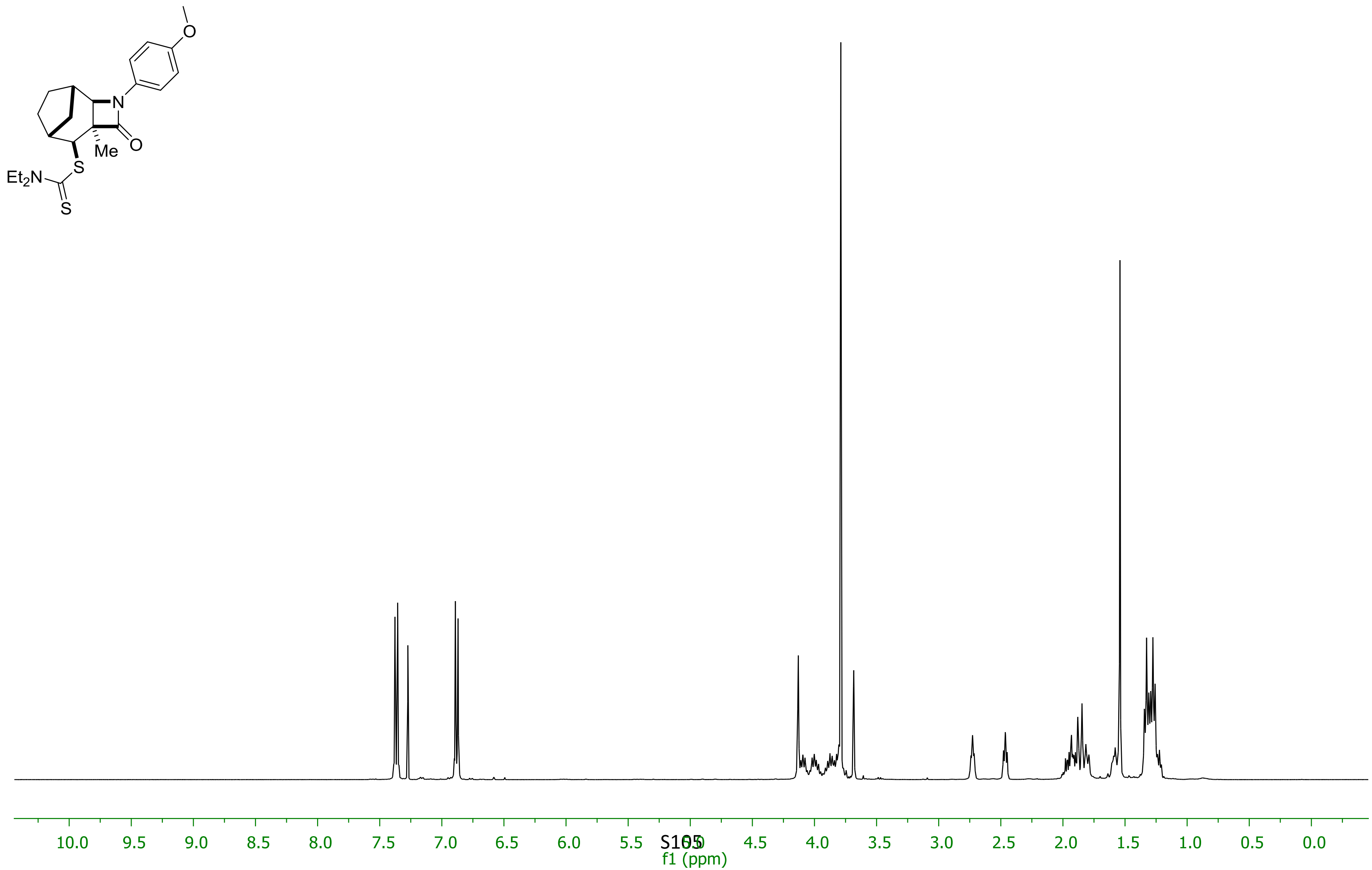

(±)-(1*S*,2*R*,5*S*,6*S*,7*R*)-3-(4-Methoxyphenyl)-5-methyl-4-oxo-3-azatricyclo[5.2.1.0^{2,5}] decan-6-yl diethyldithiocarbamate **64**; $CDCl_3$, 100 MHz

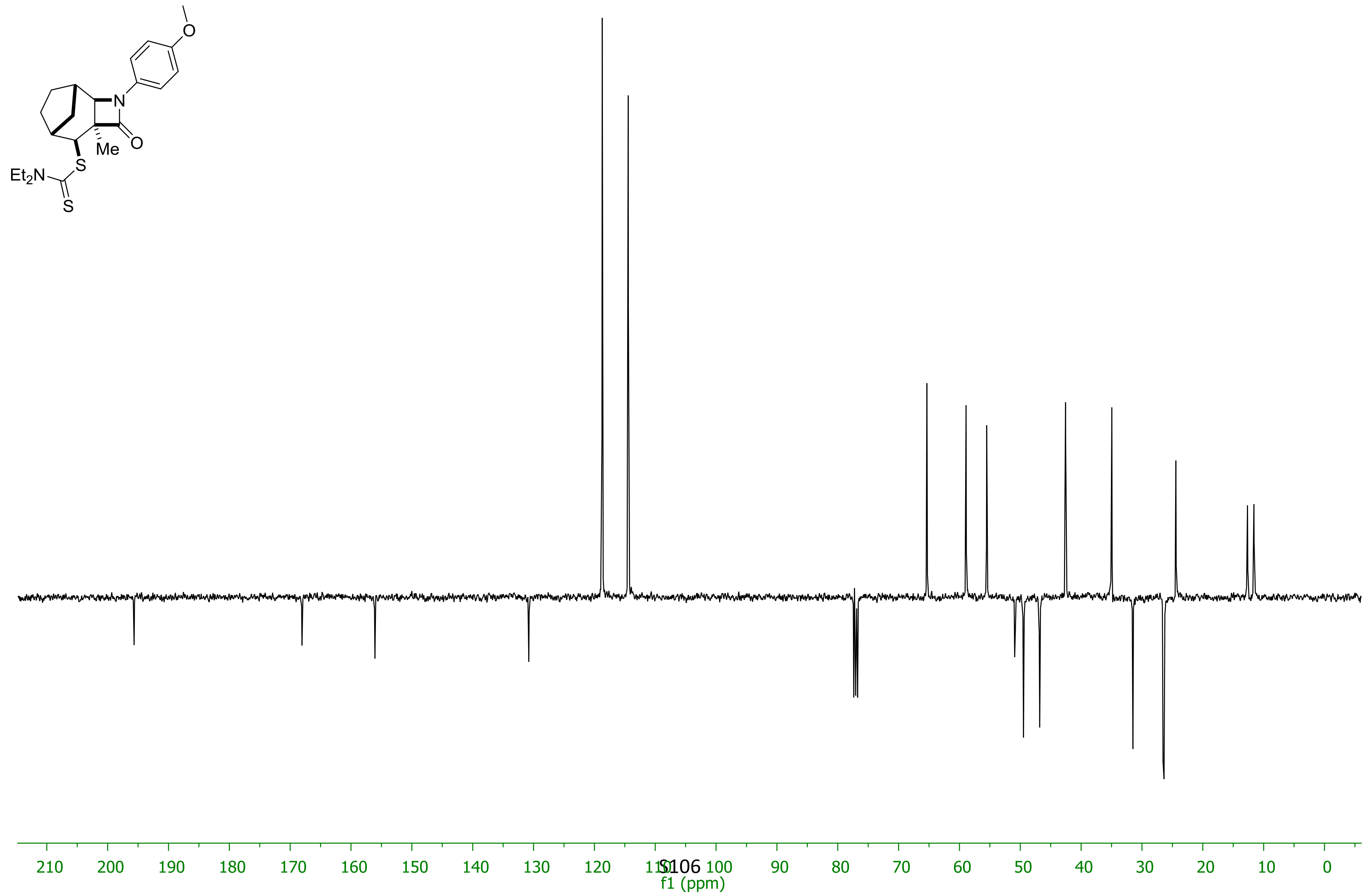

1,1-Diethyl-3-isopropylurea **66**; $CDCl_3$, 400 MHz

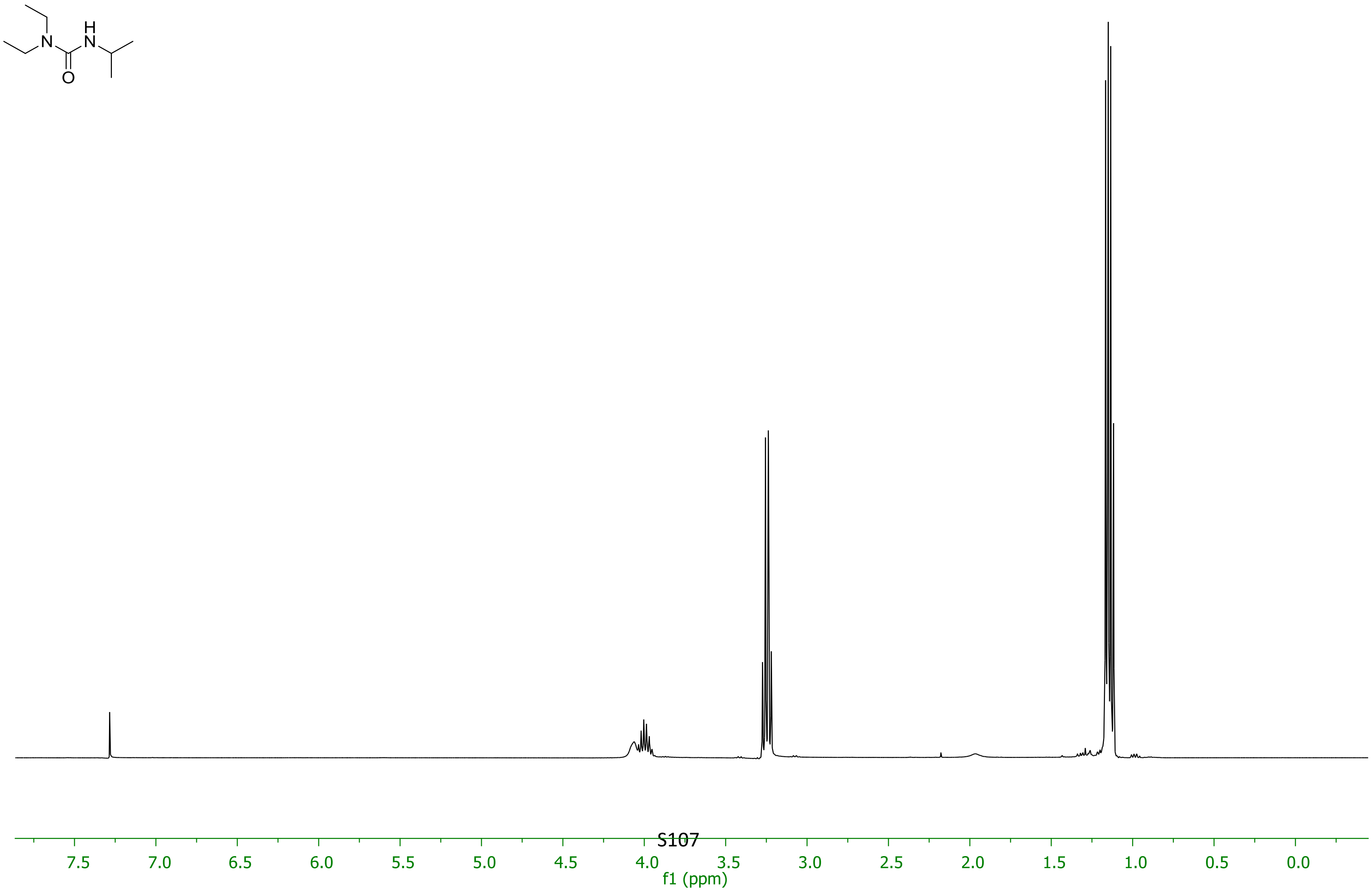

1,1-Diethyl-3-isopropylurea **66**; $CDCl_3$, 100 MHz

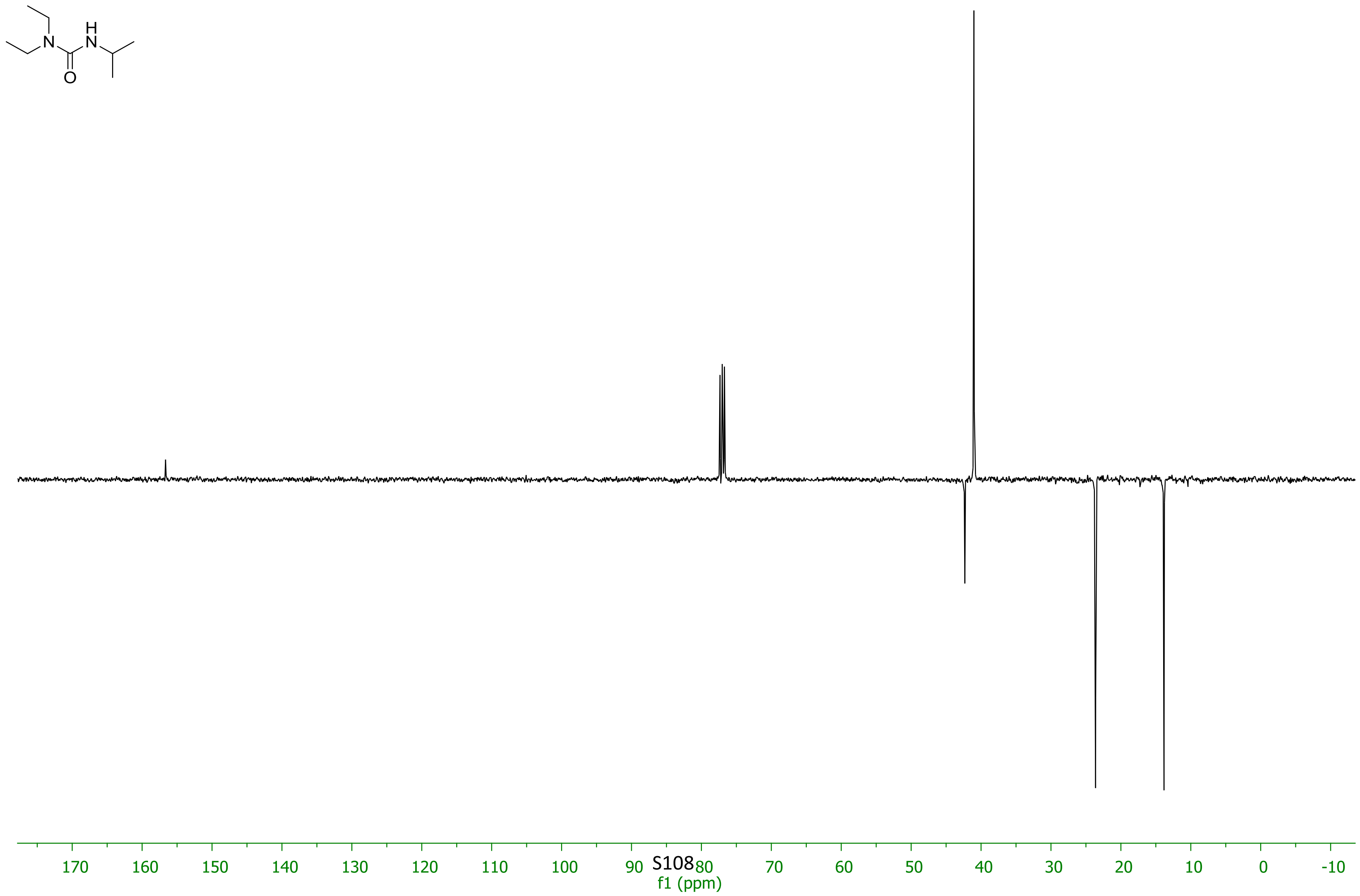

1-(4-Methoxyphenyl)-3-methyleneazetidin-2-one **67**; $CDCl_3$, 300 MHz

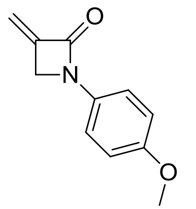

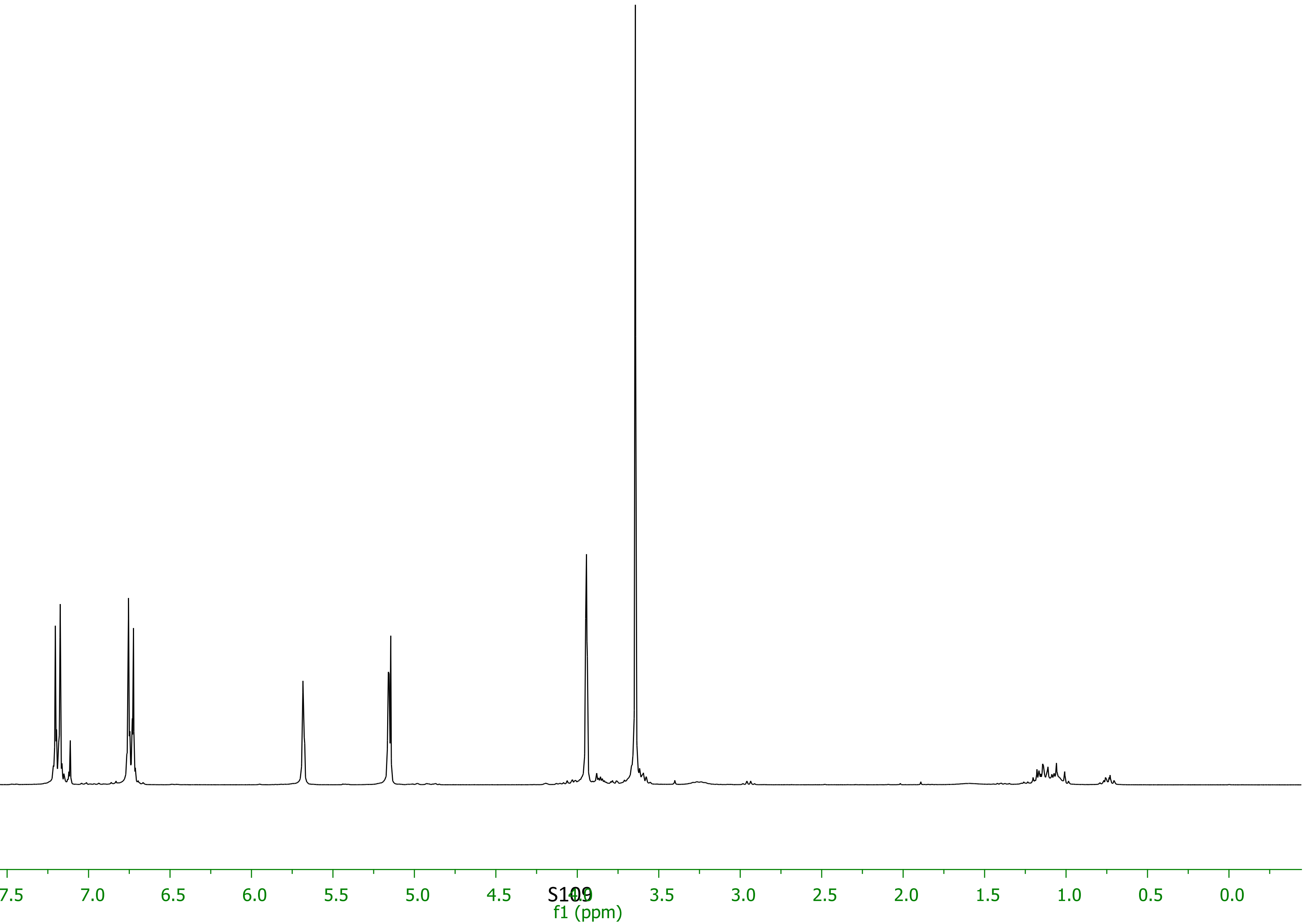

1-(4-Methoxyphenyl)-3-methyleneazetidin-2-one **67**; $CDCl_3$, 100 MHz

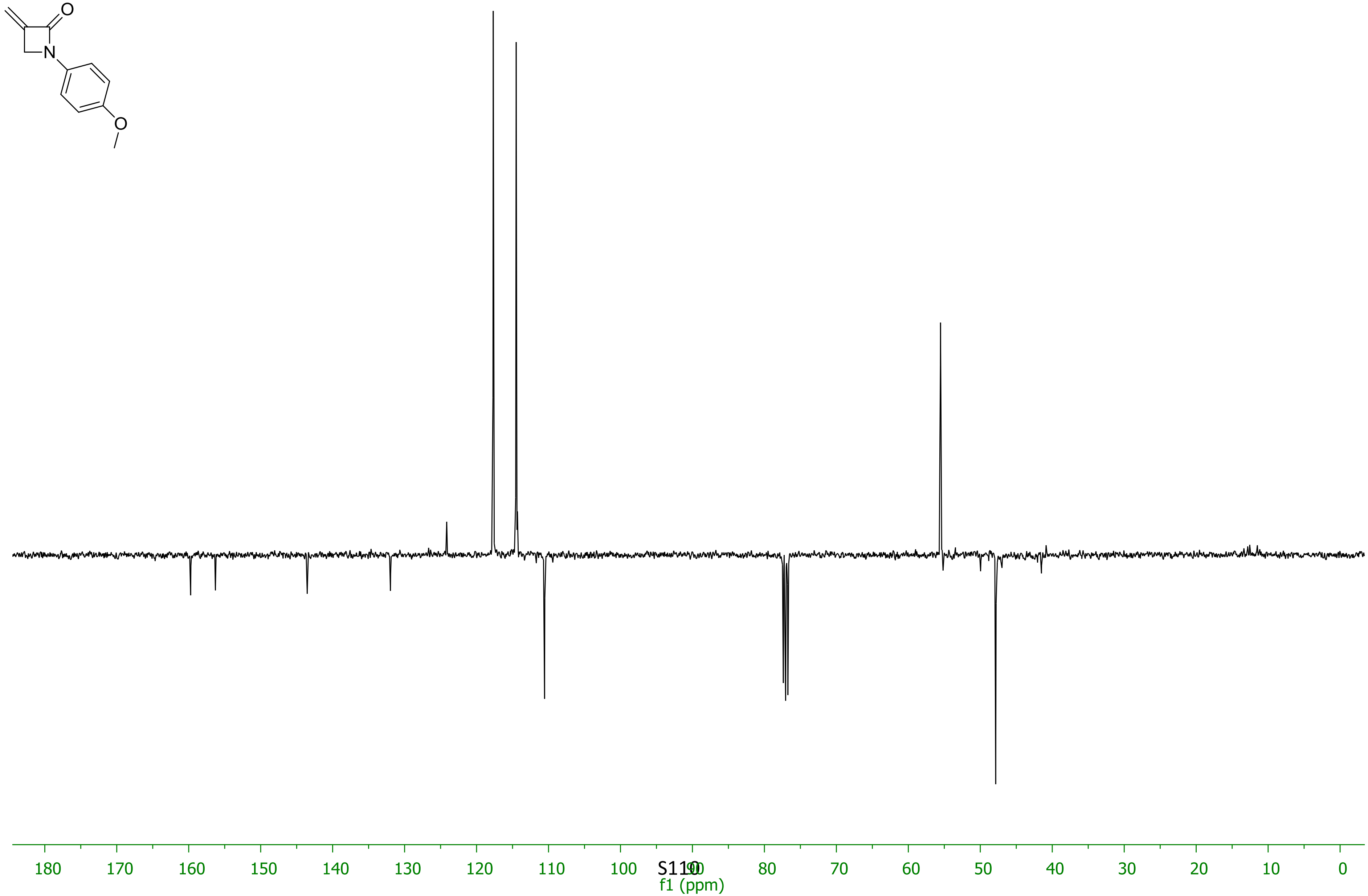

(±)-(3a*R*,5a*S*,8a*S*)-5-Benzylhexahydro-4*H*-[1,3]dioxolo[4',5':2,3]benzo[1,2-*b*]azete-2,4-dione **92**; $CDCl_3$, 400 MHz

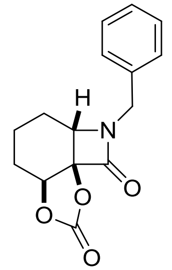

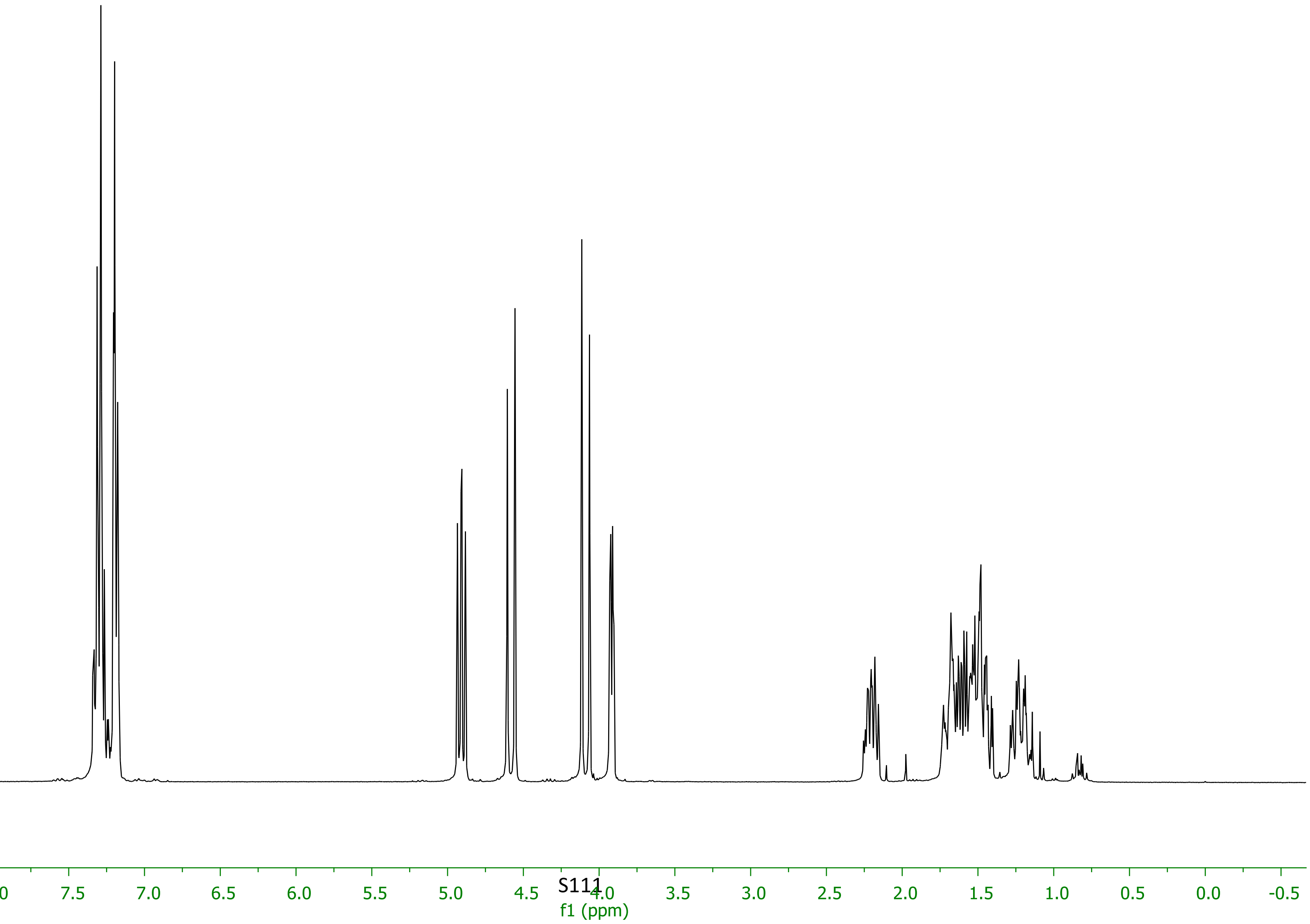

(±)-(3a*R*,5a*S*,8a*S*)-5-Benzylhexahydro-4*H*-[1,3]dioxolo[4',5':2,3]benzo[1,2-*b*]azete-2,4-dione **92**; $CDCl_3$, 100 MHz

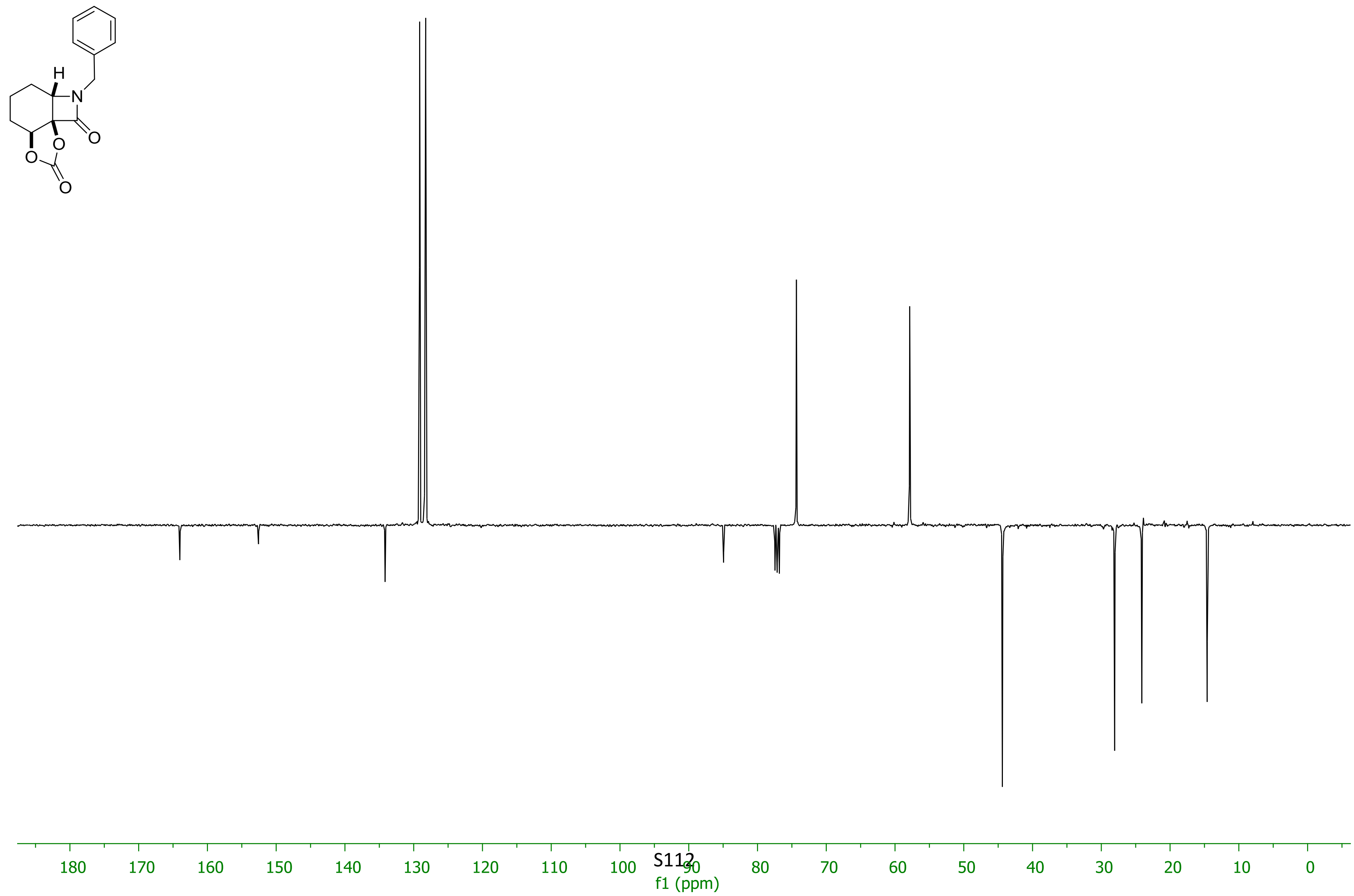

(±)-(1*S*,2*R*,4*S*,7*S*)-8-(4-Methoxyphenyl)-3-oxa-8-azatricyclo[5.2.0.0^{2,4}]nonan-9-one **70**; $CDCl_3$, 400 MHz

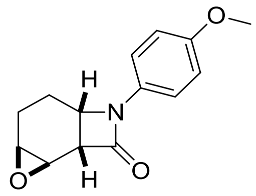

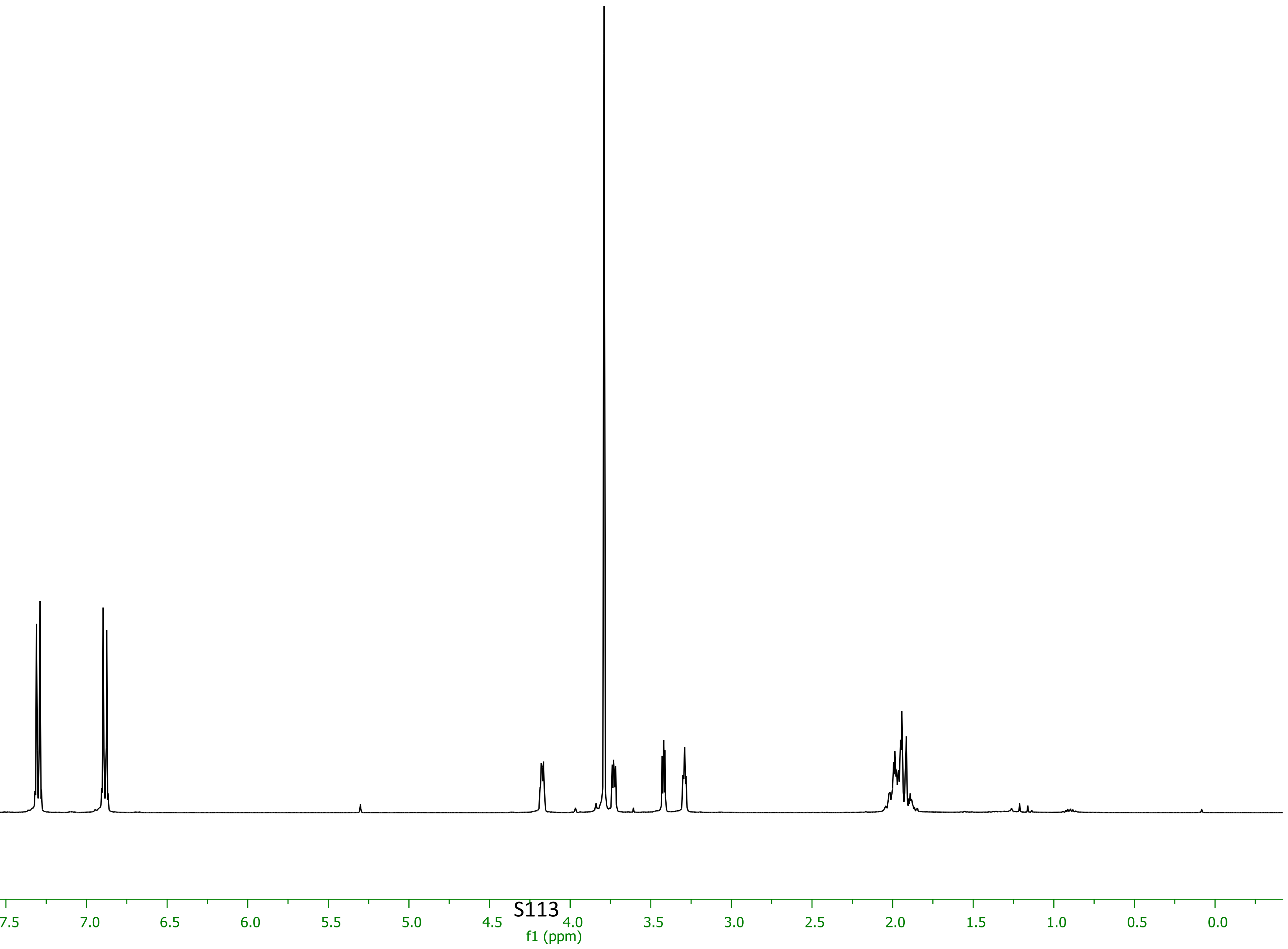

(±)-(1*S*,2*R*,4*S*,7*S*)-8-(4-Methoxyphenyl)-3-oxa-8-azatricyclo[5.2.0.0^{2,4}]nonan-9-one **70**; $CDCl_3$, 100 MHz

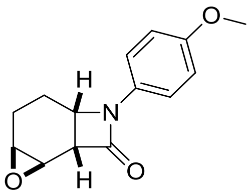

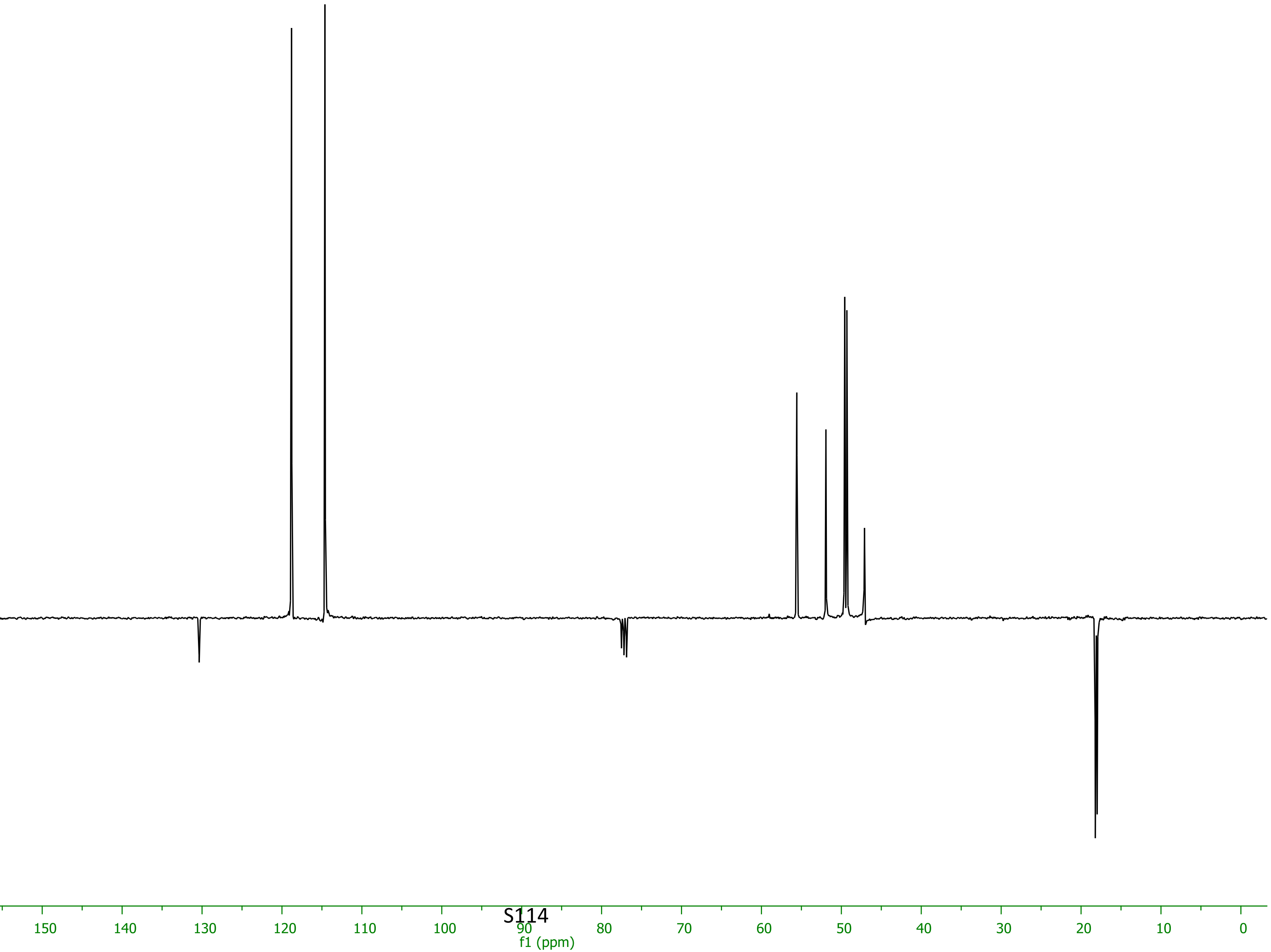

(±)-(3*S*,6*S*)-3-Hydroxy-7-(4-methoxyphenyl)-7-azabicyclo[4.2.0]oct-1-en-8-one **71**; $CDCl_3$, 300 MHz

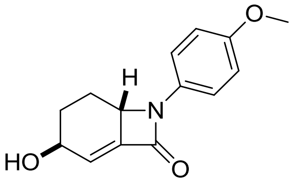

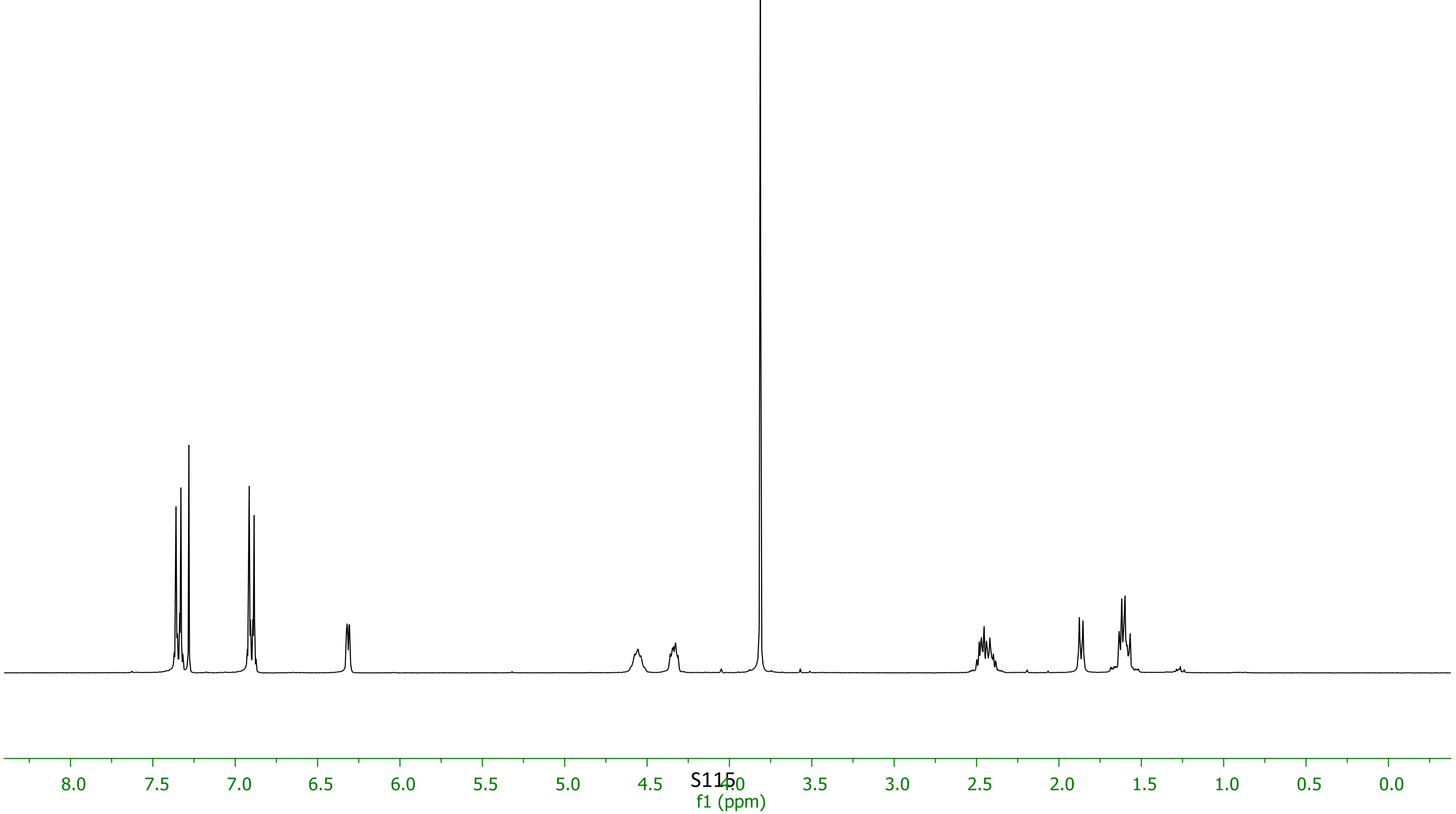

(±)-(3*S*,6*S*)-3-Hydroxy-7-(4-methoxyphenyl)-7-azabicyclo[4.2.0]oct-1-en-8-one **71**; $CDCl_3$, 100 MHz

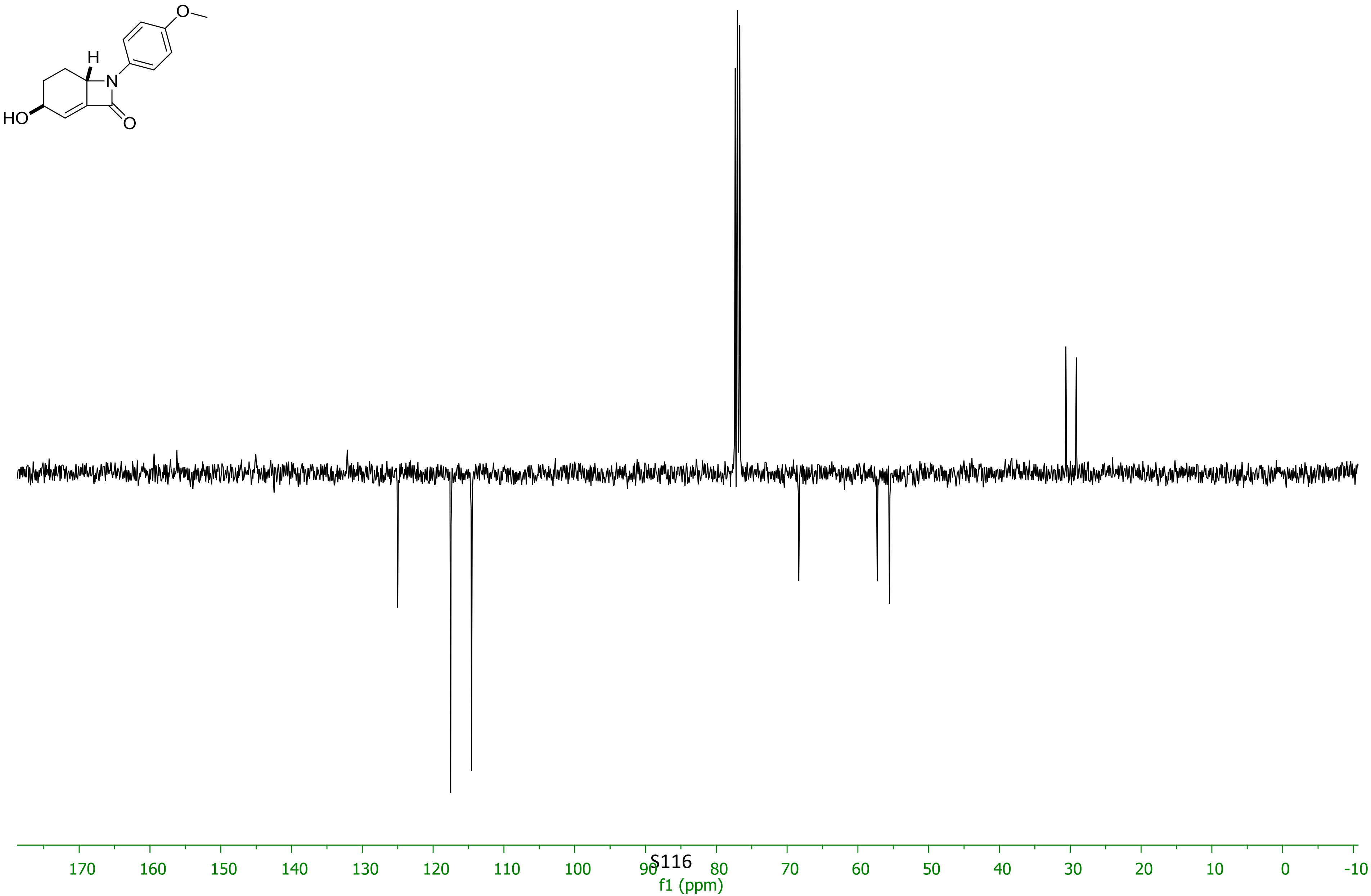

(±)-(3*S*,6*S*)-7-(4-Methoxyphenyl)-8-oxo-7-azabicyclo[4.2.0]oct-1-en-3-yl benzoate **72**; $CDCl_3$, 400 MHz

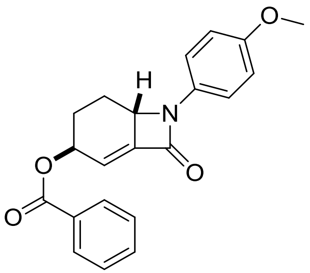

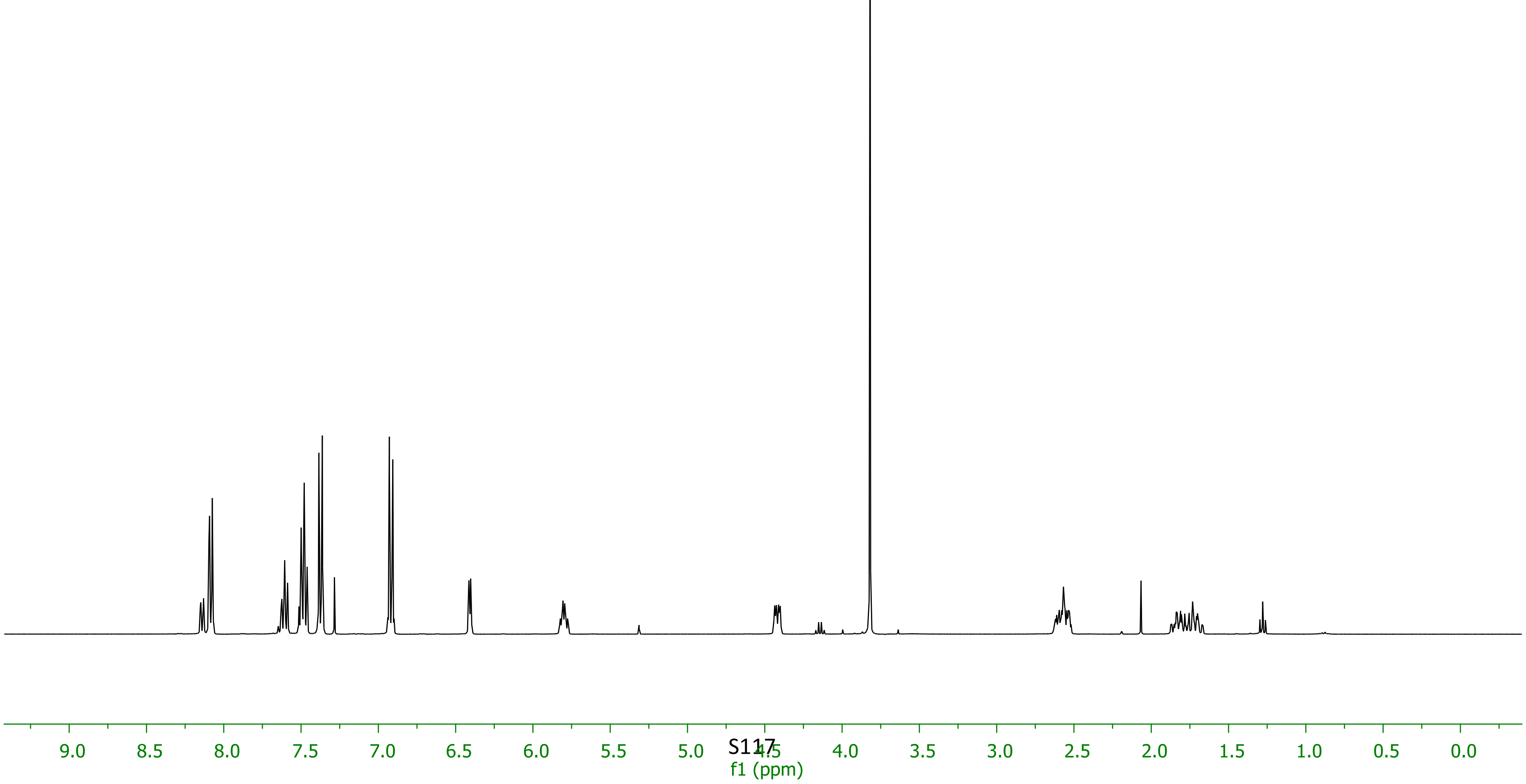

(±)-(3*S*,6*S*)-7-(4-Methoxyphenyl)-8-oxo-7-azabicyclo[4.2.0]oct-1-en-3-yl benzoate **72**; $CDCl_3$, 100 MHz

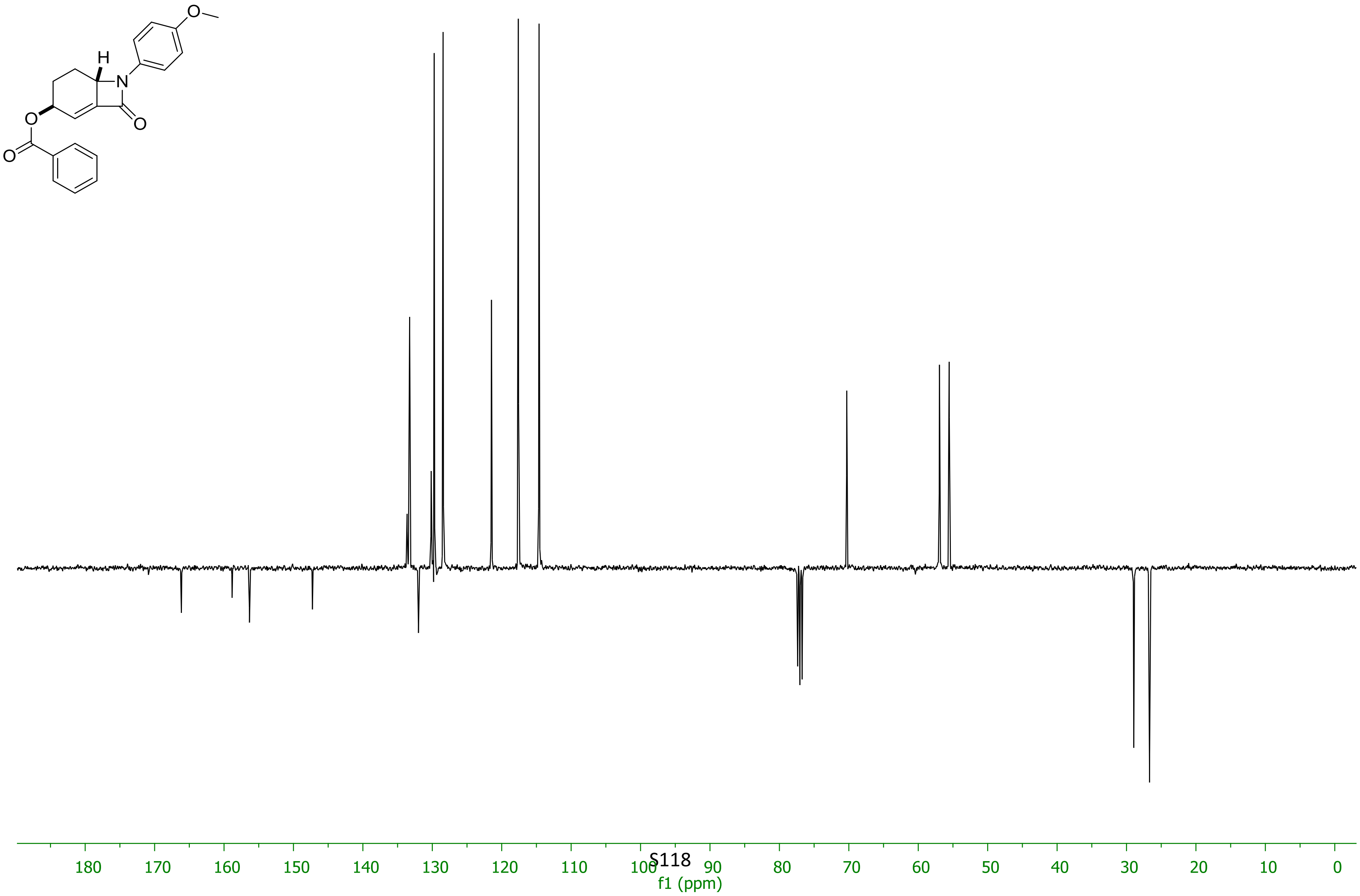

(±)-(1*R*,2*S*,3*S*,6*S*)-1,2-Dihydroxy-7-(4-methoxyphenyl)-8-oxo-7-azabicyclo[4.2.0]octan-3-yl benzoate **74**; $CDCl_3$, 400 MHz

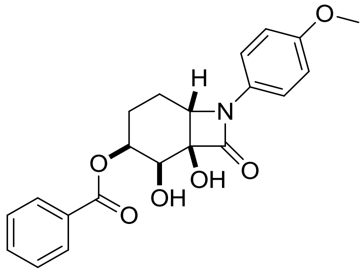

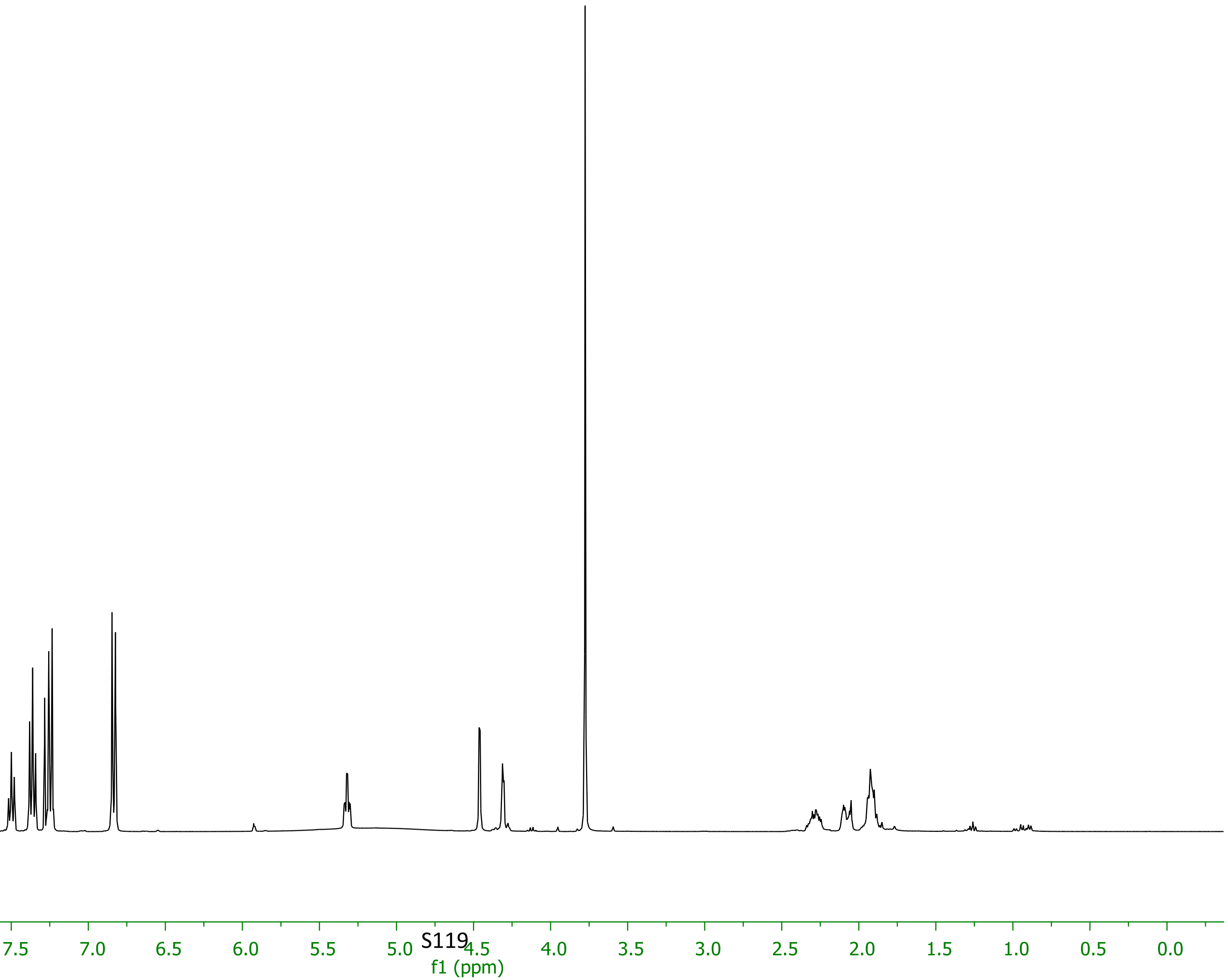

(±)-(1*R*,2*S*,3*S*,6*S*)-1,2-Dihydroxy-7-(4-methoxyphenyl)-8-oxo-7-azabicyclo[4.2.0]octan-3-yl benzoate **74**; $CDCl_3$, 100 MHz

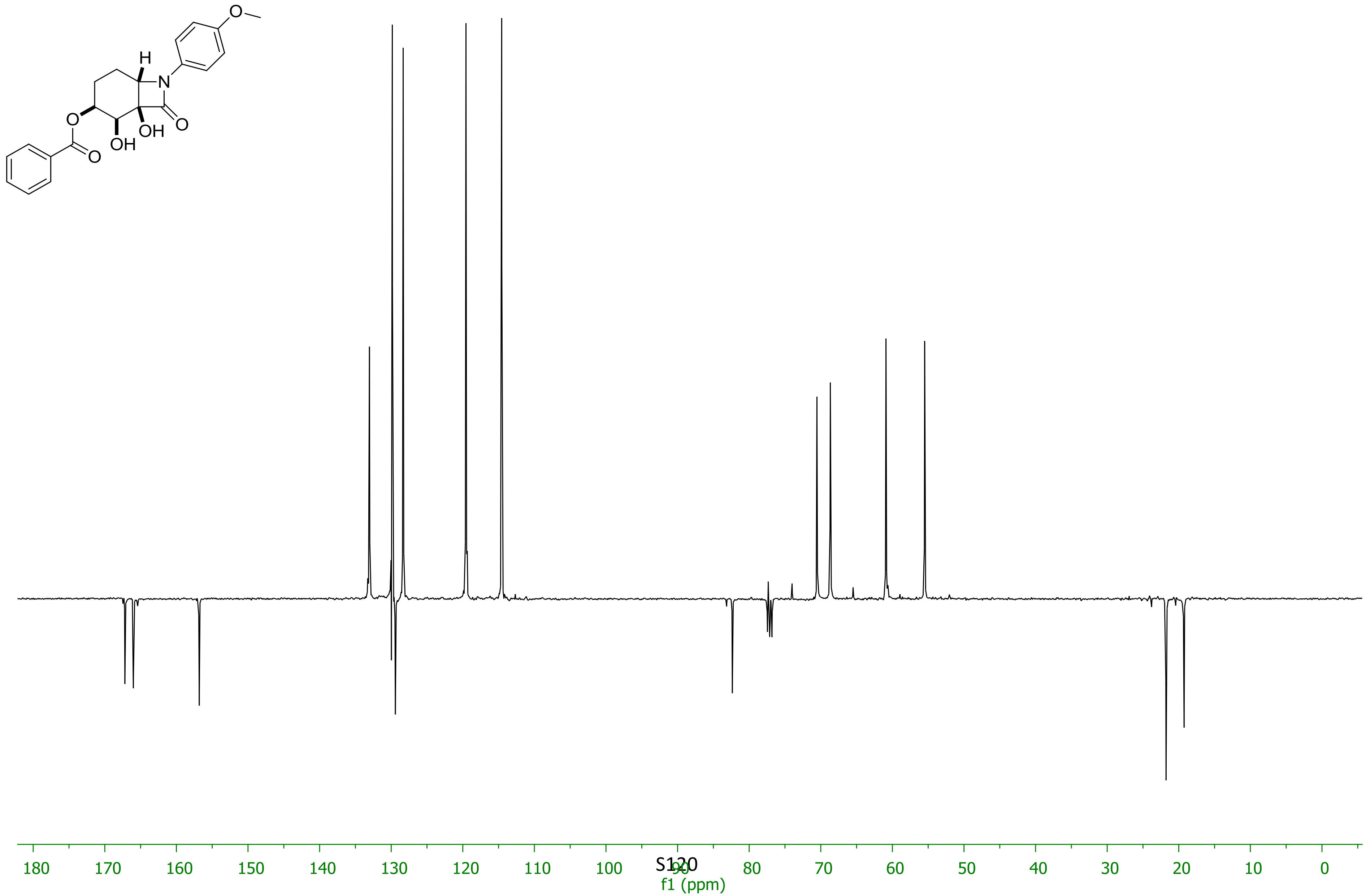

(±)-(3a*R*,5a*S*,8*S*,8a*S*)-5-(4-Methoxyphenyl)-2-oxido-4-oxohexahydro-4*H*-[1,3,2] dioxathiolo[4',5':2,3] benzo[1,2-*b*]azet-8-yl benzoate **102a**; $CDCl_3$, 400 MHz

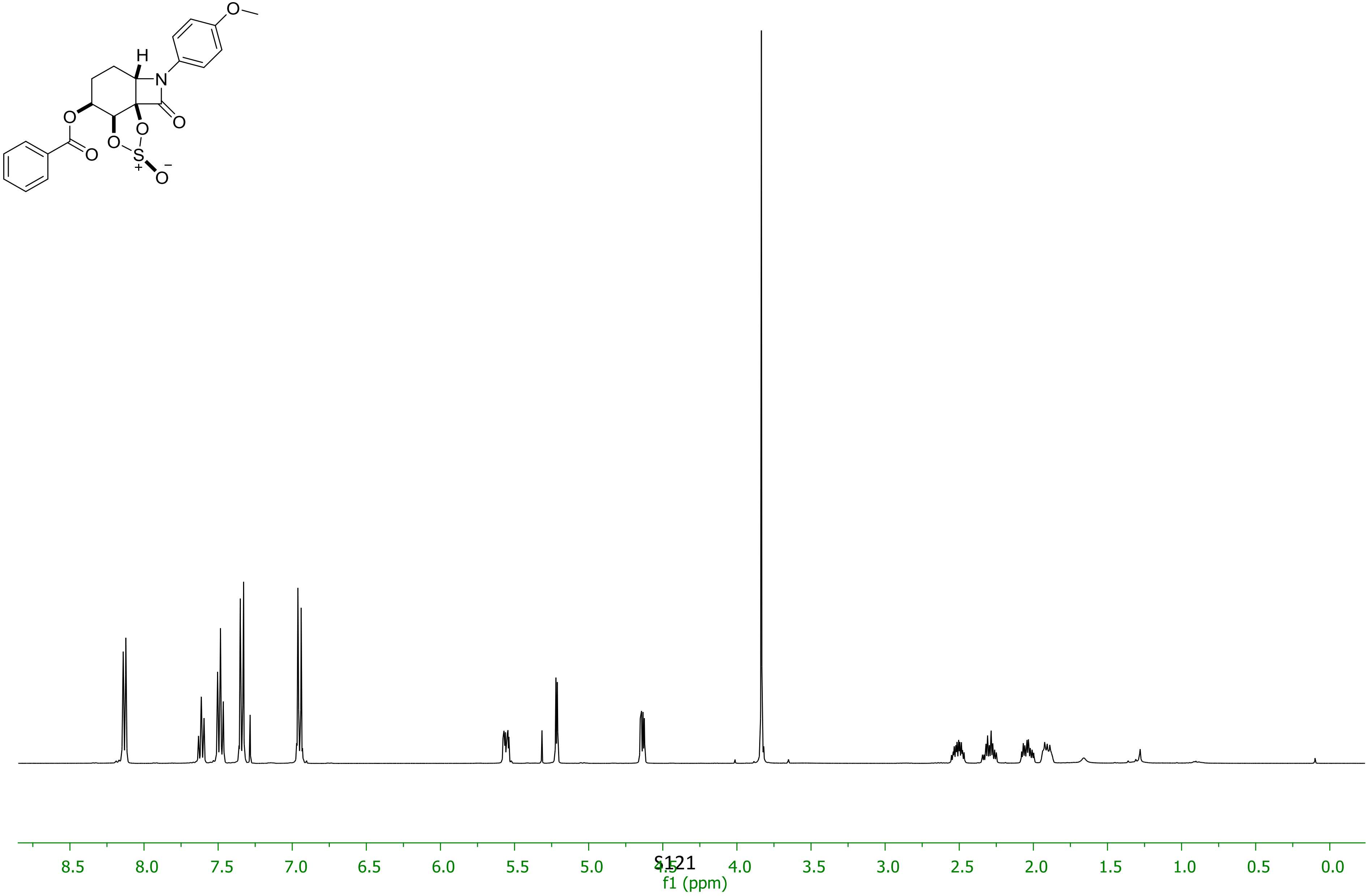

(±)-(3a*R*,5a*S*,8*S*,8a*S*)-5-(4-Methoxyphenyl)-2-oxido-4-oxohexahydro-4*H*-[1,3,2] dioxathiolo[4',5':2,3] benzo[1,2-*b*]azet-8-yl benzoate **102a**; $CDCl_3$, 100 MHz

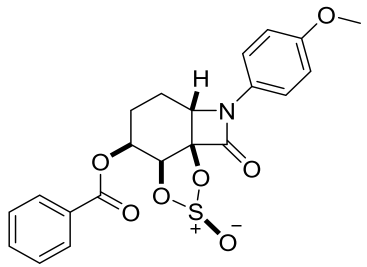

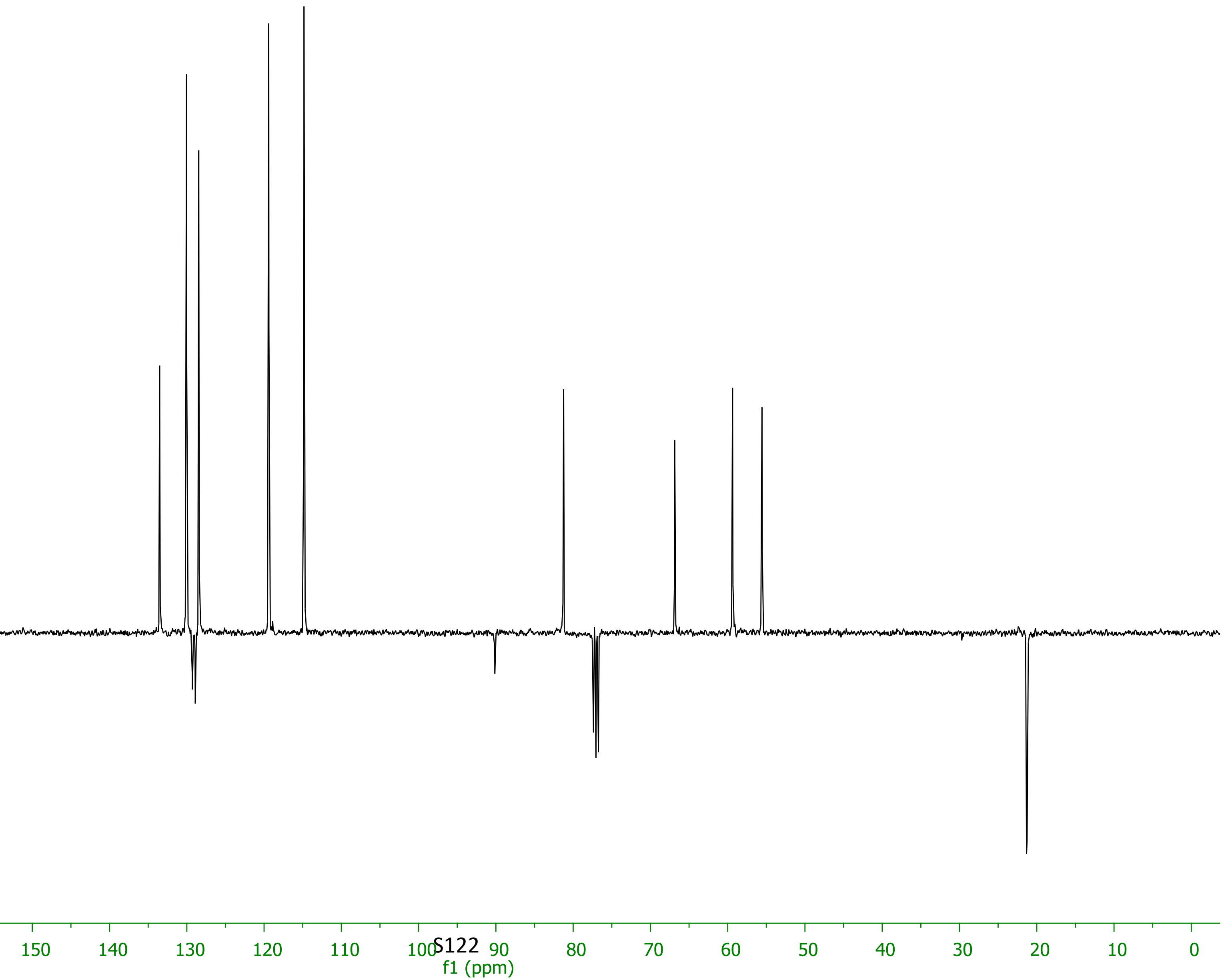

(±)-(3a*R*,5a*S*,8*S*,8a*S*)-5-(4-Methoxyphenyl)-2-oxido-4-oxohexahydro-4*H*-[1,3,2] dioxathiolo[4',5':2,3] benzo[1,2-*b*]azet-8-yl benzoate **102b**; $CDCl_3$, 400 MHz

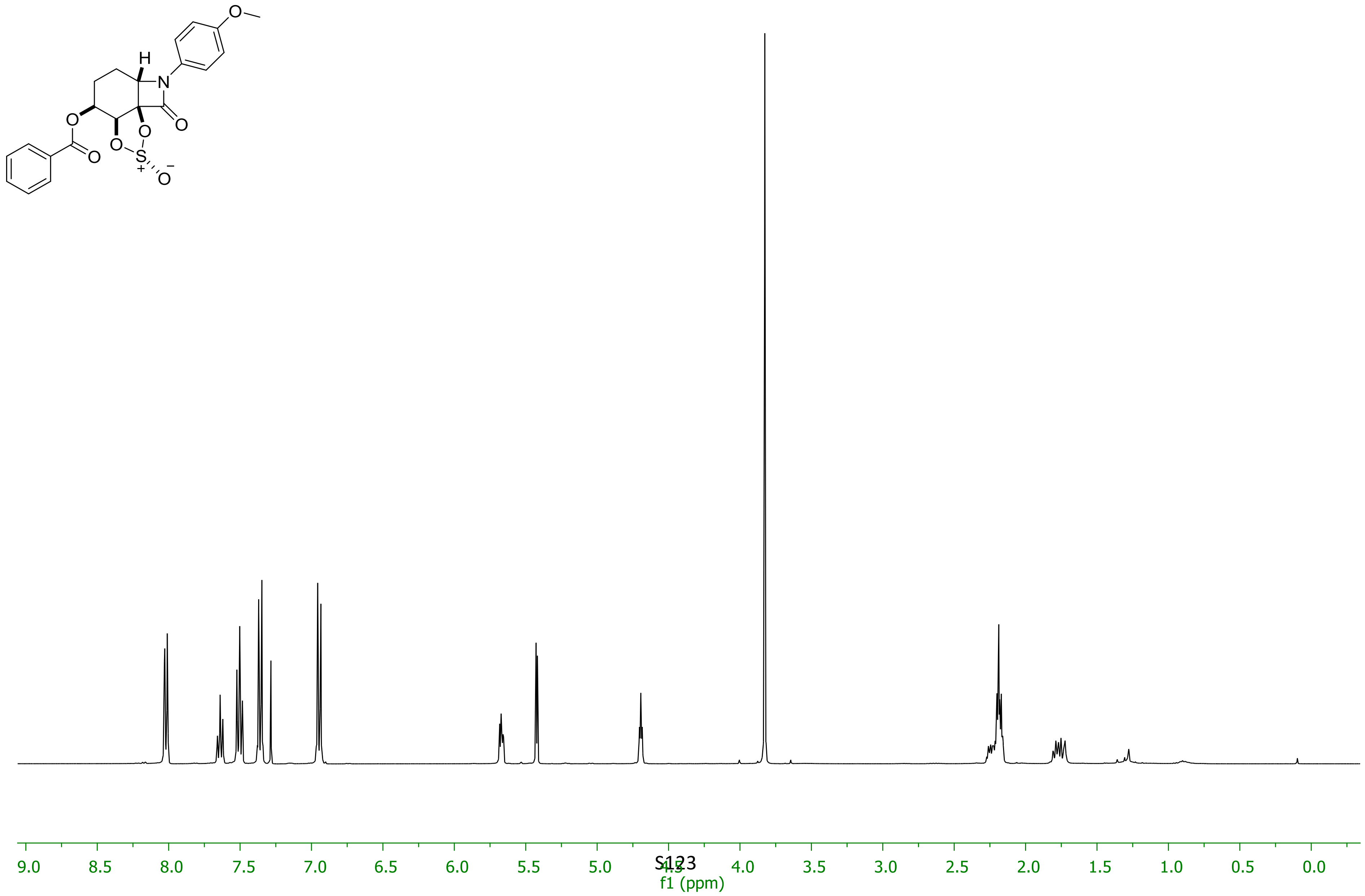

(±)-(3a*R*,5a*S*,8*S*,8a*S*)-5-(4-Methoxyphenyl)-2-oxido-4-oxohexahydro-4*H*-[1,3,2] dioxathiolo[4',5':2,3] benzo[1,2-*b*]azet-8-yl benzoate **102b**; $CDCl_3$, 100 MHz

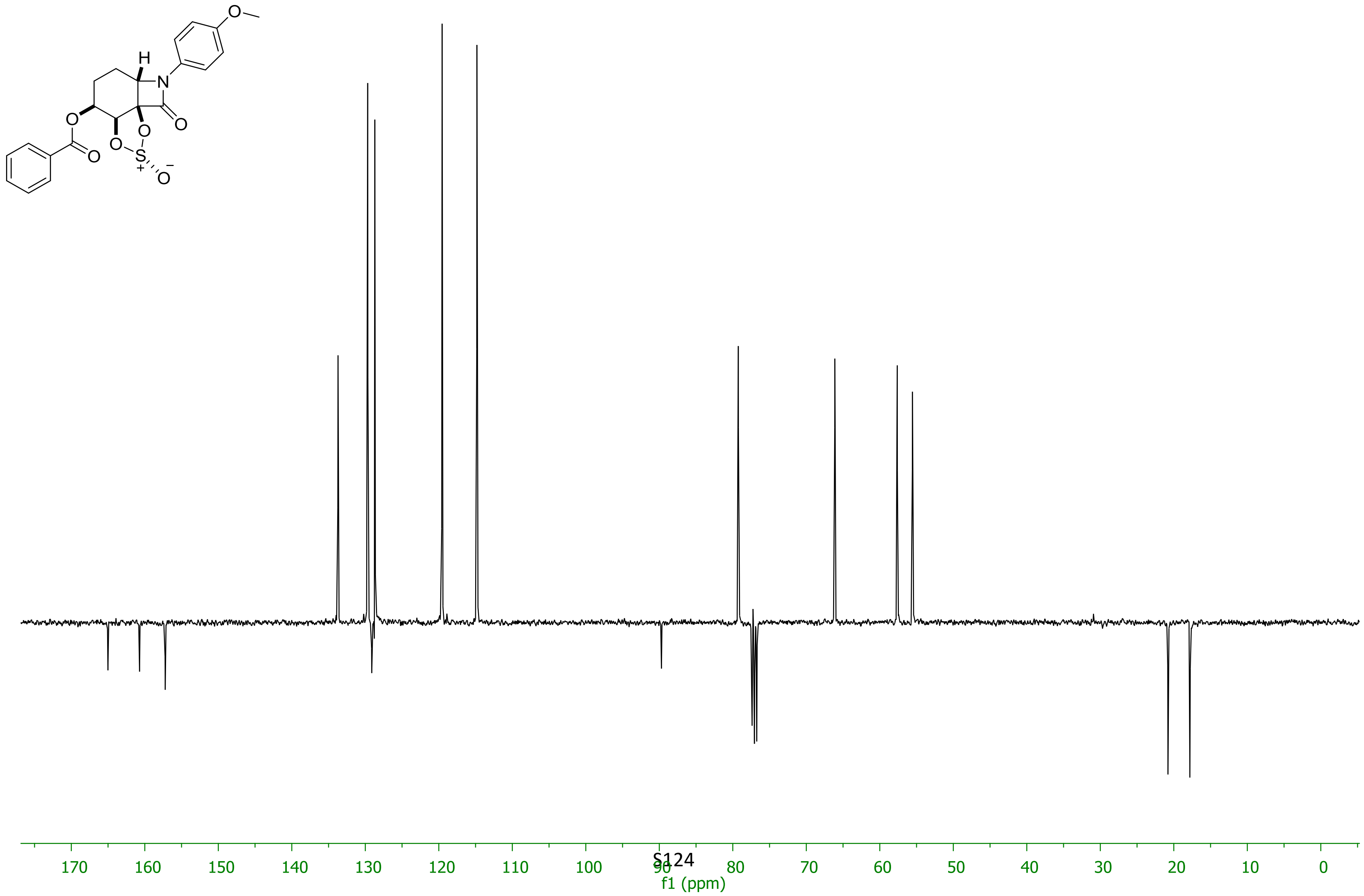

(±)-(1*R*,2*S*,5*S*)-6-(4-Methoxyphenyl)-7,8-dioxo-6-azabicyclo[3.2.1]octan-2-yl benzoate **80**; $CDCl_3$, 400 MHz

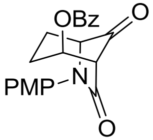

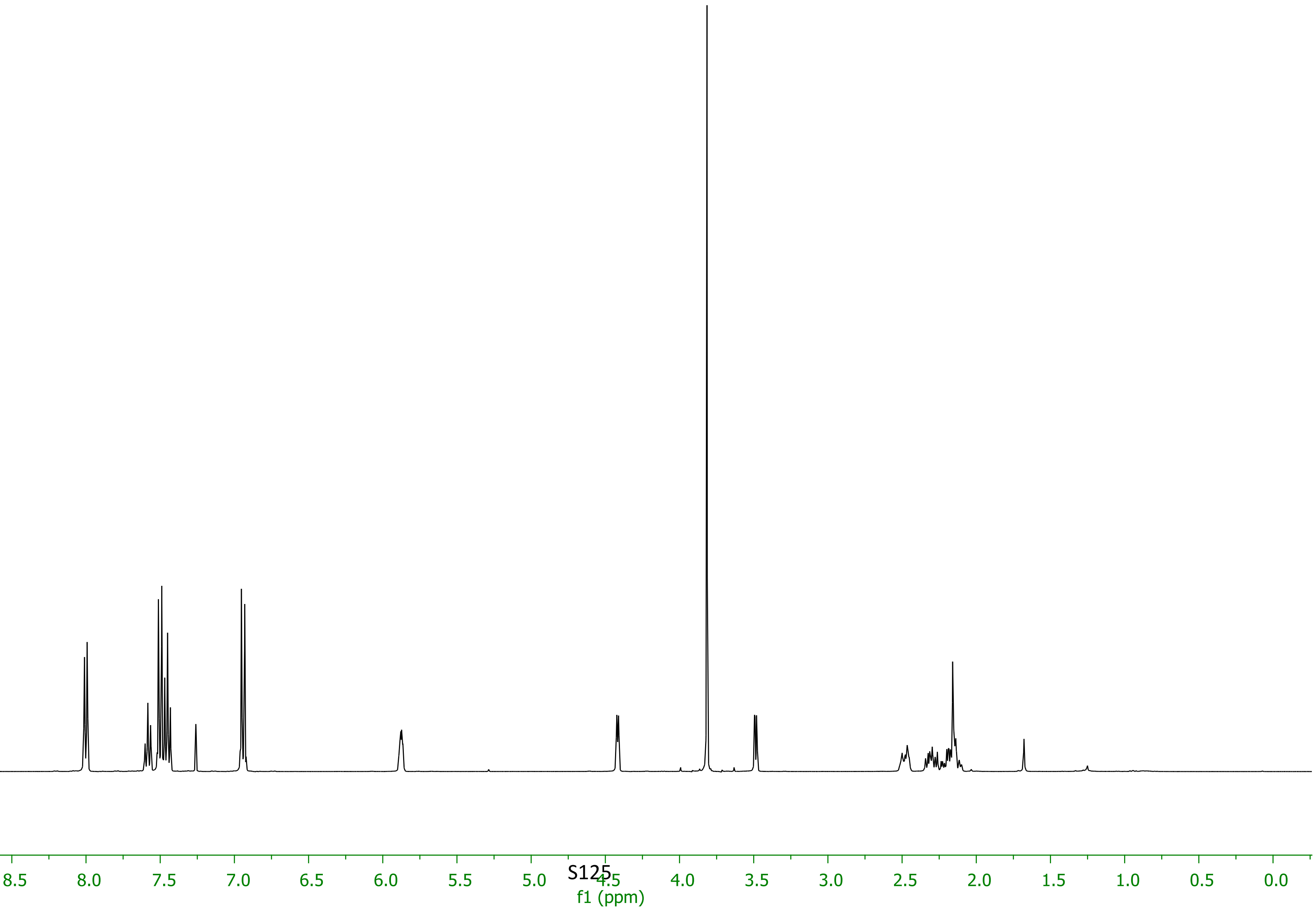

(±)-(1*R*,2*S*,5*S*)-6-(4-Methoxyphenyl)-7,8-dioxo-6-azabicyclo[3.2.1]octan-2-yl benzoate **80**; $CDCl_3$, 100 MHz

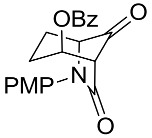

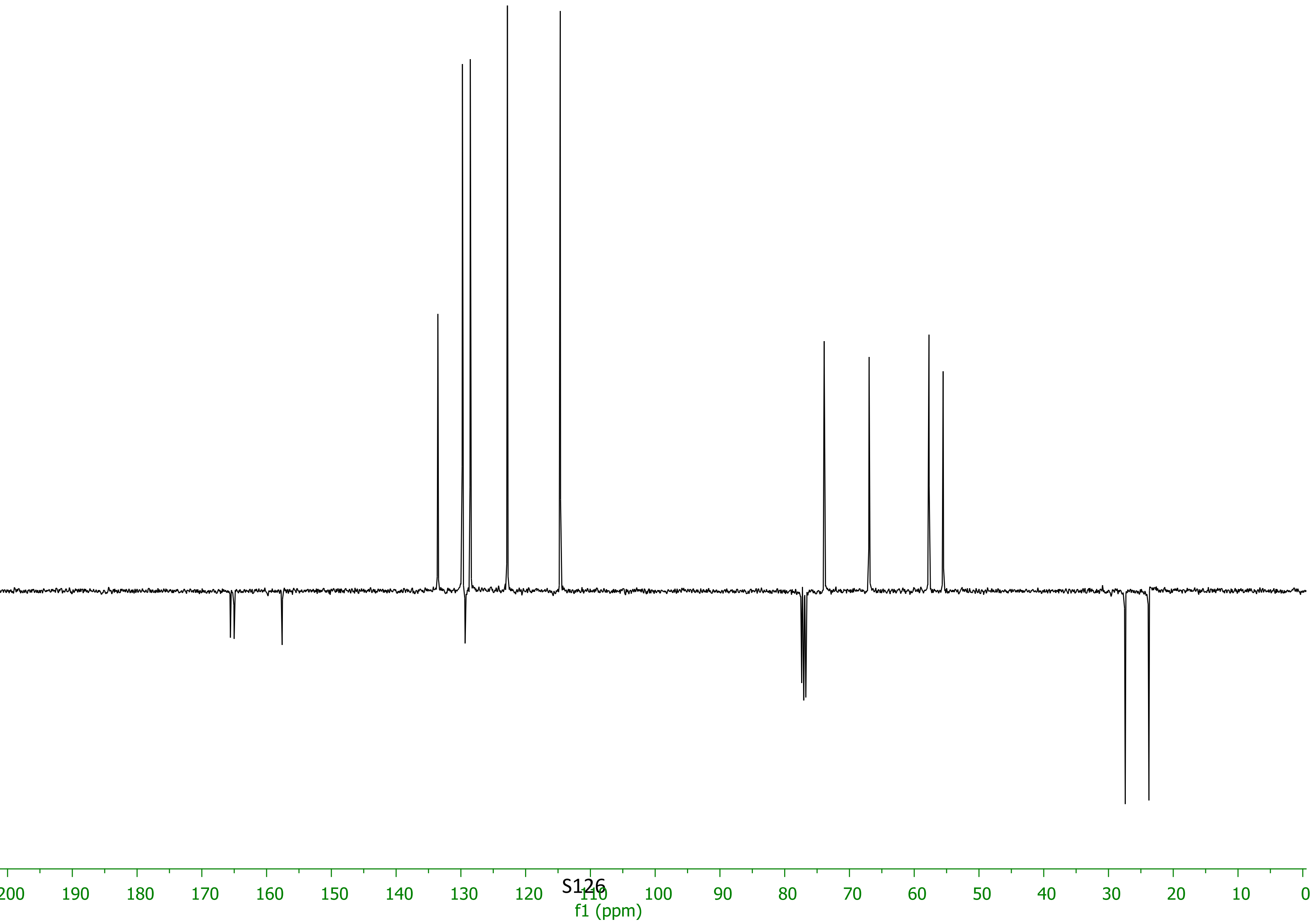

(±)-(1*S*,2*R*,5*S*,6*R*,7*R*)-5,6-Dihydroxy-3-(4-methoxyphenyl)-3-azatricyclo[5.2.1.0^{2,5}] decan-4-one **76**; $CDCl_3$, 400 MHz

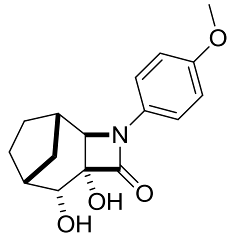

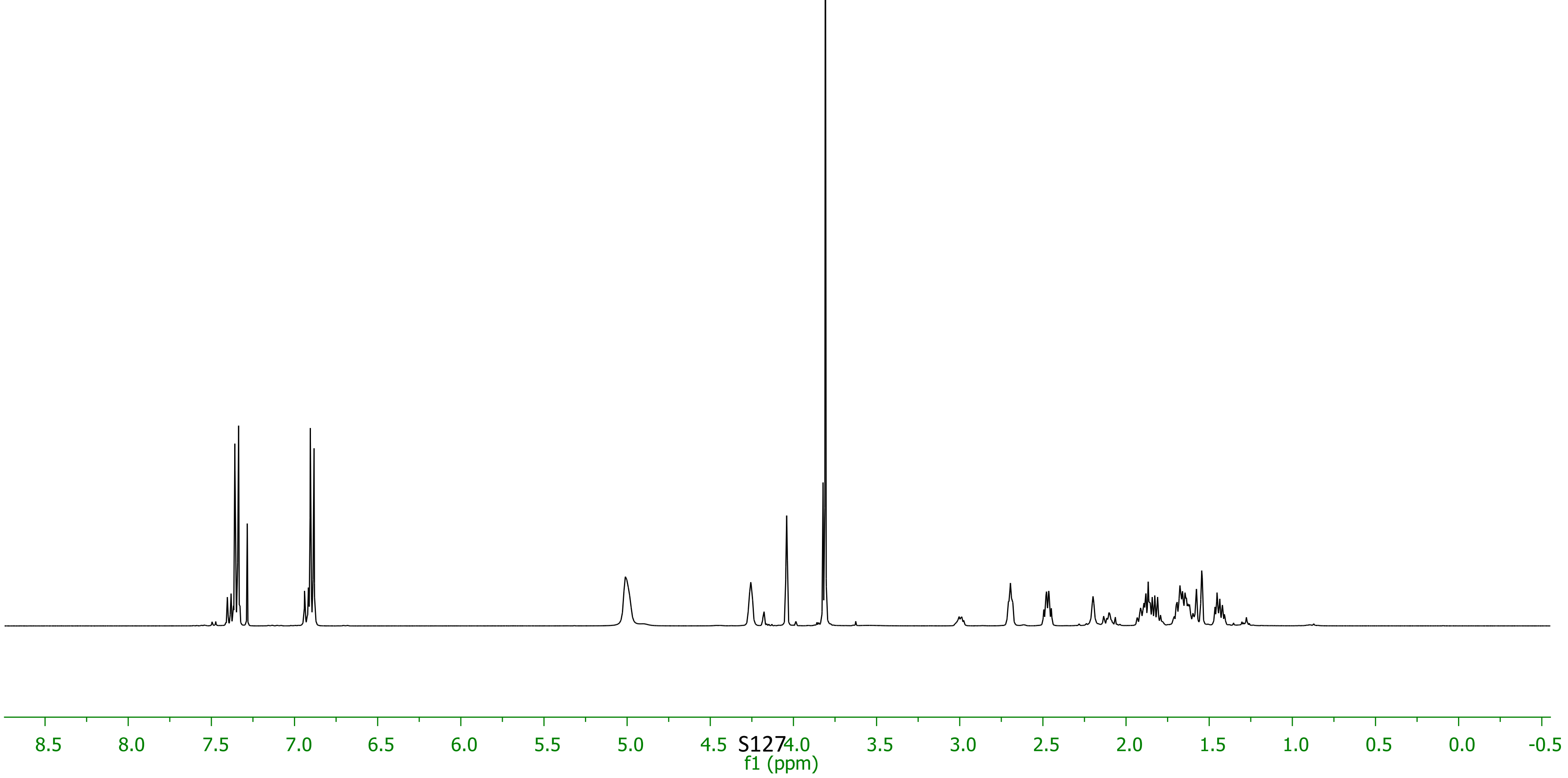

(±)-(1*S*,2*R*,5*S*,6*R*,7*R*)-5,6-Dihydroxy-3-(4-methoxyphenyl)-3-azatricyclo[5.2.1.$0^{2,5}$] decan-4-one **76**; $CDCl_3$, 100 MHz

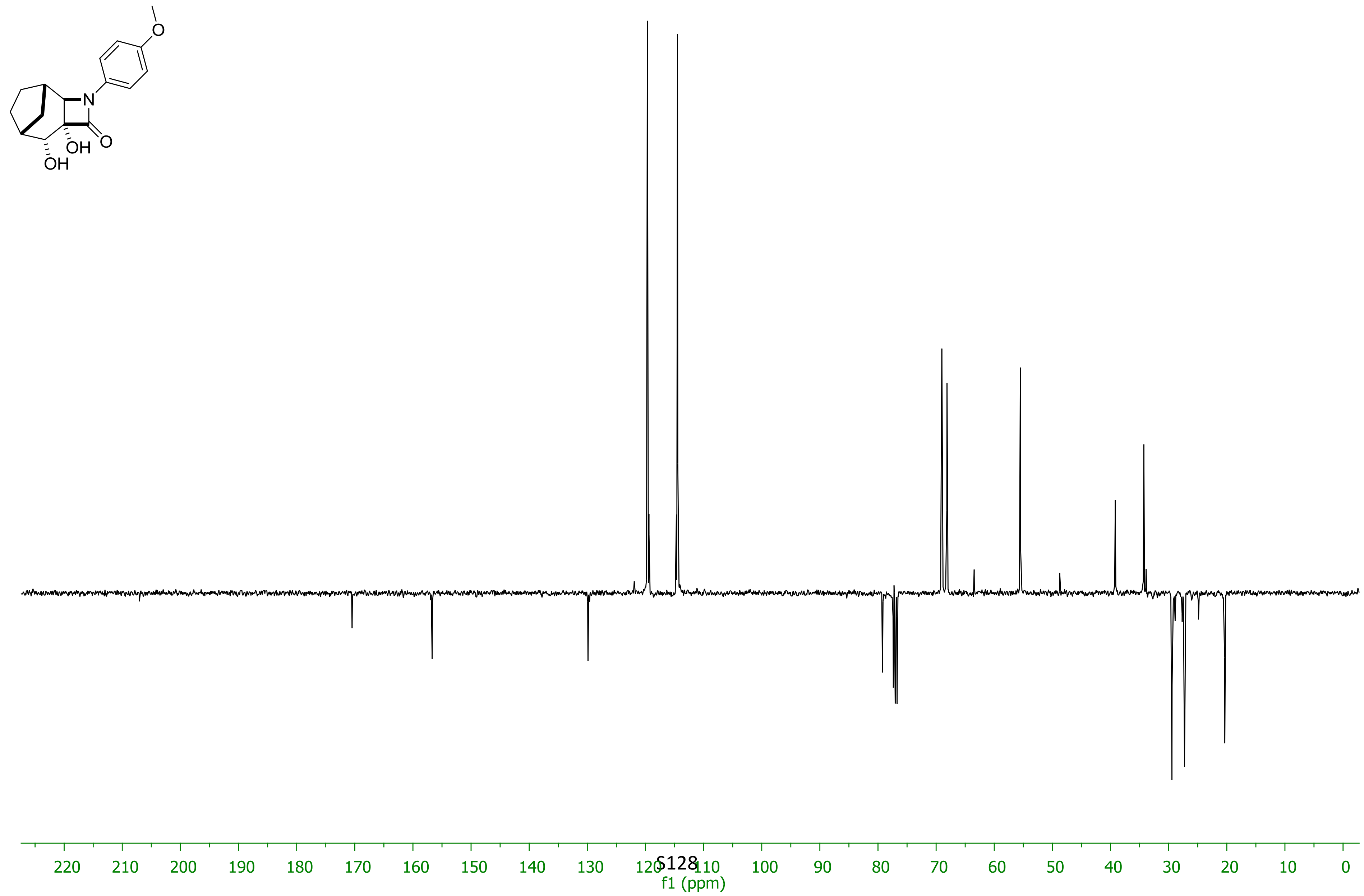

(±)-(1*S*,2*R*,5*S*,7*R*)-5-Hydroxy-3-(4-methoxyphenyl)-3-azatricyclo[5.2.1.0^{2,5}] decane-4,6-dione **77**; $CDCl_3$, 400 MHz

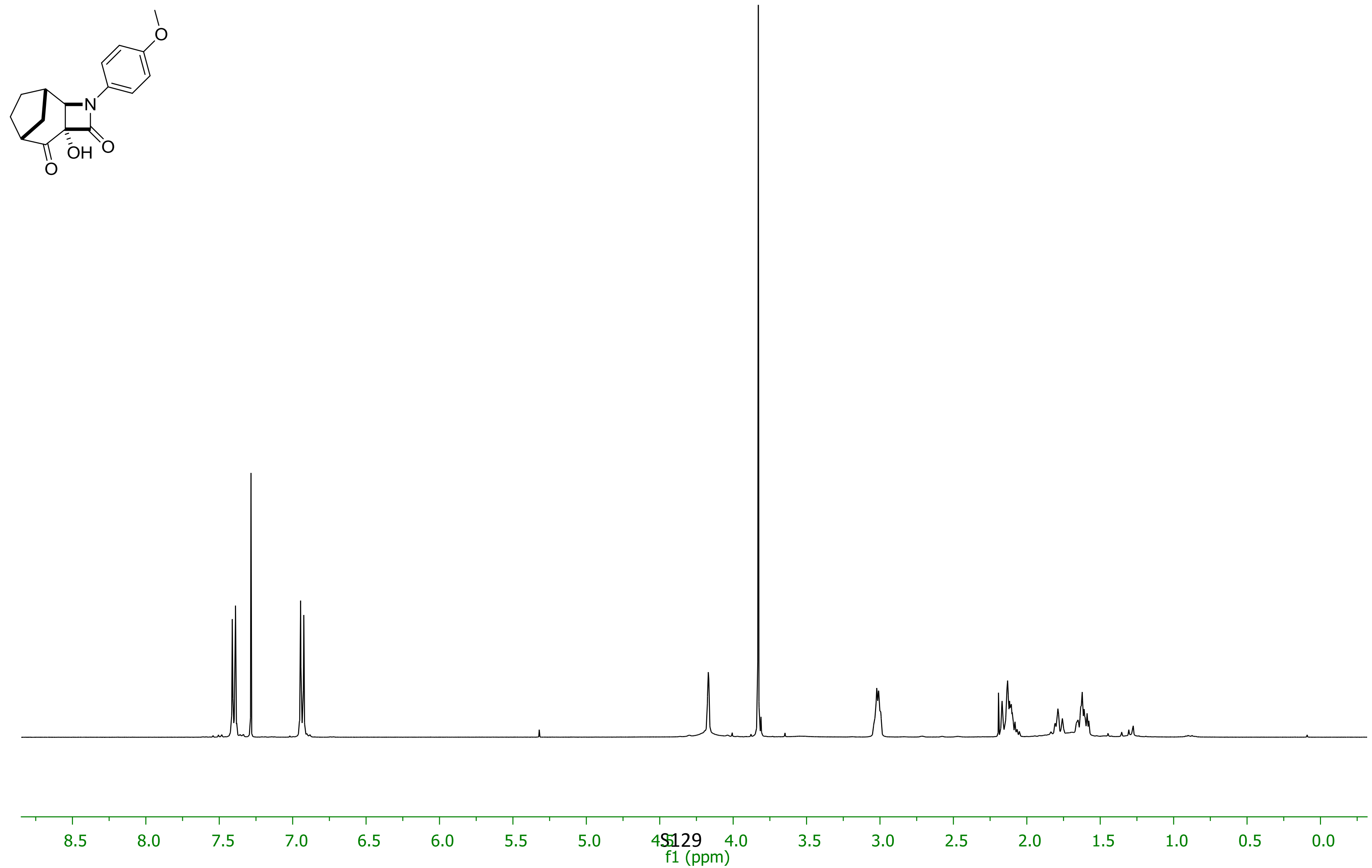

(±)-(1*S*,2*R*,5*S*,7*R*)-5-Hydroxy-3-(4-methoxyphenyl)-3-azatricyclo[5.2.1.0^{2,5}] decane-4,6-dione **77**; $CDCl_3$, 100 MHz

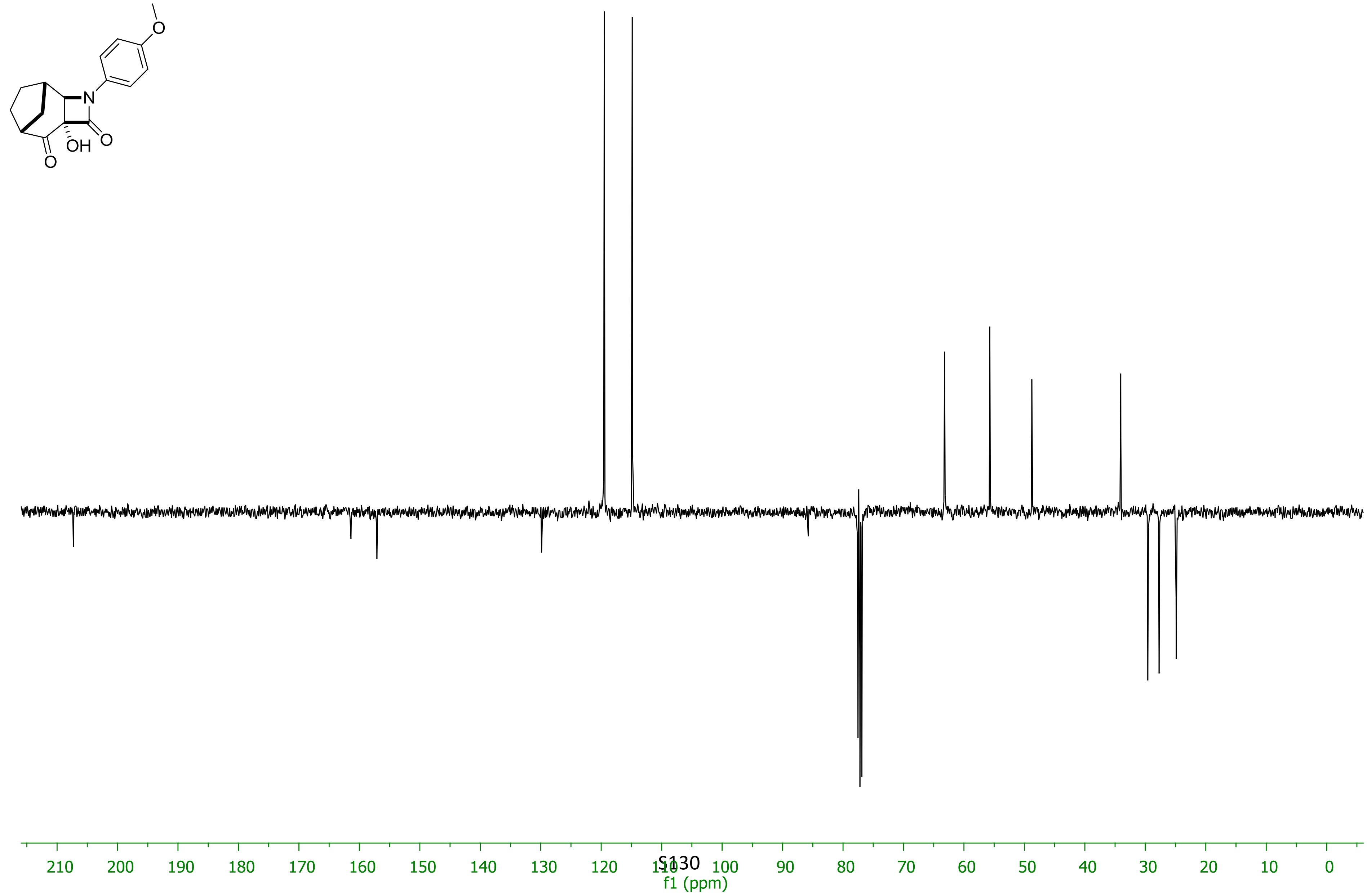

(±)-(3a*S*,5a*R*,6*S*,9*R*,9a*S*)-5-(4-Methoxyphenyl)hexahydro-6,9-methanol[1,3,2] dioxathiolo[4',5':2,3] cyclohepta[1,2-*b*]azet-4(5H)-one 2-oxide **103a**; $CDCl_3$, 400 MHz

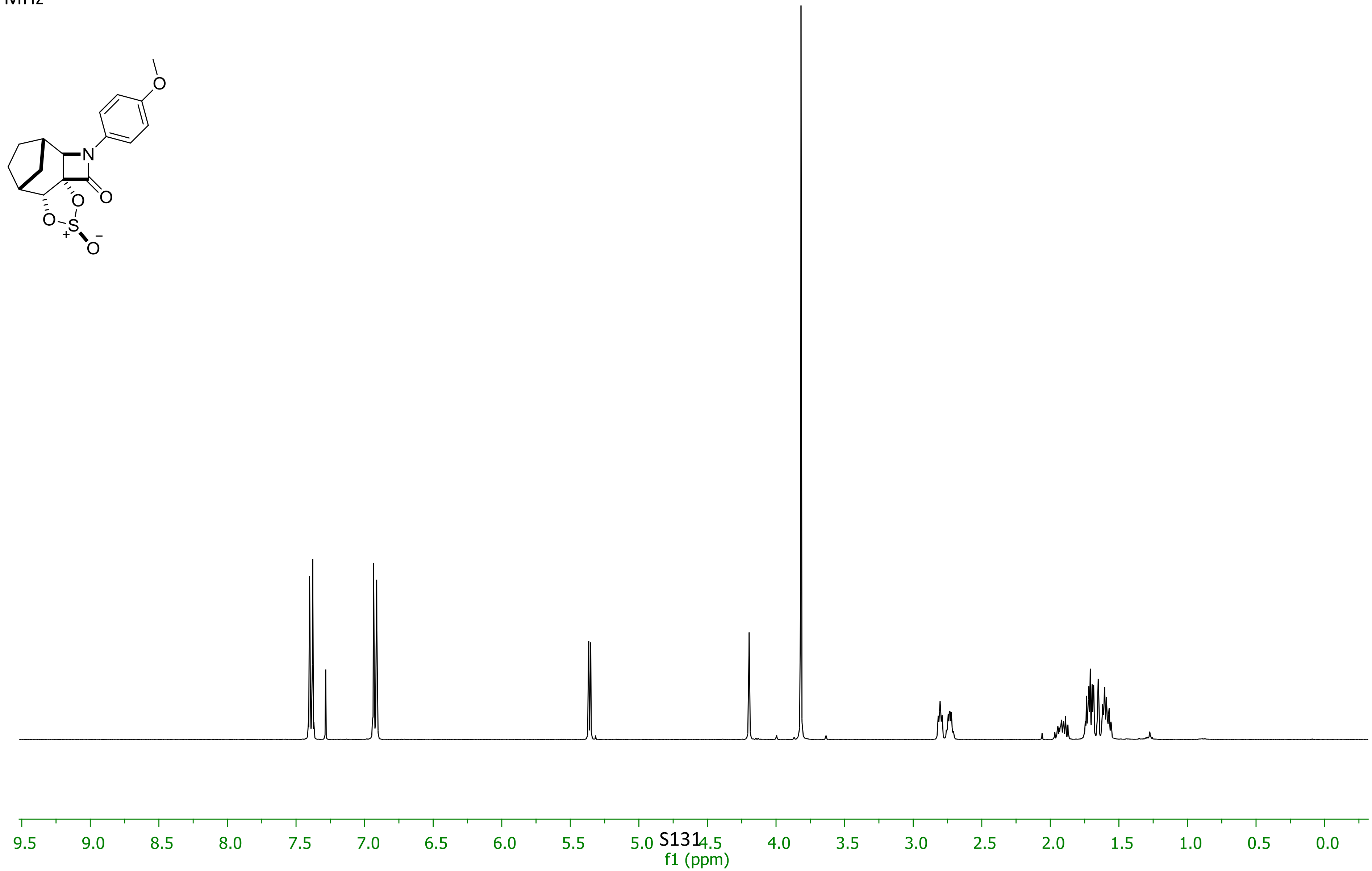

(±)-(3a*S*,5a*R*,6*S*,9*R*,9a*S*)-5-(4-Methoxyphenyl)hexahydro-6,9-methanol[1,3,2] dioxathiolo[4',5':2,3] cyclohepta[1,2-*b*]azet-4(5H)-one 2-oxide **103a**; $CDCl_3$, 100 MHz

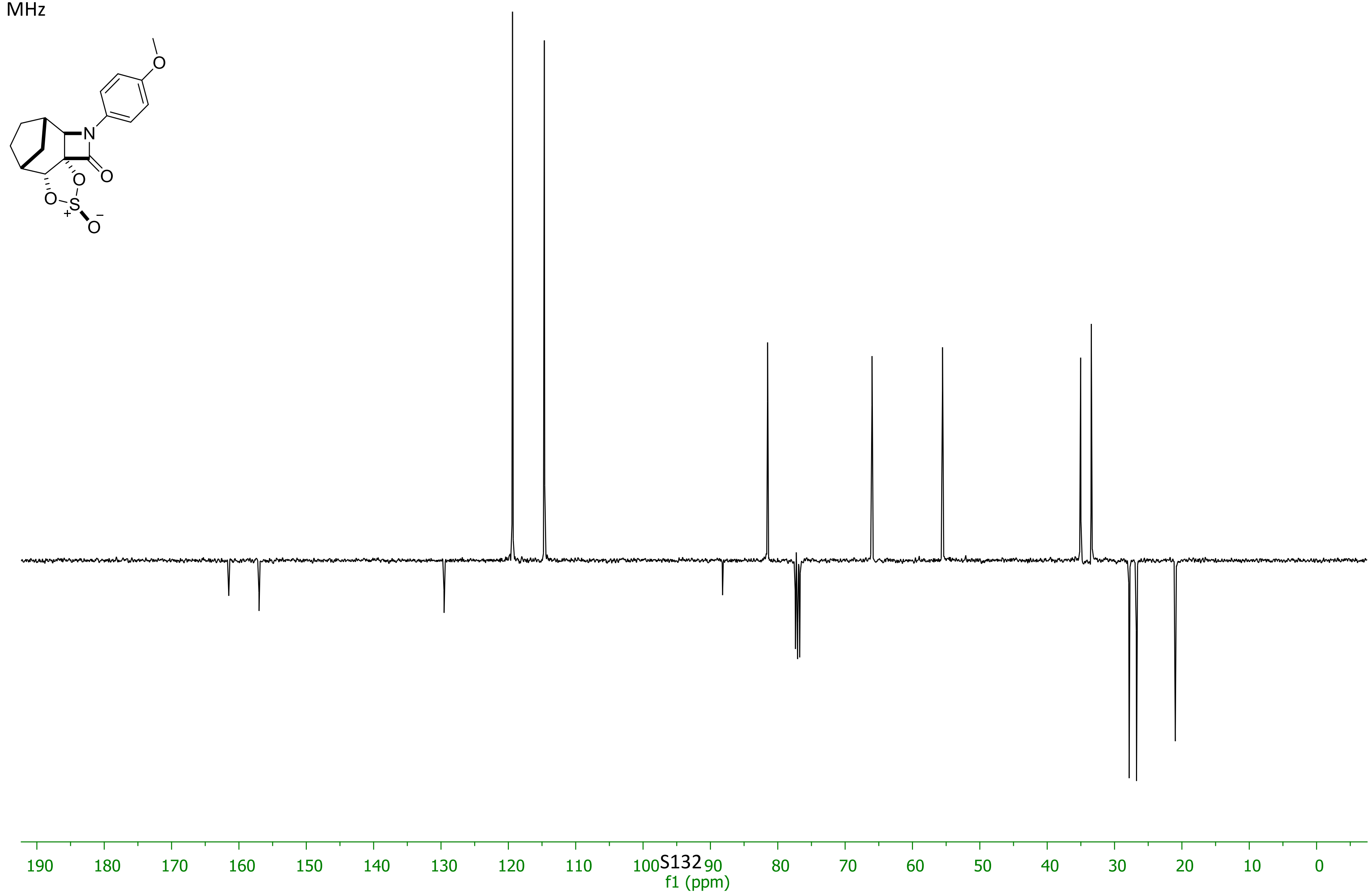

(±)-(3a*S*,5a*R*,6*S*,9*R*,9a*S*)-5-(4-Methoxyphenyl)hexahydro-6,9-methanol[1,3,2] dioxathiolo[4',5':2,3] cyclohepta[1,2-*b*]azet-4(5H)-one 2-oxide **103b**; $CDCl_3$, 400 MHz

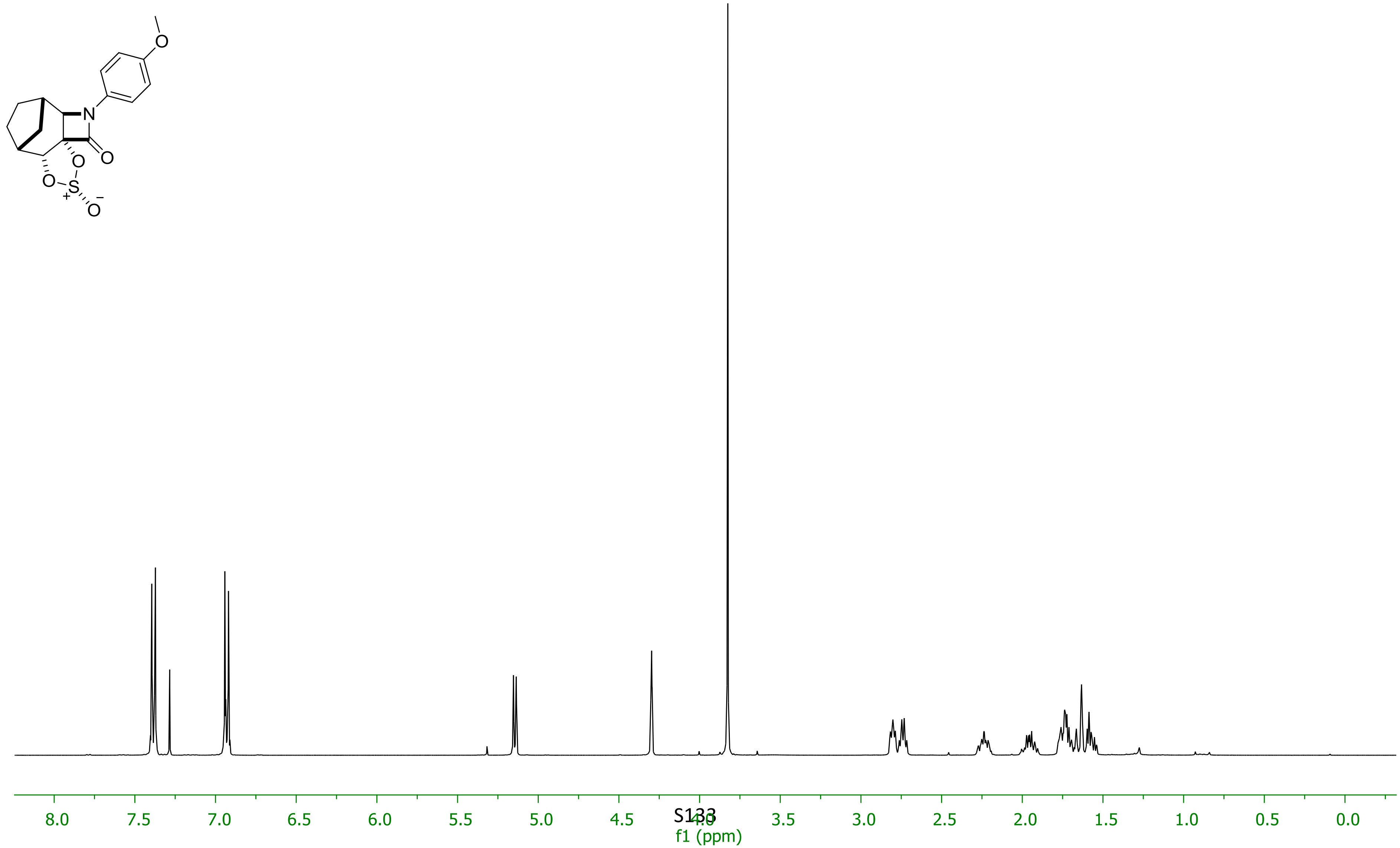

(±)-(3a*S*,5a*R*,6*S*,9*R*,9a*S*)-5-(4-Methoxyphenyl)hexahydro-6,9-methanol[1,3,2] dioxathiolo[4',5':2,3] cyclohepta[1,2-*b*]azet-4(5H)-one 2-oxide **103b**; $CDCl_3$, 100 MHz

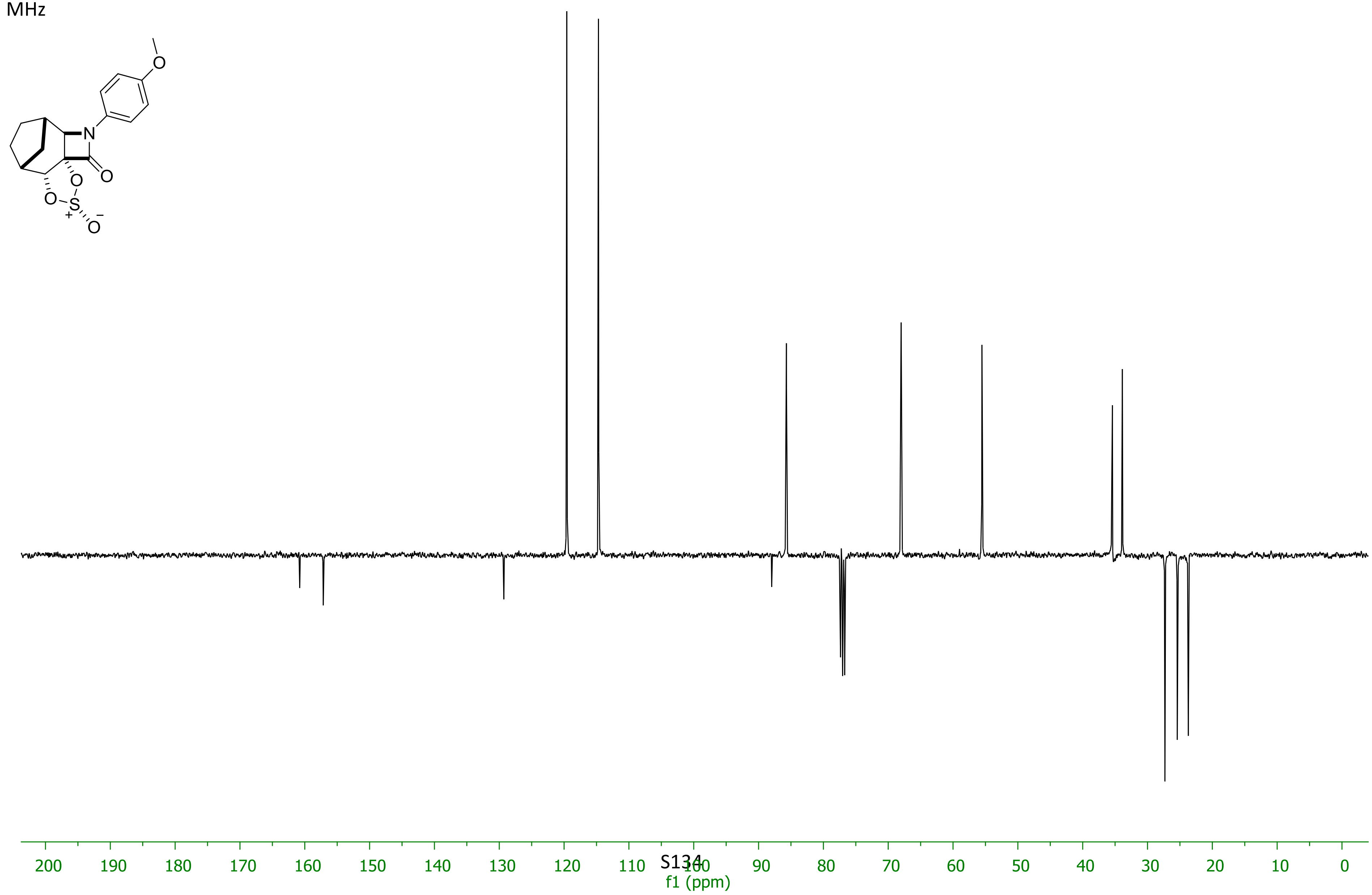

(±)-(1*S*,2*R*,6*R*)-3-(4-Methoxyphenyl)-3-azatricyclo[4.2.1.1^{2,5}]decan-4,10-dione **82**; $CDCl_3$, 400 MHz

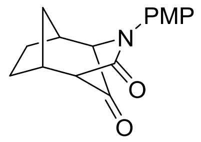

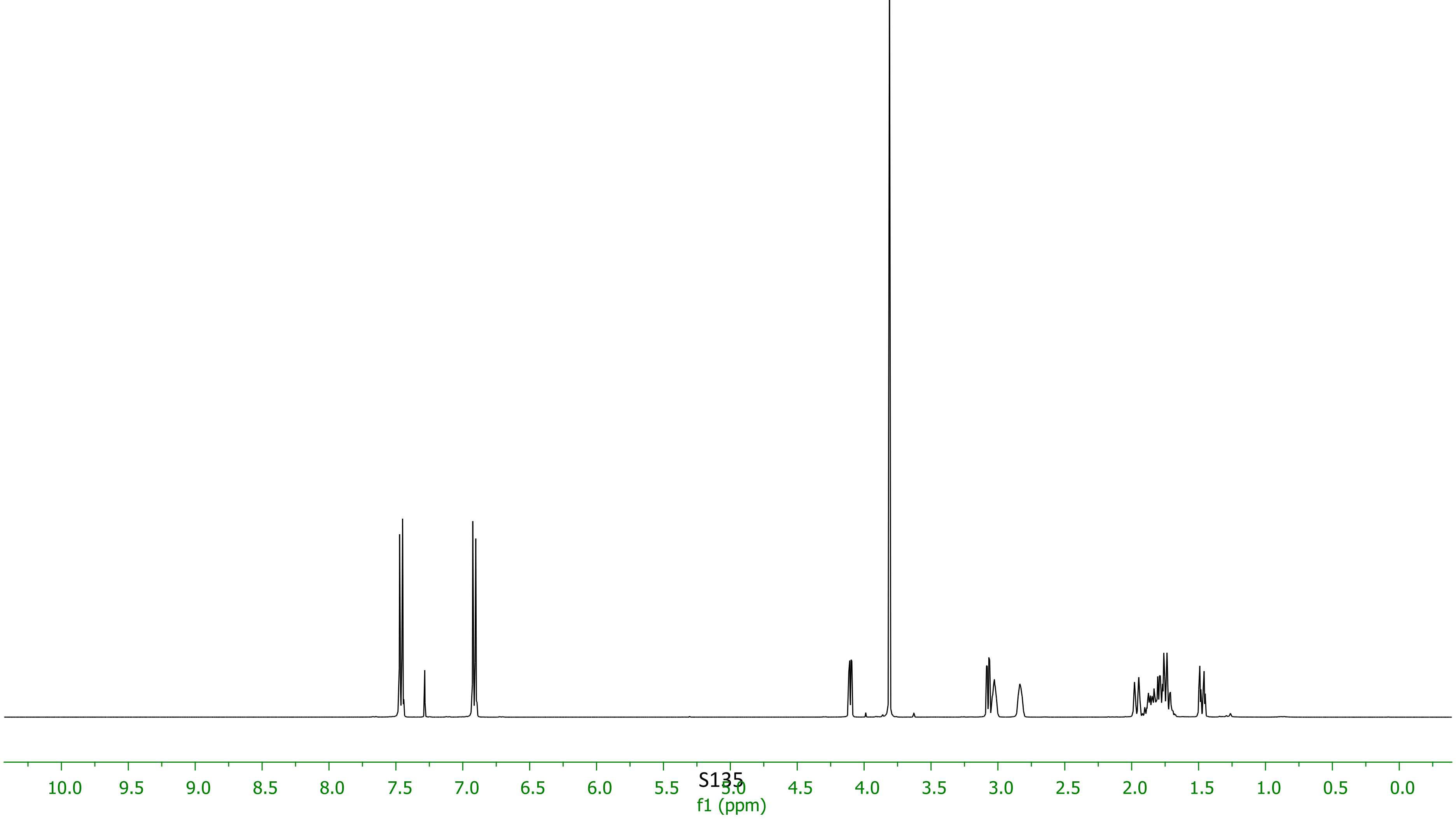

(±)-(1*S*,2*R*,6*R*)-3-(4-Methoxyphenyl)-3-azatricyclo[4.2.1.1^{2,5}]decan-4,10-dione **82**; $CDCl_3$, 100 MHz

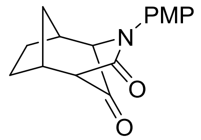

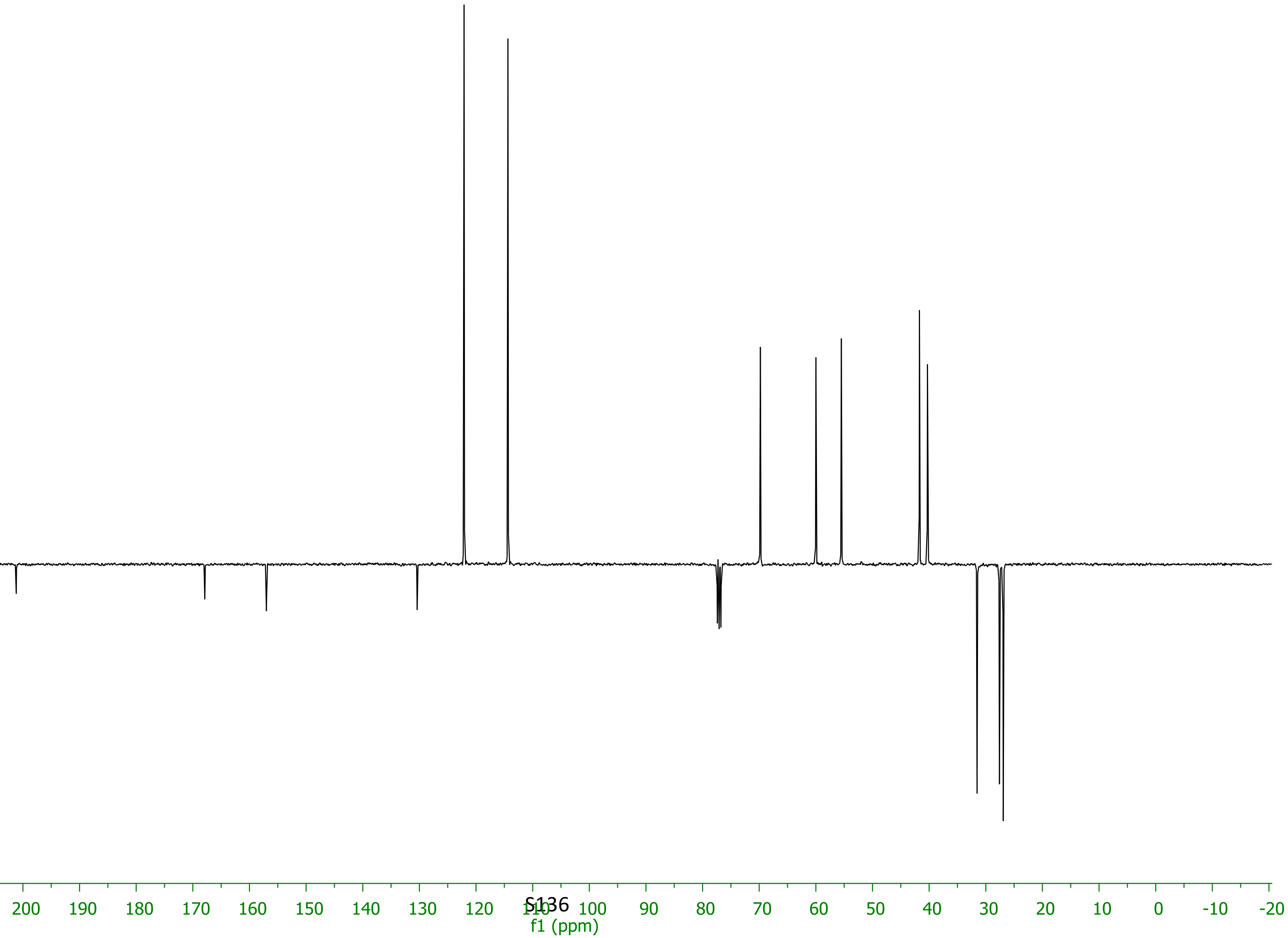

(±)-(1*S*,2*R*,5*S*,6*S*,7*R*)-6-Chloro-5-hydroxy-3-(4-methoxyphenyl)-3-azatricyclo[5.2.1.0^{2,5}] decan-4-one **83**; $CDCl_3$, 400 MHz

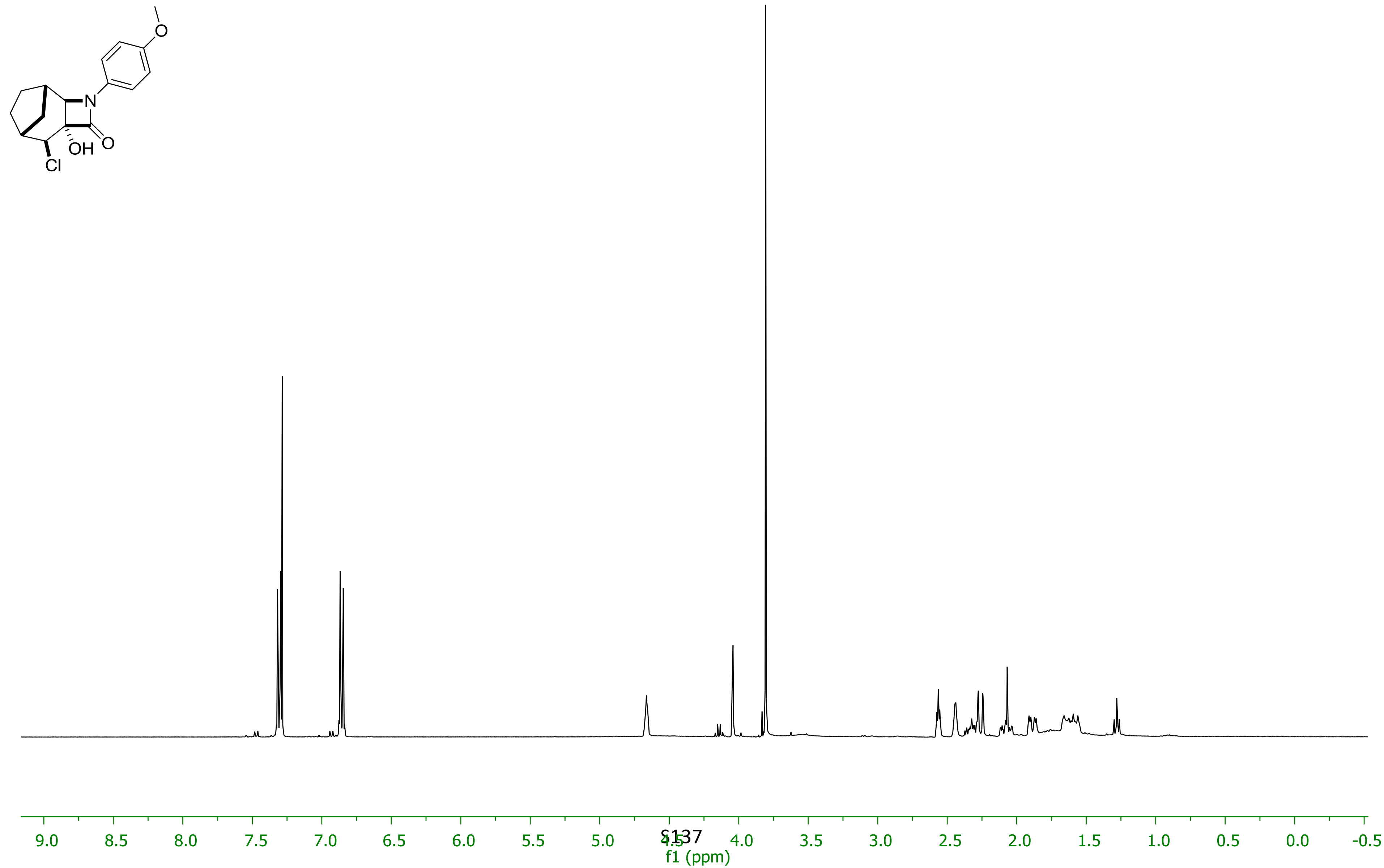

(±)-(1*S*,2*R*,5*S*,6*S*,7*R*)-6-Chloro-5-hydroxy-3-(4-methoxyphenyl)-3-azatricyclo[5.2.1.0^{2,5}] decan-4-one **83**; $CDCl_3$, 100 MHz

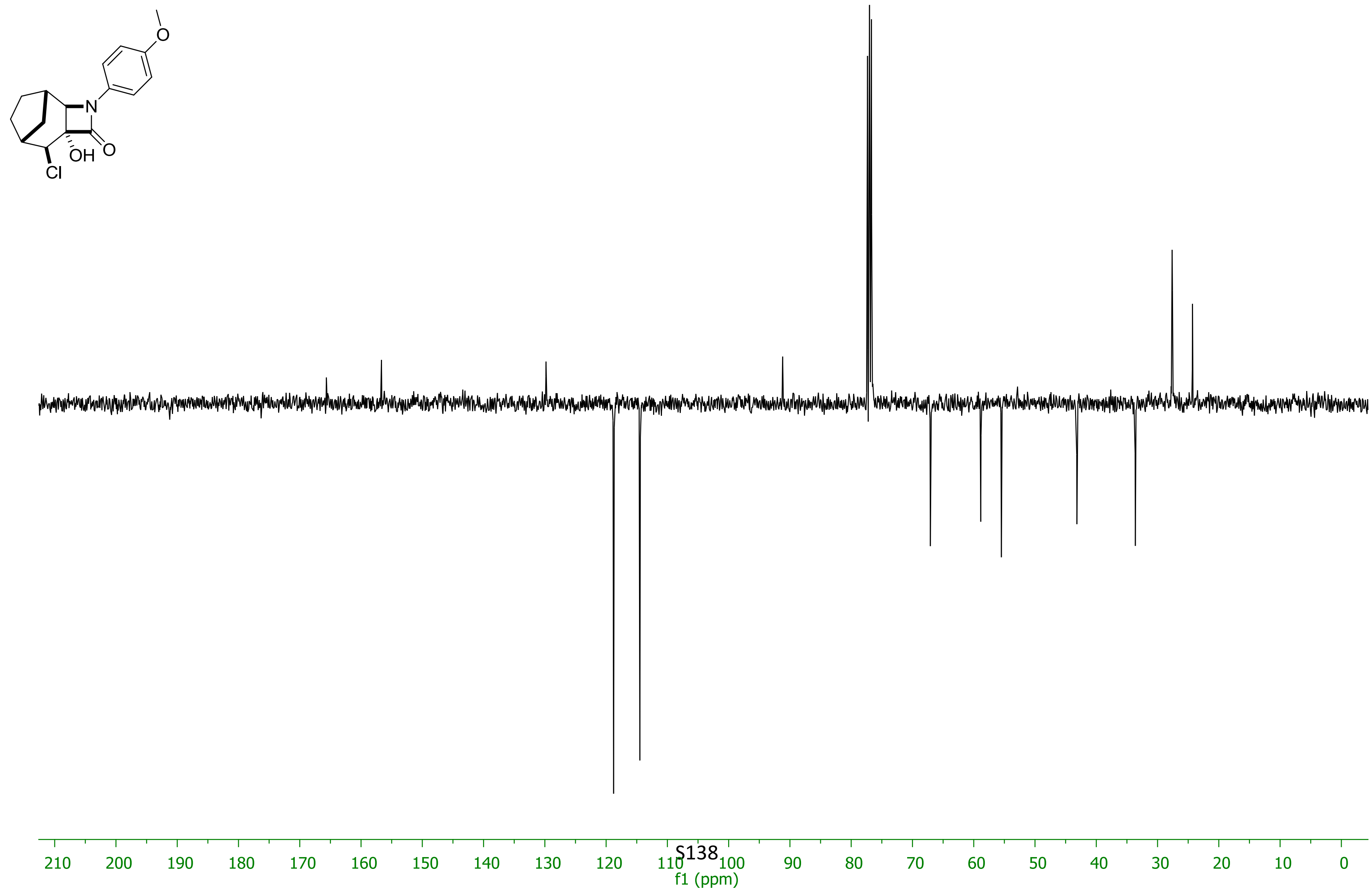

3-Hydroxy-3-(hydroxymethyl)-1-(4-methoxyphenyl)azetidin-2-one **78**; $CDCl_3$, 400 MHz

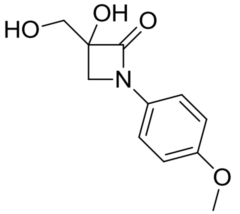

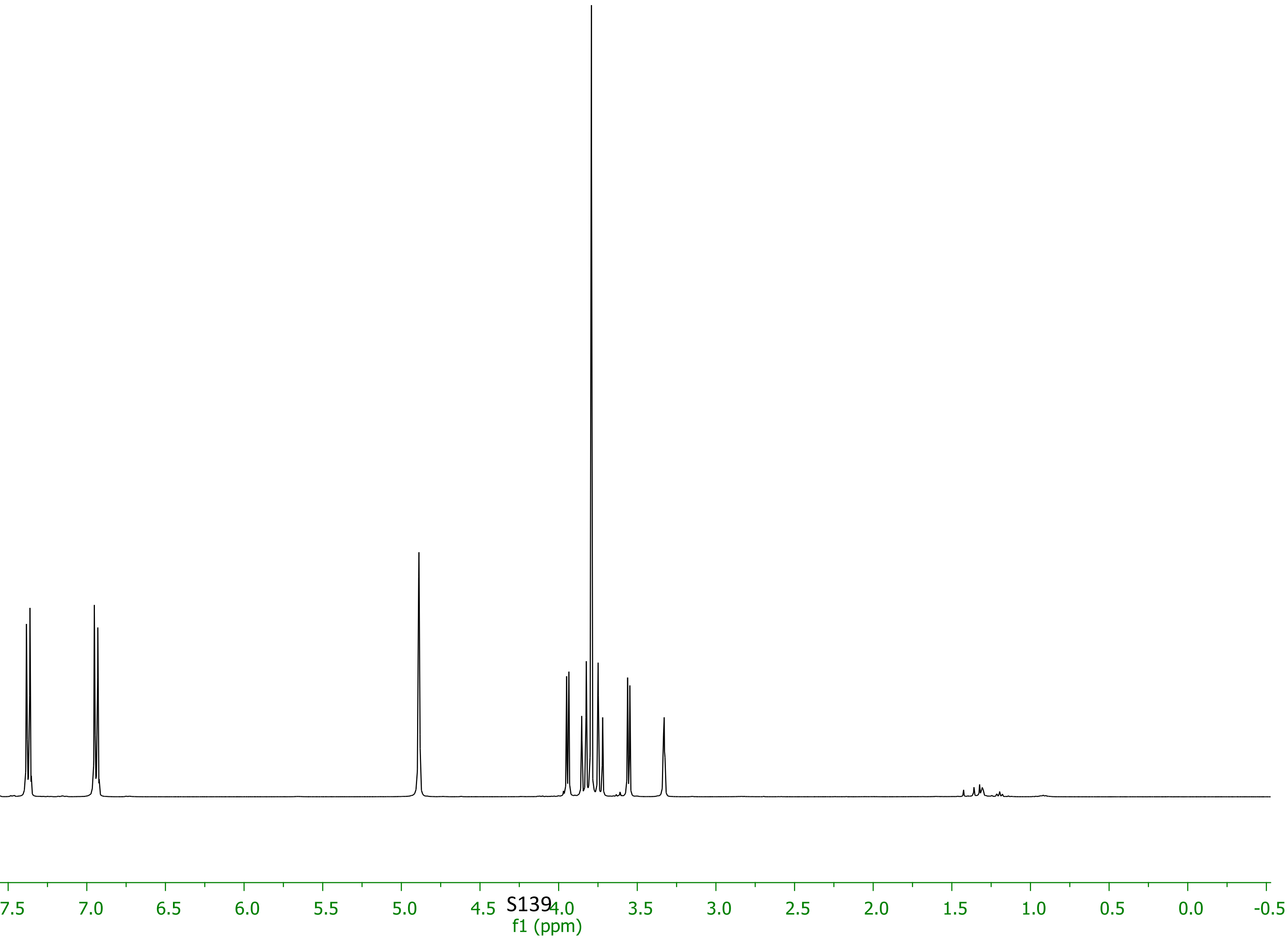

3-Hydroxy-3-(hydroxymethyl)-1-(4-methoxyphenyl)azetidin-2-one **78**; $CDCl_3$, 100 MHz

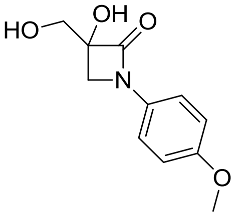

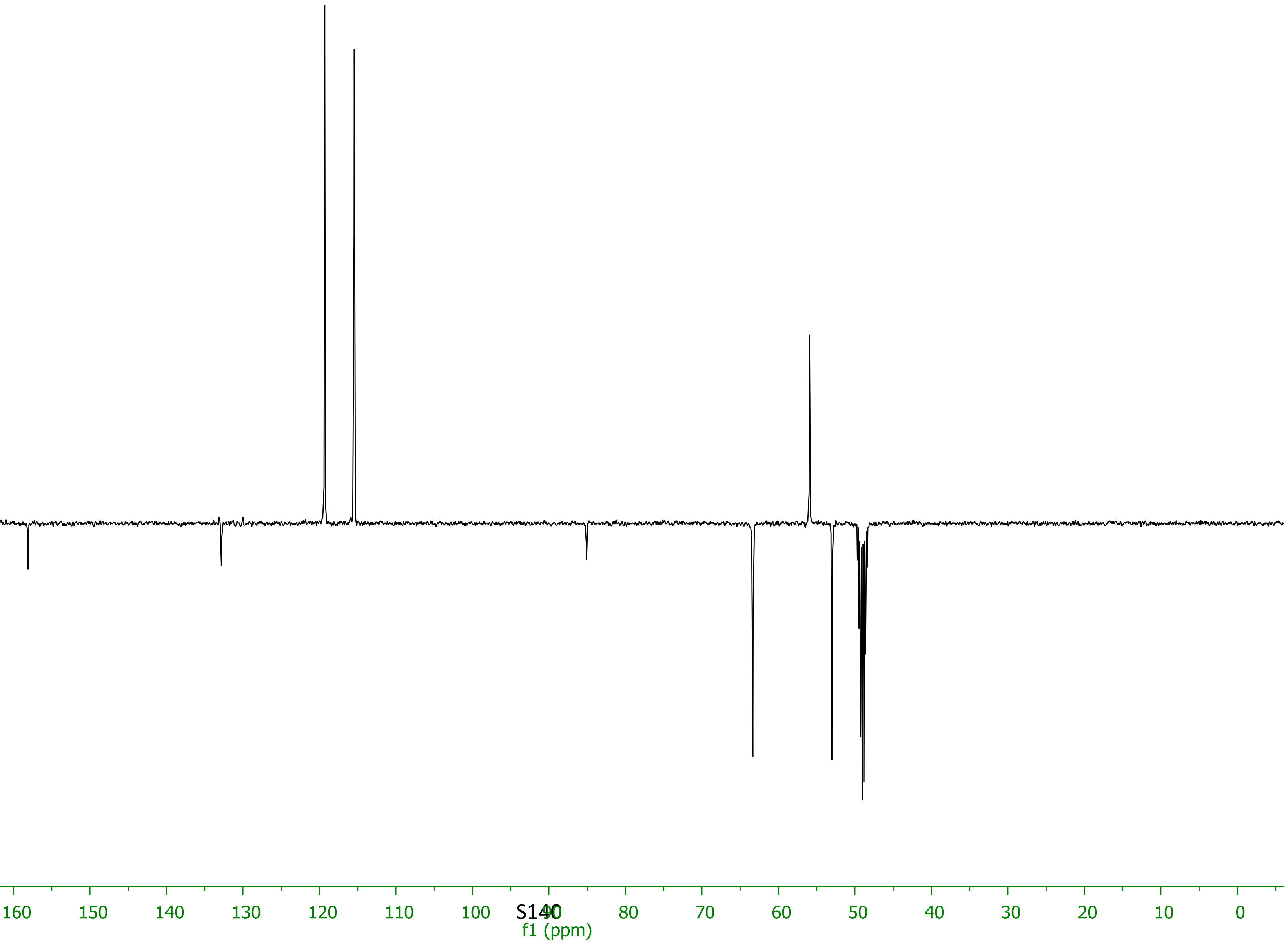

(±)-(4*S*)-2-(4-Methoxyphenyl)-5,7-dioxa-6-thia-2-azaspiro[3.4]octan-1-one 6-oxide **104a**; $CDCl_3$, 400 MHz

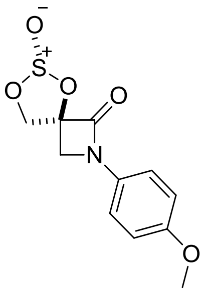

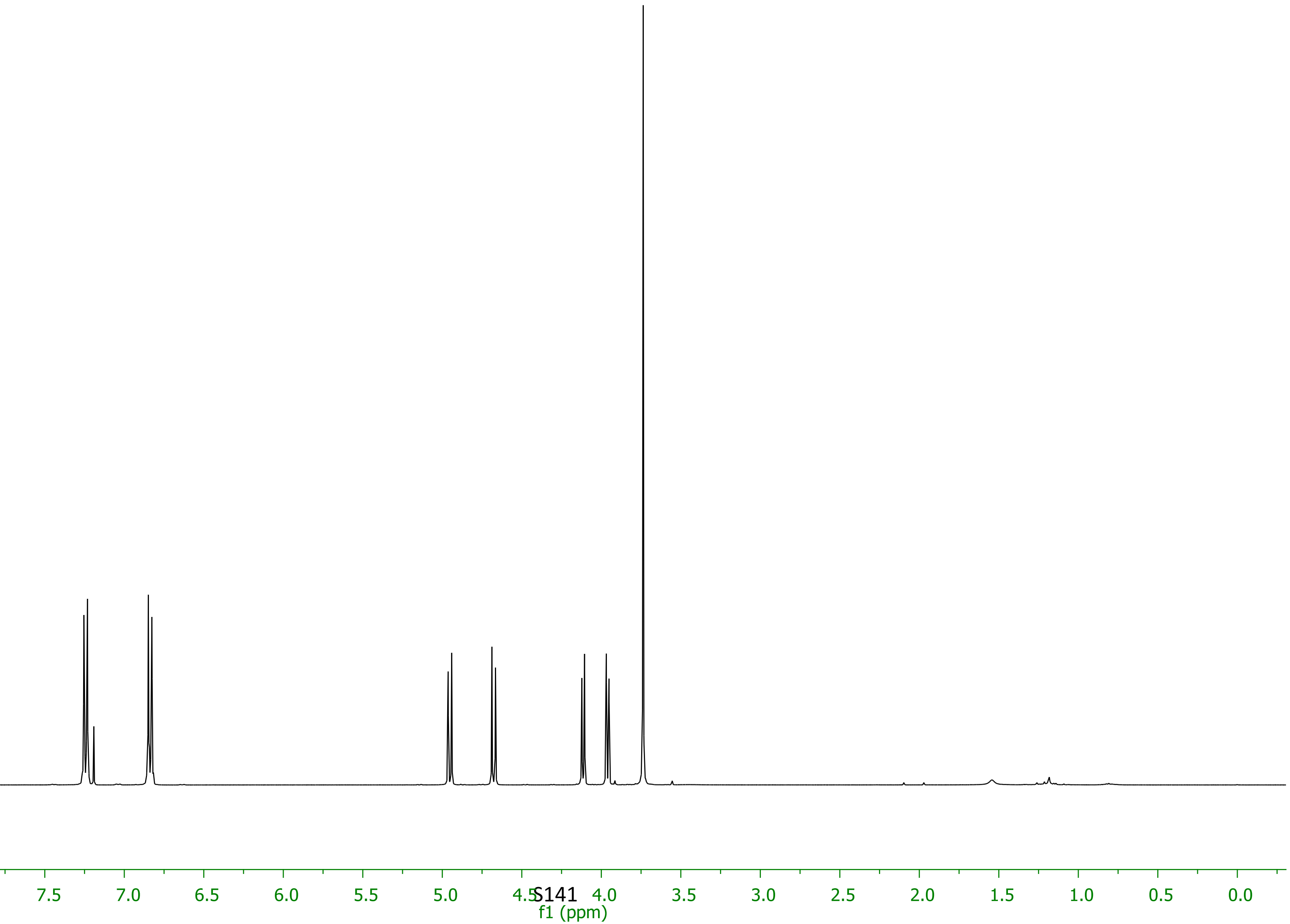

(±)-(4*S*)-2-(4-Methoxyphenyl)-5,7-dioxa-6-thia-2-azaspiro[3.4]octan-1-one 6-oxide **104a**; $CDCl_3$, 100 MHz

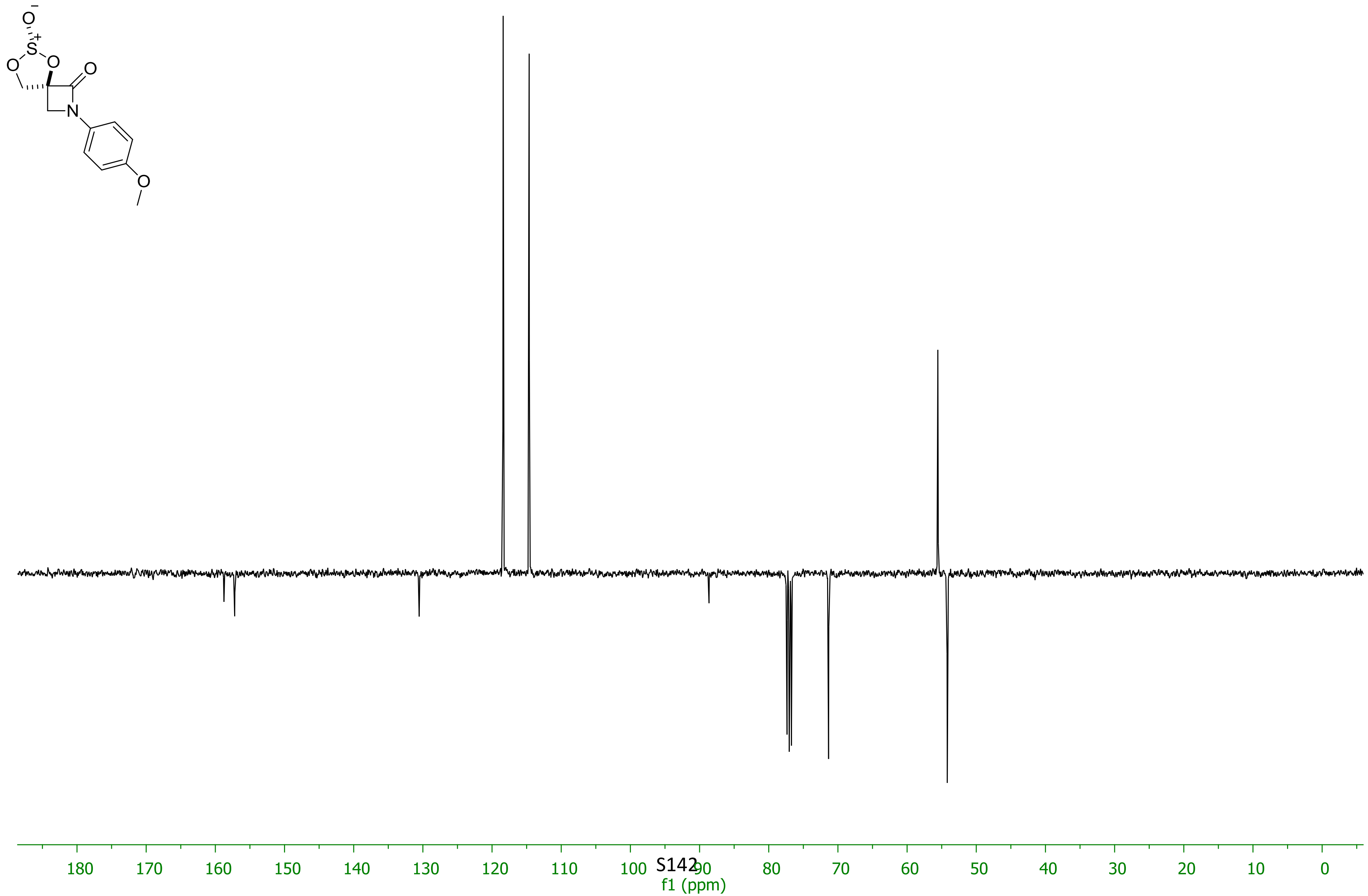

(±)-(4*S*)-2-(4-Methoxyphenyl)-5,7-dioxa-6-thia-2-azaspiro[3.4]octan-1-one 6-oxide **104b**; $CDCl_3$, 400 MHz

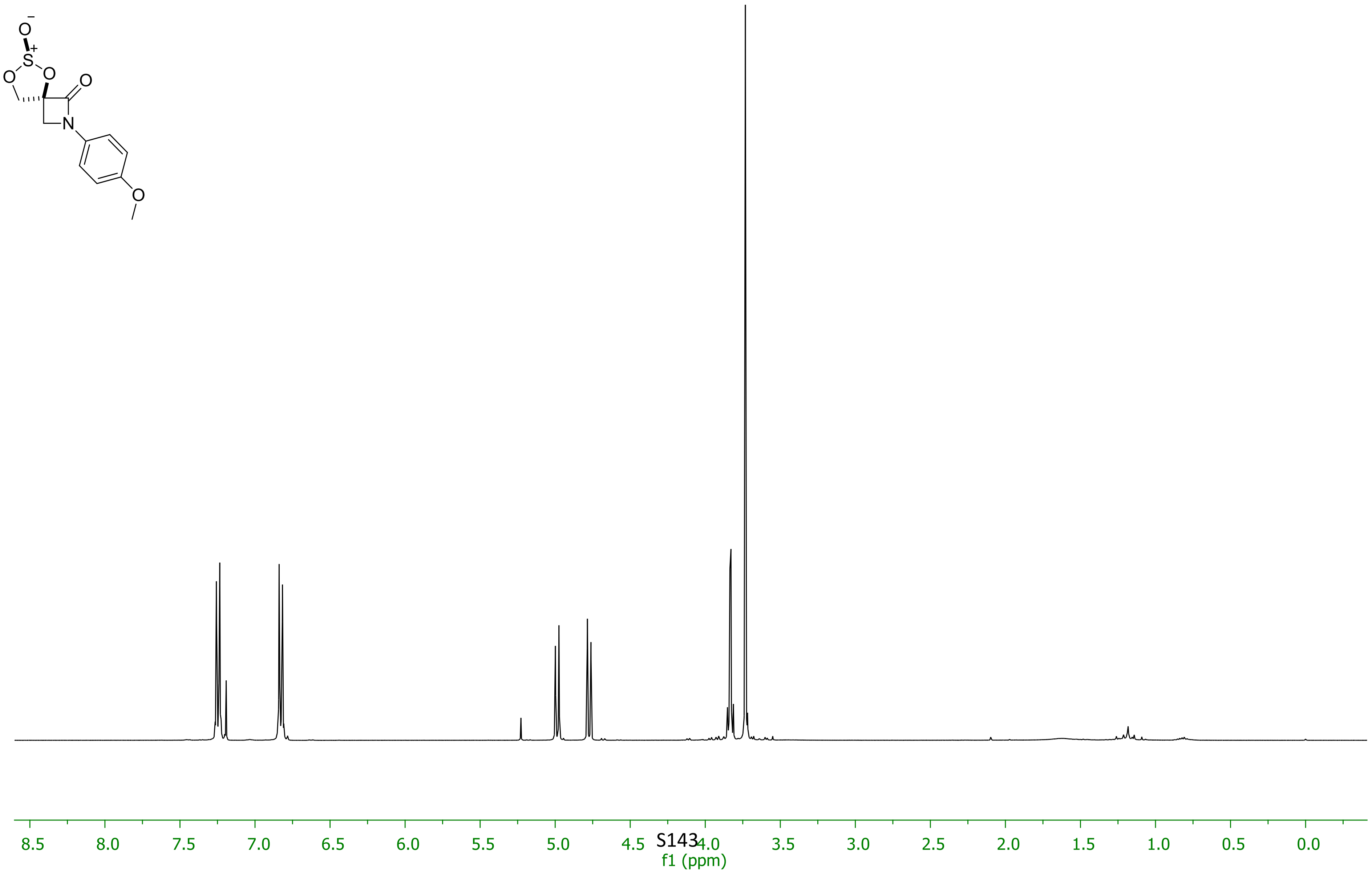

(±)-(4*S*)-2-(4-Methoxyphenyl)-5,7-dioxa-6-thia-2-azaspiro[3.4]octan-1-one 6-oxide **104b**; $CDCl_3$, 100 MHz

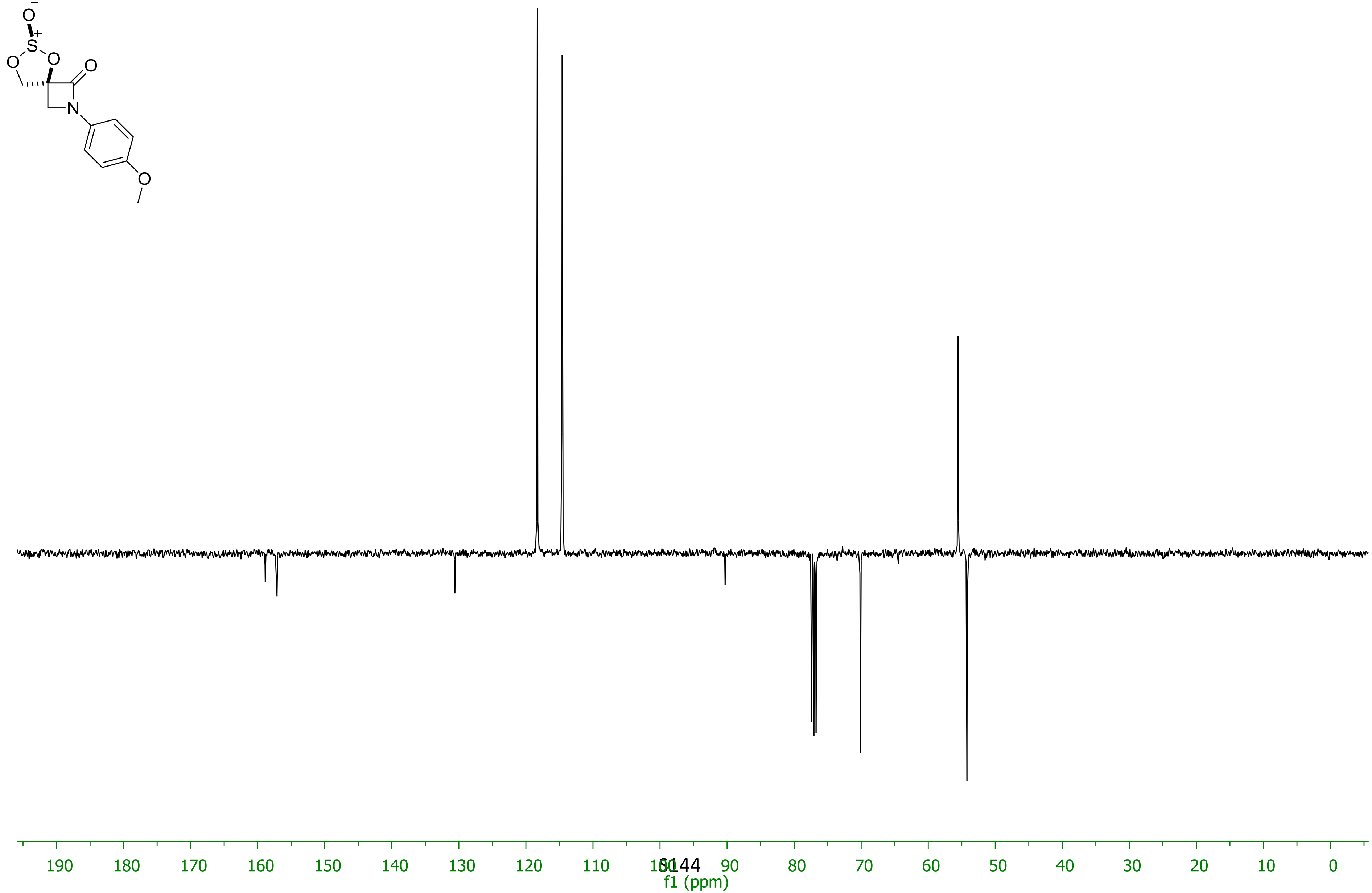

3-(Chloromethyl)-3-hydroxy-1-(4-methoxyphenyl)azetidin-2-one **84**; $CDCl_3$, 400 MHz

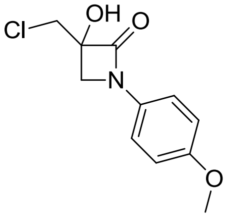

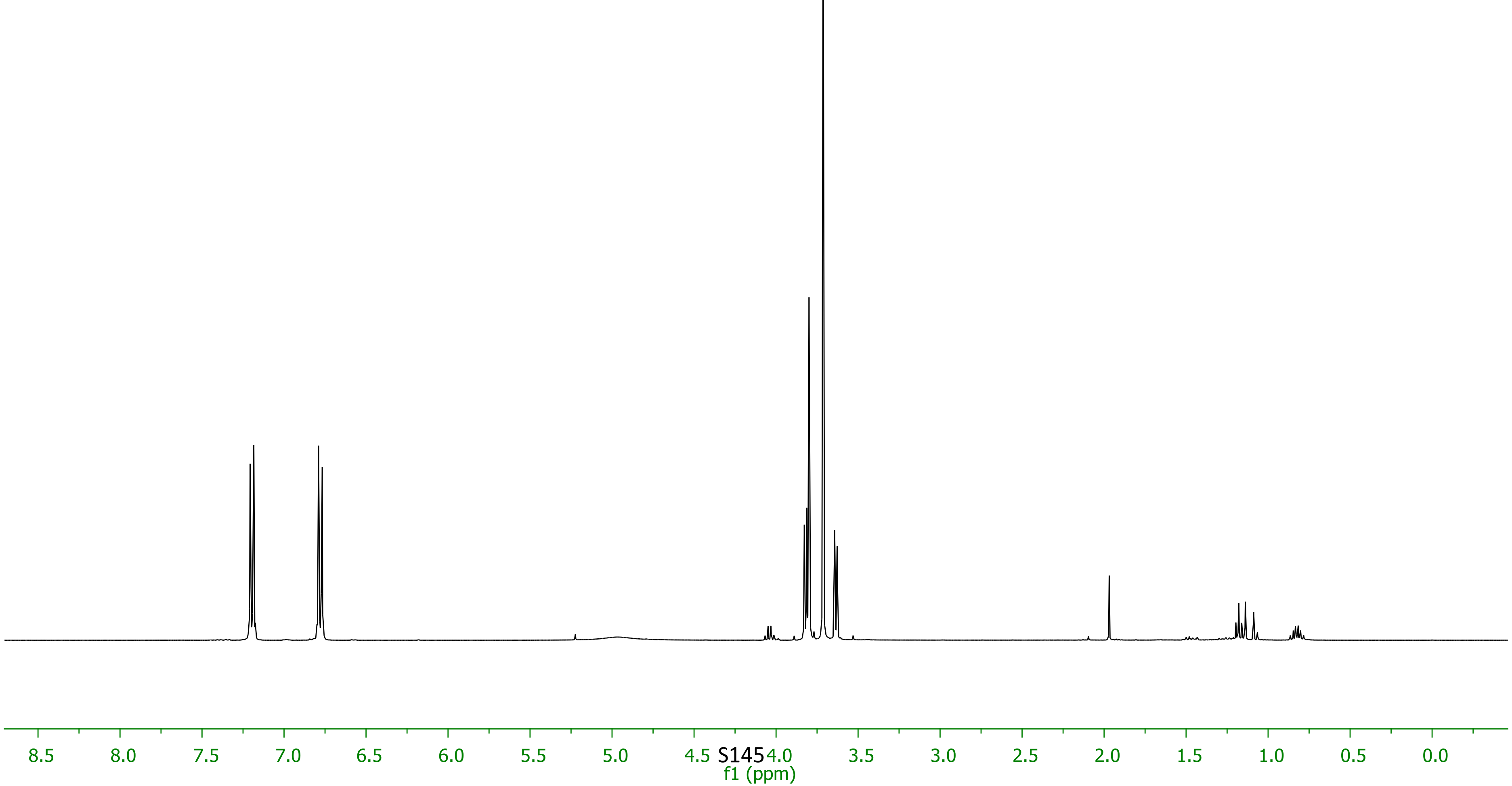

3-(Chloromethyl)-3-hydroxy-1-(4-methoxyphenyl)azetidin-2-one **84**; $CDCl_3$, 100 MHz

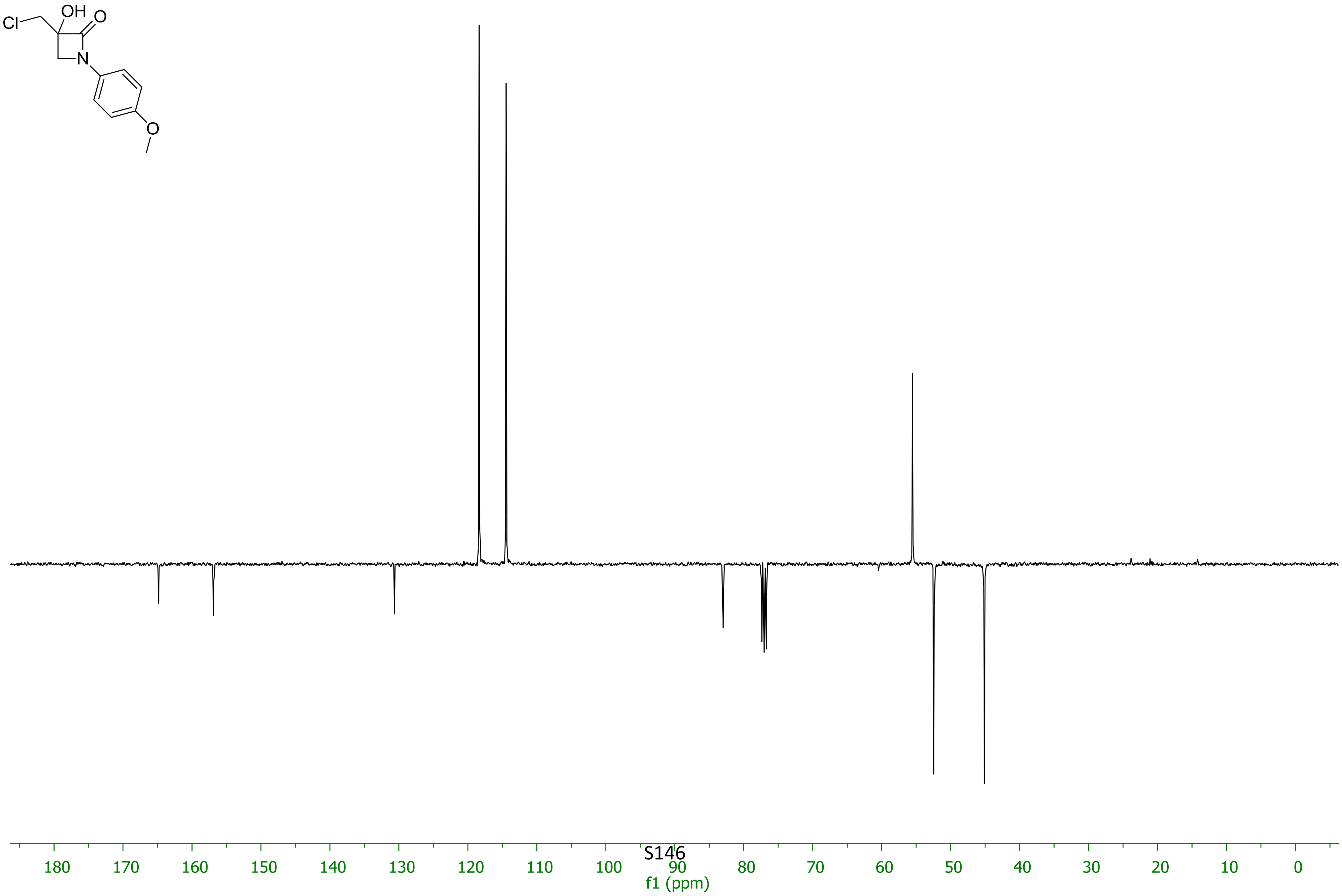

(±)-(*1R,5S,8S*)-8-Hydroxy-6-(4-methoxyphenyl)-6-azabicyclo[3.2.1]octan-7-one **85**; $CDCl_3$, 400 MHz

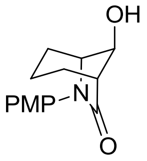

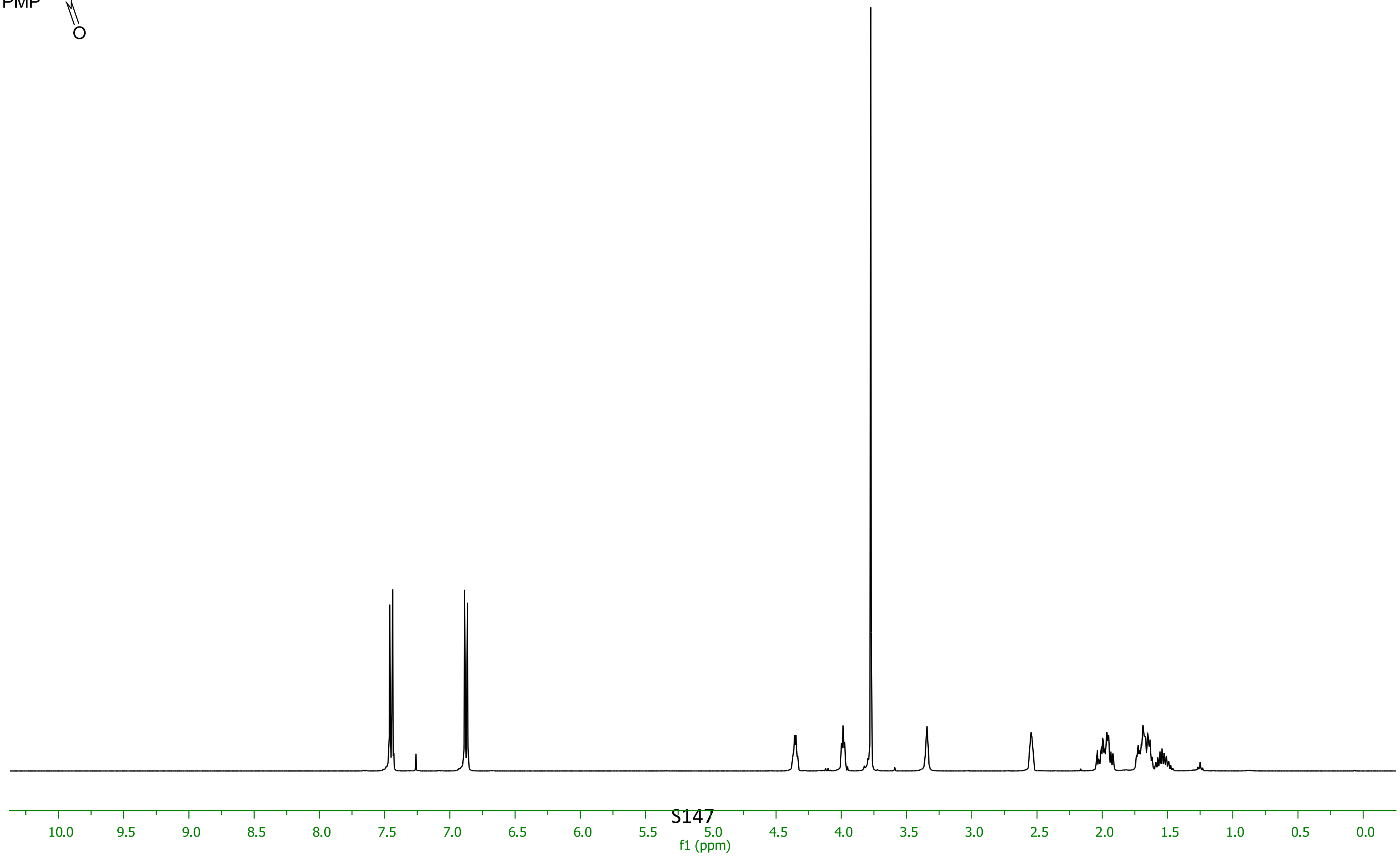

(±)-(*1R,5S,8S*)-8-Hydroxy-6-(4-methoxyphenyl)-6-azabicyclo[3.2.1]octan-7-one **85**; $CDCl_3$, 100 MHz

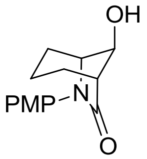

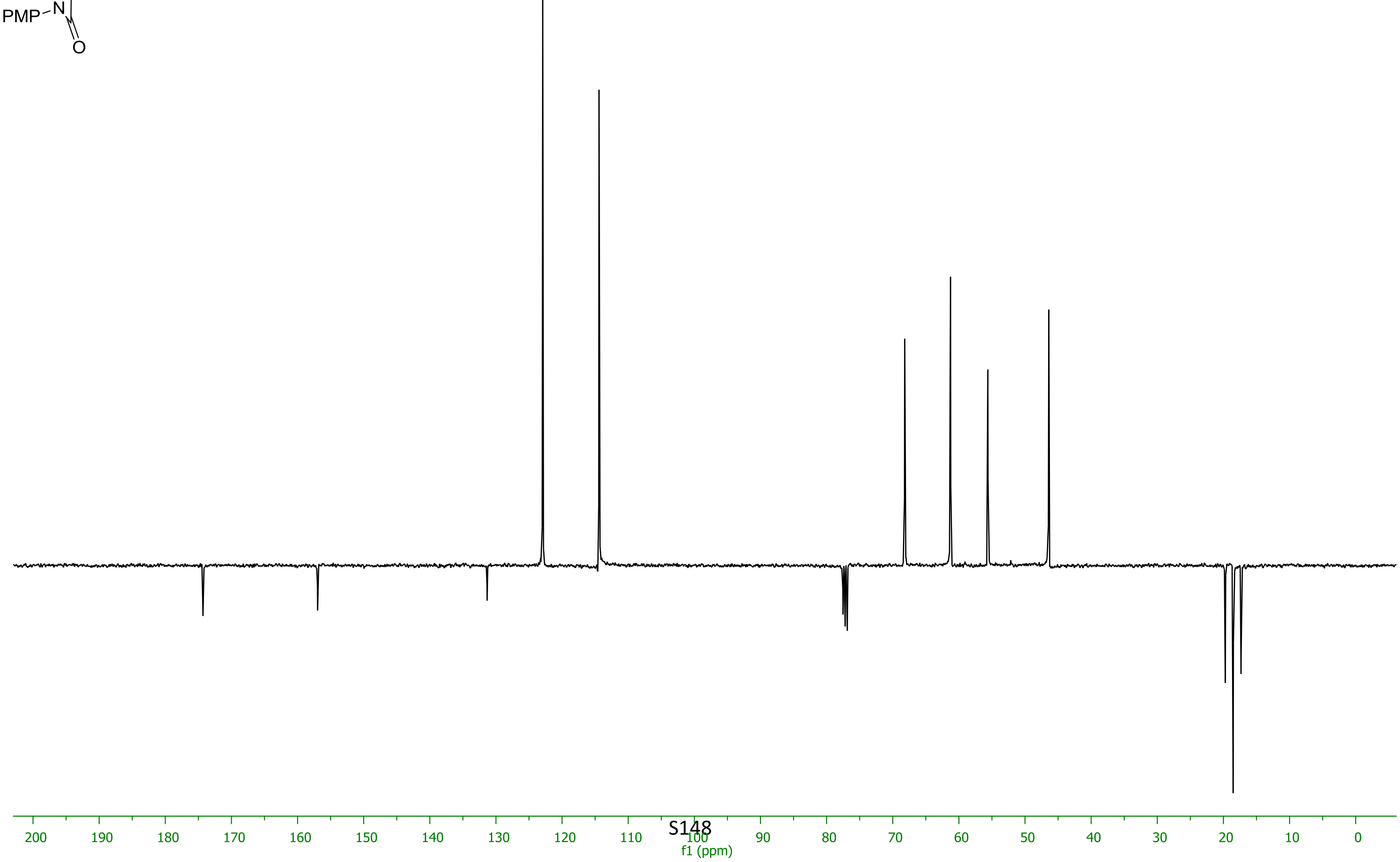

(±)-(1*R*,5*S*)-6-(4-Methoxyphenyl)-8-methylene-6-azabicyclo[3.2.1]octane-7-one **86**; $CDCl_3$, 400 MHz

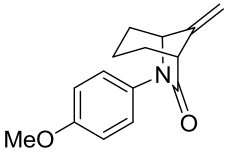

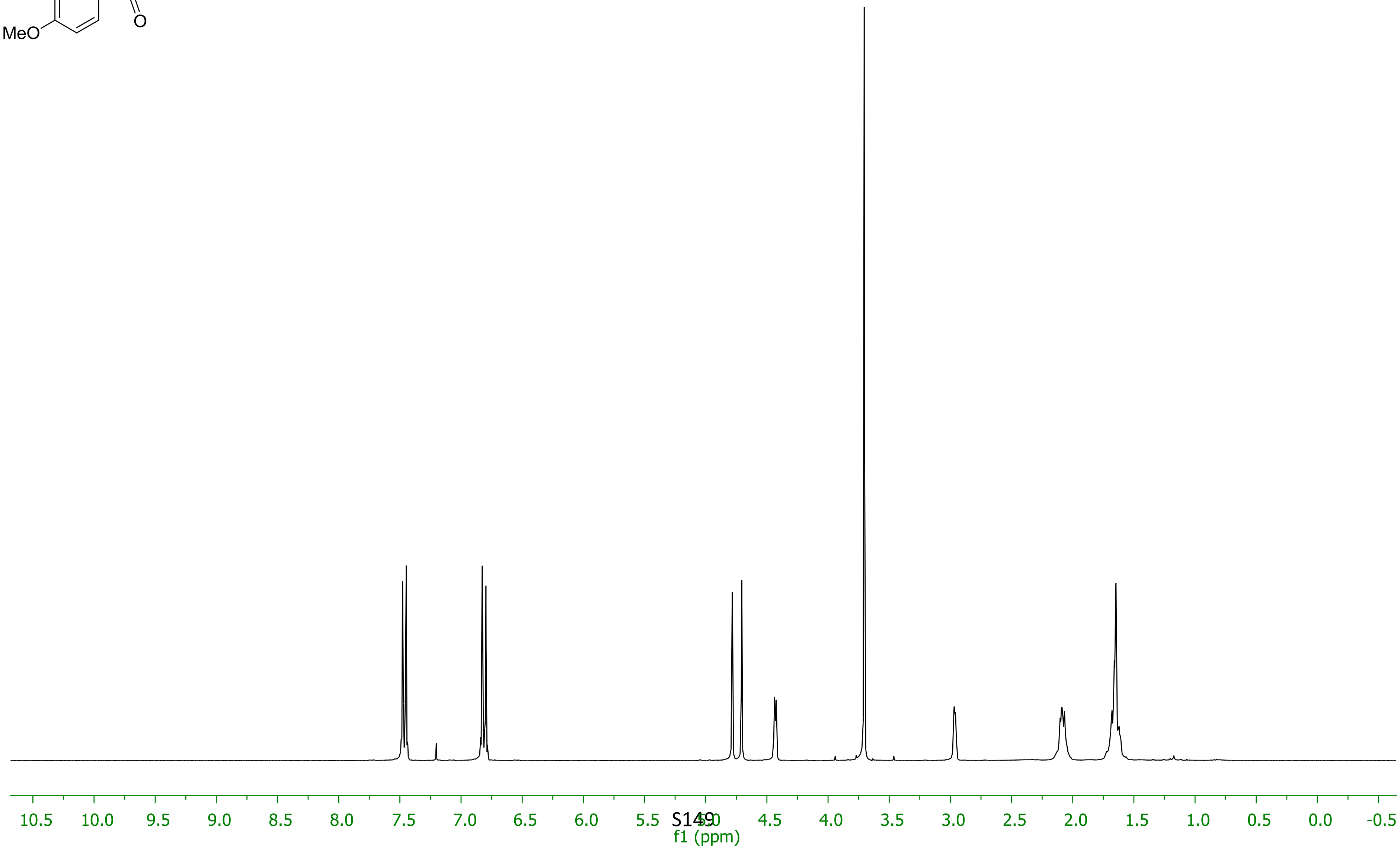

(±)-(1*R*,5*S*)-6-(4-Methoxyphenyl)-8-methylene-6-azabicyclo[3.2.1]octane-7-one **86**; $CDCl_3$, 100 MHz

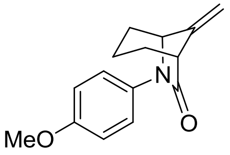

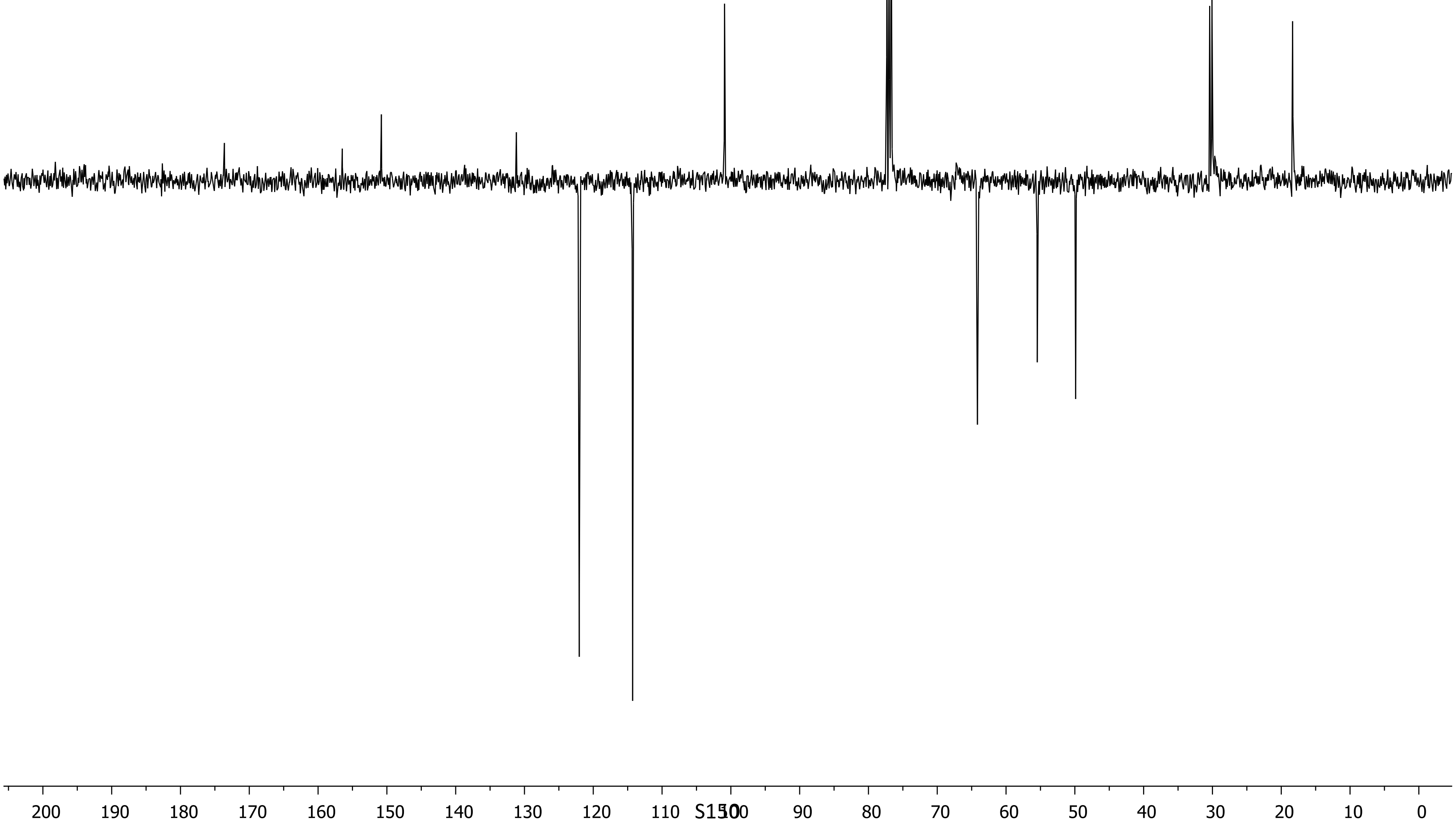

(±)-(1*R*,5*S*)-6-(4-Methoxyphenyl)-8-methylene-6-azabicyclo[3.2.1]octane-7-thione **87**; $CDCl_3$, 400 MHz

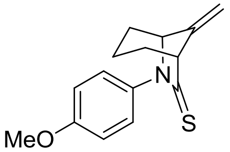

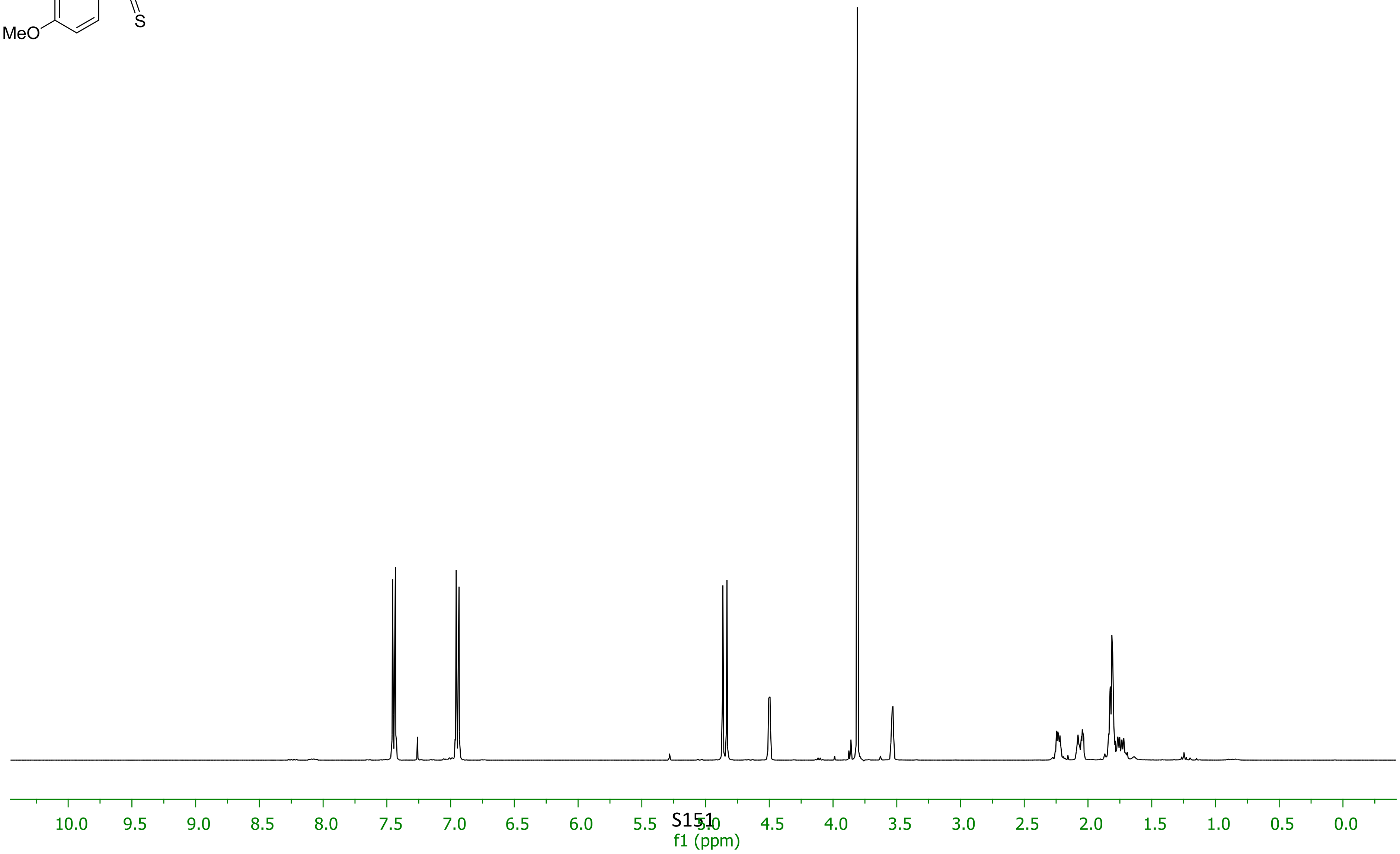

(±)-(1*R*,5*S*)-6-(4-Methoxyphenyl)-8-methylene-6-azabicyclo[3.2.1]octane-7-thione **87**; $CDCl_3$, 100 MHz

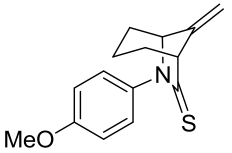

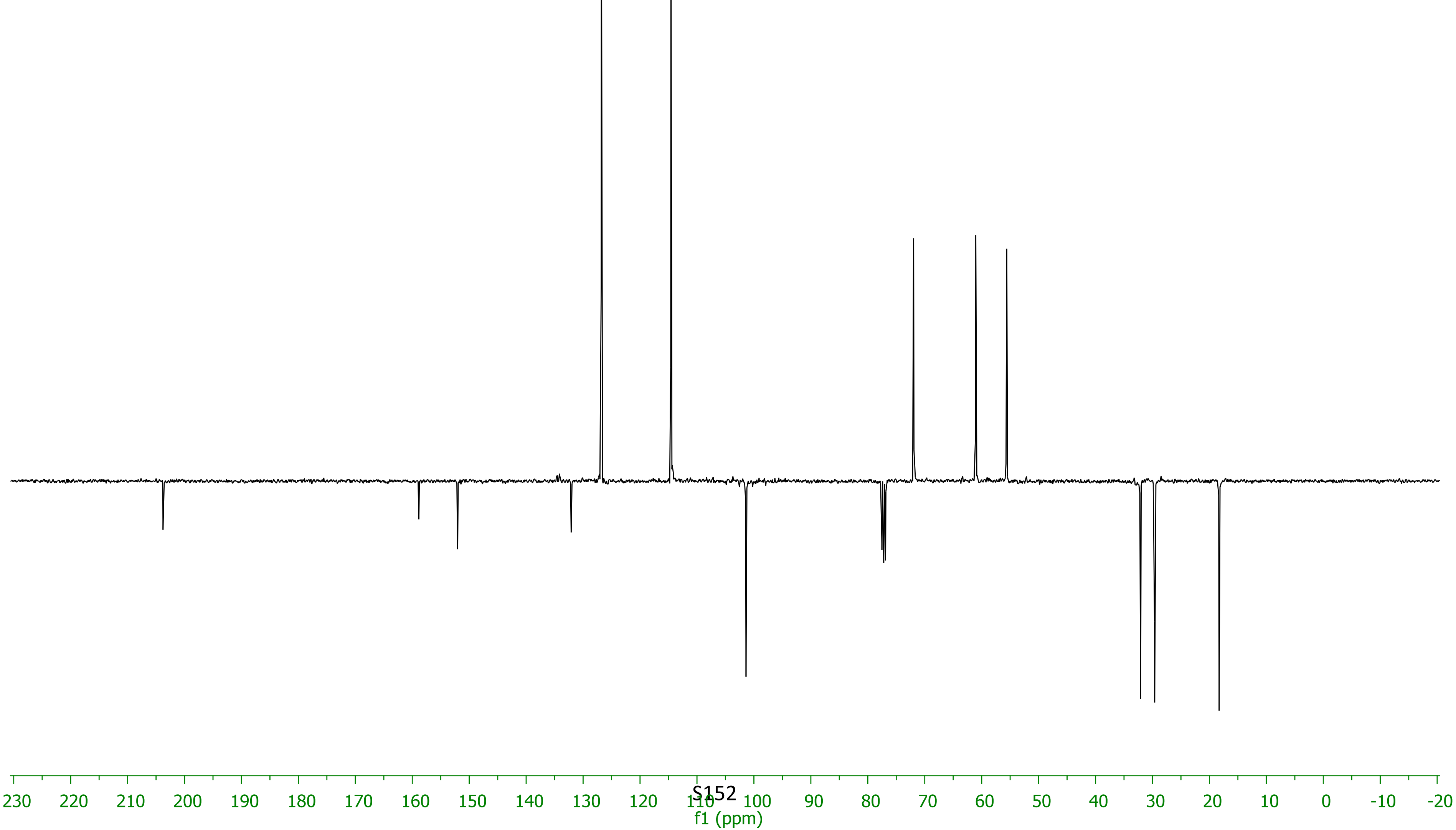

(±)-(1*R*,5*S*)-6-(4-Methoxyphenyl)-8-methylene-7-(methylthio)-6-azabicyclo[3.2.1] octan-6-en-6-ium iodide **88**; $CDCl_3$, 400 MHz

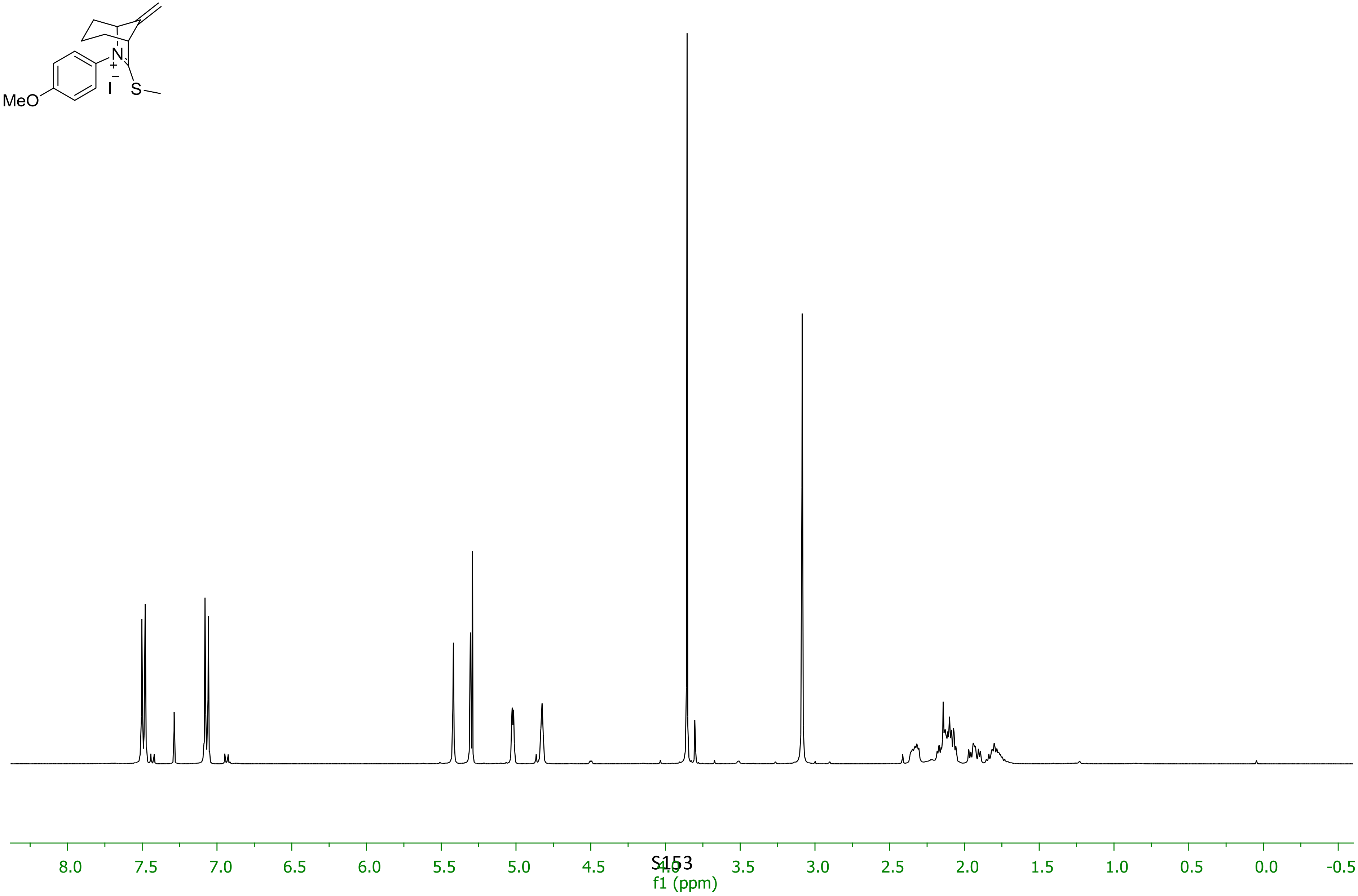

(±)-(1*R*,5*S*)-6-(4-Methoxyphenyl)-8-methylene-7-(methylthio)-6-azabicyclo[3.2.1] octan-6-en-6-ium iodide **88**; $CDCl_3$, 100 MHz

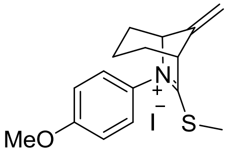

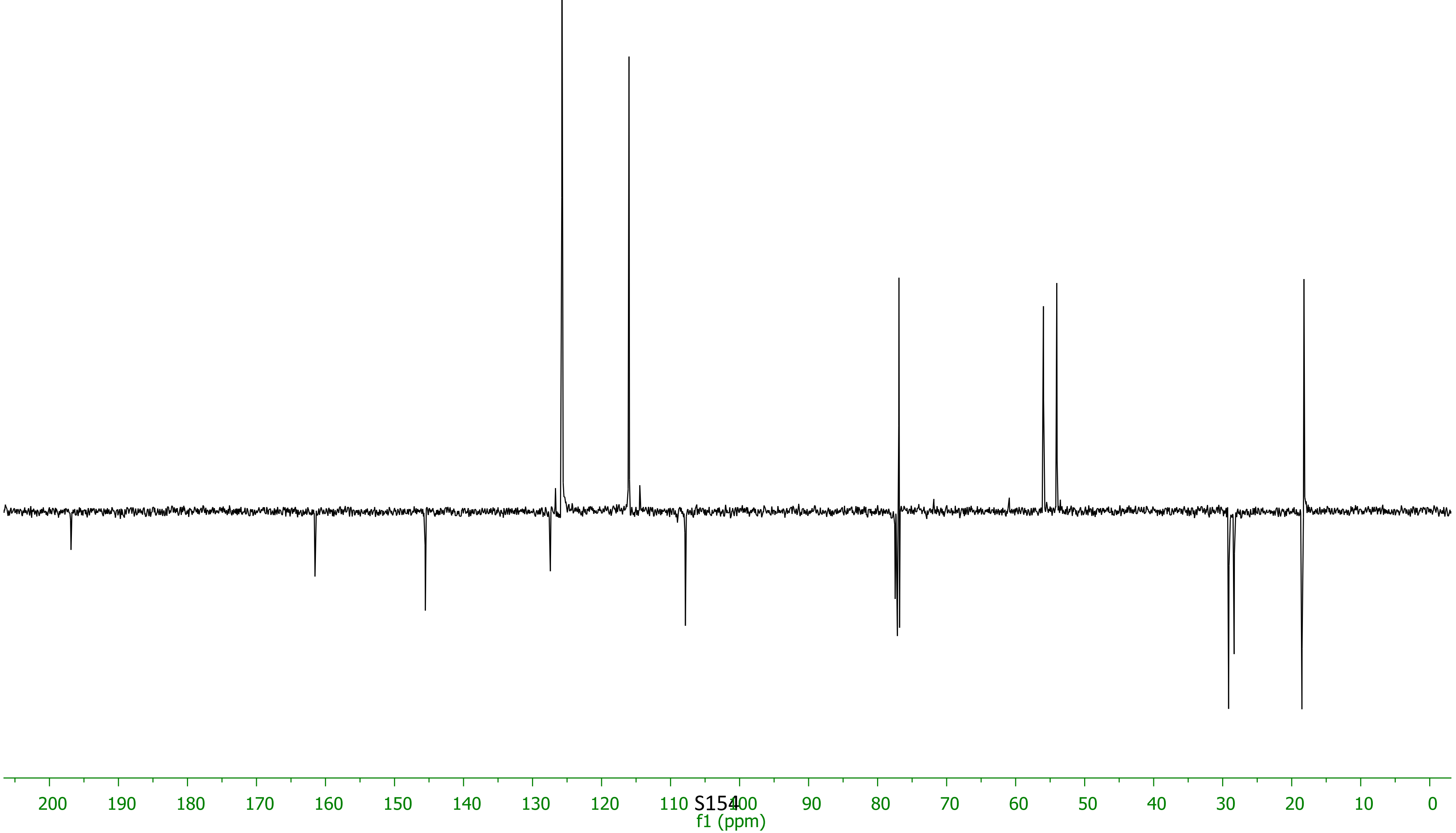

(±)-(1*R*,5*S*,7*R*)-7-Ally-6-(4-methoxyphenyl)-8-methylene-6-azabicyclo[3.2.1]octane **89**; $CDCl_3$, 400 MHz

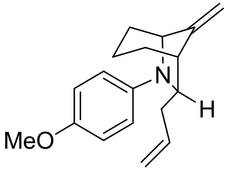

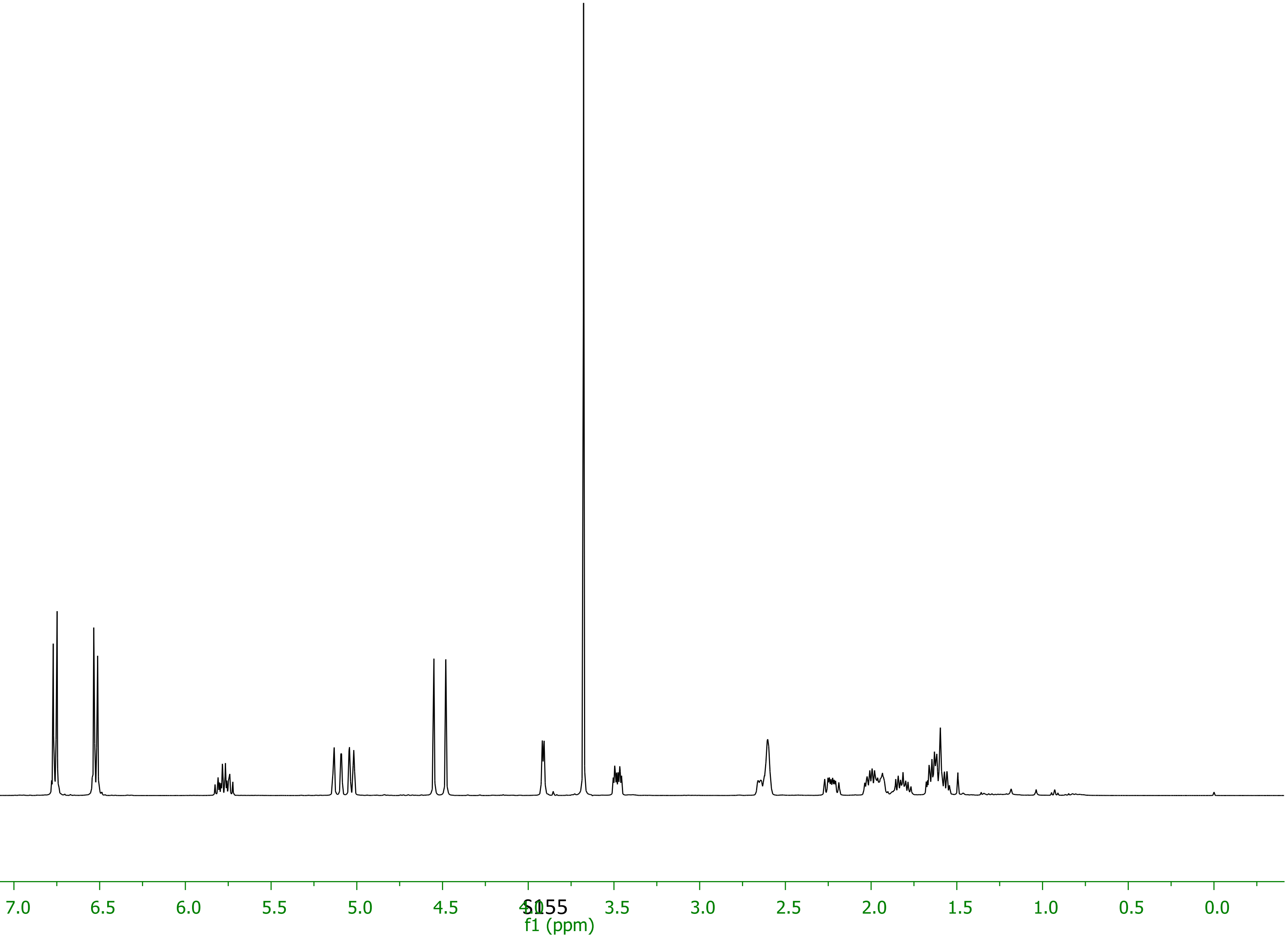

(±)-(1*R*,5*S*,7*R*)-7-Ally-6-(4-methoxyphenyl)-8-methylene-6-azabicyclo[3.2.1]octane **89**; $CDCl_3$, 100 MHz

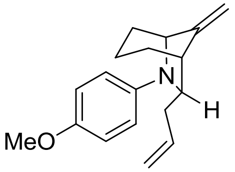

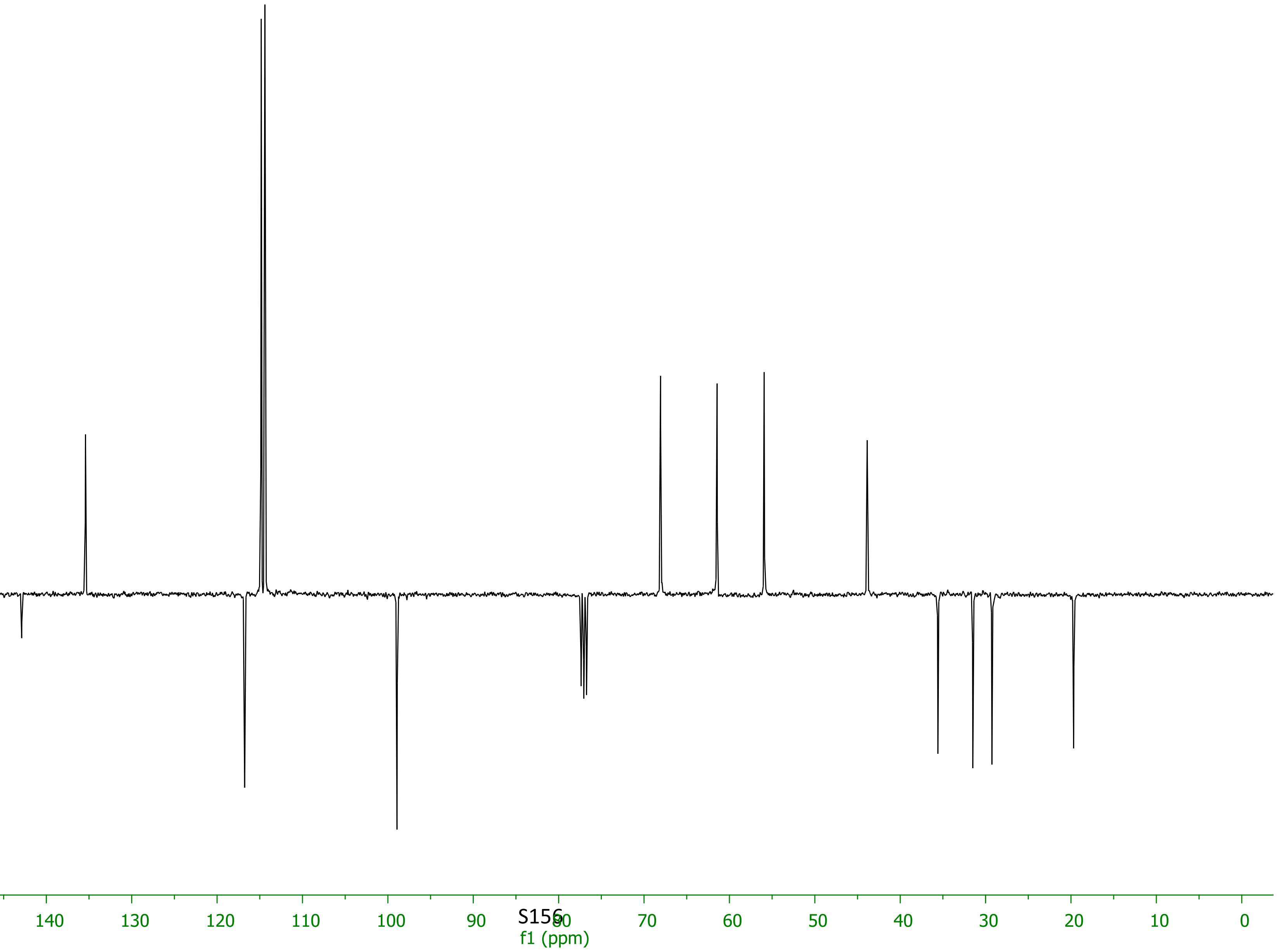

(±)-(3a*R*,5a*S*,8a*S*)-5-Benzyl-3-mesityl-5,5a,6,7,8,8a-hexahydro-4*H*-azeto[3',2':1,6] benzo[1,2-*d*] isoxazol-4-onemesitaldehyde oxime **91**; $CDCl_3$, 400 MHz

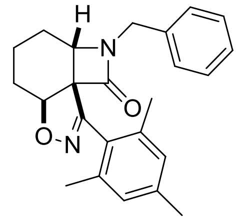

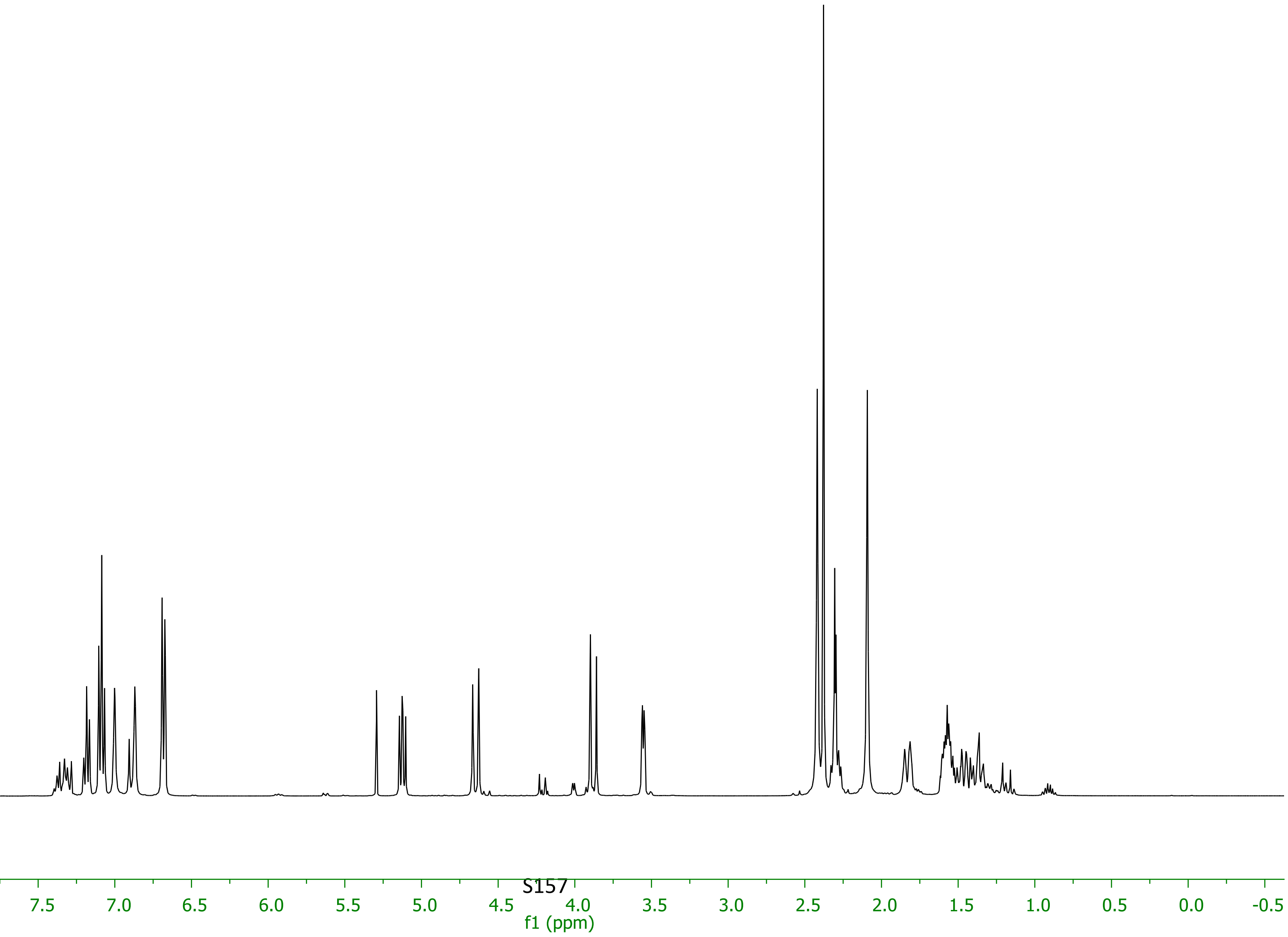

(±)-(3a*R*,5a*S*,8a*S*)-5-Benzyl-3-mesityl-5,5a,6,7,8,8a-hexahydro-4*H*-azeto[3',2':1,6] benzo[1,2-*d*] isoxazol-4-onemesitaldehyde oxime **91**; $CDCl_3$, 100 MHz

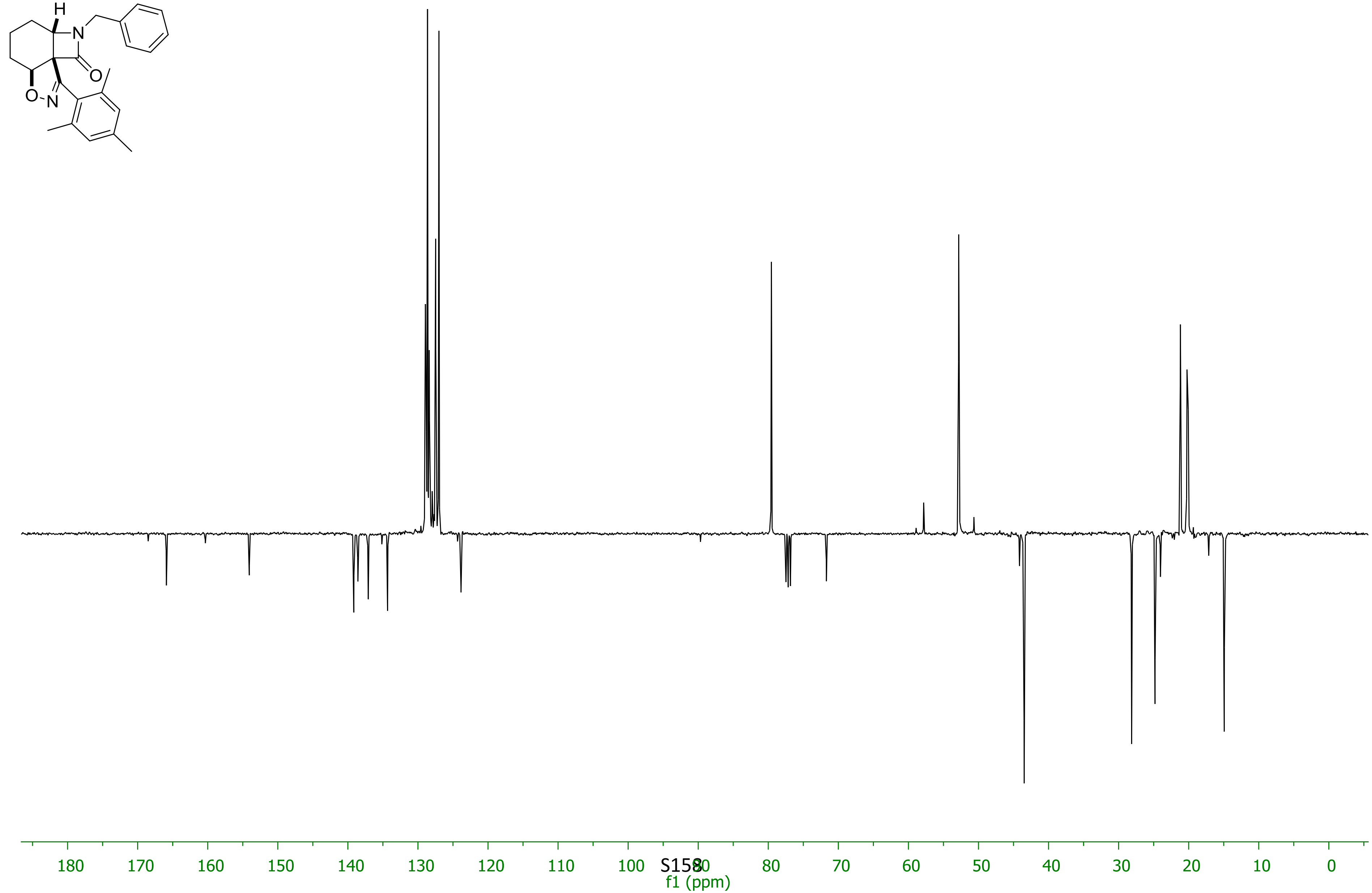

Supplement: Supplementary file 1 [file chem0020-6505-sd1.pdf]
